# iPSC-derived reactive astrocytes from patients with multiple sclerosis protect cocultured neurons in inflammatory conditions

**DOI:** 10.1172/JCI164637

**Published:** 2023-07-03

**Authors:** Janis Kerkering, Bakhrom Muinjonov, Kamil S. Rosiewicz, Sebastian Diecke, Charlotte Biese, Juliane Schiweck, Claudia Chien, Dario Zocholl, Thomas Conrad, Friedemann Paul, Marlen Alisch, Volker Siffrin

**Affiliations:** 1 Experimental and Clinical Research Center, Charité – Universitätsmedizin Berlin and Max Delbrück Center for Molecular Medicine in the Helmholtz Association, Berlin, Germany.; 2 Technology Platform Pluripotent Stem Cells, Max Delbrück Center for Molecular Medicine in the Helmholtz Association, Berlin, Germany.; 3 Institute of Biochemistry and; 4 Institute of Biometry and Clinical Epidemiology, Charité – Universitätsmedizin Berlin, Berlin, Germany.; 5 Genomics Technology Platform, Max Delbrück Center for Molecular Medicine in the Helmholtz Association, Berlin, Germany.; 6 Department of Neurology, Charité – Universitätsmedizin Berlin, Berlin, Germany.

**Keywords:** Inflammation, Stem cells, Multiple sclerosis, Neurodegeneration, iPS cells

## Abstract

Multiple sclerosis (MS) is the most common chronic central nervous system inflammatory disease. Individual courses are highly variable, with complete remission in some patients and relentless progression in others. We generated induced pluripotent stem cells (iPSCs) to investigate possible mechanisms in benign MS (BMS), compared with progressive MS (PMS). We differentiated neurons and astrocytes that were then stressed with inflammatory cytokines typically associated with MS phenotypes. TNF-α/IL-17A treatment increased neurite damage in MS neurons from both clinical phenotypes. In contrast, TNF-α/IL-17A–reactive BMS astrocytes cultured with healthy control neurons exhibited less axonal damage compared with PMS astrocytes. Accordingly, single-cell transcriptomic BMS astrocyte analysis of cocultured neurons revealed upregulated neuronal resilience pathways; these astrocytes showed differential growth factor expression. Furthermore, supernatants from BMS astrocyte/neuronal cocultures rescued TNF-α/IL-17–induced neurite damage. This process was associated with a unique LIF and TGF-β1 growth factor expression, as induced by TNF-α/IL-17 and JAK-STAT activation. Our findings highlight a potential therapeutic role of modulation of astrocyte phenotypes, generating a neuroprotective milieu. Such effects could prevent permanent neuronal damage.

## Graphical abstract

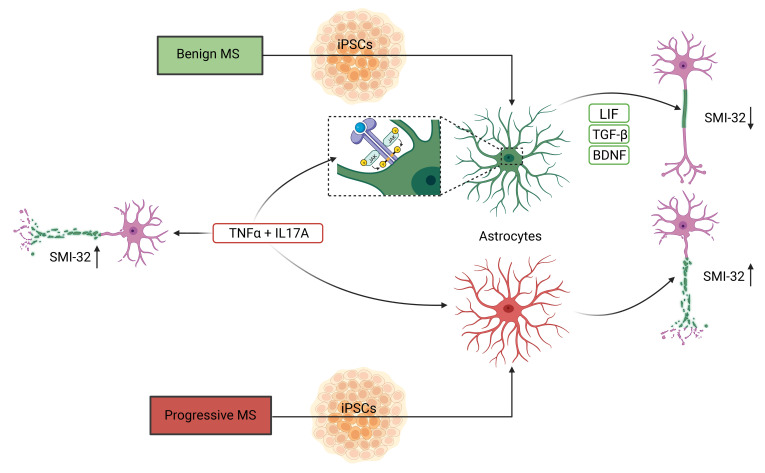

## Introduction

The major multiple sclerosis (MS) research advances involve inflammatory mechanisms. With new knowledge, effective antilymphocyte treatments were developed. These therapies reduced or terminated MS relapses and MRI activity. However, life-threatening opportunistic infections and cancer also developed (1). Despite treatment, many MS patients progress to disability. The deterioration correlates with slowly expanding preexisting MRI lesions (2). Peripheral immune cells are commonly scarce, or even absent, in these sites, although ICAM-1 and MHC-II are strongly upregulated on adjacent glial cells. This state of affairs indicates ongoing active inflammation driven by central nervous system–endogenous cells (3). We strove to better understand the underlying endogenous central nervous system (CNS) inflammatory mechanisms. We hypothesized that the process involves neurodegeneration with simultaneous neuro-regeneration (4). Not only proinflammatory but also potentially neuroprotective and regenerative mechanisms could be inherent in the astrocytes of these MS patients. Such a state of affairs could explain the heterogeneous individual MS outcomes. In early MS, astrocytes are involved and have multiple potential roles in damage and repair (5). Furthermore, astrocytes have been described as a major component in other neurological diseases (6), which highlights their central role supporting a sound neuronal network activity and CNS integrity. We focused on two MS clinical phenotype extremes, benign MS (BMS) (7) and progressive MS (PMS), as defined by large observational cohorts from before the treatment era (8). Since induced pluripotent stem cell–derived (iPSC-derived) tissue-specific culture models have added valuable research tools (9), we investigated BMS- and PMS-derived neurons and astrocytes. The cells were generated from iPSCs after differentiation to neural stem cells (NSCs). TNF-α and IL-17A represent part of the inflammatory milieu in cerebrospinal fluid and CNS lesions (10, 11) and reflect disease outcomes (12). Thus, these cytokines received our particular attention.

## Results

### Generation of neurons and astrocytes.

**Figure 1:**
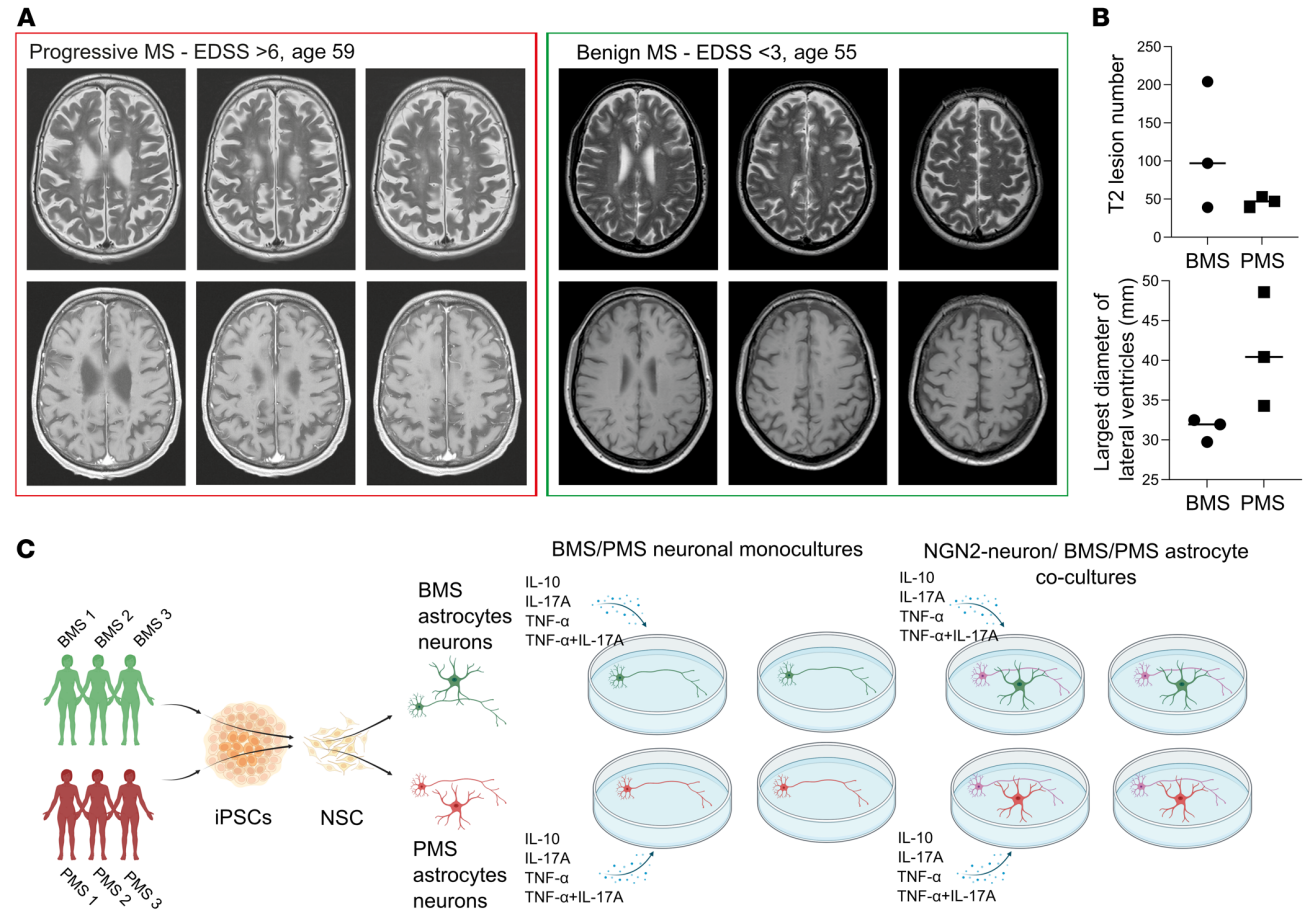
Study overview. (**A**) Representative MRI comparing progressive with benign MS, showing comparable T2 hyperintense absolute lesion counts. (**B**) Lesion count and lateral ventricle maximal diameter of BMS and PMS patients in this study. The absolute tissue loss is increased in PMS as shown by the maximal width of lateral ventricles. (**C**) Schematic overview of this study.

**Figure 2:**
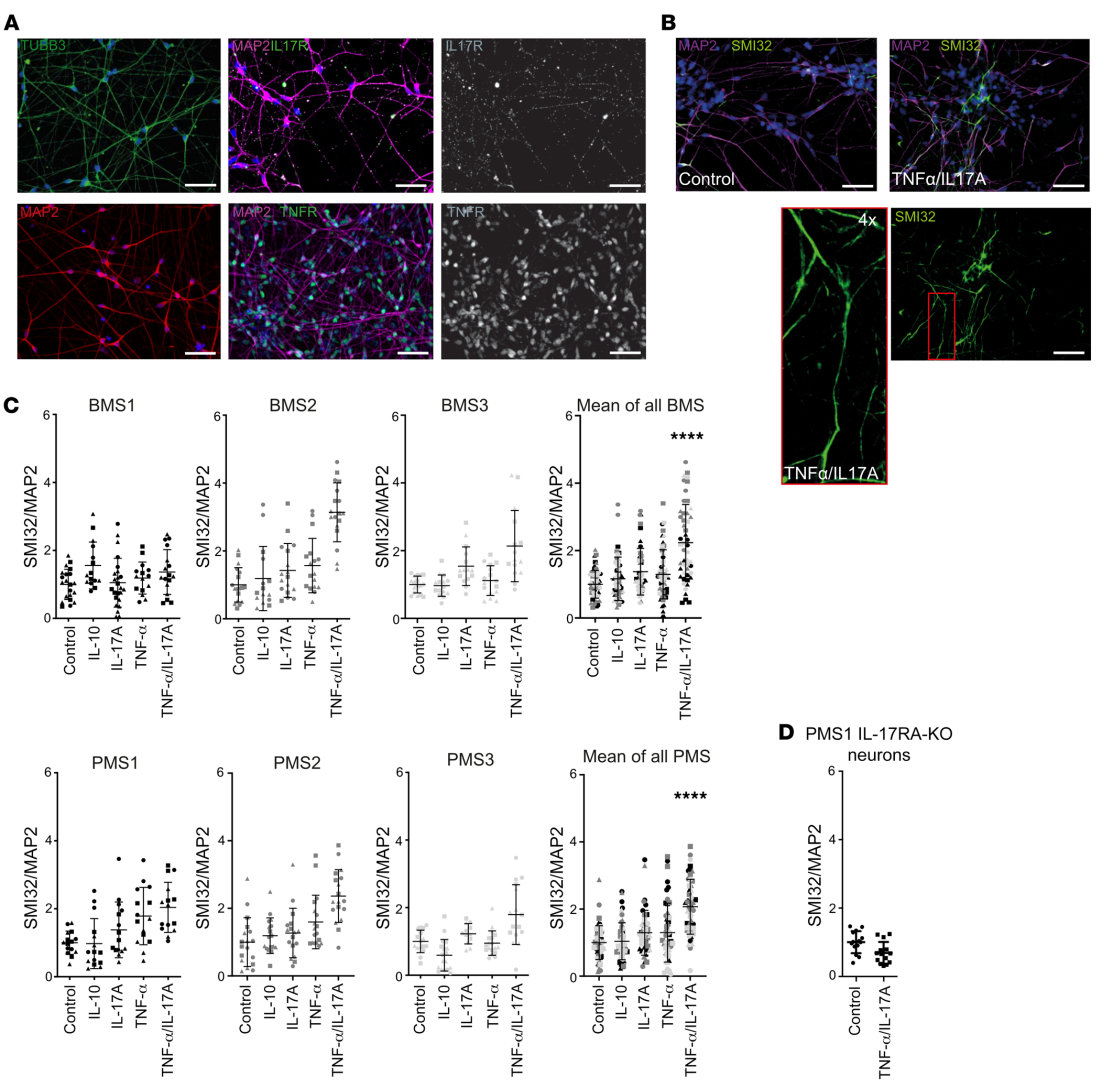
MS patient neurons without and with exposure to inflammatory cytokines. (**A**) Immunofluorescence staining of iPSC-derived neurons. Mature neurons differentiated for 3 weeks stain positive for β_III_-tubulin, MAP2, IL-17RA, and TNFR1. Scale bars: 50 μm. (**B**) Representative images of iPSC-derived neurons treated with TNF-α/IL-17A for 24 hours (50 ng/mL). Cells were fixed and immunofluorescently stained against MAP/SMI32. Scale bars: 50 μm. (**C**) iPSC-derived neurons were differentiated, and monocultures of neurons were treated with cytokines for 24 hours (50 ng/mL). Immunofluorescently stained MAP/SMI32 neurons were analyzed with ImageJ and are presented as surface ratio of MAP/SMI32 ± SD, normalized to the control. In 5 of 6 neurons the ratio was increased after TNF-α/IL-17A, irrespective of their disease phenotype. (**D**) CRISPR/Cas9 knockout of IL-17RA in PMS1. No increase of MAP/SMI32 was detectable after TNF-α/IL-17A treatment. Each data point represents a microscopic field of view (641 × 479 μm) of 3 independent experiments depicted by different symbols; pooled data represent the mean from 3 patients and 3 independent experiments. Statistical significance was tested with a Kruskal-Wallis test; ****P < 0.0001.

**Figure 3:**
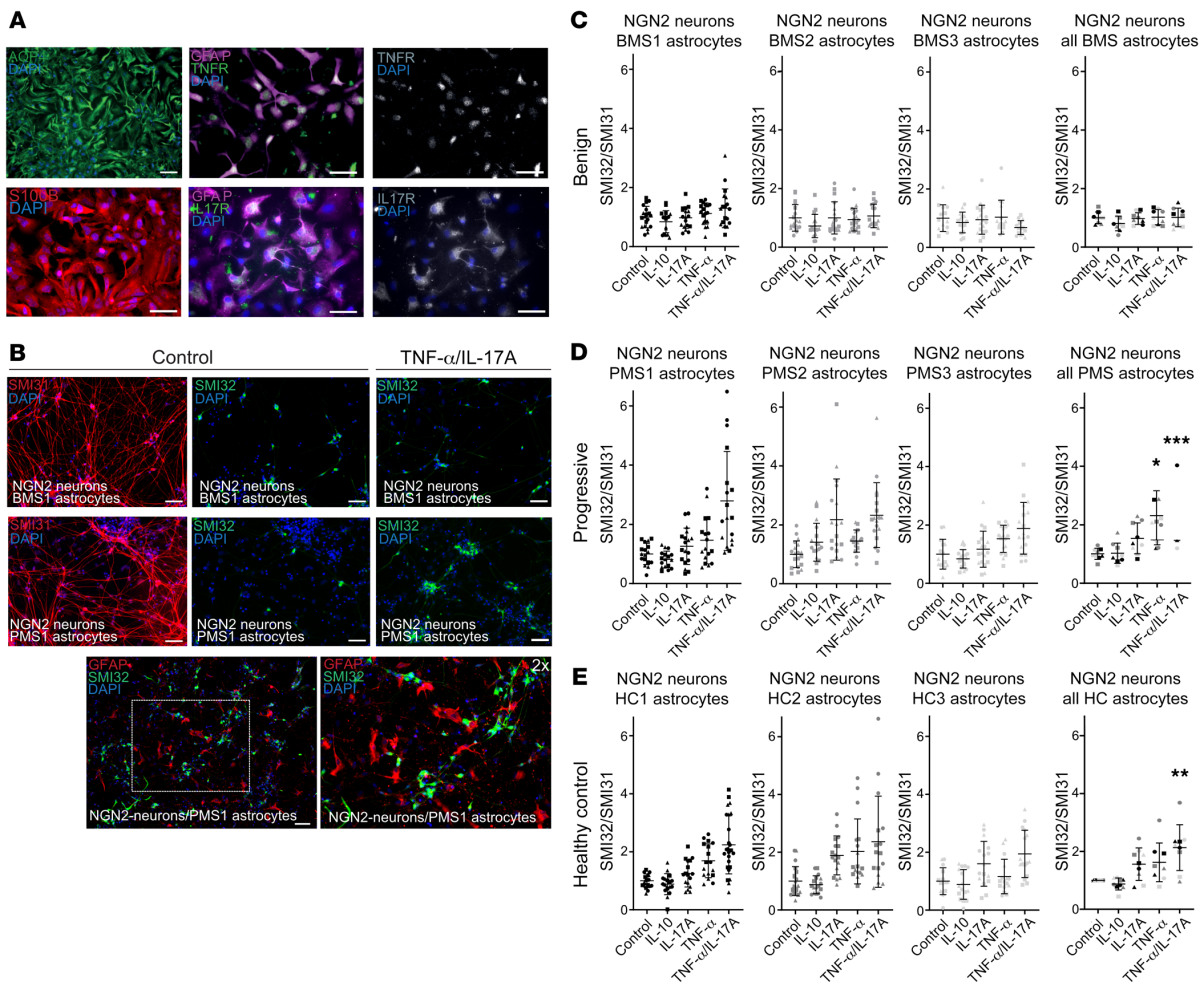
MS patient astrocytes and cocultures with NGN2 neurons without and with exposure to inflammatory cytokines. (**A**) Immunofluorescence staining of iPSC-derived astrocytes. Mature astrocytes differentiated for 6 weeks stain positive for GFAP, s100β, AQP4, IL-17R, and TNFR1. Scale bars: 50 μm. (**B**) iPSC-derived astrocytes cocultured with NGN2-neurons show close colocalization. Treatment with TNF-α/IL-17A for 24 hours (50 ng/mL) increased SMI32/SMI31 ratio in NGN2-neurons cultured with PMS astrocytes. Scale bars: 50 μm. Immunofluorescently stained SMI32/SMI31 neurons were analyzed with ImageJ and are presented as surface ratio of SMI32/SMI31 ± SD, normalized to the control. (**C** and **D**) Neurons in coculture with BMS astrocytes (**C**) were protected against TNF-α/IL-17A exposure, whereas PMS astrocytes (**D**) did not show protection against TNF-α and TNF-α/IL-17A. (**E**) Neurons in coculture with healthy control astrocytes (HC1, HC2, HC3) also showed increased SMI32/SMI31 ratios after TNF-α/IL-17A exposure. Each data point represents a microscopic field of view (641 × 479 μm) of 3 independent experiments depicted by different symbols; pooled data represent the mean from 3 individual patients (different colors) and 3 independent experiments (different symbols). Statistical significance was tested with a Kruskal-Wallis test; *P < 0.05, **P < 0.01, ***P < 0.001.

We recruited MS patients with either BMS or PMS (Table 1). The definition of BMS included an Expanded Disability Status Scale (EDSS) score less than 3 with full earning ability and a disease duration of over 15 years (7). The rapidly disabling phenotype was defined by a rapid disability accumulation (EDSS > 6) within 15 years of disease duration. All patients were not under current immunosuppressive treatment at the time point of sampling. MRI scans of the MS patients revealed comparable T2 hyperintense absolute lesion counts as a marker of comparable disease state (Figure 1A), whereas the absolute tissue loss differed as shown by the maximal width of lateral ventricles between the patient groups (Figure 1B). To analyze potential CNS-endogenous mechanisms of the divergent degree of neuroaxonal damage in BMS patients compared with PMS patients, we generated patient-specific CNS cell cultures (Figure 1C). Thus, iPSCs were generated according to established protocols from peripheral blood mononuclear cells and characterized in terms of pluripotency, normal karyotype, and line identity by short tandem repeat (Human Pluripotent Stem Cell Registry, https://hpscreg.eu). Subsequently, iPSCs were differentiated to NSCs by an adjusted published protocol (13), and the expression of NSC markers was characterized (Supplemental Figure 1A; supplemental material available online with this article; https://doi.org/10.1172/JCI164637DS1). Mature patient-specific neurons and astrocytes were differentiated from these NSCs as previously reported by our group (14). Neurons were differentiated for 3 weeks and characterized by immunofluorescence for β_III_-tubulin and MAP2 (Figure 2A) and quantitative PCR (qPCR) (Supplemental Figure 1B). Spontaneous neuronal activity as shown by Ca^2+^ imaging confirmed functionality (Supplemental Figure 1C). Astrocytes were differentiated for 6 weeks, and their maturity was confirmed by analysis of maturity markers in immunofluorescence (S100β, GFAP, AQP4; Figure 3A) and qPCR (Supplemental Figure 1D). Astrocytic functional activity was confirmed by the presence of characteristic wave-like spontaneous Ca^2+^ transitions between adjacent cells (Supplemental Figure 1E).

### TNF-α/IL-17A treatment induces neurite damage irrespective of disease course.

Tissue inflammation involves the presence of distinct proinflammatory mediators, namely cytokines. This inflammatory milieu is a major contributor to neuroaxonal damage (15). According to our previous findings regarding TNF-α and IL-17A, which induced damage of neurites in embryonal stem cell–derived neurons (16), we used this system for BMS and PMS patient–derived neurons. First, we analyzed the cytokine receptor expression of TNF receptor 1 (TNFR1) and IL-17 receptor (IL-17R), which we confirmed to be ubiquitously present on cultured neurons (Figure 2A and Supplemental Figure 7). Next, neuronal monocultures of BMS and PMS patients were exposed to IL-10, IL-17A, or TNF-α and to a combination of TNF-α and IL-17A. We analyzed non-phosphorylated neurofilament (SMI32), which has been used in many studies as a surrogate marker for neuroaxonal damage (17). Application of vincristine, an inhibitor of tubulin polymerization, confirmed SMI32 as a suitable neuroaxonal damage marker in our neuron cultures (Supplemental Figure 2A). Single cytokine treatments of BMS and PMS patient–derived neuronal cultures had no consistent effect on SMI32/MAP2 immunofluorescence. However, the combination TNF-α/IL-17A induced an increase in SMI32/MAP2 ratio (Figure 2B). This increase in SMI32/MAP2 was detectable in 5 of 6 MS-specific lines (2 BMS, 3 PMS) and statistically significant for the pooled samples in each group (Figure 2C). We investigated one of these lines (PMS1) to determine whether disrupting IL-17R signaling could inhibit the axonal damage formation. Indeed, there was no increase of SMI32/MAP2 in neurons carrying a (CRISPR/Cas9–mediated) IL-17RA knockout (Supplemental Figure 2B) and treated with TNF-α/IL-17A (Figure 2D).

### Astrocytes from BMS patients protect neurons from cytokine-induced neurite damage.

Next, we investigated astrocytes differentiated from BMS and PMS patients in context with human neurons. First, we confirmed also for astrocytes the expression of the cytokine receptors for IL-17A and TNF-α (Figure 3A). To limit confounding factors driven by variable neuron subpopulations in these neuronal coculture experiments with patient-specific astrocytes, we used an inducible control iPSC line derived from a healthy control person without reported disease (18) containing an NGN2-mediated overexpression system for rapid direct transformation into neurons (19). NGN2-induced neurons are well characterized and widely used in the community (20–22), forming a homogeneous and reproducible glutaminergic phenotype similar to that of layer 2/3 cortical neurons based on expression of the markers vGlut2, Cux1, and Brn2 (19). To investigate the role of patient-specific astrocytes in the context of cytokine-mediated neurite damage, we established a coculture setup of these NGN2-neurons with astrocytes from BMS or PMS patients. MS patient iPSC–derived astrocytes were seeded on NGN2-neurons at day 7 of neuronal differentiation, which resulted in a close colocalization of astrocytes with neurons and axons at day 10 (Figure 3B). These NGN2-neuron/MS astrocyte cocultures were subsequently treated with the same set and conditions of cytokines as for the neuronal monocultures, and neuroaxonal damage was quantified by SMI32/SMI31 ratio, SMI31 representing phosphorylated neurofilament as found in stable neurites (23). Here, a differential effect was seen between BMS- and PMS-derived astrocytes: Neurons in coculture with the astrocytes of BMS were protected from an increase in SMI32/SMI31 after TNF-α/IL-17A treatment (Figure 3C). Neurons cultured together with astrocytes of PMS exhibited significantly increased SMI32/SMI31 after TNF-α/IL-17A exposure (Figure 3D). To place these results in perspective, we included cocultures of NGN2-neurons with astrocytes derived from healthy control (HC) persons’ iPSCs (HC1, HC2, HC3). These cocultures showed increased SMI32/SMI31 ratio after TNF-α/IL-17A treatment, comparable to the PMS phenotype (Figure 3E). Thus, astrocytes derived from patients with a progressive disease course or HC persons could not protect from cytokine-mediated neurite damage. In contrast, astrocytes from benign patients were able to outbalance the negative effect of TNF-α/IL-17A treatment on neurites. We also performed experiments with MS-derived astrocytes in coculture with autologous neurons (instead of NGN2-neurons). These experiments generally showed similar results (Supplemental Figure 3) but were burdened by higher variances. This result is most likely due to lower homogeneity of neuron populations under these autologous cocultures. The HC experiments indicate that we observed a special protective phenotype in BMS astrocytes rather than a neurodegenerative or neurotoxic phenotype in PMS patients.

### Single-cell transcriptomic analyses of TNF-α/IL-17A–treated cocultures.

**Figure 4:**
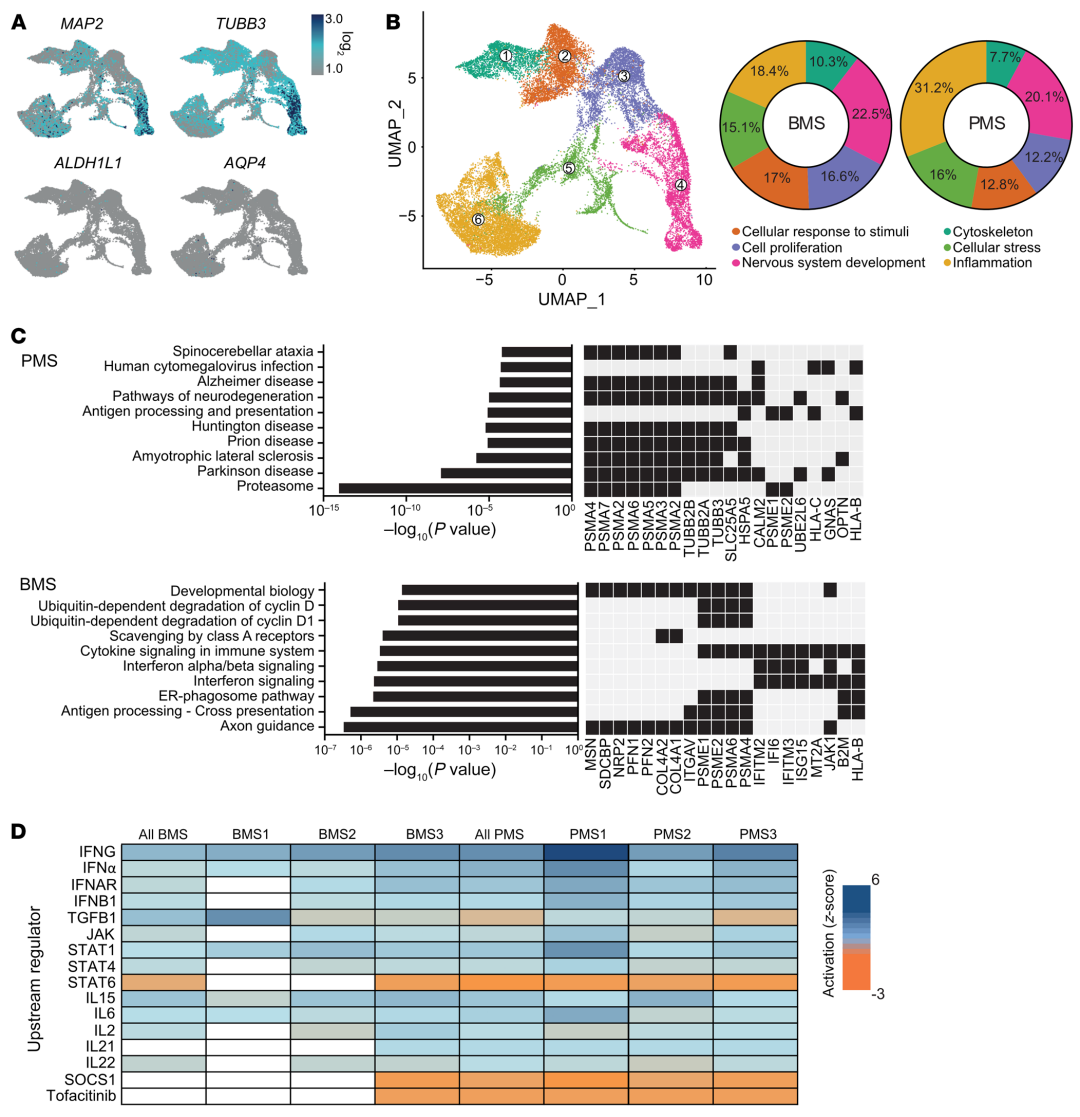
Single-cell transcriptome analysis of MS patient astrocyte/NGN2 neuron cocultures. Cells were treated for 24 hours with TNF-α/IL-17A or left untreated (control) and harvested with Accutase. Single-cell suspensions of 6 technical repeats per sample were filtered, and subsamples were labeled with CMOs to allow pooling for multiplexed libraries. (**A**) UMAP of all cells with projection of expression of MAP2 and TUBB3 (neuronal markers) and ALDH1L1 and AQP4 (astrocyte markers). (**B**) UMAP and pie chart to analyze the proportional distribution of cells in the color-coded subclusters, which were named according to the most prevalent defining genes (ranked by log_2_ fold change) and their prospective function. Neurons of PMS cultures were found to be higher in the inflammation cluster compared with BMS. (**C**) Enrichr analysis of KEGG 2021 and Reactome 2016 pathways comparing PMS and BMS. A black box represents the presence of a gene. (**D**) IPA analysis comparing samples according to potential upstream mediators (short list; for complete analysis see Supplemental Table 1). The *z* score represents the activation score of a predicted regulator based on the expression of the input genes; a positive score represents an activation and a negative score an inhibition. Each column of BMS or PMS represents the data set of 1 experiment (TNF-α/IL-17A treated vs. control); “all BMS” and “all PMS” are the mean of samples BMS1–BMS3 or PMS1–PMS3, respectively. The analysis predicted a JAK/STAT activation and a negative score for tofacitinib in all PMS samples.

To investigate the mechanisms that led to the observed differential effect between the two MS phenotypes, we used single-cell RNA sequencing (scRNA-Seq) to identify specific changes in the transcriptome upon treatment with TNF-α/IL-17A. Neuron/MS patient–derived iPSC astrocyte cocultures of TNF-α/IL-17A–treated or untreated samples were dissociated, washed, filtered, and tagged with individual Cell Multiplexing Oligos (CMOs) for single-cell analysis according to the manufacturer’s protocol. The aim was to CMO-tag 2,500 cells of every sample to allow pooling of 4 subsamples to reach a targeted cell recovery of 10,000 cells. Sequencing was performed on an Illumina NovaSeq1. We performed unbiased clustering using uniform manifold approximation and projection for dimension reduction (UMAP) of the scRNA-Seq data and correlated this cell cluster analysis with expression of established markers for neurons and astrocytes (Figure 4A). The cluster identity analysis revealed that most sequenced cells were of neuronal subtype and hardly any of an astrocyte identity (see Supplemental Table 1 for cluster marker gene expression). This finding is most likely due to clumping of astrocytes, which formed syncytia in cocultures that could not be dissociated in single-cell suspensions.

The analyzed cells in the biggest subcluster shared genes of inflammatory processes (Figure 4B). Subcluster analysis between PMS and BMS revealed major differences in proportion for the cells in the inflammation, cellular response to stimuli, and cell proliferation subclusters (Figure 4B). Analysis of the top 50 differentially expressed genes (DEGs) in the inflammation subcluster between the individual patient-derived TNF-α/IL-17A–treated samples (Supplemental Figure 3) revealed, among others, *CDH2*, *WLS*, and *NRP2* to be more highly expressed in BMS compared with PMS; these genes are known to express proteins involved in signal transduction, such as NRP2, involved in regulating axon guidance (24). *PMP22*, *IFITM1*, and *CAV1* were more highly expressed in TNF-α/IL-17A–treated PMS. *IFITM1* has been described as an important type I interferon–dependent antiviral effector (25), indicating inflammatory activation.

Enrichment analysis (Kyoto Encyclopedia of Genes and Genomes [KEGG]) revealed several activated neurodegenerative pathways involved in Parkinson’s disease or amyotrophic lateral sclerosis and proteasome activation (Figure 4C), as several proteasome-related genes, such as proteasome 20S subunits α and β (*PSMA2/3/5*, *PSMB2/4/6/7*), were upregulated in PMS astrocyte–cocultured neurons. In BMS (Figure 4C), processes of neuroprotection and neuroregeneration were activated, such as axon guidance (*MSN*, *NRP2*, *PFN1*, *PFN2*, *JAK1*, and others) and pathways of innate immune responses, for example IFN-α/β signaling (*IFITM2*, *IFI6*, *ISG15*).

Analysis of upstream factors by Ingenuity Pathway Analysis (IPA) gives indirect clues to the respective mechanisms by comparing the DEGs with previously published mechanistic studies. We identified — among others (see also Supplemental Table 2) — possible neuroprotective upstream mediators predominantly present in the BMS group, such as erythropoietin (EPO) and brain-derived neurotrophic factor (BDNF) or IL-9. Use of IPA to compare TNF-α/IL-17–treated groups between PMS and BMS and explore druggable targets revealed JAK and STAT among others (Figure 4D and Supplemental Table 3). Notably, the JAK/STAT signaling pathway as well as JAK/STAT–regulating cytokines (IFN-γ, IFN-α, IL-15, IL-6, IL-2, IL-21) were differentially regulated in the PMS group compared with BMS. In line with these findings, the JAK1/2 inhibitor SOCS1 was predicted to be inhibitory and the pharmacological JAK inhibitor tofacitinib was identified as a possible regulator in the PMS versus the BMS group.

### Bulk sequencing demarcates benign from progressive phenotype.

**Figure 5:**
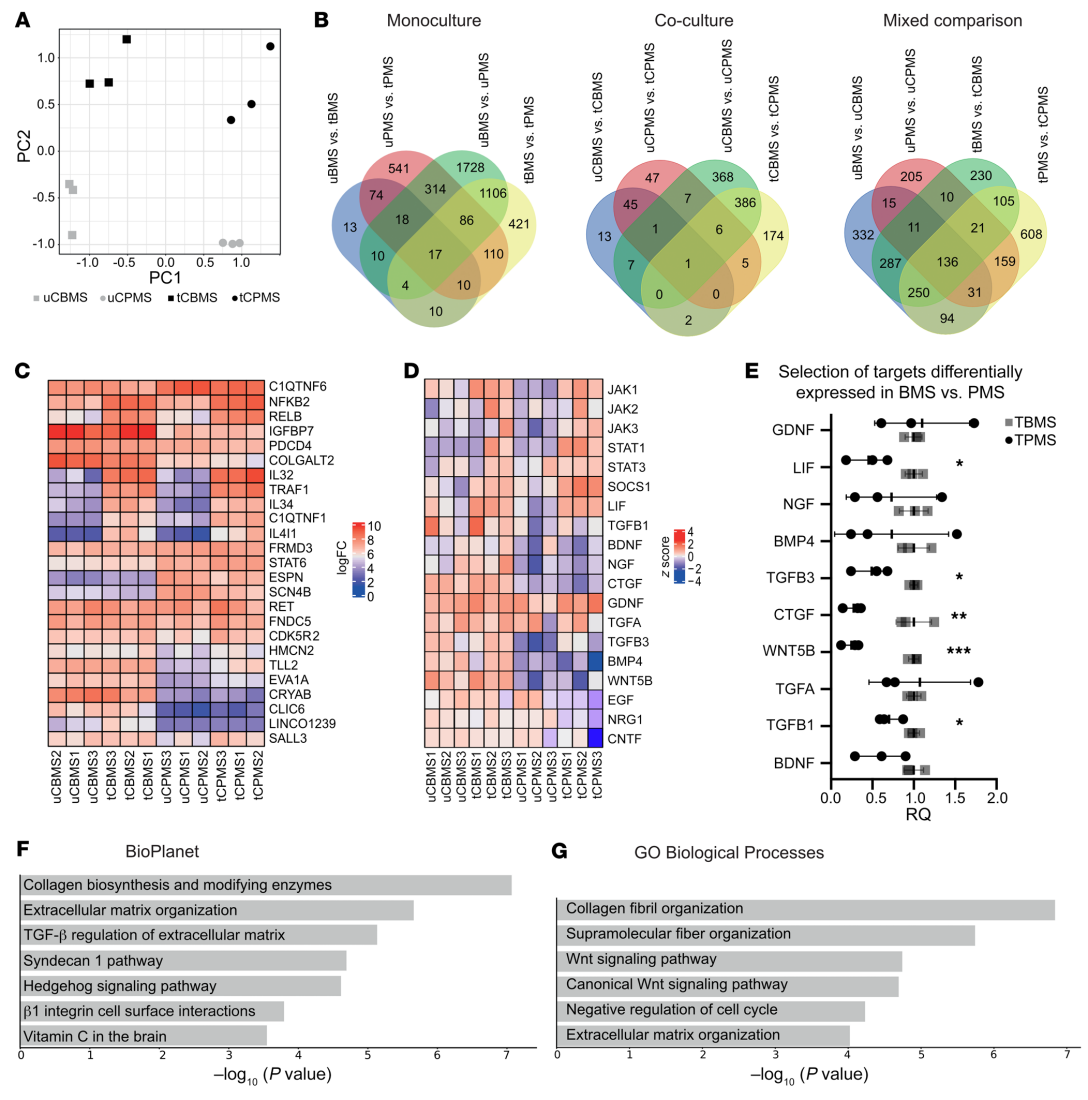
Bulk transcriptome analysis of MS patient astrocytes from monocultures and cocultures with NGN2 neurons. Cells were treated for 24 hours with TNF-α/IL-17A or left untreated (control). Neurons were washed off, and remaining astrocytes were used for further processing. (**A**) PCA plot of bulk RNA-Seq of coculture-derived astrocytes shows distinct segregation of samples according to patient group (PC1) and according to cytokine treatment versus control (PC2). (**B**) Venn chart representing shared and unique genes comparing mono- and coculture-derived astrocytes. (**C**) Heatmap showing the top 25 regulated genes ranked by the adjusted *P* value. (**D**) Heatmap representing expression of genes encoding neurotrophic and JAK/STAT–related factors. The *z* score was calculated including all 12 samples. (**E**) Reverse transcriptase qPCR of selected targets confirmed differentially expressed genes between BMS and PMS after treatment. Statistical significance was tested with an unpaired 2-tailed *t* test; *P < 0.05, **P < 0.01, ***P < 0.001. (**F** and **G**) Enrichr analysis of activated pathways (**F**) and Gene Ontology (GO) labels of biological processes (**G**) comparing tCBMS versus tCPMS.

scRNA-Seq of cocultures resulted in overrepresentation of neurons after the isolation of single cells, due to astrocytes’ biological property of being an extensively branched and entangled cell subset that is difficult to dissociate. Alternatively, to avoid losing any information, we performed bulk sequencing of astrocytes from monocultures, as well as astrocytes of cocultures with NGN2-neurons, from which we washed off the neurons before sample harvesting. A principal component analysis revealed clustering of BMS- and PMS-derived astrocytes between untreated (u) BMS and uPMS, as well as treated (t) versus untreated cells, both monoculture- and coculture-derived (C) astrocytes (Figure 5A and Supplemental Figure 5A). Looking at shared DEGs between the astrocyte groups, the largest differences were found between patient groups. In monoculture, the majority of DEGs (1,728 genes) were seen in the group of uBMS versus uPMS (Figure 5B, left); treatment led to a change of 421 DEGs in the group of tBMS versus tPMS, indicating a clear separation of patient subgroup–derived astrocytes. In coculture (Figure 5B, middle), the differences of DEGs were overall lower in number, but again highest between the different patient group samples (uCBMS vs. uCPMS, 368 genes; tCBMS vs. tCPMS, 174 DEGs). Cytokine treatment itself had distinct effects mainly in the cocultures (47 DEGs for uCPMS vs. tCPMS; 13 DEGs for uCBMS vs. tCBMS; 45 shared DEGs). A mixed comparison of DEGs between monoculture- and coculture-derived cells for sequencing highlights the general difference between these 2 culture models (Figure 5B, right). Comparison of untreated monoculture versus untreated cocultures showed a difference in DEGs of 332 genes and 205 genes for uBMS versus uCMS and uPMS versus uCPMS, respectively. The same holds true for the observation of the treated monoculture versus its treated coculture, in which 230 DEGs were found between tBMS and tCBMS and 608 DEGs between tPMS and tCPMS. Overall, these results highlight the more homogeneous results of cocultured astrocytes, but also the clear separation between BMS- and PMS-derived astrocytes.

By comparing the top 25 regulated genes (ranked by adjusted *P* value) between patient groups, we identified a clear distinction between cocultured BMS and PMS astrocytes in homeostasis but also after TNF-α/IL-17A treatment (Figure 5C). Focusing on general differences between both phenotypes, we could observe, for example, that in treated cocultured BMS (tCBMS) astrocytes, the TGF-β–dependent IGF-binding protein 7 (IGFBP7) — a marker previously described in cerebrospinal fluid of MS patients (26) — was more strongly upregulated than in tCPMS. CRYAB — a marker of the cellular stress response and previously described in immunomodulation in MS (27) — had a substantially higher expression in CBMS than in CPMS.

Focusing on JAK/STAT–related genes, we found the basal expression of JAK1 and JAK3 to be higher in uCBMS compared with uCPMS (Figure 5D). Treated astrocytes showed increased *JAK1*, *JAK2*, and *JAK3* expression in both phenotypes; contrastingly, *SOCS1* expression was higher in tCPMS. The downstream genes *STAT1* and *STAT3* were also upregulated after treatment in both phenotypes. *TGFβ1*, *TGFβ3*, and *LIF* expression were markedly lower in the PMS phenotype. Neurotrophic factors such as *BDNF*, *CNTF*, *NRG1*, and *EGF* were all higher in BMS astrocytes compared with PMS. We controlled our observations with selected targets by qPCR (Figure 5E). A pathway analysis of DEGs (Figure 5F) comparing tCBMS and tCPMS astrocytes revealed activated pathways involved in collagen biosynthesis and modifying enzymes, extracellular matrix (ECM) organization, and TGF-β regulation of the ECM. The top 3 Gene Ontology labels of biological processes were collagen fibril organization, supramolecular fiber organization, and Wnt signaling pathway (Figure 5G). In addition, a pathway analysis of untreated astrocytes showed various pathways regulated by nervous system development, such as activation of axon guidance, NCAM signaling for neurite outgrowth, and dendrite morphogenesis, in BMS astrocytes (Supplemental Figure 5B). Thus, TNF-α/IL-17A–treated BMS astrocytes in cocultures were activated via numerous signaling pathways in which we identified JAK/STAT as a major driver, thereby upregulating various soluble neurotrophic factors such as LIF, TGF-β, and BDNF.

### BMS astrocyte–produced soluble mediators are protective.

**Figure 6:**
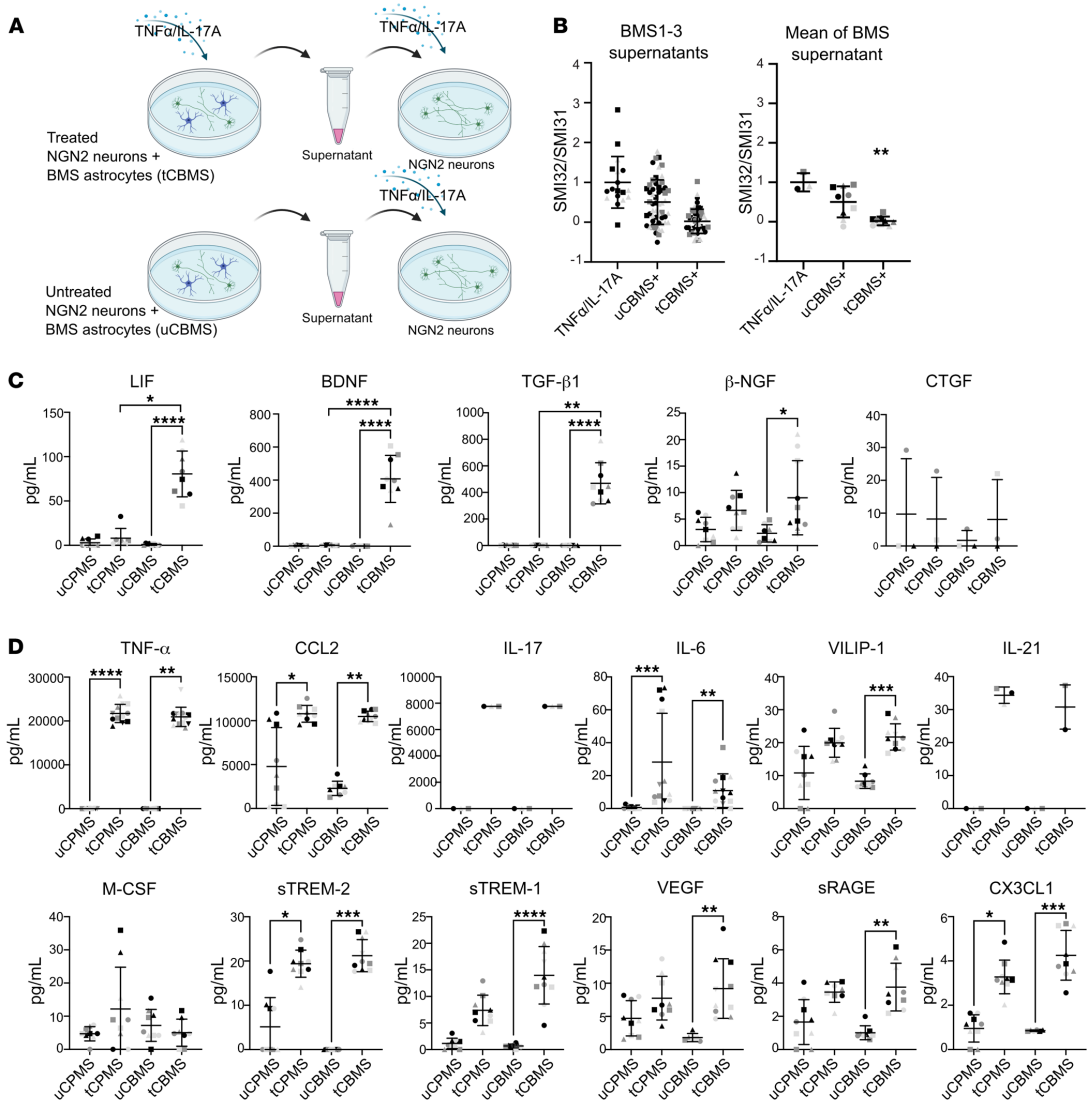
Supernatant analysis of MS patient astrocyte/NGN2-neuron cocultures. (**A**) Supernatants of TNF-α/IL-17A–treated and untreated NGN2 and BMS astrocytes were collected after 24 hours of stimulation and applied on monocultures of TNF-α/IL-17A–preincubated NGN2-neurons. (**B**) NGN2-neurons were treated with TNF-α/IL-17A (50 ng/mL) for 24 hours (represented by “+”), and medium was replaced with untreated or treated coculture supernatants. Each data point represents a microscopic field of view (641 × 479 μm) of 3 independent experiments depicted by different symbols; pooled data show the mean from 3 patients (different colors) and 3 independent experiments (different symbols). Neurons showed protection when given supernatants of treated BMS cocultures. “+” represents an arbitrary proportion of the target in tBMS compared with tPMS; “=” indicates an equal level. (**C** and **D**) Bead-based multiplex assay and ELISA (CTGF) analysis of supernatants. LIF, BDNF, and TGF-β1 concentrations were significantly higher in tCBMS than in tCPMS. Each data point represents 1 sample collectively of 3 individual patients (different colors) and 3 independent experiments (different symbols). Statistical significance was tested with a Kruskal-Wallis test; *P < 0.05, **P < 0.01, ***P < 0.001, ****P < 0.0001.

To test whether the neuroprotective effect of BMS astrocytes was dependent on direct astrocyte-neuron contact or driven by soluble factors, we next used conditioned medium of BMS astrocyte/NGN2-neuron cocultures to treat TNF-α/IL-17A–exposed NGN2-neurons (Figure 6A). No statistically significant differences were seen by repeat treatment of adjusted concentrations of TNF-α/IL-17A (Supplemental Figure 6). The analysis of the supernatant treatment of TNF-α/IL-17A–exposed NGN2-neurons revealed that all TNF-α/IL-17A–treated BMS-derived supernatants exhibited protection from SMI32/SMI31 upregulation in NGN2-neurons (Figure 6B). In contrast, supernatant derived from cocultures with HC astrocytes did not protect TNF-α/IL-17A–exposed NGN2-neurons (Supplemental Figure 9A). Based on these findings, we analyzed in depth the differences between tCBMS and tCPMS in our astrocyte bulk RNA-Seq data, concentrating on secreted factors that (a) were more highly expressed in tCBMS than in tCPMS, (b) are relevant as neurotrophic factors, and, secondarily, (c) have a connection to the JAK/STAT pathway. We narrowed down our selection to “classical” neurotrophic factors (BDNF, NGF, GDNF, LIF), mediators belonging to the TGF family (TGF-α, TGF-β1, TGF-β3, CTGF), and Wnt/BMP signaling factors (Wnt5b, BMP4) and confirmed by qPCR for most factors a higher expression in tCBMS (Table 2).

We next performed a multiplexed immunoassay supernatant analysis, including the factors LIF, NGF, BDNF, TGF-β1, and CTGF (Figure 6C). Here, we found that LIF, BDNF, and TGF-β1 concentrations were significantly higher in tCBMS compared with uCBMS and with tCPMS supernatants. NGF was marginally detectable, while CTGF was present in only 2 samples (BMS3 and PMS3) but with comparable levels after treatment. Evaluation of a neuroinflammation multiplexed assay panel revealed an increase of various analytes after treatment (Figure 6D). A substantial residual presence of TNF-α (20 ng/mL) and IL-17A (8 ng/mL) after 24 hours was confirmed in supernatants of treated cultures (initially 50 ng/mL for both cytokines). The TNF-α/IL-17A treatment induced high concentrations of the monocyte-attracting chemokine CCL2 and rather low concentrations of the inflammatory cytokines IL-6 and IL-21 and the chemokine CX3CL1. All these treatment-related changes of inflammatory mediators were without distinction between the patient groups. Notably, VILIP-1, VEGF, and sRAGE were exclusively enhanced in tCBMS but not in tCPMS, albeit in low concentration (<50 pg/mL). The inflammation mediators sTREM-1 and sTREM-2 were induced in both treated groups in comparison with the untreated cocultures. Supernatants of cocultures with HC astrocytes exhibited a similar increase in LIF concentration after treatment, high amounts of BDNF, but only traces of TGF-β1 (Supplemental Figure 9B). Taken together, neurite-protective supernatants from BMS patients contained significantly higher amounts of LIF, TGF-β1, and BDNF as potential mediators. Besides, BMS astrocytes presented as very active cells in secreting various mediators of inflammation after treatment.

### BMS astrocyte–mediated neurite protection is a JAK pathway–dependent process.

Next, we followed up on the observation that BMS-mediated neuroprotection was an active, TNF-α/IL-17A–dependent process. JAK pathway modulation via tofacitinib has been identified as a potential upstream event resulting in differences of BMS and PMS astrocyte cocultures in our scRNA-Seq analysis of cocultured neurons (Figure 4D). Therefore, we used the drug tofacitinib, mainly a JAK3 inhibitor with additional potency against JAK1 and JAK2.

**Figure 7:**
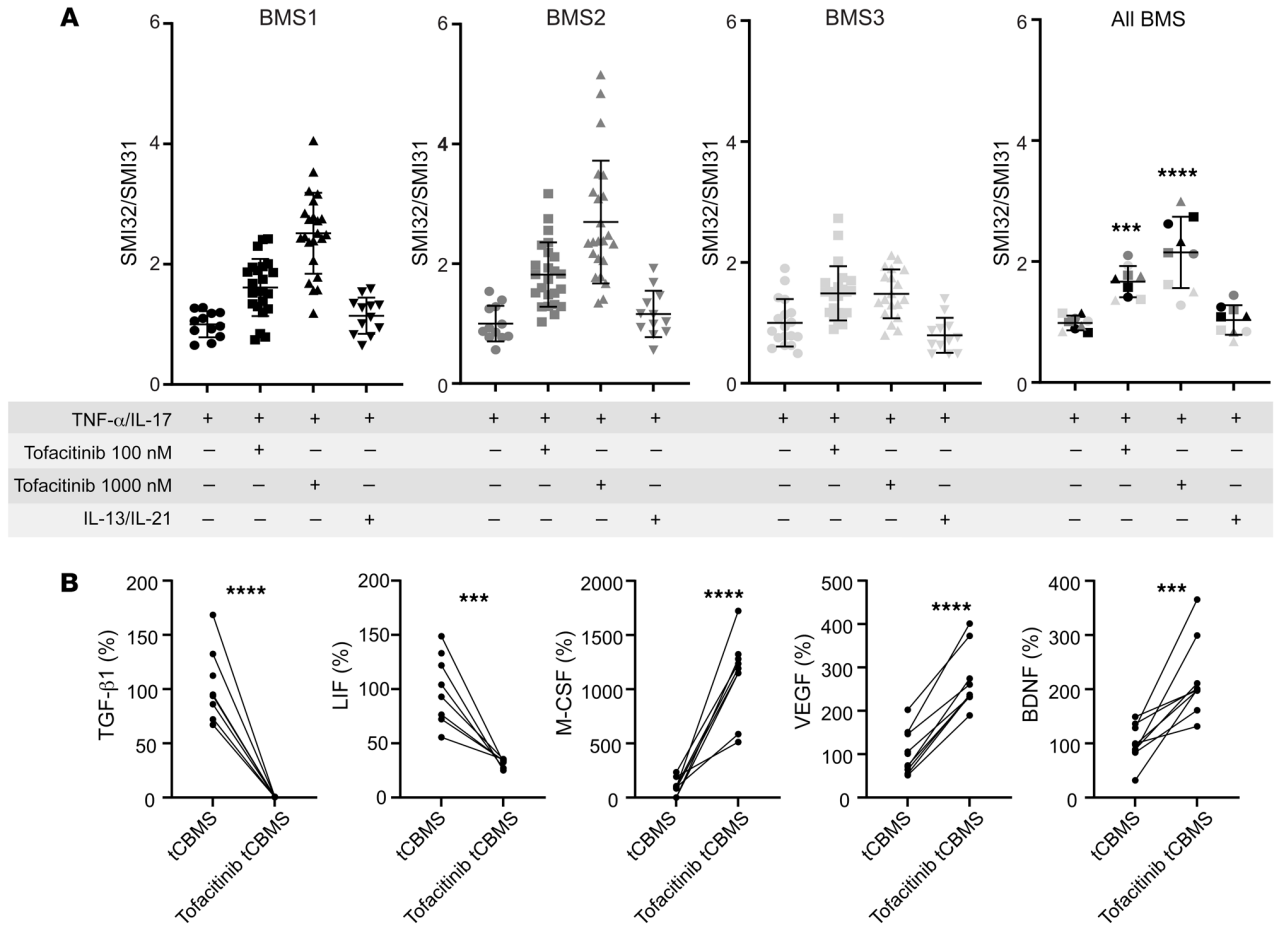
Blocking JAK/STAT with tofacitinib in MS patient astrocyte/NGN2-neuron cocultures. (**A**) iPSC-derived astrocytes cocultured with NGN2-neurons were treated with tofacitinib (100/1,000 nM) or IL-13 plus IL-21 (50 ng/mL each) for 24 hours, followed by a treatment with TNF-α/IL-17A (50 ng/mL) for 24 hours. Inhibition of JAK/STAT by tofacitinib led to failed neurite protection, as shown by an increase in SMI32/SMI31 ratio. Immunofluorescently stained SMI32/SMI31 neurons were analyzed with Imaris (Bitplane) and presented as surface ratio of SMI32/SMI31 ± SD, normalized to the control. Each data point represents a microscopic field of view (641 × 479 μm) of 3 independent experiments (different symbols); pooled data represent the mean from 3 individual patients (different colors) and 3 independent experiments (different symbols). Statistical significance was tested with a Kruskal-Wallis test; ***P < 0.001, ****P < 0.0001. (**B**) Supernatants of tofacitinib-treated samples were collected after the cytokine stimulation period of 24 hours. Relative changes of cytokine concentration in tofacitinib-treated versus control cocultures were analyzed. LIF and TGF-β1 levels were decreased, whereas M-CSF, VEGF, and BDNF were increased. Changes are displayed in percentage compared with (control) tCBMS (100%); each data point represents a single supernatant measurement. Statistical significance was tested with a Mann-Whitney *U* test; ***P < 0.001, ****P < 0.0001.

We tested the hypothesis that inhibiting the TNF-α/IL-17–dependent activation of JAK/STAT pathways in astrocytes would abolish the protective effect of BMS astrocytes in cocultures. To test this target, we preincubated cocultures with tofacitinib (100 nM and 1,000 nM) for 24 hours before adding the TNF-α/IL-17 treatment to evaluate differences in neuroprotection with JAK/STAT inhibition. Indeed, the inhibition of JAK/STAT activation led within the BMS cultures to significantly increased neurite damage as shown by higher SMI32/SMI31 ratios after TNF-α/IL-17A exposure, i.e., a loss of neuroprotection in these JAK/STAT–blocked astrocytes (Figure 7A). To monitor the effect of JAK/STAT activation, we included the cytokines IL-13, an activator of JAK1, and IL-21, a relevant JAK3 activator. In addition, IL-21 was found as an activating factor according to IPA analysis (Figure 4D). However, these factors did not change the SMI32/SMI31 ratio, i.e., there was no additive effect. Monocultures of NGN2-neurons treated with tofacitinib, in the absence of TNF-α/IL-17 stimulus, did not lead to significant changes in SMI32/SMI31 ratio (Supplemental Figure 8).

Accordingly, in the supernatant analysis we observed that in tofacitinib-treated tCBMS supernatants the elevated TGF-β concentration typical of BMS was almost completely lost. The LIF concentration was also significantly lower in comparison with tCBMS (Figure 7B). In contrast, M-CSF, VEGF, and BDNF concentrations were found to be even higher in tCBMS tofacitinib supernatants (Figure 7B). Thus, we found a TNF-α/IL-17A–induced upregulation of neurotrophic factors in BMS astrocytes cocultured with neurons via the JAK pathway. Inhibition of the JAK pathway modified the supernatants of these cocultures so that they were no longer sufficient to mediate the neurite-protective effect. We identified LIF, TGF-β1, and BDNF as having significantly higher concentrations in BMS-descended supernatants, and this difference was abolished for LIF and TGF-β1 after JAK inhibition. Therefore, interfering with the JAK/STAT pathway under TNF-α/IL-17A treatment suppressed the protective effect of BMS astrocytes in cocultures and identified LIF and TGF-β as critical molecular mediators.

## Discussion

We investigated novel neuroprotective immune-mediated mechanisms potentially underlying benign MS (BMS). We compared patients with a benign disease course with those patients who rapidly accumulated irreversible deficits, namely PMS. We used iPSC-derived neuronal and astrocyte cultures and cocultures. We found that astrocytes from BMS patients protected from TNF-α/IL-17–induced neurite pathology. This neuroprotective effect was an active response via TNF-α/IL-17–induced JAK/STAT signaling of astrocytes. Supernatants of these BMS astrocytes were neuroprotective and contained the soluble mediators LIF, BDNF, and TGF-β1.

Our major finding was the differential effect of BMS patient–derived as compared with PMS patient–derived or HC astrocytes on TNF-α/IL-17–mediated neurite damage. BMS-derived astrocytes were neuroprotective; HC and PMS astrocytes were not. In neuroinflammation, the major focus in astrocyte research has hitherto been on their neurotoxic role (28). For instance, astrocytes were able to critically elicit inflammatory CNS processes and neuronal damage formation by autocrine glycolipid-mediated astrocyte activation (29). Astrocytes stimulated with strong inflammatory mediators downstream of LPS signaling were shown to induce oligodendrocyte toxicity via saturated lipids (30). However, this extreme proinflammatory stimulus is at one end of the spectrum, and astrocyte reactivity can be very heterogeneous. Astrocytes have been shown to adopt multiple roles in the process of damage and repair in MS lesions (5). In MS biopsies, demyelinated axons were described as forming unusual membranous communications with nearby astrocytes, which indicates a possible metabolic function (31). When astrocytes were depleted in experimental autoimmune encephalomyelitis (EAE), an animal MS model, an increase in immune cell infiltrates and exacerbation of clinical signs were observed (32). Thus, astrocytes clearly have the potential to also exert beneficial effects in CNS inflammation, which is in line with our finding that BMS astrocytes protect from inflammatory neurite damage. Our previous work in embryonal stem cell–derived neurons revealed that TNF-α/IL-17A combination treatment led to neurite disturbances, while single cytokine treatments did not (16). Here, we found that similar TNF-α/IL-17A–induced neurite damage in MS patient iPSC–derived neurons occurred and that the effect was independent of the disease course phenotype. The deletion of *IL-17RA* protected MS-iPSC–derived neurons from TNF-α/IL-17A–induced neurite damage. This finding is in line with reports from others who reported IL-17A–mediated neuronal damage in human iPSC–derived neuron cultures from Parkinson’s disease patients (33). In both experimental setups the activation of nuclear factor-κB–mediated (NF-κB–mediated) pathways in neurons was identified as a key pathway of neuronal damage processes.

Interestingly, type I interferon–dependent genes were upregulated in BMS astrocyte–cocultured neurons, which might be interpreted as a neuronal resilience program (34). This contrasts with the PMS astrocyte/NGN2-neuron cocultures in which a neurodegenerative phenotype in NGN2-neurons was prevalent with genes associated with primary neurodegenerative diseases, such as Parkinson’s disease or amyotrophic lateral sclerosis (35). These pathways typically involve genes of the ubiquitin-proteasome system as a proapoptotic program, which is a plausible player in the observed neurite damage with accumulation of non-phosphorylated neurofilaments. A reproducibility asset but also a limitation in our study is the use of the healthy control–derived NGN2-neurons as a reference population in the patient-derived astrocyte coculture assays. We cannot rule out the possibility that patient-derived neuronal properties may have exerted an additional role in disease phenotype, though we have no clear indication of such an effect from the data of cytokine-treated neuronal monocultures. We observed variation in neuronal monocultures, for instance in cytokine receptor expression (data not shown). However, these findings were not consistent for one or the other patient group. In the future, transgenic NGN2 from all iPSC lines used in this study could be adapted to obtain more homogeneous neuronal populations. Another confounding factor is the use of 1% FCS for the astrocyte differentiation. This technique has been identified as a potential trigger for astrocyte reactivity (36). However, we found no evidence for reactivity in our astrocytes, and notably, no FCS was used during coculture experiments.

The analysis of potential upstream mediators of the protection from TNF-α/IL-17A neurite damage identified a distinct regulation of the JAK/STAT pathway, which is a conserved pathway acting as a central communicator to translate extracellular signals, mainly cytokines, into various downstream effects including inflammation, tissue repair, or apoptosis. In recent years, more and more evidence has accumulated demonstrating that a dysregulated signaling cascade is also profoundly acting on autoimmune disease. The inhibition of the JAK/STAT pathway has been used to prevent gene expression regulated by disease-associated proinflammatory cytokines, and several JAK-blocking drugs have been approved, which are used for the treatment of rheumatoid arthritis (RA) or psoriatic arthritis (37). Our results from the astrocyte transcriptome analysis identified that JAK1 and JAK3 expression were enhanced in BMS; contrastingly, STAT6 and SOCS1 were enhanced in coculture-derived PMS astrocytes, which is suggestive of a distinct JAK/STAT–related activation between MS astrocyte phenotypes. Application of tofacitinib, a drug approved for treatment of RA and inflammatory bowel disease (IBD), on TNF-α/IL-17A–triggered BMS astrocyte/NGN2-neuron cocultures resulted in the loss of BMS astrocytes’ ability to protect neurons against the TNF-α/IL-17A–induced neurite damage. Thus, a sustained activation of the JAK/STAT signaling in BMS astrocytes seems to be involved in the observed BMS astrocyte–mediated neuroprotection. Interestingly, a case report suggested iatrogenic demyelination in a patient to whom tofacitinib was prescribed (38).

Similarly, blocking a proinflammatory cytokine in one disease can have opposing effects in another. For instance, inhibition of TNF-α is an efficient treatment target in several autoimmune diseases, including IBD (39) and RA (40). However, in MS patients the results were deleterious (41). As a matter of fact, MS appeared to be activated or elicited de novo in RA and IBD patients (42). Thus, cytokine downstream effects critically depend on the organ and the cell types in autoimmune diseases. TNF-α can exert immunomodulatory effects in the CNS, which might also be commensurate with our findings of TNF-α involvement in the stimulation of neuroprotective astrocytes. The matter is less clear for IL-17A, which is used as treatment target for psoriasis (43) and ankylosing spondylitis (44). IL-17A signaling inhibition has not yet convincingly been shown to be efficient or detrimental in MS. This state of affairs is even more intriguing since studies in animal models and in humans have suggested prime pathogenic roles for both of these cytokines (45–48).

Both IL-17A and TNF-α use TNFR-associated factor (TRAF) signaling pathways that synergize upon activation of inflammation-related genes via CCAAT/enhancer binding protein (C/EBP) (49). Another potential synergistic mechanism was the observation that IL-17A post-transcriptionally stabilized TNF-α–induced NF-κB signaling by increasing the half-life of the mRNA and ultimately promoting enhanced gene expression (50). We found that these cytokines in combination have both a neurite-damaging potential and can induce neuroprotective programs in astrocytes of BMS patients. This state of affairs could explain why blocking these mediators in MS patients has not been helpful. A recent study in Huntington’s disease patients showed that reactive astrocytes activated via the JAK2/STAT3 pathway were able to reduce the toxic huntingtin protein aggregation in neurons (51). While IL-17A is not known to be the classical interleukin to activate JAK/STAT, there is evidence of an IL-17–JAK/STAT–VEGF axis in reactive astrocytes (52). In our transcriptome analysis, we observed major differences between the 2 astrocyte phenotypes, shown by our principal component analysis, the number of DEGs, and their expression profiles. BMS astrocytes may also have a higher neuroprotective commitment, as the transcriptome of resting BMS astrocytes revealed several activated pro-neuronal pathways, which were not present in PMS astrocytes. Interestingly, while we do not have evidence for a complete dysfunctional JAK/STAT signaling in PMS astrocytes, the IPA upstream analysis of unstimulated astrocytes revealed several JAK/STAT activating factors, including EPO, BDNF, and IL-9, in homeostatic benign astrocytes. This observation as well as the JAK/STAT activation of neurons in the scRNA-Seq analysis indicates a bidirectional communication of astrocytes and neurons via the JAK/STAT cascade in homeostasis and upon inflammatory stimuli.

The differentially expressed factors of BMS and PMS astrocytes were LIF, BDNF, and TGF-β1. Production of LIF and TGF-β1 was inhibited by tofacitinib, while BDNF production was independent of JAK inhibition and rather increased, which could, however, not prevent the neurodegenerative phenotype. LIF belongs to the IL-6–like cytokine family and has been shown to exert axon-protective effects in diverse injury models in vivo (53). Originally, LIF has been described not only as a mediator leading to differentiation in murine leukemia cells, but also as a hematopoietic stem cell–stabilizing factor (54), since its upregulation has been found in diverse models of stress, such as in bone marrow irradiation, promoting stem cell survival (55), and in spinal cord injury, promoting regeneration. The latter indicates a general role also in states of regeneration (56). LIF induction in astrocytes has itself been attributed to TNFR2 signaling and resulted in mediation of a promyelinating phenotype in oligodendrocyte precursor cells (57). LIF signals through a heterodimer receptor (gp130 and LIFRb) of the IL-6 family group, which is downstream relying on JAK/STAT signaling (58) and mediates neurite protection and growth promotion in mouse retinal ganglion cells (59). As a result, LIF is important for the maintenance of distal axons (60). Furthermore, studies of forced LIF induction in EAE support an immunomodulatory and beneficial effect of this cytokine in CNS inflammation (61).

As a further BMS astrocyte–derived soluble factor, we identified TGF-β1, which is both a growth factor and an immunomodulatory cytokine with independent relevance for cells in both the immune system and the CNS. While TGF-β1 is not detectable in the healthy adult brain, it has been described in several CNS pathologies, including ischemia, MS/EAE, Alzheimer’s disease, and others (62). In MS lesions, production of TGF-β proteins has been attributed to both glial cells and invading peripheral immune cells (63). Blocking TGF-β1 in EAE led to disease exacerbation (62). TGF-β has been described as an essential factor in the context of induced Treg generation (11); however, it is also involved in the IL-23/IL-17A axis and thus in the generation of encephalitogenic T cells of the Th17 phenotype (64). Thus, TGF-β1 has a positive effect on cell proliferation and differentiation, but the outcome is highly dependent on the costimuli. TGF-β1 was absent in the supernatant of treated HC and PMS cocultures, which indicates a crucial role of this factor for the observed neuroprotective effect.

In general, our data support the idea of beneficial immunity. In particular, the crosstalk between immune cells (producing TNF-α/IL-17A) and CNS cells has been previously discussed as a potentially beneficial mediator that results in downmodulation of an inflammatory response and activation of recovery programs (65). Due to the laborious procedure of iPSC generation and neural cell differentiation, such a study is only possible with rather low sample sizes. Therefore, confirmation of these data in independent samples seems appropriate. Nevertheless, our work not only shows that CNS-endogenous responses to inflammatory stimuli explain the positive outcome of chronic inflammatory disease in some patients, but also highlights the potential to modulate these CNS-endogenous pathways in patients with an unfavorable disease course.

## Methods

### Reprogramming and characterization of human iPSCs.

For this study, 3 MS patients for the “benign” group were enrolled, all with relapsing-remitting MS (female, White). For the “progressive/disabling” group, 2 patients diagnosed with primary progressive MS (female, White) and 1 with secondary progressive MS (female, White) were included (Table 1). All patients were recruited and sampled at the MS outpatient clinic of the Charité – Universitätsmedizin Berlin. Peripheral blood mononuclear cells were isolated with BD Vacutainer CPT blood collection tubes according to the manufacturer’s protocol and frozen at –80°C until further use. One HC person (BIH238-A; female, White) was recruited in parallel to the patient recruitment, and 2 further HC iPSC lines (BIHi242-A, BIHi250-A) were provided by the Technology Platform Pluripotent Stem Cells at the Max Delbrück Center for Molecular Medicine in the Helmholtz Association, Berlin (MDC). By the time of sample harvesting, all patients had not been under immunosuppressive treatment for the past 6 months (e.g., natalizumab, mitoxantrone, rituximab, dimethyl fumarate, fingolimod). MRI had been acquired in clinical routine in the Charité Neuroradiology Department and/or in different private practices and was provided by the study participants for evaluation in this study. Clinical T2-weighted 2D sequences were used to count hyperintense brain lesions by 2 expert MRI technicians (blinded to the purposes of this study) with more than 10 years of MS research experience. The ventricle width was identified in 2D scans for tissue loss (66).

Reprogramming of iPSCs was done by the Technology Platform Pluripotent Stem Cells at the MDC, as described previously (67). All lines were characterized in terms of undifferentiated phenotype, normal karyotype, line identity by short tandem repeat analysis, sterility (bacteria/yeast/fungi/mycoplasma), and absence of the reprogramming vector. The certificate of analysis can be found at the Human Pluripotent Stem Cell Registry (https://hpscreg.eu) by reference to the BIH sample name.

### Differentiation to neural stem cells, neurons, and astrocytes.

iPSCs were differentiated to neural stem cells (NSCs) according to an adjusted protocol described by others (13). In brief, iPSCs were seeded in mTesR plus (Stemcell Technologies) containing 10 μM ROCK inhibitor Y-27632 (Stemcell Technologies) to a density between 200,000 and 400,000 cells in Geltrex-coated (30 μg/cm^2^; Thermo Fisher Scientific) 6-well plates (Corning). At day 3, medium was changed to Neural Induction Medium: DMEM/F12 (Thermo Fisher Scientific) containing 10 μM SB-431542 (Stemcell Technologies), 10 μM dorsomorphin (Stemcell Technologies), 1% B27, 2% N2 (both from Thermo Fisher Scientific). Medium was changed daily, and cells were split on day 8 on a Geltrex-coated 6-well plate. After splitting, cells were cultivated in Neural Expansion Medium (NEM) consisting of Neurobasal Medium/Advanced DMEM (1:1), 2% Neural Induction Supplement, and 1% penicillin/streptomycin (P/S) (all from Thermo Fisher Scientific) at different cell densities. NSCs were fed every other day, expanded, and split until passage 4. Cells were characterized by immunofluorescence for expression of NSC markers Sox1, Sox2, Pax6, and Nestin (all antibodies from Human Neural Stem Cell Immunocytochemistry Kit, A24354, Invitrogen). Cell banks in this passage were cryopreserved.

For subsequent neuronal differentiation, NSCs were thawed in NEM supplemented with 10 μM ROCK inhibitor Y-27632 and plated on Geltrex-coated 24-well plates. To start differentiation, medium was switched to Neuronal Differentiation Medium 1 (NDM) containing DMEM/F12, 1% B27, 1% P/S, 10 ng/mL NT-3 (PeproTech), and 10 ng/mL BDNF (PeproTech). After 1 week, cells were detached with Accutase (BioLegend), strained through a 70 μm cell filter (Corning), and seeded on plates coated with poly-l-laminin (5.6 μg/cm^2^ poly-l-ornithine from Sigma-Aldrich, 2.8 μg/cm^2^ laminin from R&D Systems). At day 10, medium was changed to Neurobasal Medium (Thermo Fisher Scientific) supplemented with 1% B27 and 1% P/S, and cells were cultured for 2 more weeks.

For astrocyte differentiation, NSCs were thawed and cultured until confluence in NEM. Medium was switched to astrocyte differentiation medium containing DMEM (Thermo Fisher Scientific), 1% Glutamax (Thermo Fisher Scientific), 1% P/S, 1% FCS (Sigma-Aldrich), 2% N2 (Thermo Fisher Scientific), and 20 ng/mL CNTF (Miltenyi Biotec).Medium was changed every 3 days, and cells were passaged when they reached confluence and differentiated for 6 weeks in total. For coculture experiments, FCS was withdrawn from the medium for at least 3 days.

### Coculture experiments.

For coculture experiments, an inducible iPSC line (derived from healthy donor BIHi005-A-24; provided by the Technology Platform Pluripotent Stem Cells at the MDC) overexpressing NGN2 was used as described by others (18, 19). Cells were seeded at a density of 50,000 per well of 24-well plates and cultivated in mTesR supplemented with 10 μM ROCK inhibitor Y-27632. The next day mTesR medium was replaced by Neuronal Induction Medium 2 containing DMEM/F12, 2% N2, 10 ng/mL BDNF, 10 ng/mL NT-3, 1% Non-Essential Amino Acids Solution (Thermo Fisher Scientific), and 10 μM doxycycline (Sigma-Aldrich). On day 2, puromycin (0.8 μg/mL; Thermo Fisher Scientific) was added for negative selection. On day 5, medium was changed to Neuronal Medium (NM) containing Neurobasal Medium, 1% B27, 10 ng/mL BDNF, 10 ng/mL NT-3, 1% Glutamax. Fifty micromolar cytosine arabinoside (Ara-C, Selleckchem) was used for 24 hours to remove remaining stem cells. On day 7, iPSC-derived astrocytes were seeded on top of the neurons and allowed to settle for 2 more days. Notably, no FCS was present in the coculture medium. Medium was replaced with NM without BDNF and NT-3, and for experiments, cultures were incubated with the cytokines TNF-α (PeproTech), IL-17A (PeproTech), or IL-10 (PeproTech) or with a combination of TNF-α/IL-17A (each 50 ng/mL) for 24 hours. For tofacitinib (Santa Cruz Biotechnology) experiments, cultures were treated with either 100 nm or 1,000 nm tofacitinib or IL-13/IL-21 (each 50 ng/mL; PeproTech) for 24 hours, followed by incubation with TNF-α/IL-17A (each 50 ng/mL) for another 24 hours.

### Immunofluorescence staining.

For immunofluorescence stainings, cells were fixed with 4% paraformaldehyde (Santa Cruz Biotechnology) for 10 minutes at room temperature (RT), washed with PBS, and permeabilized with 0.1% Triton X-100 (Sigma-Aldrich) for 5 minutes. Cells were washed twice and incubated with primary antibodies against Tubb3 (1:250; clone TUJ1, 801201, BioLegend), MAP2 (1:100; clone A-4, sc-74421, Santa Cruz Biotechnology), IL-17R (1:50; clone G-9, sc-376374, Santa Cruz Biotechnology), TNFR1 (1:100; clone H-5, sc-8436, Santa Cruz Biotechnology), AQP4 (1:100; E-AB-64864, Elabscience), and GFAP (1:250; GA52461, Agilent) for 1 hour at room temperature. Directly labeled antibodies were used for NF-L (clone NFL3, 845908, BioLegend) and NF-H (clone SMI31, 801610, and clone SMI32, 801706, BioLegend) stainings. Information regarding all antibodies can be found in Supplemental Table 4. After 2 washing steps, the matching secondary antibodies goat anti-rabbit AF 488 (A-11008), goat anti-mouse AF 488 (A-11001), goat anti-rabbit AF 594 (A-11012), and goat anti-mouse AF 594 (A-11005) (all diluted 1:1,000; all from Invitrogen) were given for 1 hour, and cells were counterstained with DAPI (1:4,000; 422801, BioLegend); for mounting, DAKO Mounting medium was used. Images were taken on a Leica DMI6000 B fluorescence microscope with LAS X software. IL-17R control staining can be found in Supplemental Figure 7. For coculture experiments, monoclonal antibody SMI31 (binding phosphorylated NF-H) was used as a pan-neurite reference. For monoculture experiments, monoclonal antibody binding microtubule-associated protein 2 (MAP2) was used as reference neurite marker as SMI31 showed only weak immunofluorescence in these assays. Monoclonal antibody SMI32 (binding non-phosphorylated NF-H) was used as a neurite damage marker (17). The resulting SMI32/MAP2 and SMI32/SMI31 ratios were used in the monocultured and cocultured assays, respectively. Analysis of fluorescent images of MAP2, SMI31, or SMI32 area was done with ImageJ (NIH).

### Calcium imaging.

Neurons were seeded on black 96-well plates (Ibidi). Fluo-4AM (1 μM; Thermo Fisher Scientific) was used as a calcium indicator and loaded within Neurobasal Medium without phenol red (Thermo Fisher Scientific) for 15 minutes at 37°C. After 1 wash, cells were equilibrated for another 10 minutes. During microscopy, cells were in an incubation chamber at 37°C, 5% CO_2_, and images were taken with an Olympus CellR microscope using a ×20/0.75 DIC objective with 483/32 BP excitation filter. Stacks of images were recorded with a Hamamatsu ImagEM CCD 9100-13 camera at a rate of 5 Hz for 3 minutes and a 512 × 512 pixel resolution. Activities of single neurons or astrocytes were tracked with ImageJ.

### Single-cell RNA sequencing.

Cocultures of NGN2 and iPSC-derived astrocytes were seeded in a 24-well plate and treated with TNF-α/IL-17A or left untreated (control). All samples were processed on the same day and harvested with Accutase. For cell multiplexing, subsamples were labeled with Cell Multiplexing Oligos (CMO309/CMO310/CMO311/CMO312, 10x Genomics) according to the manufacturer’s recommendation and filtered with a cell strainer (70 μm) to get a single-cell suspension. Labeled cells of 4 subsamples with about 2,500 cells per sample were pooled, each containing a unique CMO, and loaded into a Chromium Next GEM Chip G (PN-1000127, 10x Genomics). The library preparation was done with Chromium Next GEM Single Cell 3′ Reagent Kits v3.1 with feature barcode technology for cell multiplexing protocol. Quality control was done with the Agilent 4200 TapeStation system. The cDNA and the multiplexing library were sequenced on an Illumina NovaSeq6000 instrument (run setting of 28-10-10-90 cycles) to a depth of 30,000 reads per cell.

### Single-cell data analysis.

Using Cell Ranger v6.0, multiplexed sequencing samples (CMO tagged) were first aligned to the human reference genome (hg38) following demultiplexing and statistical analysis in the R-based package Seurat v4.0 (68). Here, doublets were excluded before data were normalized and variance stabilized using sctransform (69). Cells were included only if expressing at least 200 genes, and a gene was included only if at least 3 cells expressed it. Only genes with an adjusted *P* value less than 0.05 qualified as differentially expressed genes (DEGs). Enrichr was used for pathway analysis (70) and IPA (Qiagen) for upstream analysis.

### Bulk RNA sequencing of astrocytes and analysis.

To remove neurons from cocultures, prewarmed PBS without Ca^2+^/Mg^2+^ (Thermo Fisher Scientific) was applied to the wells to wash the cells followed by a solution of 0.5 mM EDTA in PBS without Ca^2+^/Mg^2+^. Plates were incubated for a few minutes at 37°C and then observed under the microscope while gently rocking. Detached neurons were then removed, and wells were washed again with PBS without Ca^2+^/Mg^2+^. For RNA isolation, cells of 6 technical repeats were lysed in RNA isolation buffer containing 1% 2-mercaptoethanol (Thermo Fisher Scientific) and isolated with Quick-RNA MicroPrep according to the manufacturer’s protocol (Zymo Research). All samples were checked for RNA integrity number values greater than 9, and mRNA sequencing library was done according to protocol (Illumina). Libraries were sequenced on a NovaSeq S1 flowcell, SR100 mode, with 30 million reads per sample. Raw Fastq data were aligned against the human reference transcriptome hg38 using Salmon (71), and data analysis was performed with the R software package limma (72). For DEGs, only genes with 2-fold differences and below an adjusted *P* value of 0.01 were included. For pathway and Gene Ontology (GO) analysis, the web database Enrichr was used (70).

### Supernatant analysis.

Supernatants of control (untreated) and TNF-α/IL-17A–treated cocultures were collected and stored at –80°C. For CTGF analysis, a Sandwich ABTS ELISA was used according to protocol (PeproTech). Analysis of all other factors was performed according to protocol with bead-based multiplex assay panels using LEGENDplex Human Neuroinflammation Panel, T Helper Cytokine Panel, and Hematopoietic Stem Cell Panel for LIF (BioLegend). Flow cytometry measurement was performed on a BD FACSCanto or LSR Fortessa. FCS files were analyzed with LEGENDplex data analysis software (BioLegend).

### Data availability.

The sequencing data that support the findings of this study are available on request from the corresponding author. The data are not publicly available because they contain information that could compromise participant consent.

### Statistics.

GraphPad Prism 9 was used for statistical testing. Data were tested for normal distribution. Outlier tests were performed using the robust regression and outlier removal (ROUT) method. Kruskal-Wallis test (Figure 5, Figure 2C, Figure 3, C–E, Figure 6, B–D, and Figure 7A), Mann-Whitney test (Figure 7B), or unpaired 2-tailed *t* test (Figure 5E) was used to test for statistical significance, defined as *P < 0.05, **P < 0.01, ***P < 0.001, ****P < 0.0001.

### Study approval.

Approval was given by the Charité’s Ethics Committee of Charité – Universitätsmedizin Berlin (EA1/023/15), and all subjects gave their written informed consent.

## Author contributions

VS and JK designed the research project and the experiments. TC advised on, supported, and performed transcriptomics experiments in the core facility for genomics. SD advised on, supported, and performed iPSC generation in the core facility for stem cells. MA, JK, JS, BM, CB, and KSR performed experiments. MA and KSR supported analysis of the sequencing data. DZ performed statistical analyses. FP advised on study design and data analysis, and supported patient recruitment. VS and JK wrote the manuscript. CC supported MRI analysis and revision of the manuscript.

## Supplementary Material

Supplemental data

Supporting data values

## Figures and Tables

**Table 1:**
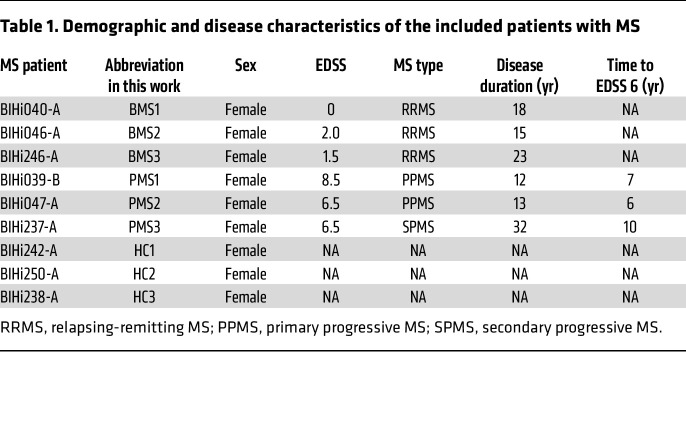
Demographic and disease characteristics of the included patients with MS

**Table 2:**
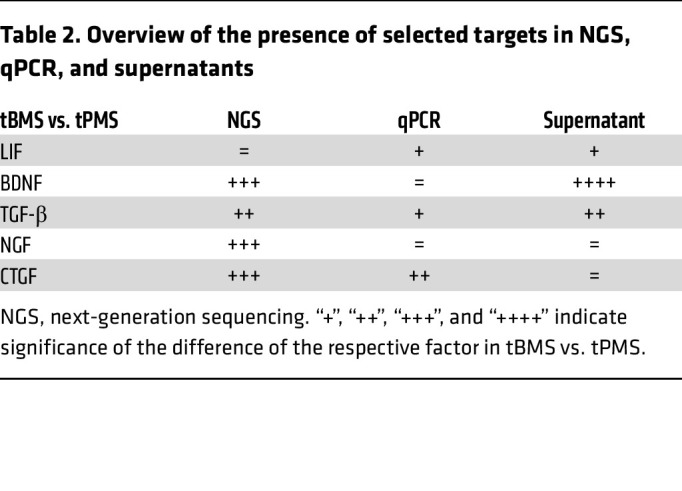
Overview of the presence of selected targets in NGS, qPCR, and supernatants

## Version 1 05/23/2023

In-Press Preview

## Version 2 07/03/2023

Electronic publication
