# Supplementary material for: iPSC-derived reactive astrocytes from patients with multiple sclerosis protect cocultured neurons in inflammatory conditions
Source: J Clin Invest. 2023 Jul 3;133(13):e164637. doi: 10.1172/JCI164637 (PMC10313373; doi:10.1172/JCI164637)
Supplement: Supplemental data [file jci-133-164637-s072.pdf]

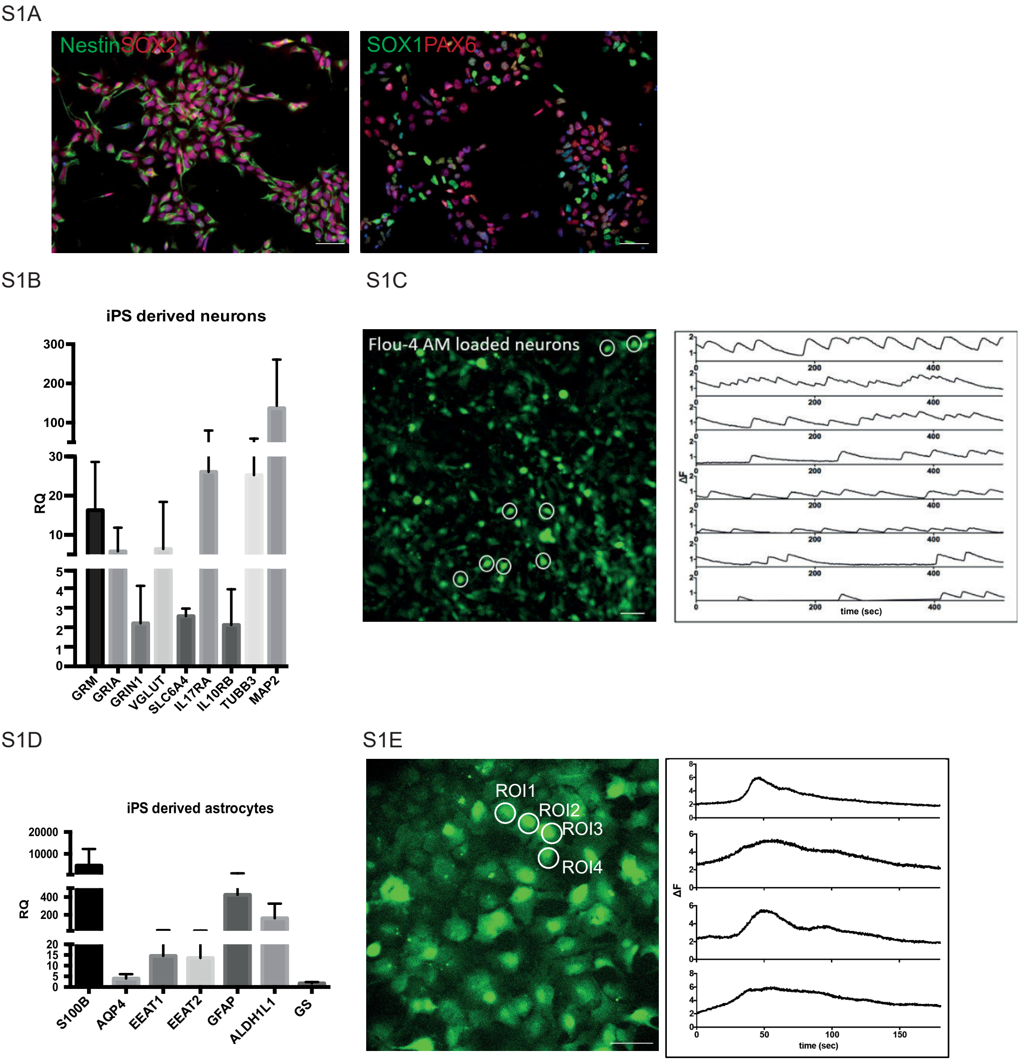

Figure S1 (A) Characterization confirms successful iPSC differentiation into NSC. Immunofluorescence staining of neural stem cell marker Nestin, Sox2, Sox1 and Pax6 of NSCs in p4. Scale bar: 50 µm. **(B) Rt-qPCR of differentiated iPSC-derived neurons presenting a heterogenous neuronal phenotype.** Bar graph shows the mean RQ + SD of two different lines (BMS1; PMS2) of three independent experiments each. GAPDH and actin were used as housekeeping genes. **(C) Ca^{2+} imaging show spontaneously active iPSC-derived neurons.** iPSC-derived neurons were loaded with 1 µM Fluo-4 AM for 15 min at 37 °C. Imaging was done within an incubation chamber at 37 °C, 5% CO_2 and observed with an Olympus Cell^R microscope. Stacks of images were recorded at rate of 5 Hz for 3 minute and activities of single neurons were tracked with ImageJ. **(D) Rt-qPCR of differentiated iPSC-derived astrocytes confirm a mature phenotype.** Bar graph shows the mean RQ + SD of all six lines. GAPDH and actin were used as housekeeping genes. **(E) Ca^{2+} imaging show spontaneously active iPSC-derived astrocytes.** iPSC-derived astrocytes were loaded with 1 µM Fluo-4 AM for 15 min at 37 °C. Imaging was done within an incubation chamber at 37 °C, 5% CO_2 and observed with an Olympus Cell^R microscope. Stacks of images were recorded at rate of 5 Hz for 3 minute and activities of single astrocytes were tracked with ImageJ.

S2A

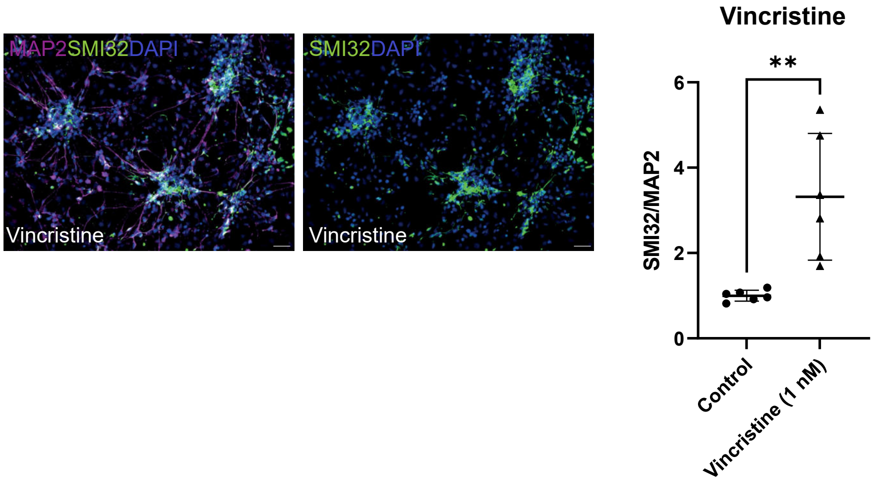

S2B

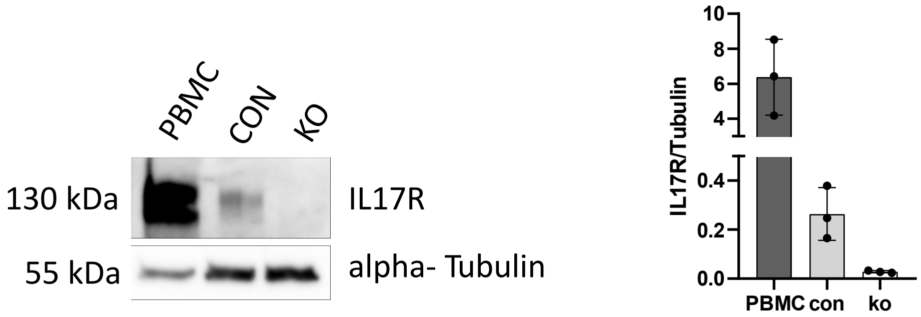

Figure S2 (A) Evaluation of SMI32 as neuroaxonal marker. iPSC-derived neurons were treated with 1 nM vincristine for 24h and stained against SMI32/MAP2. Neurons showed significantly increased in SMI32/MAP2 ratios and confirmed SMI32 as suitable neuroaxonal marker. **(B) Western Blot confirmed knock-out of IL-17R in PMS1**. PBMCs were used as positive control and iPSC without knock-out as negative control for the IL17R-knockout line. All lines were normalized to α-tubulin and bar graph shows the mean + SD ratio of IL-17R/α-tubulin of three independent experiments.

S3

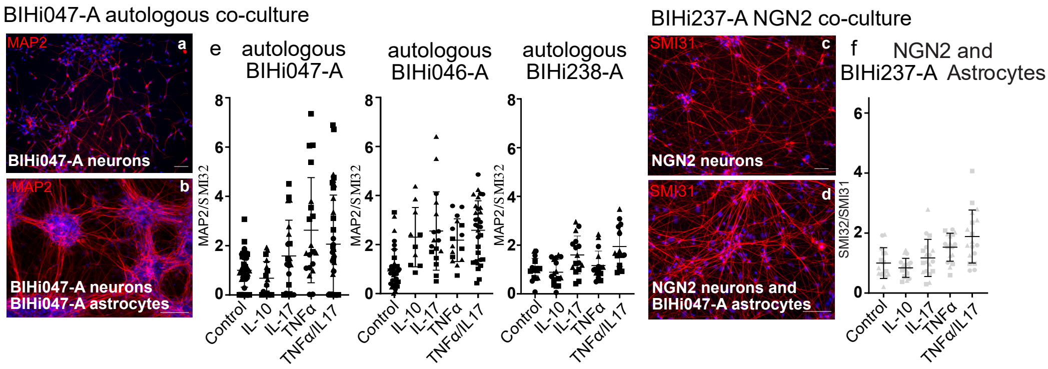

Figure 3 Comparison of autologous and NGN-2 co-cultures (a) iPS-derived neurons and (c) NGN2-neurons show similar morphology with a trend to more interconnectivity in the NGN2-neurons. (b) Autologous co-cultures show clustering of neurons, whereas in (d) NGN2-cocultures there are more branching and interconnecting processes. (e) SMI32 analysis in co-cultures show higher variability than SMI32 analysis in (f) NGN2 co-cultures. Scale bar: 50 µm. Disclosure: Fig. f also appears in Fig. 3D (PMS3).

S4

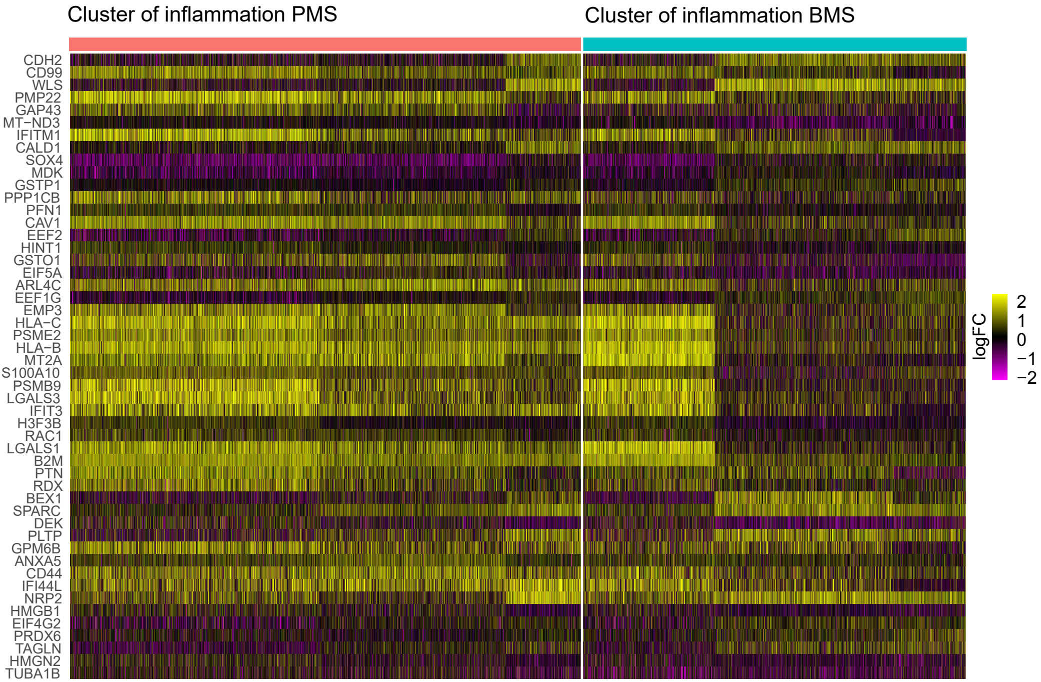

Figure S4 Heatmap of the top 50 DEG (ranked by adjusted p-value) in the cluster of inflammation. Single-cell RNAseq data, comparing treated PMS and BMS samples, each line represents a single cell.

S5A

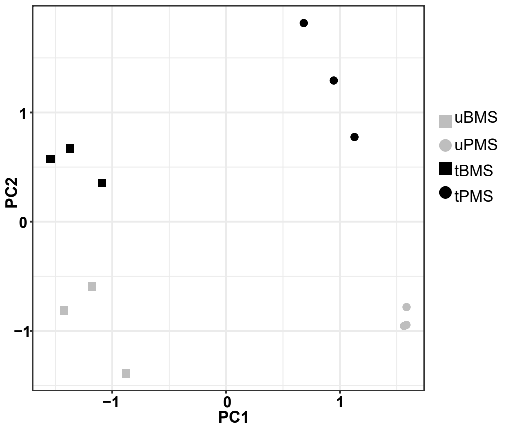

S5B

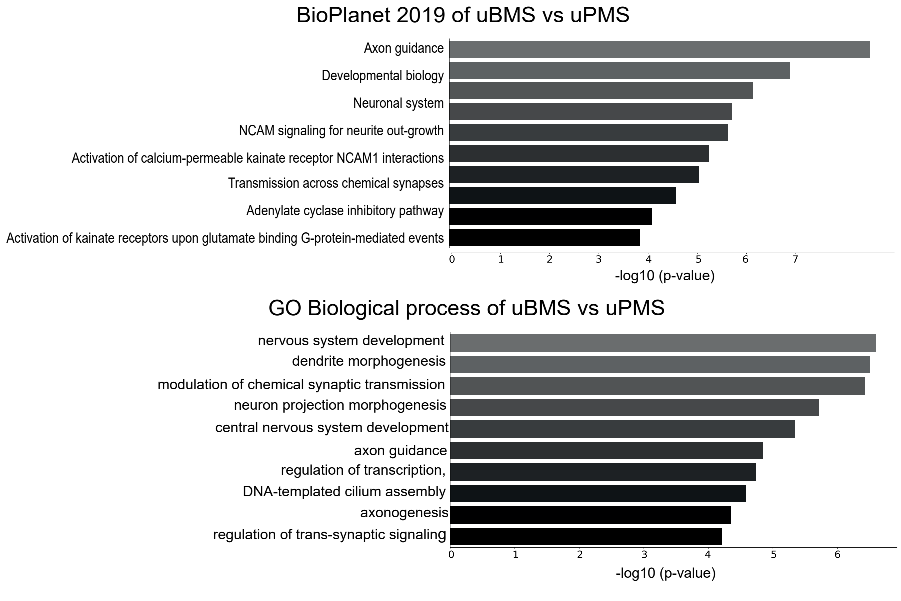

Figure S5 (A) PCA plot of bulk RNAseq of mono-culture derived astrocytes show distinct segregation. Samples were segregated according to patient group (PC1) and according to cytokine treatment vs control (PC2) **(B) Enrichr analysis of untreated BMS vs. PMS show activation of neuroprotective features.** BioPlanet 2019 pathway analysis showed activation of pathways related to axon guidance in BMS and GOs of biological processes were found in nervous system development.

S6

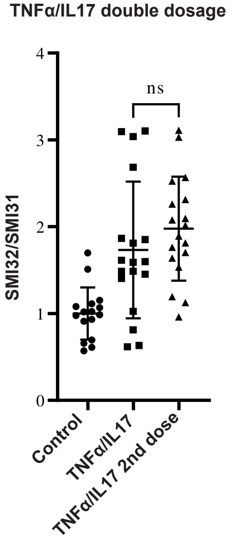

Fig S6 TNFα/IL17 double dosage. NGN2 monocultures neurons were treated with TNFα/IL17 (50ng each) for 24h. The next day neurons received a second dose of 20 ng TNFα and 8 ng of IL17 for 24h (measured concentrations in supernatants). No significant difference were seen between single and double-dosage treatment of TNFα/IL17. Each data point represents a microscopic field of view (641 x 479 µm); pooled data from three independent experiments. Statistical signifance was test with a Kruskal-Wallis test.

S7

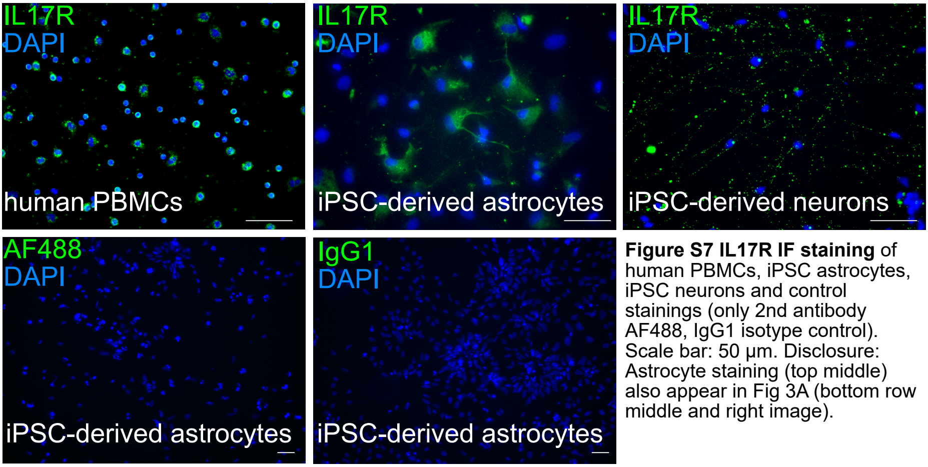

Figure S7 IL17R IF staining of human PBMCs, iPSC astrocytes, iPSC neurons and control stainings (only 2nd antibody AF488, IgG1 isotype control). Scale bar: 50 µm. Disclosure: Astrocyte staining (top middle) also appear in Fig 3A (bottom row middle and right image).

Tofacitinib treatment on NGN2 monoculture

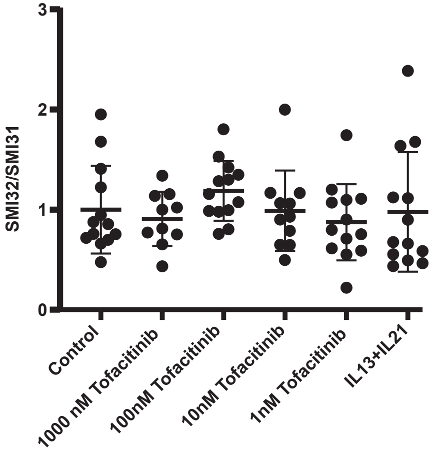

Figure S8 Tofacitinib treatment of NGN2 monocultures. Immunofluorescently stained SMI32/SMI31 NGN2 neurons were analysed with Imaris and presented as surface ratio of SMI32/SMI31+ SD and normalized to the control. Each data point represents a microscopic field of view (641 x 479 µm); pooled data from three independent experiments. No differences were seen after Tofacitinib treatment or IL-13 + IL-21 (50g/ml each) after 24h.

Figure S9

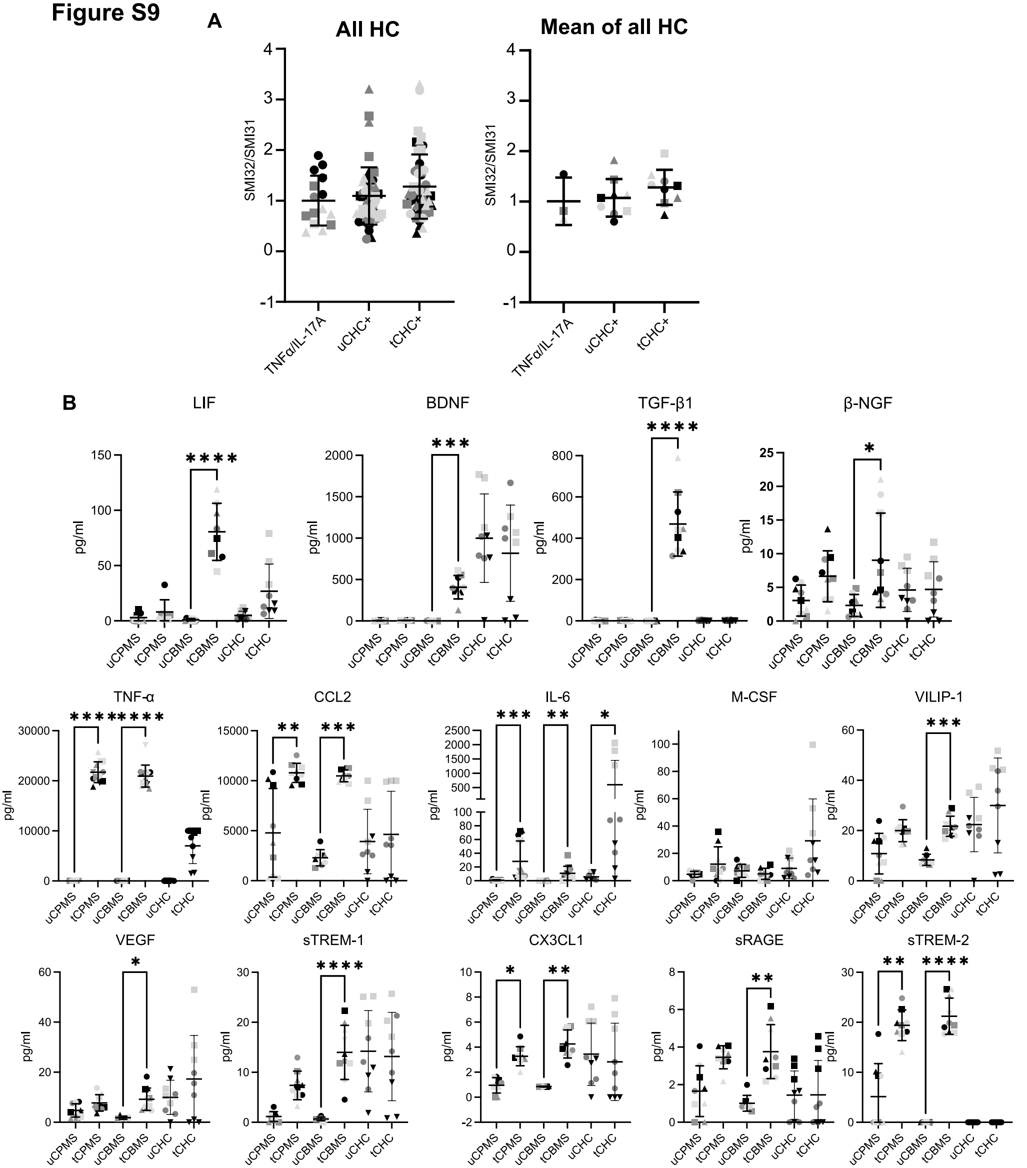

Figure S9.Supernatant analysis of healthy control astrocytes with NGN2 neuron co-cultures (A) NGN2 neurons were treated with TNFα/IL17A (50 ng/ml) for 24h (represented with "+") and medium was replaced with medium control, untreated- or treated co-culture supernatants from HC1-3. SMI32/SMI31 does not show significant reduction after treatement with HC supernatants (i.e. no reduction in neurite damage).

(B)Bead-based multiplex assay analysis of supernatants of HC1-3 (uCHC - untreated co-culture of HC astrocytes / NGN2 neurons; tCHC TNFα/IL17A treated co-cultures of HC astrocytes / NGN2 neurons) in comparison with MS supernatants (see Fig. 6). Each data point represents one sample of collectively three independent experiments. HC and MS supernatants have been measured in different experiments, therefore a batch effect between HC and MS cannot be ruled out. Statistical testing was only performed between treated/ untreated samples of the same group. Similar symbols are used for the individual person's derived cultures.

Statistical significance was tested with Kruskal Wallis test. *p<0.05,***p<0.001, ****p<0.0001; (IL-17A, IL-21, CTGF were not analysed).

Table S1 - Cluster defining genes

Cluster 1 - Cytoskeleton		Cluster 2 - Cellular response to stimuli		Cluster 3 - Cell proliferation		Cluster 4 - Nervous system development		Cluster 5 - Cellular stress		Cluster 6 - Inflammation	
**Gene**	**avg_log2FC**	**Gene**	**avg_log2FC**	**Gene**	**avg_log2FC**	**Gene**	**avg_log2FC**	**Gene**	**avg_log2FC**	**Gene**	**avg_log2FC**
ANKRD1	2.99562337593789	SFRP2.2	1.58886762338952	POU5F1.2	2.49369832074484	STMN2.4	4.18610761023128	MTRNR2L12.4	3.29429378923887	CCL2	3.06776112161808
NPPB	2.99279134413432	CENPF.2	1.42860900536341	L1TD1.1	2.34969396836053	NEFM.4	4.01533936896675	MT-ND5.4	2.39006091713935	IFITM3	3.01381442252737
TAGLN	2.95905126606757	HIST1H4C.2	1.39837507716592	MIR302CHG	2.03185880214932	NNAT.3	2.62156479234601	MT-ND1.2	2.37861761933319	IFITM2	2.79824749487566
SPP1.1	2.71317327186975	SFRP1.3	1.23728563212321	ESRG	1.76644597095024	NEFL.4	2.59283359632819	MT-CO3.2	2.37639824530724	LGALS3	2.53666232179441
MYL9.1	2.66157917456433	ID3	1.18252360092235	KRT18.2	1.53439289934371	TAGLN3.4	2.57104930692366	MT-ATP6.2	2.35417899285942	B2M	2.43902296247652
KRT8.1	2.44948751876578	CENPV.1	1.15850142796714	APOE.2	1.51881137941897	SNCG	2.52080182565187	MT-CO1.3	2.28674459458102	HLA-B	2.36037377317869
TPM2.1	2.39459916130752	PRTG.3	1.15450209933058	MT1X.2	1.46779228157795	DLL3.4	2.38443881346868	MT-ND4.2	2.2592037317731	LGALS1	2.29085733212529
CCN2.1	2.38388233647131	HMGB2.2	1.13531350111473	POLR3G.1	1.36786153511046	RTN1	2.29030316976346	MT-CYB.4	2.23050250234325	MT2A	2.27897679592715
TFPI2	1.96585998475325	TYMS.2	1.07675148311633	AL353747.4	1.32968838142721	TUBB3.4	2.10039836904485	MT-ND2.2	2.17939297876506	PMP22	2.17544732451816
CALD1.1	1.93859249048871	NASP.2	1.05573586884947	TERF1.2	1.31116039289613	INA.4	2.06159227912912	MT-ND3.3	2.1322714799426	ISG15	2.16029405685387
TPM1.1	1.81067154421517	LIX1.2	1.03813964654856	AC009446.1	1.29889031324115	DCX	1.99317778233357	MT-CO2.3	2.11025143724826	IFITM1	2.11079344813938
S100A11.1	1.6890212396439	FGFBP3.3	1.03502572018651	DPPA4.1	1.22992595019036	SYT1.4	1.90431432097517	MT-ND6.3	1.94441916179958	PTN	2.10556182358999
CCN1	1.57175282722279	TMSB15A.1	1.02245653179425	SCGB3A2	1.20669601837761	STMN1.3	1.83929557702463	MT-ND4L.3	1.91567917714557	COL1A1	2.05366626113747
FN1.1	1.56064460848647	DEK.2	1.01995687302309	CD9	1.18012422076971	ONECUT2	1.82464206482359	RPS14.1	-0.708320659050041	HLA-C	2.02889813766319
AKAP12.1	1.50734324493451	HMGN2.1	1.01738332990073	DNMT3B	1.08710161331003	MLLT11.3	1.80063635080776	RPS12.2	-0.797201251941915	CHCHD2	1.83833847432587
ANXA1.1	1.49133076450917	RANBP1.3	1.00680211215766	CD24.2	1.08226298511262	GNG3	1.7557324000543	RPLP1.2	-0.931239159870216	IGFBP5	1.79433412567084
CNN1	1.446037118695	H2AFZ.3	1.00184894631844	SPINT2.1	1.05921705654116	SCG3.3	1.7122212287974	RPS8	-0.706400656604484	TSPO	1.71361870732305
TCIM	1.41551544793318	IGDCC3.3	0.99753300341348	TSTD1.2	1.05419537438699	CFAP298.4	1.6946661523928	RPL10	-0.747793943266299	FTH1	1.70467509224871
NEXN.1	1.30457814949246	ANP32B.2	0.974287326073584	EPCAM	1.05399818107423	KIF5C.4	1.69309918975006	RPS23.3	-0.728265278078979	GPM6B	1.69009713114683
ACTA2.1	1.29296203974367	CKS1B.3	0.944528982372129	DMKN	1.04248677960659	TUBA1A.2	1.66707803674334	RPL26.1	-0.667954721399846	IL32	1.68765806457118
CCND2.1	1.21006580771053	FST.3	0.92549524034092	UGP2.1	1.03558527123187	ELAVL3.4	1.60413515728384	RPL7A	-0.694042851562104	CAV1	1.6632267859025
TAGLN2.1	1.20562616191164	TOP2A.2	0.915471468312626	SEMA6A.2	1.03523623495586	NOVA1.4	1.57285121129487	RPS27A.2	-0.684757173126698	S100B	1.59475335339091
ACTN1.1	1.16772794496255	PCLAF.1	0.898647287186314	LIN28A.1	1.02648505012482	NCAM1.4	1.51871987211462	RPS18.2	-0.679773026899294	A2M	1.55802913811221
MYH9.1	1.1495845422664	HMGB1.2	0.89003265208428	FXYD5	0.978351228881327	MAP1B.3	1.48198190305208	RPL15.2	-0.60766431737682	IFI6	1.53282492007499
GNG11.1	1.14507181312574	TTYH1.2	0.879362685649359	TDGF1	0.972496432144749	STMN4	1.46349759011104	RPS3A.2	-0.614382246097604	NTRK2	1.45683703361277
ITGB1.1	1.12831962157014	HSPD1.2	0.872836136805233	PMAIP1.1	0.965021213959111	NREP.3	1.40964793301522	RPS7.1	-0.600517702986692	CD74	1.44687182780813
LIMS1	1.12434630048714	HES5	0.869299476705525	HMGA1.2	0.955578172395437	MIAT.4	1.38465923440818	RPL41.1	-0.571386616420793	HLA-DRA	1.43184071042501
NES.1	1.10305373039676	ANP32E.1	0.867948814555515	GRID2	0.951149226159631	CPE.4	1.38028573706402	RPL18	-0.566651439405553	CD99	1.41885770516494
HMGA2	1.07167298520186	MCM7.3	0.864199177855942	CEBPZ.1	0.933329545981479	IGFBPL1.2	1.33996393726286	RPL11.1	-0.596685193906344	ANXA2	1.41049696473184
UPP1.1	1.06093786725598	TUBA1B.2	0.857924871696223	MT1E	0.921859635126644	ELAVL2.3	1.3308541507659	RPL17.3	-0.615302749256879	SERPINA3	1.39528751798254
EDN1	1.03840647679642	NUSAP1.2	0.847901863038761	CLDN6.1	0.896696989478597	ELAVL4	1.31866785012252	RPL30	-0.564269507807606	IFI27	1.36179675737475
RHOB.1	1.0305864626568	HNRNPD.2	0.83883908904449	CFAP298.2	0.889906678714632	CDKN1C.1	1.31096885375195	RPS15A.1	-0.555944606750815	ARL4C	1.35441839676222
TPM4	1.02864110712269	CCNB1.2	0.83472966122162	PTMA.1	0.888870601926702	BASP1.3	1.30766932927539	RPL32	-0.533468616104709	PTX3	1.32479319918795
PALM2-AKAP2.1	1.00935254029093	NCL.2	0.827832073237444	VASH2.1	0.880664074074432	SOX11.2	1.29059669926987	RPS28.1	-0.541922308352028	FABP7	1.28313261039701
CNN2	0.999068695837052	IGF2BP1.2	0.817198342987847	SNRPN.1	0.852167295418091	PPP1R14A.1	1.25830946005182	MT-ATP8.1	1.48932318434429	TIMP1	1.27321857327778
LAMC1	0.987050666322692	PTTG1.2	0.814960468051391	NPM1.1	0.850406168672392	NHLH1	1.25659157495917	RPL5.2	-0.558747539789366	COL4A1	1.26654959170144
ACTB	0.970914340357869	NPM1.2	0.813351305421972	DIAPH2	0.850103192570038	CRMP1.3	1.23613043025647	H3F3A.4	-0.669294585787997	CLU	1.26158965086038
COTL1	0.951144152793064	BIRC5.1	0.809584098851815	RPA3.1	0.837428357675285	RGS10.2	1.23316686557888	RPS6.3	-0.583539280630363	SPARC	1.26139741467498
CDH11	0.947052602381785	CCNB2.2	0.806911723719346	SLIRP.1	0.833398325519093	RBP1.1	1.22224380836026	RPL19.1	-0.543370393979168	VIM	1.25701678819223
MYL12A.1	0.933441970074386	PLAGL1.2	0.802734117475171	TUBA1C.1	0.826358138042796	PPP1R1A.1	1.21584730263354	RPL13.1	-0.44775685777452	ARHGAP29	1.25570515717545
TIMP3	0.927084284329304	HELLS.3	0.791650370985634	C1QBP.1	0.814239365686249	NSG1.1	1.18955397360572	RPL35A.1	-0.525312988381437	TMEM158	1.23150783482639
SLC2A1	0.925305349586243	ODC1.2	0.786811136725729	FABP5	0.805351375496707	PBX1.2	1.18618972675094	ACTB.1	-0.786307407639059	SPARCL1	1.20612189028778
LPP.1	0.921638463496596	TMPO.1	0.782749611671252	SEPHS1.1	0.789279660259644	KLC1.3	1.17521917759715	RPS4X	-0.596751843138279	IRF1	1.17722420321199
ITGAV.1	0.916890074476022	MAD2L1.3	0.778925958721906	USP44.1	0.782902317756058	SOX4.2	1.14972634631597	MTRNR2L8.2	1.65543152664417	IFIT3	1.17702668507257
CSRP2.1	0.899013632994773	DHFR.1	0.773731494390907	LINC00678	0.771568256021946	SRRM4	1.14361094061515	RPL21	-0.495254487157761	TIMP2	1.17405460467024
BCAT1.1	0.890033148548769	GINS2.3	0.770212911121641	AC006329.1	0.769262310451266	MAP2.2	1.11931536468561	RPL28.1	-0.43328236412269	BAALC	1.16780862874644
CDH6.1	0.88967080177246	SRSF3.2	0.764034534160968	FOXH1	0.768575425746033	TMEFF1.1	1.11115825293582	RPS19	-0.508776259219522	NRP2	1.16706005301111
IGFBP3	0.871069838661583	SNRPD1.2	0.76194615803089	PRDX1.1	0.762824010098432	GDAP1L1	1.07969364559455	RPL8.2	-0.518603679289083	HLA-A	1.16321103669935
PDLIM7	0.869021954409393	SMS.2	0.758513863802683	AC005062.1	0.76239827142339	SYT4	1.07381223415907	SUMO2.3	-0.534403459594218	PDLIM4	1.15495618260775
GSTA1	0.854033785600306	HNRNPAB.3	0.757972789564583	JARID2.1	0.761718471677311	USP18	1.06292043153277	RPL12.3	-0.532473575900325	PCSK1N	1.14941935456421
SORBS2.1	0.837234164847588	TPX2.2	0.755172433329618	UQCRH.1	0.759499858363499	ANK2.3	1.05730838119758	NDUFA4.3	-0.539431389637329	PSME1	1.14920893636719
PAPPA	0.81908328229123	CLSPN.2	0.754575814559805	GJA1.2	0.755105248894956	OLFM1	1.03765540558269	RPS2.3	-0.633616346650372	CD44	1.14653539249421
GPC4	0.807297090233727	RRM1.1	0.753716354799167	LITAF.1	0.753667252055028	PKIA.2	1.02408053783495	RPS13.1	-0.49490072388596	PLAU	1.13323803507978
DUSP6.1	0.806581770901488	SRSF7.2	0.751460867010911	HSPE1.1	0.753249031751865	ONECUT3	1.01348717054272	RPL29	-0.457330132660592	CCDC80	1.12380260169963
GLIPR1.1	0.796351982304926	MKI67	0.748385794251571	KRT8.2	0.717632110508372	SEZ6L2.2	1.01282808552488	RPL34	-0.453697166450231	NEAT1	1.12253119879643
FBLN1.1	0.785374643279425	GMNN.3	0.747642526447726	HSPD1.1	0.716496323206784	RGS16.2	1.01065867114512	RPS3.4	-0.534197895840406	CD59	1.106652749437
FLNC	0.784130329122376	ID2.1	0.741533017348906	NASP.1	0.713790004794174	CKB.2	1.00861775043975	RPL23.1	-0.475475195687663	HTRA1	1.1040657878031
SMS.1	0.78284137776345	SRSF2.2	0.738663685916462	HMGB2.1	0.692009913055654	BEX2.3	0.998863073197527	RPS11	-0.467068698278855	EMP3	1.06052782621621
SFRP1.1	0.782046274065864	LMNB1.2	0.738054554340809	CHCHD10.1	0.691915403007266	CCNI.1	0.985141324747552	TPT1.2	-0.477160932635065	PSMB9	1.05650375804176
ARL2BP	0.780842312735132	ASPM.1	0.737175516241217	NQO2	0.69025445642519	CALM2.2	0.963897969774111	RPS24.2	-0.436248616370109	WLS	1.05209258389192
DSTN	0.779377638040986	PRKDC.2	0.733831187910759	SNRPF.1	0.680246100702414	PPP1R17	0.938454296943757	RPL24.2	-0.428204433244534	S100A11	1.03442124727313
FLNB	0.755951403003245	TMEM97.2	0.732737851634638	FKBP4.1	0.672787945436896	ATP1A3	0.938197559706837	FTH1.5	-1.25541407062925	RCAN1	1.03285719426016
IGFBP2.1	0.745583541392746	SET.2	0.731638996133885	UBE2C.1	0.669592271439905	VAMP2.2	0.937415147732534	TMSB10.3	-0.743622728774583	RNF19A	1.03032172776564
PRDX6.1	0.737258216315697	HNRNPA2B1.2	0.727756481201089	PSIP1.1	0.658718962169728	EDIL3.1	0.927205487085964	RPL39	-0.397674083592729	LY6H	1.02483358853326
NECTIN3-AS1	0.735051409833563	NUCKS1.2	0.725838265553317	UBE2S.2	0.657054841607954	PGM2L1.2	0.914673750493608	GNG5.1	-0.53732631101864	NUPR1	1.02181502455709
ACTG1	0.720994152652589	MCM3.2	0.719516866087759	HMGB3.1	0.656062951114301	PSTPIP1.1	0.913711293246161	RPS20.1	-0.472213107491422	IER3	1.02033976712383
CLIC1.1	0.718210014074393	SNRPB.3	0.709267182040858	EIF3K.1	0.6524347967918	MAP6.2	0.913682705621332	RPS25.1	-0.42581851801611	SVIL	1.01858928744413
CALU.1	0.705732440458428	SNRPF.2	0.70698630632904	CYBA.2	0.64845610621257	CDKN2D	0.912083058663886	RPS15.1	-0.298235207985312	NQO1	1.01764602953765
AFP	0.697476962945749	NME1.2	0.705371240625588	CYCS.1	0.646953684597365	GNG4.2	0.904256714783882	RPS17.4	-0.477389798469586	NMB	1.01585488168442
HBEGF	0.669173090852178	HNRNPA1.2	0.703997633743011	HSP90AB1.1	0.640644232034852	MAPT	0.899674644952172	HINT1	-0.441194085011686	IFI44L	1.01416522389254
GPC6	0.667081307977339	ACAT2.2	0.702130549073996	RANBP1.2	0.634988033400065	PCBP4.1	0.899372097075459	RPL7.1	-0.46148985862193	BST2	1.00951615127656
NTM	0.665548263804463	SNRPE.2	0.692477643612807	AK4	0.627387421203119	RUNX1T1	0.884231559037726	RPS5.3	-0.435533657148248	COL1A2	1.00142443223343
MAP3K7CL	0.665212596210078	CKS2.2	0.691543201081704	TIMM50.1	0.62330650697684	ANK3	0.883125664480993	NACA	-0.416588282351006	TAPBP	0.984930924251769
PRTG.1	0.662241294391324	HNRNPM.3	0.687647481156128	NDUFAB1.1	0.616374056586844	RAB3A	0.875619190027469	RPL3	-0.472287895204587	PLAAT4	0.977988469153325
EXT1	0.657004276071547	PPA1.1	0.686629772518487	MT1F	0.612944242602513	CD24.3	0.875565114172856	EEF1A1	-0.540864351421386	TNC	0.976296872993481
PDIA6	0.648875280549746	SFPQ.1	0.680586866870829	PAICS.1	0.610732297292324	ONECUT1	0.86655754527487	STMN2.5	-1.11454424244008	PSME2	0.970797031803058
HACD1	0.646366715395016	CENPH.2	0.677215552355528	FOXD3-AS1	0.608961851728097	NRXN1	0.865457455605464	ATP5MC2.2	-0.426227639547194	LMNA	0.970197765654252
ANXA3	0.640340698855186	HSPE1.2	0.676207769114965	GSTP1.1	0.606025735283337	RUFY3.2	0.850446366038337	RPLP0.4	-0.44511222261132	CD47	0.958331289758757
MYL6	0.611413000258121	LDHB.2	0.672663699843049	NR6A1.1	0.603630779083915	TERF2IP.3	0.841840318009377	RPL18A.2	-0.416156942999654	MSN	0.937974101293622
SEMA3C	0.604994850516655	PCNA.2	0.667577191991827	RAN.1	0.603572766508963	SCG5	0.840772379235595	SERF2.2	-0.394510444631488	COL4A2	0.936056297911873
RNF152	0.599763617477785	SERBP1.2	0.662345324220661	PDGFA	0.598272031964522	GLRX.2	0.825564157850243	GAPDH.2	-0.470684268395611	PPIB	0.915138718280843
LOXL2	0.599366960479486	CKB.1	0.662273316505723	TRIM71.1	0.596362604497169	EBF1	0.82192829764563	RPS16.2	-0.411557552566866	PDLIM1	0.913590819123934
FBN2	0.591327021879751	HMMR.2	0.661823331928997	MFAP4	0.58992764260253	TLCD3B	0.821327822696889	SNHG14.3	1.06301574869029	PARP14	0.895489636041461
TSPAN18.1	0.558749320353174	HNRNPH3.2	0.6610509121551	APRT.1	0.588245887499366	EPB41.2	0.819526416393971	DST.4	0.946238720168441	PPP1CB	0.892362978788143
ITGA1	0.558692163743399	SUPT16H.1	0.65829469047102	ATP5PD	0.58679079318312	CEP170.2	0.818064003621351	RPL9	-0.368063470191681	PON2	0.888444715468323
ROBO2	0.558600791751898	RAN.2	0.656375897080857	CACYBP.1	0.584300311985264	FXYD7	0.816833341003347	RPL37A	-0.361894389778511	ARL6IP5	0.884455968138408
MAN2A1	0.554752666957929	LBR.2	0.656314529954277	PTTG1.1	0.582953802409315	THSD7A	0.816477782153384	BTF3.3	-0.426782299602738	NFIB	0.868384375413634
FILIP1L	0.531402179680548	CDC20.2	0.655482057091095	ASS1	0.582325987052235	DPYSL2.1	0.814577791282667	RPSA.3	-0.444736141937355	S100A10	0.864574520715796
PDGFB.1	0.528763920079355	ISYNA1.1	0.651023708625049	RPS27A	0.58157782369894	ATP6V1G2	0.813185024985899	TMSB4X.4	-0.850226343320751	SQSTM1	0.863965199204399
TMEM178A	0.495474531543821	H2AFX.1	0.648996103174209	PPP1R14A	0.580170619067822	GABARAPL2.2	0.808168658873047	NEFM.5	-0.505541679486524	VCAM1	0.863694854113011
COL8A1	0.468231661844611	HNRNPDL.1	0.646537467670937	SLC25A5.1	0.577323592402375	NKAIN4.3	0.806804134162174	COX7C.4	-0.386023326094457	GDF10	0.84671715920677
ANKRD45	0.436967273592068	H1FX.1	0.645637812605985	CKS1B.2	0.574381749427681	AC004540.2	0.805693953370321	PFN1.3	-0.495020176494227	NFKBIA	0.844670061544186
L1CAM	0.428913773362016	NMU.2	0.64299977313345	MPP6.1	0.570669736379535	MARCKSL1.3	0.803717264935433	RPL6	-0.357361759862929	CD63	0.843762403066638
EMILIN2	0.417366639926781	DLGAP5.2	0.640877776807142	H2AFZ.2	0.570394442307679	PMEL	0.801002186079714	COX7A2.3	-0.421142873879261	PSMB8	0.839674862558038
CRYBG2	0.416206138581132	PA2G4.2	0.637117931103468	CXCL12	0.569517963197547	GADD45G.1	0.799270266655794	RPS9	-0.371972875153788	SELENOM	0.838346258739127
PLXND1	0.41473562318733	PIMREG.2	0.634682218187662	MRPL11.1	0.565808175461887	RBFOX2.2	0.798274379478078	COX4I1.1	-0.364495445672101	OPTN	0.836716110443526
IL11	0.39604783585736	DBF4.2	0.632069910409739	SLC16A1.1	0.56072774056526	CELF4	0.794517214975758	MDK.4	-0.567742541939818	TENM2	0.835278640797246
AC145124.1	0.394793357081865	RRM2.2	0.631454446055395	CDC20.1	0.557111750156853	C4orf48.3	0.789317769314869	NDUFS5.2	-0.366964846140228	CST3	0.826800649067267
VGLL3	0.325092189600435	HNRNPA3.3	0.63056377059773	DDX21.1	0.551636804431197	GPC2.1	0.787480880557959	UBA52.1	-0.341284758647991	ATOX1	0.82105903991674
MYH10.1	0.855190563109248	EXOSC8.2	0.628879913803396	RBPMS.2	0.549726675309306	GPM6A	0.773244136968786	NDUFA13	-0.33112049324837	TGFBI	0.817851189458548
SAMD4A	0.383345180328851	MND1.2	0.625509283166704	ATP5MC3.1	0.54863061997675	SSTR2	0.772070305273547	ACTG1.2	-0.672553137627643	RAB32	0.809869116787572
BCAR1	0.566565108157233	HSP90AA1.1	0.625201779526614	HMMR.1	0.546879551646922	LHX1.1	0.768722091577389	RPS26.3	-0.416109109721126	LGALS3BP	0.808705404807476
MIR4435-2HG.1	0.61963116823022	SMC3.2	0.622921692633662	NPM3.1	0.546309914321338	C9orf16.2	0.734704530871557	RPL37	-0.330634616382736	CDKN2A	0.806824180784154
ACTN4	0.764518579577192	PTMA.2	0.62142582549999	EEF1B2.1	0.543820640274151	EBF3	0.733714174389583	KCNQ1OT1.4	0.924358127101848	CEBPD	0.800656315715373
DIAPH3	0.578020667373658	HNRNPR.3	0.618697715614472	MRPL12.1	0.539600397514088	GABARAP.4	0.733464306483334	ATP5MC3.2	-0.435672619868469	IGFBP2	0.796906107770142
RHOC.1	0.824131754947296	HNRNPC.2	0.618537185322351	PA2G4.1	0.539276472610733	GNAO1	0.728760635412543	FAU	-0.322815025775303	GBP1	0.792606411413144
GSTP1	0.682576258079119	TOMM5.2	0.618517288991584	RBPMS2	0.538810827062643	RND2.3	0.728545038809414	RPL27A.3	-0.390477430493451	SERPINB6	0.778687130427428
FSTL1	0.900292864166604	PAICS.2	0.618251040724201	SERBP1.1	0.538597743266569	SEC11C.1	0.718251953076726	WSB1.3	0.964197552062248	NR2F1	0.778348728855804
TENM3-AS1.1	0.475478370851536	EZH2.1	0.615939106202286	LYAR.1	0.535101919408316	CCDC50.4	0.704761982854367	TRAPPC6A	-0.473551825732888	NT5E	0.766861773727841
POU3F2.1	0.777789386306396	MIS18BP1.2	0.612534574034261	HMGN5.1	0.530751107699828	ATP6V1G1.1	0.699909142738036	CALM1.2	-0.470128292840483	NPC2	0.762560265780418
TUBB6	0.796126457172748	RPA3.2	0.61117769595807	ATP5MC1.1	0.530149722800211	TTLL7	0.696505521421273	PSMA7	-0.383256088793621	DKK3	0.761870237754392
SPARC.1	1.0394066901699	ORC6.3	0.610482025304756	NANOG	0.529755969427414	H3F3A.3	0.696382978136079	IGFBP7.4	-0.368901883434133	PHLDA2	0.758096099301354
AOPEP	0.520793646361734	SIVA1.3	0.605837438641542	BOLA3.1	0.5288488808238	NSG2	0.693959162169836	FTL.4	-0.820658613949403	S100A16	0.754586300377345
SERTAD4-AS1	0.382449094742745	ATAD2.1	0.605642932082455	SET.1	0.522716481758039	RTN3.1	0.687192353380872	EEF1B2.4	-0.420868792759208	RDX	0.746318863411011
NRIP1	0.636587242075481	SMC4	0.601887501514783	ABRACL.2	0.521528576648407	RPAIN.1	0.676136453823263	PTMA.3	-0.616691392934555	TMSB10	0.7384791980036
PCSK1N.1	-1.59934722307127	UBE2T.3	0.601456305062347	NTS	0.515555010769317	TUBB.2	0.674504583994604	NKTR.1	0.736385716740096	ADAM9	0.736584638476694
MSRB3.1	0.425782164252903	DUT	0.59791648390868	FAM136A.1	0.514001806055467	POU3F1	0.664734047256436	RACK1	-0.355287109883193	LYPD1	0.734531444166653
IGF1R	0.709170395359155	AURKB.2	0.581890168663307	KRT19	0.513430614829177	CPLX2	0.660334901926853	NME2.2	-0.396076456833389	WWTR1	0.73183053158978
FRMD6	0.483785116254315	NAE1.2	0.581864241295053	VRTN	0.511584231526467	RTN4.3	0.645667209544312	WASHC3	-0.423365141541587	TCEAL3	0.728070092763236
ATF3	0.727417287728102	GMPS.1	0.579813933825869	C9orf135	0.506805896676046	DNER	0.638302876813077	MACF1.4	0.723424318935139	SLFN5	0.725712570016724
TGFB2	0.585960956229068	SOX3	0.577823965419934	BCL2L12.2	0.505415162645138	GABRB3.1	0.633065108129346	RPLP2.1	-0.273538622714049	CYTOR	0.723877658881134
CYTOR.1	0.76675690013168	FUS.2	0.577143392988738	SNRPD1.1	0.503299804065327	PIK3R3.1	0.626654560533113	COX6B1.1	-0.33989764628321	PSAP	0.72365295814455
NECAB2	0.345418657129208	DNAJC9.2	0.573847220798381	PSMA2.1	0.501731424663293	BTBD17	0.624353959462466	PPIA.2	-0.348214971196242	CD151	0.718639339558584
FERMT2	0.65083794943619	PTGES3.2	0.573735652799454	SINHCAF.2	0.497107475969776	NEUROD4	0.622850122367455	RPL13A	-0.290382095542485	STAT1	0.716990514151614
SERPINH1.1	0.71184210574038	LSM4.1	0.573127415628965	MIS18A.1	0.496066241344284	H3F3B.2	0.619288149728741	UFSP2	-0.377706561531743	IFIT1	0.716058198957485
CXCR4	0.470219130380502	YBX1.1	0.571698906659739	RPS12	0.495568042503574	LINGO1	0.618564028940565	UBB	-0.377262358988442	COL3A1	0.715467699519579
RBPMS.1	0.544563719025099	DNMT1.1	0.570304676463176	PAK1	0.492160898201407	PELI2	0.617306421719334	RPL36.1	-0.274347077127581	LOX	0.715042786151163
AMOTL2	0.476719445697515	PHGDH.2	0.569098429244522	OLFML3	0.490500213521608	KIF5A	0.613659666626475	RPL31	-0.310544802810981	COL11A1	0.714417135161351
TNFRSF12A.1	0.842174245709227	CCDC34.1	0.568526522146116	PLAAT3	0.486958889034036	FNDC5	0.613139231434301	FKBP1B.2	-0.403825401389164	PGLS	0.713737940008623
UTRN	0.499142847278615	HSP90AB1.2	0.568281854725091	NMU.1	0.481778557821965	COX7A2.2	0.61100777421797	UQCRH.2	-0.387880600607908	ALPK2	0.711492318081284
TPM3	0.586253624728164	RFC4.2	0.565196271633109	RPS23.1	0.476562402401909	CHGB	0.610083968585876	SEC61B.1	-0.341521275077979	ADGRG1	0.710885513762897
GJA1.1	0.733165304136482	NAP1L1.1	0.564955541747155	POLR3K.1	0.472668139723359	IGDCC3.4	0.602714750709321	COX5A.4	-0.433471181987696	PDPN	0.710549751734811
DUSP1	0.491250489233346	USP1.1	0.562817190208935	CCNA2.1	0.472476298967415	INSM1	0.596207301985218	MFAP2.2	-0.443898683670625	NDFIP1	0.709814419783608
P3H2	0.303342043926487	DTYMK	0.556418587222086	NUDT1.1	0.471868954674047	CALM3.1	0.594182857457744	NDUFB11.2	-0.368529178641058	RAB13	0.704872448789857
RRAS.1	0.590539776578122	SINHCAF.3	0.555565426119824	MRPS9.1	0.471109349376635	C14orf132	0.588672178447499	PSMB1.1	-0.354664509535706	FEZ1	0.701491709335573
AXL	0.374696620507025	NCAPG.1	0.555529844070248	MAD2L1.2	0.47077326498961	PKDCC	0.584251884446174	C1orf54.2	-0.429751421785062	CTSB	0.700999071674313
DUSP15	0.337933041291325	HMGA1.3	0.554299402580392	CENPH.1	0.470755020534222	ATCAY	0.583062569984312	EIF1AY.4	-0.472680499174682	RHOBTB3	0.700391779479274
TLN1.1	0.656262991448022	PHF6.1	0.553100669568545	CTSC	0.469326113615194	MAPK8	0.581019500676421	MYL6.3	-0.447333697600152	FEZ2	0.697711479989816
ETS1	0.340596764127236	XRCC5.2	0.549456842156344	DDT.1	0.467585459056815	ATP6V0B.2	0.576749701748231	TPI1.1	-0.363796270201253	FBXO32	0.69702505346447
IGF2BP1.1	0.590885226628404	CCNA2.2	0.543119664468904	RPL8	0.464605419145285	NDUFB10.3	0.574606109038056	RAB8B	-0.383966298607687	POLD4	0.69220516454255
VIM.1	0.658946076655633	CNBP.2	0.541359566931709	NMRK2	0.464013528768584	EBF2	0.574313234896696	CIB2	-0.381368434021975	ANXA5	0.683566438765441
TXNRD2	0.313983937863103	VRK1.3	0.539038246894882	CCNB1.1	0.461276288650682	CHGA	0.568117498195371	RPL27.1	-0.297762706420897	SH3BGRL3	0.683052951105058
RAB31.1	0.645890168845436	ILF2.2	0.538229797499327	RRAS2.2	0.459221595294524	C18orf32.4	0.566921728655496	RPL10A.3	-0.344252427628773	NIBAN1	0.681698154918815
THBS1	0.401667638822214	CENPU.2	0.53373537067301	SNRPG.1	0.458587793138115	TP53I11	0.545444144091407	RPL14.2	-0.30372110139644	NFIA	0.678289400035263
SCRG1	0.411616382381499	HAUS1.1	0.531466318132758	ARMT1	0.45769979262791	PAK3	0.541356245399627	RPL36A.2	-0.309919882189789	COL5A2	0.676794146488572
CDKN2B.1	0.7873644421384	ANP32A.1	0.530885966744721	TIMM13.2	0.457649350962078	NDUFB8.1	0.540611090616668	PPIL3	-0.355720236821028	FBLN5	0.676459971659361
EIF1AY.1	0.485041518045736	POU3F2.3	0.529361082434399	SNRPD2.1	0.455230167039862	NDUFA4.2	0.536342156994145	SNX7.2	-0.388395943878043	PALLD	0.673473276851002
CHCHD2.1	-1.34063538307067	MARCKSL1.2	0.524716356672476	MYBL2.1	0.455017132730265	JPT1.1	0.534923442891396	EIF3K.2	-0.377089718161034	A1BG	0.672285312950537
C12orf75.1	0.748913500107408	ERH.2	0.523893884382871	QPRT.2	0.454881281942817	CELF5	0.526489490970179	NDUFB2	-0.330366999701648	RRAS	0.671905509911732
SORBS3	0.426893654974331	PSIP1.2	0.521216778500452	SALL4.1	0.452863019060682	ROBO2.1	0.524667469270515	AC090204.1	-0.359387545158572	P4HA2	0.67128160332498
PALLD.1	0.67484709743857	FBXO5.1	0.519966240787662	RBM8A.1	0.452801474894176	NAP1L5	0.52441137444218	PFDN5.1	-0.31095901873177	ENC1	0.670768933014111
SERPINE1.1	0.698831567849598	RFC2.2	0.518788829204057	MTA3	0.451882032103221	SNAP25	0.50633330910155	HSP90AB1.3	-0.473371346037093	CAST	0.670705239323627
STK38L	0.459109689715904	GLI3.1	0.518691857851197	DAZAP1.1	0.447812117618751	CELF3	0.491756102855433	TXN	-0.383972789579831	RHOC	0.666886103730128
EFNA5.1	0.475164474315656	GCSH.2	0.516326213563544	TNNT1	0.447289367786327	SERPINI1	0.485690395606667	NQO2.1	-0.375474824707738	DCLK1	0.66675882107912
MAGED2	0.643309020683066	H2AFV.1	0.514095060215646	LSR	0.441302877503072	COX6A1	0.482468098790223	ATP5MG	-0.323423004911034	IFIT2	0.664192783834442
CAP1	0.649380408298082	KIF11	0.513343652066747	RPLP1	0.439762885377426	SYP	0.481507080805604	EBAG9	-0.350506488951707	EIF2AK2	0.663466608101449
IFITM1.1	-1.53627838567139	HMGB3.2	0.511675717805602	RPS17.1	0.437473823334522	UQCRB.2	0.476442102934379	FUNDC1	-0.354191071030501	NFIX	0.653329667064104
ID4.1	0.771446666503843	ALYREF.3	0.508405613348285	RPL14.1	0.436626164255778	NDUFB11.1	0.474451529346358	RPS27.1	-0.261960936999755	TNFRSF12A	0.650028549662343
PIM1	0.477800190246317	LRP2	0.507398535943917	ATP5ME.1	0.436153936802779	XKR4	0.471846302002783	DBI	-0.394903965589794	ALCAM	0.649964836083328
ARPC5.1	0.566207042874185	MCM10.1	0.50546307507557	MIF.1	0.435055618447127	STARD4-AS1	0.457787097783183	RPL23A.1	-0.285453409140925	JAK1	0.644094363957258
TNFRSF21	0.363310633611504	CENPN.2	0.503208769851564	RPS16	0.433618606977464	DNAJC12	0.4560135823475	UNC50	-0.351160962343225	HLA-E	0.643005575868338
NUAK2	0.393869905315932	LSM5.2	0.500991169904106	COX5A.1	0.430156782120987	SRRM3	0.455403824726971	COX5B.4	-0.354770165759024	FAM114A1	0.63931390116923
ACTG2	0.552411941211048	SRRM1.1	0.500663926779283	COX5B.2	0.427557937782178	GREB1L	0.453724689027028	RPL22.2	-0.282555820166141	JAM2	0.638925019251152
LGMN	0.558411651105066	CCT2.3	0.499588601800117	APELA	0.422422787111634	RNF165	0.440659297564199	AC100810.1	-0.35896703133013	RNASEK	0.637764792020967
STK26.1	0.436504467326963	CDCA8	0.492498287318595	RPSA.1	0.421608615530312	COX5B.3	0.437365804674553	PARK7	-0.326003620254415	PMEPA1	0.634472794062134
PLAGL1	0.484428516231671	PRDX2.2	0.490084364476	DLGAP5.1	0.418557319687611	COX6C	0.435679230558536	BBS7	-0.348658429423252	EVA1B	0.633999708032138
NUAK1	0.501875491512922	NUF2	0.488314697200368	PNP.1	0.413991990634429	TMEM169	0.424258224251505	CMC4	-0.354784166900363	EFNB2	0.632629578534314
ITGA5	0.330843836212094	POLD3	0.485481965807214	COX7B.2	0.413575441356695	SEZ6L	0.42230321471404	ATP5PO.3	-0.339952147408974	PJA2	0.631075397401434
RAI14	0.618751586167535	BRCA1.1	0.483714887504389	RPS6	0.407970516389053	NAPB	0.416227616366003	SLC39A3	-0.348340617600433	QKI	0.629541507670924
ZYX	0.60661302881042	ZBTB16	0.483497671096417	CDCA7L.1	0.407616808939258	SHF	0.399688594319793	CCDC88A.2	0.609729217044433	SERPINE1	0.625899622519738
PDGFC	0.332252956532301	UBE2I.2	0.47993814763992	CAPG.1	0.395935859654603	KALRN	0.389544693356487	ARL6IP6	-0.350381900460871	C1orf122	0.623887701321915
GADD45B	0.430353040978656	CHAF1A.1	0.479764199317168	PFN1.1	0.388408704321135	ASPHD1	0.382309157606491	ANAPC11	-0.345037140215097	CALU	0.621751383900781
GNG5	0.408936078252154	HILPDA.1	0.466422998599913	RPL15.1	0.387740346899694	RPS15	-0.349263198040503	EIF4G1	0.428687292014735	MMP24OS	0.618168489731238
ARID5B.1	0.647337924048933	RAD51AP1.2	0.464226926931102	PIM2	0.385132831571119	RPLP1.1	-0.402734973464934	SELENOW.1	-0.393338908366718	GUK1	0.615405233791444

MDK.1	0.4463708319722	MYCN	0.460920858108768	RPL26	0.384949612417004	RPL23A	-0.445003765811567	COMMD6.3	-0.351248594424788	MAML2	0.612774380854396
RGS5.1	0.665963969491897	FANCI.1	0.460665740556361	F11R	0.381135614529579	RPS12.1	-0.445260834065912	COX8A	-0.296288397469427	LAMA4	0.612655156529807
TGFB1	0.453949052306808	TSPAN18.2	0.457161310884977	RPL12	0.371081182572354	RPS26.2	-0.455249494174514	ATP5PF.1	-0.306808865669395	IFI16	0.611447607771046
NECTIN2	0.546060055836562	HMGN1.2	0.450511247086213	NDUFA4.1	0.370608535592708	RPL36	-0.459405168192649	C12orf76.1	-0.338725593739977	MICOS13	0.610270155339902
CLDN6	0.525576952651343	PRIM1.2	0.449974762122007	RPS3.1	0.369616830806775	RPL27A.2	-0.468728777013947	COX6C.1	-0.316522471128905	XIST	0.606301109812039
PDE4D	0.375151123188899	SNRPG.2	0.447214806511133	FLT1	0.368735519321335	RPL12.2	-0.474996242036766	GSTP1.3	-0.510225063856405	VGLL4	0.603814618464078
CTDSPL	0.310741052602315	ATP5MC2.1	0.446300261773054	COX7C.2	0.368667553160986	AP2S1.1	-0.495231190027948	UQCR10	-0.282415087755861	TMBIM4	0.595786362470129
EPB41L2.1	0.496593679039149	COL2A1.1	0.441431853745076	RPL19	0.363799437922577	PRELID1.2	-0.499781534799096	STX8	-0.379824860788459	ANXA1	0.595193378197518
PTPRD	0.421209288494448	HYMAI	0.439886847587712	RPS29	0.363158243471044	RPS18.1	-0.506790900443757	DOK5.2	-0.41255563105949	RCN1	0.594984542546117
MFAP2	0.500251021071323	EEF1B2.2	0.438516251261198	BTF3.1	0.362637265614904	SYPL1.1	-0.515145262802395	GSTZ1	-0.376484329812762	CCPG1	0.594800884442169
RNF217	0.314373417907116	SPC25.1	0.436100063223115	RPL18A.1	0.362333301219875	PDIA6.2	-0.536611442051524	PSPH	-0.349142488829333	GYPC	0.592702020843529
CDC42EP1	0.329963392160471	LSM7.1	0.427807356308687	RPL22.1	0.355822672050209	EIF2S2	-0.544796501782235	FAM104B	-0.322220581395834	ZIC1	0.591817447701588
LIMCH1	0.425628729247355	PBK	0.417039726273544	AC104461.1	0.35225418956412	SSR3.2	-0.54594934774423	UBE2Q2.1	-0.314945473757265	PLTP	0.590767102421529
EMP1.1	0.499659166281689	MDK.2	0.411741460019496	RPS3A	0.350068128718876	RPL41	-0.550780999931291	COMMD10	-0.338304609292981	SYT11	0.590091523171207
CDH2.1	0.500345902449749	PTCH1	0.411195769946525	RBM47	0.347876540611191	SPART	-0.559444328687697	PRDX1.3	-0.523194660209479	AHNAK2	0.588264682356294
PFN1	0.437252256796317	SNRPD2.2	0.409171419497727	RPL11	0.341421155334221	CIB1.2	-0.562658816512046	H2AFJ.1	-0.399709267917207	IFI27L2	0.587788858380069
NFIA.1	-0.764793804259683	C21orf58	0.406550458601762	RPL24.1	0.333097008482793	AGTRAP.1	-0.571772008186654	PIGBOS1	-0.317843208590034	APLP2	0.585685354956458
PHLDB2	0.343815286274548	SOX1	0.377237288703192	RPL5.1	0.332438285366594	GPX8.1	-0.610936222491899	C17orf75	-0.306205049170296	OGFRL1	0.582513819942673
ADAM19	0.341031824488866	PPIA.1	0.370907876576606	RPS25	0.332057655433108	CNN3.3	-0.61480794200737	SRP14.1	-0.289915966702853	P4HA1	0.579472759780624
CAVIN1.1	0.717797534817717	RPLP0.2	0.37009554178718	RPS7	0.330022708089035	PFN1.2	-0.621730366732473	MDM2.2	0.447941276761602	LHFPL6	0.579269582878181
CDV3	0.58028458546674	RPS17.2	0.368218883956832	RPS21.1	0.329243203175308	REST.1	-0.624703495175854	JPT1.2	-0.400496859411215	CRIP2	0.577879586858476
IKBIP	0.646247088675335	DEPDC1B	0.348340040307627	TPD52.1	0.325222210274678	PLS3.3	-0.64163204674212	TEAD2	-0.339208908099069	EPS8	0.577087382711214
VCL.1	0.65032471936541	SUMO2.1	0.345963281663666	RPS5	0.323905597782967	RRAS.4	-0.65693923808391	RAN.4	-0.39828626505143	CALD1	0.576420206323053
SYTL2	0.383758502497759	RPS23.2	0.310963506124441	RPS24	0.322334992913416	CAVIN1.4	-0.682337874433008	TMED1	-0.316804555946563	PEA15	0.576037232629987
CITED2.1	0.51648858355382	LMO3	0.3047518591818	RPL23	0.310971802615695	PDLIM2.4	-0.686275206007485	TOMM7.3	-0.342166597771181	TLN1	0.575449414055897
OCIAD2	0.655490679455622	FABP7.3	0.268046575198888	RPL17	0.30799117375307	PPIB.3	-0.695189392679953	BET1	-0.342494685488938	OST4	0.574626145606239
MTCH1	0.549659422439285	PRDX5.3	-0.549051448122893	FRAT2	0.307678758466867	NME2.1	-0.712668437310498	PSMB6	-0.316684099075013	JUN	0.567594194369978
SEPTIN7	0.441780992951536	LAPTM4A	-0.587976358153012	RPS28	0.30647928627132	TLN1.4	-0.7182900676142	PYM1	-0.310452772109591	ATP5F1E	0.564099446747754
FST.1	0.641552886505371	DSTN.2	-0.614908513463362	LDB2	0.303185384179471	AHNAK.4	-0.725138383529652	GIHCG.1	-0.281828108337951	CA12	0.564015482463012
FJX1	0.499841915507586	RABAC1.2	-0.626666991088705	RPS18	0.302663158595357	CALR.2	-0.727836300214077	TM9SF1.1	-0.302475823989464	COL6A2	0.563761149797591
ANXA11	0.365981691805835	ANXA5.1	-0.706072810518958	RPP25	0.296981014747343	LIMS1.2	-0.732229781072184	BEX3	-0.324855579002068	PHLDA1	0.559916284170108
EDNRB	0.442537397202285	RNASEK.3	-0.707131811804309	RPL35A	0.294755982708024	CCDC80.4	-0.748583367675199	SPC25.2	-0.370568343531276	TCEAL9	0.558866440224919
LGALS3.1	-1.84339487579481	AP1S2.2	-0.738033749101812	RPS15A	0.280875739821508	CALU.4	-0.749747042716352	EIF1.3	-0.339580720221673	SORBS2	0.556956410075438
LAMA1	0.303106754814552	OPTN.2	-0.743225353992016	CCDC169	0.270838049238542	NPC2.3	-0.77544072115786	PPDPF.2	-0.330229512994275	TMOD3	0.555193464360989
NID1	0.281068532373657	ARL6IP5.2	-0.749455657489146	RPS14	0.263920645769957	VCL.2	-0.78403868841742	TMEM60	-0.307806315783583	ZFP36L1	0.554408984199701
LRATD2	0.324554638266859	PDLIM1.1	-0.798589545700514	OST4.1	-0.501586737117935	ENO1.2	-0.796662723201516	C17orf58	-0.291840005438007	DST	0.554268460470045
ANXA2.1	0.663828743441488	PHLDA1.2	-0.822237215130106	TMBIM6.1	-0.503901962523294	QKI.2	-0.79854339805942	MTRES1	-0.301092650610271	GLIPR1	0.55176167784669
FHL3	0.377469915972842	ITM2B.1	-0.842934699265722	PPDPF.1	-0.54090290354128	FLNA.3	-0.831982632374495	CAAP1	-0.301925511349588	GNG12	0.551315853791722
CNN3.1	0.426548786065632	CD44.2	-0.878822400824681	MGST3.1	-0.543933278590056	SERPINH1.2	-0.849507706012723	NDUFC2	-0.300307401455539	RGS4	0.548128057122806
ELFN1	0.265202243766776	TMSB10.2	-0.882538046719811	CNN3.2	-0.552659802643942	EEF1D.1	-0.86362966825469	MTLN	-0.340294323186394	CSTB	0.543567970106055
PTGR1	0.406357232787069	CD63.2	-0.892545671429311	HP1BP3	-0.562008665859315	ARHGAP29.3	-0.871644857605665	IFT52	-0.349723145811972	UQCRQ	0.540668656562497
NR2F1.1	-0.990730697753692	SH3BGRL3.3	-0.896625477184414	VPS28.1	-0.576735245662529	GNG11.4	-0.878245547770581	ARPC3	-0.339099124558201	BLVRB	0.539043952631985
DSP	0.50152530764327	FTL.2	-1.06026050917919	YWHAZ.1	-0.580874050484134	CD44.3	-0.890675367269189	DNAJC15.4	-0.436441796716621	AHNAK	0.538111329859023
LUZP1	0.549856279411299	SQSTM1.3	-1.10066034215897	GUK1.1	-0.58119447937845	CCND1.2	-0.915881124448405	AKAP9.2	0.62407591762805	COL6A1	0.536562178265076
NEDD9	0.388940743772602	TSPO.3	-1.11853511895291	KIF5B.1	-0.583913186235496	UPP1.4	-0.935815476597616	BOD1L1.1	0.526576913779806	P4HB	0.534056738020225
IFITM3.1	-2.32383302059951	FTH1.3	-1.31820473357103	PGLS.1	-0.587142653790395	PALLD.4	-0.942129753817665	NUDT9	-0.296822326266473	VAMP5	0.533840115162369
PDLIM3	0.31125066996188	HLA-C.3	-1.32492042542586	DYNLT1.1	-0.595431976193495	HSPB1.4	-0.949193255796474	LDHB.3	-0.36439166553968	TMBIM6	0.53193917412524
CAPN2.1	0.560182993075112	CAV1.3	-1.41008650166206	ARPC5.2	-0.596475146266502	GSTP1.2	-0.967920083728418	SPPL2A.1	-0.3186122944003	PLAUR	0.531267588172876
CORO1C	0.472506309828435	CHCHD2.3	-1.45236588910892	RTL8C.1	-0.601555334191619	CYTOR.4	-0.97710361662313	IFT74	-0.310711165012541	IL6ST	0.530672126477762
TUBB2A	0.599341411817969	PMP22.3	-1.47071558374426	DDAH2	-0.602951534190541	PDLIM1.2	-0.983678673088024	HMGN2.3	-0.566516433129349	HSPB1	0.52654526718074
EIF5.1	0.443276354661106	S100A10.3	-1.48452102905533	COTL1.1	-0.605817226116498	CLU.4	-0.997623742894822	SPA17	-0.307154434584249	IFI35	0.52494092677225
MUC12	0.271366409452779	ANXA2.3	-1.54895235420743	FEZ2.1	-0.617725459875703	RPL22L1.2	-0.999672343091376	RBM7	-0.286077155407966	TGFB1I1	0.524334999834619
MYL12B.1	0.499087102480143	IFITM1.3	-1.64500714040487	PPIB.1	-0.629792820421944	IGFBP2.3	-1.00458277889222	PHPT1.2	-0.349995024086563	NR2F2	0.524334450680435
EIF5A.1	0.452984784290459	GAP43.2	-1.7114219526785	PTTG1IP.2	-0.632249835503388	ZFP36L1.3	-1.013354914955	ING2	-0.280625330425414	CNN3	0.523168985636322
TMEM88	0.386492327786935	S100A11.3	-1.76228420792688	DCTN3	-0.634727750472576	RAB13.4	-1.03432743053587	NDUFB4.1	-0.349668187546196	SMDT1	0.522446142252954
ODC1.1	0.395259934275998	COL1A1.2	-1.80493511493033	C1orf122.1	-0.642720532908756	ACTN1.4	-1.04632528388815	PIP4P2.1	-0.341323254875217	GNAI2	0.522434877963943
CXCL14	0.392782683854709	PCSK1N.3	-1.86609434345947	C18orf32.2	-0.649342467559171	EMP3.3	-1.07327680561044	PABPN1	0.498402366080515	SP100	0.52212414431735
WDR1	0.586895268574743	B2M.3	-1.87370590679301	CALU.2	-0.653185596122778	TSPO.4	-1.08024603526526	KMT2A.1	0.544423093136323	GINM1	0.52171891444892
SLC25A24	0.400390093440183	LGALS1.2	-2.00369199440043	FZD3	-0.65790658869313	PRDX6.4	-1.09948295622913	ENC1.3	0.365099855906221	ITGA2	0.521273838455711
SHROOM3	0.434652431422697	LGALS3.3	-2.09960988129022	CCNI	-0.665229881364382	TTYH1.3	-1.11888571063378	PRRC2B.1	0.479870265897807	RABAC1	0.518467009121018
TFPI.1	0.523034872535843	CCL2.3	-2.4230722974559	RNASEK.2	-0.704176080425992	COL4A1.4	-1.12515166834225	GABPB1-AS1	0.720556512732262	TMEM50A	0.517447914436593
GPX8	0.459742902860038	IFITM3.3	-2.61948358468653	CD47.2	-0.710983665391555	MGST1.1	-1.18725862932371	DDX17.1	0.573923129969855	FNDC3B	0.517438398767867
HABP4	0.387887675013865	DST.2	-0.867981249930568	PSAP.1	-0.711378726916095	GPM6B.4	-1.20415011477487	SET.3	-0.42724410812255	SH3BP5	0.516643354215753
DSC2	0.354506953713053	ATAD5.1	0.442388405471384	PPP1CB.2	-0.711516415322573	CHCHD2.4	-1.2088286474044	DYNC1H1.2	0.534082948250575	HERPUD1	0.51473697697363
SRPK1.1	0.549718259314961	SAP30	0.464295667209861	RTN4.1	-0.71711030592095	TAGLN2.3	-1.21738523227512	RBX1	-0.30707005091748	ACTR3	0.509722461961147
PLK2.1	0.610748260993445	LSM6.2	0.501762711802468	CD63.1	-0.723826728234609	WLS.3	-1.25256166240972	TXNDC15	-0.288387044498147	CAV2	0.509595965240031
COL2A1	0.337716277162843	CACYBP.2	0.620125847926038	SEPTIN11.2	-0.726861275387922	TNFRSF12A.4	-1.25372776584845	GOT1	-0.298678720277524	LAMP2	0.508038306602431
SERPINE2	0.75526886239704	PDCD5	0.438351337053549	ATOX1.1	-0.732879529724234	TIMP1.4	-1.26655019623556	MVB12A	-0.359852082628317	SCARB2	0.506557742844677
SULF2	0.382521735347447	HIST1H1B	0.701090832928894	EFNB2.2	-0.74266637221453	CD99.4	-1.28991629629557	BCL10	-0.298373477698345	FTL	0.506505533346009
COL5A1.1	0.492934586450385	HLA-B.3	-1.56700983139115	PTMS	-0.751492185064978	PMP22.4	-1.43184906578071	GOLT1B.1	-0.266752955433766	LRP10	0.50546149022465
SLC44A2	0.420284180014906	SSBP1.2	0.406917940948292	QKI.1	-0.760618415081613	SFRP2.3	-1.49668458002671	ITGB3BP.1	-0.355097199142659	SDCBP	0.503015670219897
CAPG	0.475327087732843	HNRNPU.1	0.728944116450419	ANXA1.2	-0.768436975231957	ANXA1.4	-1.53171380875025	FUT8	-0.295029009272489	TMEM179B	0.502459514843419
PFDN1	0.534989923061248	CCND2.2	0.554641491712631	PJA2.1	-0.769952615549779	SPARC.4	-1.5821523214411	YWHAB	-0.301650250589007	GAP43	0.502408037616776
ANKRD33B	0.297325908556152	H2AFY2.1	0.486719378178871	BASP1.1	-0.771162597485841	CAV1.4	-1.68128023127976	OSTM1.1	-0.307849168412162	CBR1	0.500633575988674
SUGCT	0.34349107401201	PNRC1.2	-0.615172994131491	RDX.1	-0.774785601883634	COL1A1.3	-1.69693014269839	PTGES3.3	-0.372080522920851	PRXL2A	0.499595180687956
WLS.1	0.379054982776833	CSRP2.2	0.438002252562383	RCN1.2	-0.790597938252236	CALD1.4	-1.88707403886502	MICU1	-0.276371836038103	PRRX1	0.499507698968891
COL11A1.1	0.53801905342435	BCAT1.3	0.534259826595193	LHFPL6.1	-0.793821631181099	PTN.4	-1.88715495473731	ATP5MF	-0.279880148608964	NPAS3	0.497917125368945
TIMP1.1	0.478933169640257	SNRPA1.2	0.532985318666782	PON2.1	-0.795750197149839	B2M.4	-1.88781119474224	PDCL	-0.274597852360979	CIB1	0.497821377010796
SDC2.1	0.489640678570977	HMGN3	0.441696594740162	CD59.1	-0.796887530643774	LGALS3.4	-1.90724434745315	SRPRA	-0.28783524303922	C5orf56	0.492017530563145
CDK6	0.569378032017445	LMNA.2	-0.899318142424617	RHOC.2	-0.797070803404366	TPM1.4	-1.91708067132119	SSBP2.2	-0.289674367028114	IDS	0.488150720976176
SNX7	0.380809392615882	RPA2.1	0.47492959327685	GNG11.2	-0.798353083113172	MT2A.4	-2.02441578177581	GABARAP.5	-0.403228639092155	SNX3	0.484502765283085
BTG3	0.495546902550843	MRPL11.2	0.554508266886624	BAALC.2	-0.804936193042763	VIM.3	-2.0764260062549	HMGB1.4	-0.557551567566111	PDP1	0.483351614776646
ILK	0.518150839200863	KCNQ2.1	0.499401594369528	COL4A2.2	-0.806921416355562	IFITM3.4	-2.52254566200227	ZCCHC7	-0.280548332690501	TMED10	0.483326771854679
PDLIM2.1	0.508434428793057	HIST1H3B	0.442955138880652	SORBS2.2	-0.85849096791844	TAGLN.2	-2.70887492769254	EEF1AKMT2	-0.2793018769831	GNG11	0.482743392987701
DNAJC1	0.456022743010876	BANF1.1	0.471255648208249	ARL6IP5.1	-0.86564778075879	GNG12.2	-0.554052434261388	DMAC2L	-0.269368249551166	PDE1C	0.479349170796786
LRRC8A	0.400769885667318	MAP1LC3B.1	-0.623299797370676	GABARAP.2	-0.873459541912746	TMSB15A.2	0.608270927872011	MT1X.5	-0.59857877392913	RNF213	0.478945480299175
TRIM24	0.567903351200292	FEN1.1	0.602297122393546	MARCKS.1	-0.884379078743675	CCN1.1	-0.864418635673965	HIST1H1C.2	-0.406522141726126	UBXN4	0.4727148658918
ABRACL.1	0.483113599979452	ATP6V1G1	-0.538394246063227	IGFBP2.2	-0.884522649629834	MIR4435-2HG.4	-0.581792917929905	ANKRD11.2	0.540237969194914	SEL1L3	0.466380602218978
UGDH	0.39480350412263	HIST1H2BH.1	0.377257885058431	ZNF428	-0.898756049034841	RCN1.3	-0.708698415135967	COX6A1.1	-0.270995459716629	MRC2	0.463489049755732
TGFBR1	0.426179626655546	CTNNAL1	0.506309726335913	TMSB10.1	-0.900608985751751	IFITM1.4	-1.48456921834907	POMP	-0.276867656845475	GSN	0.461673749217711
ATP5F1D	-0.487027212414791	CBX5.1	0.466183726193539	METRN.1	-0.921673005004441	TMOD2	0.446401624198488	ACBD5.1	-0.276745078080961	IQGAP1	0.458474733282868
CD99.1	0.41388067158037	SMARCC1.1	0.546682464878496	NR2F1.2	-0.940522440573596	FNBP1L.1	0.655922097912255	SUB1	-0.267664476843328	KTN1	0.458141221006848
B4GALT1	0.258181537157742	RPS6.1	0.303266755090545	ATP5F1E.1	-0.9417183694104	FABP7.4	-1.23190641276311	RAC1.3	-0.289490902104355	TMEM219	0.457099649922991
LASP1	0.435087971017833	CCT5.2	0.477539928765686	CST3.2	-0.957993469849357	RHOC.4	-0.740224093451471	SPTBN1.4	0.365262076834302	BAD	0.456139200278917
RPS26.1	-0.432942577124182	KNOP1.2	0.495698344475932	SH3BGRL3.2	-0.993493854409481	COX4I1	0.401816143756747	SMAD1	-0.293431768246747	CANX	0.455517777873533
SIPA1L2.1	0.421030307409701	MYL12B.3	-0.554551515440427	MLLT11.1	-0.999253864593868	IER3.4	-1.00026176982568	EDF1	-0.266512879122387	TAX1BP1	0.45543657814211
CFAP298.1	-1.37466753657773	CDCA7L.2	0.394941029968813	C4orf48.1	-1.00480534299593	EIF4EBP1.1	-0.73255270956844	ERH.3	-0.329839645003155	SSPN	0.453440067801152
TES	0.349426642949065	GINS1	0.355751354585457	DST.1	-1.02125240749584	CAST.4	-0.639787121440286	NDUFB10.4	-0.34891417527147	ADAM12	0.452829361516391
PRSS23.1	0.742932714943925	MCM4.2	0.596733795489444	MAP2.1	-1.02700237356109	EPS8L1	0.496055036794006	CDK11B	-0.251535884508056	PPDPF	0.452487827088776
MID1	0.447568308941968	CYYR1	0.350092167366324	TSPO.2	-1.03766287278862	GJA1.3	-0.647218852549797	GOLGB1.1	0.517334203688501	DECR1	0.451317038475587
HSPB1.1	0.703993137033312	TIMP1.3	-0.928758363183664	ID4.2	-1.04689646930032	SPATS2L.3	-0.566707477229016	GNAI1.1	-0.287815672196543	DAD1	0.451290255905404
IER3.1	0.499926073213807	CENPE.2	0.659761239560903	CDH2.2	-1.05271318286699	GNAS.1	0.786412597653173	LSM7.2	-0.275038956767513	CHPF	0.450712480517606
CDC42	0.400053544495075	CDCA3	0.424471225282955	ARL4C.2	-1.08111648604199	PHGDH.3	-0.647855935892759	PPIB.4	-0.49093865587592	TACC1	0.450117544172793
VASP	0.472224092495072	ZWINT.2	0.445387525243161	TIMP1.2	-1.09731939790043	NENF.2	-0.597516486207384	RABAC1.3	-0.395252365833569	BEND6	0.450076070528288
IGDCC3.1	0.446523841113919	FOXB1	0.316702884745978	WLS.2	-1.10204003076019	KDM5B.1	0.64221006562807	MSL3	-0.269012962026687	KIFAP3	0.44961409902007
NME3.1	-0.545788415584892	ATOX1.2	-0.65486634069138	TTC3.1	-1.17060339876928	APLP1.2	0.946265481635494	CELF1	0.442271838829017	MIR4435-2HG	0.448204425880451
UGCG	0.533391191568153	RAD51C.2	0.487728989058906	CAV1.2	-1.17624676584041	HSP90B1.1	-0.620843883318708	CLEC11A	-0.28341491087162	DYNLT1	0.447927880247826
PDIA3	0.434722645623836	MSH6.1	0.586859528417884	ANXA2.2	-1.25334972370646	EFNB2.4	-0.726324485473057	STAM	-0.289850012618228	IRF2BPL	0.447044194308612
MEF2C	0.254048050473731	MGST1	0.472595186262433	VIM.2	-1.30060842576627	SIVA1.4	-0.555178885186422	OST4.3	-0.357122268124396	NFE2L2	0.444596902052793
HSPG2	0.276751032716317	RTKN2	0.374514806957045	MAP1B.1	-1.3205385157769	HMGA2.3	-0.660666598897166	HSBP1	-0.277401468423094	TCEAL4	0.439821854126657
HNRNPA3.1	0.430466539884396	MYBL2.2	0.437764674891432	SPARC.2	-1.32149628083481	HAGH.1	0.603993028903044	NDUFA11	-0.263922729617668	TNFRSF1A	0.438610548729495
CCL2.1	-2.10843490653858	GPC3.1	0.450007303156466	GPM6B.2	-1.32596015915718	ANXA2.4	-1.18296577047098	SCG3.4	-0.561998055286955	C17orf49	0.438056379924713
TRAM2	0.261010869392037	H2AFY.2	0.514461248017975	FABP7.2	-1.33521322819471	CADPS	0.499772968592532	PAK1.1	-0.341296530055685	CCDC102B	0.436897621892818
TGFB1I1.1	0.38957345838702	CENPM	0.40707206705072	FTH1.2	-1.33748599810238	PTPRZ1.1	-0.606528217311843	SEC61G.1	-0.30706930292389	NR2F1-AS1	0.43437852407111
ROCK2	0.495756521212101	SGO1	0.375021714424752	PMP22.2	-1.39342698140011	NEDD4L	0.500832750675424	COX7B.3	-0.282486705653364	NENF	0.430081340640854
MIF	-0.44692302434511	DDX39B.2	0.435800042940195	MALAT1.1	-1.46976412444666	RAB34.2	-0.531191890860096	RSBN1.1	-0.257766368126207	PCGF5	0.428420400510197
KCTD10	0.321555135889565	KIF15	0.338755021061031	GAP43.1	-1.48346069589438	CSRP2.3	-0.583491676635229	EVA1B.5	-0.345974579656241	MYDGF	0.426236003575484
CAP2	0.386383174585258	PRMT1.1	0.475125183626551	CD99.2	-1.51477023323572	COL4A2.4	-0.777272331796796	AP2S1.2	-0.296123506110549	GLIS3	0.423111949094546
CALR.1	0.484919962715469	KPNA2.3	0.737049750828894	PCSK1N.2	-1.65832623444566	LIN7B	0.369171626513951	NR6A1.3	-0.375538700491775	MORC4	0.422318727843411
APAF1	0.376850959408049	RFC3.1	0.40217548212991	TUBA1A	-1.68174038065532	UBE2E3.2	0.510538414518901	SMIM14.1	-0.260522177130913	ARPC2	0.42140962722347
ENO1	0.397062434236672	SOX2	0.530708334411561	COL1A1.1	-1.69814195624489	PTX3.4	-0.947350373544132	SORBS2.5	0.38054386847413	TAP1	0.420774529548371
LHX1	0.33651819999267	TUBB2A.2	-0.647010764281073	B2M.2	-1.77762931736765	ADAM9.3	-0.547998992436042	IGF1R.2	0.3965712188658	SELENOS	0.419161510343565
FBLIM1	0.29539780940781	FBL.2	0.569012934518767	LGALS1.1	-1.82897814036386	STXBP1	0.469766417384486	ARFGAP3.1	-0.301591483593761	TMEM263	0.414586294126302
VMP1.1	0.45964439077285	LAMA1.1	0.280229852469108	LGALS3.2	-1.89944811360775	GDI1.2	0.683752171465395	MMD.1	-0.317521228651895	CAVIN1	0.414388809321221
RND3	0.465657014314547	CDCA7	0.396986774661637	PTN.2	-2.44758055944333	PSME2.4	-0.770472118359678	ATRX.1	0.497775847179023	LTBP3	0.413039511915888
PLIN3.1	0.504721762430437	SKA2	0.495654936382234	TIMM8A.1	0.441888795828039	HES6.3	1.18426779930954	GCLM.1	-0.278659621147413	MAGI2-AS3	0.403221017027604
SMCO4	0.321551620758314	GSTO1.3	-0.676553659812004	PODXL	0.495085400449791	RPL35.2	-0.456782925096565	ATP5F1E.3	-0.32899162444098	SPATS2L	0.403057598297478
CYBA.1	0.600153786874209	RPL17.1	0.272705366012682	PSTPIP1	0.416396954113956	PLIN3.2	-0.528889075701281	HIST1H1E.1	-0.338482528511061	MAF	0.402781534539815
PTTG1IP.1	0.468062816287258	SKP2	0.37325397160348	HIST1H1D	0.685830443854666	PTMS.1	0.731523803657408	SERPINB6.4	-0.324792366004215	TNIK	0.401932293820256
ENTPD4	0.32195616997606	BLM	0.337421394318781	SNRPB.2	0.473628931947217	ARPC1B.1	-0.514533606813318	H2AFZ.5	-0.673564363221262	CALCOCO2	0.401408787626581
TCTN3	0.373521930011286	NCAPH	0.340138977157608	KIF20B.1	0.438356939213126	MAPRE2	0.724216728860274	ZNF638	0.481444808827029	FUCA2	0.401102461746348
PTPRF	0.401583393710774	C1orf122.2	-0.584724164807523	SDHD	0.448005440161842	NQO1.4	-0.90010349216109	CNTFR.3	-0.275033651823312	TRIM56	0.401092642271593
TMEM159	0.303128594360914	RPS3A.1	0.260495083171546	PGAM1.1	0.45749334296821	RPL13	-0.316127688367986	MEG3.4	0.831182234086574	JOSD2	0.400687349056636
YWHAZ	0.415278100196241	POLD4.2	-0.574462743962656	CENPN.1	0.415866651380357	CTSC.1	-0.663395145457956	JPX	0.485589295239384	LIFR	0.397792786559623
SMAD3	0.311693080520223	KHDRBS1.1	0.445653088172144	RHOBTB3.1	-0.702639781135013	TPD52.2	0.511854203471013	SPTAN1.3	0.461791866776093	CAMK2D	0.396438005640604
COL5A2.1	0.436526448126956	CDK1.3	0.547162174499807	NTRK2.2	-0.868683291876495	GAP43.3	1.38064464426037	ARID1B.1	0.516092735901793	KRT10	0.394354298143644
LINC00472	0.393658427718837	LIG1	0.395224857110515	SYT11.2	-0.657219611867442	ZC2HC1A.1	0.654608543059219	HSD17B4	-0.285113483857925	MIR99AHG	0.393349372182004
PICALM	0.389158049148183	C18orf32.3	-0.599997690809211	RPLP2	0.320747958235563	NPM1.3	-0.656164773670614	PHF5A	-0.264633603758394	MTDH	0.391800733557284
CDC42EP3.1	0.441223175469024	CCDC138	0.35316300028785	NCL.1	0.511711602729791	CCN2.3	-1.29803313051076	VIM.4	-0.967077279006761	COL5A1	0.389402453820795
PELO	0.382143920511213	BARD1.1	0.430217390640655	EZR.2	0.524596639992946	PCGF5.3	-0.482034117669554	PNISR.1	0.508294992611726	TMEM9B	0.388584503057245
GOLIM4	0.435703741590161	CENPK.2	0.409579730020089	CKS2.1	0.49835814559348	GTF2I.1	0.588681301978877	CCN2.4	-0.364522871471874	C1orf54	0.387964179690329
JAG1.1	0.419978920203923	TIMP2.3	-0.783684186911255	NFYB.1	0.44452745327278	CAMK2N1.4	0.481926329086374	BMERB1.2	-0.286021748538414	IMMP2L	0.38672858179723
FAM200B	0.475694310297414	DTL	0.427008915046689	NDFIP1.2	-0.637783634287807	COL11A1.4	-0.828696528150397	EWSR1.1	0.364038250025195	LARP6	0.385880904550337
KLHL42	0.359837451142955	SMC2	0.496459596669191	PHB.1	0.460573748647151	MIR99AHG.2	-0.506340951644406	DYNLL1	-0.263282521003397	NTRK3	0.385668050742739
CREB5	0.289130318762555	KIFC1	0.329126622491807	KRT10.1	-0.533449554490459	ID3.1	-1.10402170628565	ETHE1	-0.304590841831067	TTC3	0.385643903206213
ARID3A.1	0.383599186705624	GTSE1	0.472340844048483	MZT2B.1	-0.468596541192832	C12orf75.2	-0.645221134320295	SLC25A5.2	-0.340193833024439	BACE2	0.385639418233292
WEE1	0.484282224688195	MMP24OS.2	-0.563168162253452	BRI3.1	-0.508672443554654	NKAIN1	0.441870511872906	GOLGA4	0.414599195895905	CEBPB	0.383474345042311
GLRX3.1	0.418374711269253	CYTOR.3	-0.699256970879314	WDR83OS	-0.472972736008394	FAT1.1	-0.579959864392465	SEM1	-0.267622414716547	RHOJ	0.383417478571632
CREB3L2	0.312157487819101	ANXA1.3	-0.799941552700305	NDUFB10.2	0.370384509130676	GDAP1	0.535884084850485	TSTD1.5	-0.50372355078266	IRF9	0.382616634933797
INPP5F.1	0.472447115790664	PPAT.2	0.468983520324763	CCT5.1	0.412084257903461	WEE1.1	-0.552894347651561	EFNA5.2	-0.255885299395675	EIF1	0.382087089680287
RAB34	0.452854203129472	RPS5.1	0.270137419808932	CTSV	0.291235751723226	TSTD1.4	0.501514291043606	TBC1D7	-0.281053150236349	TRIM22	0.381215494776629
CMTM3	0.38718052850535	CHEK1.1	0.451059556398575	GMNN.2	0.441177928314576	NDUFAF8	-0.590447518713718	TMEM258.2	-0.270233974063919	FAAP20	0.381063065589561
ANP32B.1	0.420864426662811	XRCC2	0.359450010543556	SLC2A3.2	0.455391942990635	C3orf14.1	0.550613882683224	UQCR11.2	-0.276629520346008	TPT1	0.379430529329063
GGH	0.419449370985662	RBMX.1	0.492238312390791	CAST.2	-0.612193640518079	SOX2.1	-0.548285123157022	VMP1.5	0.585511107584306	PMM1	0.377753928895193
ARL4A	0.517902360049005	CKAP2	0.541813918547464	BRK1	-0.470872109912519	NME4.1	-0.570775380474252	FAT1.2	0.412956749064433	IFNAR1	0.375513936635988
TSPO.1	-0.947004641895555	HIST1H2AG	0.343628382294344	PCOLCE2	0.346968921891566	PSAT1.2	-0.61974569067924	KIF1B.2	0.417949544603372	SPPL2A	0.375434890921909
MMP2.1	0.557951590699316	POLE3	0.474109216782327	CFL1.1	-0.440259679734007	MAP1LC3A.1	0.555095673710985	SEC11A.1	-0.282713861753368	SOX9	0.372433065495567
KLF6	0.471955866093507	PRC1	0.478485209708493	CHCHD2.2	-1.22265790853759	PRDX4	-0.495005293127151	VCAN.2	0.492002022773789	COPS9	0.370419823604206
HLA-C.1	-1.16356139121561	UBE2C.2	0.850438607983114	CCL2.2	-2.25380236412452	ANXA5.2	-0.523007210216811	OGA.1	0.375924920293475	EGFR	0.36932749499218
ZNF93	0.331745552799753	EMP3.2	-0.679856002212197	RAD51AP1.1	0.391895927481887	WDR47	0.411206523795105	JKAMP	-0.268904668278873	NR3C1	0.369018055979089
RASSF8	0.337018572502117	CENPW.3	0.437370350155462	MAGED2.1	-0.628110040150441	YBX3.2	-0.644576360620392	TMEM170A	-0.261320512920381	IFNGR2	0.36731070595036
FSCN1	0.607967017937551	UBE2H.3	-0.581847456066473	MAD2L2.2	0.48454851652888	WWTR1.3	-0.545887521744111	SCAMP2	-0.259903000435707	KDELR1	0.366899076512759
CAMK2D.1	0.431608684327964	CNTFR.1	0.345668627798291	CKAP4.2	-0.588127500409292	LGALS1.3	-1.84995755131159	MEG8	0.366975409080187	TEX264	0.365103037012319
RHEB	0.398173162406976	TOMM6.1	0.393314614259083	DNAJC15.2	0.445377938621299	EVA1B.4	-0.513285859890176	DSTN.3	-0.393723557873699	CDO1	0.363461104967326
KLF3	0.333952598322328	SSB.2	0.394250492195574	CNIH4.1	0.468972249129825	P4HB.3	-0.605349835762018	UQCRB.3	-0.280074053979377	WNT5A	0.363172586792473
IRS1	0.273268156540081	NDC80	0.41610369522227	KIFAP3.1	-0.592635533309777	CDH6.3	-0.897435576531174	C12orf65	-0.283612495885555	EPB41L3	0.36178906951189
NEK6.1	0.338378447833689	ACTL6A.1	0.438018939154553	FEZ1.1	-0.606179228357844	A2M.4	-0.895124825095693	N4BP2L2.1	0.486944000054289	DAB2	0.361680940020443
ZNF706	0.396571639771931	H3F3A.2	0.260415841762167	PRMT2.1	-0.540449920796186	OSTC.1	-0.433827293874967	ZNF32	-0.270000271401982	MYOF	0.360717434297576
DDAH1	0.303836804595983	MIS18A.2	0.407905704583685	HNRNPD.1	0.425897106703279	KIDINS220.2	0.90394936748921	SPCS1	-0.274385167937608	RIT1	0.360365012513096
AC093673.1	0.323598366913559	ARL4A.2	0.466285978770227	CHCHD3.1	0.446035144359684	GLIPR1.4	-0.648931482637196	CEP63	-0.263568125949865	FOSL2	0.358306605939563
CASP3.1	0.472775043872155	BZW2.2	0.507700497011482	ENC1.1	-0.79842363439538	TUBB6.1	-0.575212102457627	LAMP2.3	-0.250744952497328	PPIC	0.355338931929385
NECTIN3	0.374521262076057	SMC1A.1	0.512132947045938	SRSF2.1	0.456495096725531	HMGB1.3	-0.552743337653729	NFIA.4	0.619384935495806	SIL1	0.355075379566426
SINHCAF.1	0.426023120292256	TIMELESS	0.317948740411397	SSBP1.1	0.399303285867349	KTN1.3	-0.495917666376324	FBXO22.1	0.413724550646938	EMP1	0.354867597418059
CCDC50.1	0.49003749374034	BUB1B	0.400974201364588	SNRPA1.1	0.477196237235439	TUBB2B.2	0.985053150571518	TXNDC17.1	-0.257677852566569	KCNMA1	0.352133595969611
HSPA5	0.456452324615312	POLR3K.2	0.469565469504014	CRIP2.1	-0.561613385168173	P4HA1.1	-0.48456008154663	NAP1L1.2	-0.282306183010193	RPS26	0.351704190607962
CASP6	0.342129818344041	MCM6.2	0.483852313293038	HNRNPC.1	0.388102880324597	EIF4A2.2	0.68504941117211	IGFBP2.4	-0.610955274454033	TFPI	0.348842557284891
SPATS2L.1	0.543369542103751	SRPK1.2	0.470250783881	EIF1AY.2	0.405510843752843	MDK.3	0.444344546840737	SF1.1	0.375088621248733	NOS1AP	0.348477848608428
SC5D	0.439012861903677	MRPS6.1	0.427261579650472	EXOSC8.1	0.437044976255755	SLC3A2.2	-0.652631142438589	PEBP1.1	-0.292347555247396	MIR100HG	0.346951642230841

HADH	0.376545456473691	PPIL1.1	0.423960003176343	SMIM3	0.260649424701154	RPL28	-0.290990668213126	EXOSC5.2	-0.294133148905961	CHST3	0.345644776000156
LRRC42	0.320616137231572	RPSA.2	0.28039177691686	LSM6.1	0.436790878407953	PLPPR3.2	0.769910519363457	REEP3.1	-0.326235406627497	L3HYPDH	0.344644476921218
TYMS.1	0.502821196200874	NUDT1.2	0.429422974713867	ADSL.1	0.445101700630065	AFAP1	0.431994476017072	MED31	-0.267183091302349	NFIC	0.342539068734286
FGFBP3.1	0.526449309248416	RPS3.2	0.387101776239436	CARHSP1.1	0.441893414503289	PCNA.3	-0.55613492057427	SNRPG.4	-0.294972617228527	ODF2L	0.339302887112459
CKAP4.1	0.432420904136376	CDC7	0.257189942884534	HNRNPAB.2	0.43808891918634	PSMB10.2	-0.508327754475534	LUC7L3.2	0.498422517212708	RFTN1	0.338023482034426
LBR.1	0.416107309212852	RPL36A.1	0.275214056171764	NOP58.1	0.454777497945215	REXO2.3	-0.518305452774823	BRD2	0.428497000838233	SMIM29	0.337303003912373
ARPC1B	0.445772243307969	MYL6.2	-0.448158200355096	CTXN1	-0.522587470490592	SLC2A1.1	-0.663571115477145	SEZ6L2.3	-0.29550682461428	FAM89A	0.337057547382661
EFNB2.1	0.451704997910647	NQO1.3	-0.875595488620223	ALPL	0.277889878689649	CTNNAL1.1	-0.494225654130436	RHOA.2	-0.271777711215347	SCD5	0.328706055766577
HSP90B1	0.424532014415684	SERF2.1	-0.344800380706387	MXRA7.1	-0.563532172055488	SLC1A5	-0.477550481604243	STX2.1	-0.263842651479479	GSTM3	0.327596990825395
PCDH7	0.353168348795573	KIF2C	0.351326070966462	CDH6.2	-0.856654504972846	NR2F1.3	-0.407527809693512	XPO1.1	0.372724324918158	SLC22A23	0.32298902594723
CRIM1	0.340271905788229	FDPS.2	0.519376775662905	TRMT112	0.400901722493059	TOMM5.3	-0.52518900892435	RUFY3.3	0.288919412531727	FCGRT	0.322510686956377
RHOA	0.317982619022952	CDC45	0.285962589585167	MCM3.1	0.41954974563661	COL5A2.4	-0.524456970418885	MARCKSL1.4	-0.362024411431503	PRNP	0.320958051236498
MFGE8	0.396988812216823	RPL10A.2	0.283940008542154	SRGAP3.1	-0.620318566769086	PDPN.2	-0.536242969163624	KIF5C.5	0.316142701284707	POU3F3	0.31983993291797
PARVA.1	0.34216552718114	KLHL23.1	0.439538003411715	USP28	0.355199895100342	RGS5.4	-0.726701137782721	YBEY	-0.265581679060497	IRF2	0.319233423462947
PRKAG2	0.279394007139171	GINS4	0.340787807811003	MIS18BP1.1	0.404414655690439	NEK6.3	-0.43833854988732	PSMA2.2	-0.292968120120154	LINC02802	0.317647947278997
SLC4A7	0.365955829041531	MAGOH.2	0.451548313687636	COL4A1.2	-0.95767155394028	ATL1	0.488246865171172	NPM1.4	-0.450969129292449	CTSA	0.316282318558042
ENAH.1	0.45608961098796	DANCR.2	0.535325973770248	AURKA.1	0.422218977466307	HES1.1	-0.523196417528389	ARGLU1.2	0.504593146584402	AP2S1	0.314874707894624
SNHG17.1	0.372451009908382	SNRPA.2	0.437014627571394	ITGB1.2	-0.676284180415325	FAM171B	0.588608442595165	BDP1.1	0.444077641615543	P4HTM	0.302976572763756
PPP1R18	0.339352790032642	ITGB3BP	0.367193475941145	VAMP2	-0.638703670555713	DEK.3	-0.571320254368381	ZNF207	0.398180280267325	SERF2	0.301772843449365
GALNT1	0.441351916318751	CDK4	0.446741292914035	CPE.2	-0.961572679937298	EIF1B.1	0.465912934790688	A1BG.5	-0.311586401320919	IFIT5	0.300879253869031
DERA	0.339777497257716	EIF5A.3	0.36989531032938	MAGED1.1	-0.60126179930092	HLA-C.4	-1.18871252499949	FUOM	-0.267218829260253	ENDOD1	0.298974391294025
CDH8	0.313711423904838	SOX2-OT	0.346947718325177	SNRPE.1	0.387781598005731	SEC11A	-0.389236048585397	NAPG	-0.250044627149535	PIPOX	0.288088952182463
LAMB1	0.349464601101367	CD59.2	-0.727781599557729	SCARB2.1	-0.562018550781323	FAM155A	0.413627693459691	GLT8D1	-0.267251593028693	KIFC3	0.28652622824123
MT2A.1	-1.57084701321874	SELENOM.2	-0.625445171063372	DYNC1I2	-0.527172169686026	HMGN2.2	-0.436781609055186	SH3BGRL3.4	-0.487023335324738	AFF3	0.284954177427914
MTHFD1L	0.365082124517219	ALPL.1	0.263657407193611	DSP.1	0.317469289041346	MFAP4.1	0.586966112070379	TMEM256	-0.343275696946557	FNDC4	0.283411265423902
RAC1.1	0.325046604782245	NTRK2.3	-0.805261409696677	SSB.1	0.404127545140721	NFIB.4	-0.526217109170809	SSBP1.3	-0.266204683415414	BOC	0.276922632305685
CCT7.1	0.387511466267578	CMTM7.1	0.306439393586425	HELLS.2	0.427355684565255	RPS2.2	-0.485276264983105	RTRAF.3	-0.259651412739092	RPL24	-0.251575810901937
TMEM65	0.270646435094265	PJA2.2	-0.591494739487648	HMGB1.1	0.383383698890587	MAP1LC3B.2	0.506384763448422	RAB9A.1	-0.283627539179586	RPL15	-0.263291255289996
ARF4	0.395828104389752	IER3.3	-0.874816059153822	LINC00665.1	0.409891463780555	PRKAR2B.1	0.619846127392274	GCSH.4	-0.306303040028793	RPL22	-0.307598367921989
TMSB4X.1	0.328298809850783	TNFRSF12A.3	-0.72496682756284	PRSS23.2	-0.680242100855821	LYPD1.4	-0.600784509112955	PNN.2	0.391945679034422	RPL38	-0.316172706130379
PDIA4	0.404086678473306	DTX4	0.292638237997543	GPATCH4.1	0.455519101490763	NBEA	0.581440194612389	ZNF292.2	0.405730861216848	RPS17	-0.329983096974098
MAP1LC3A	0.289454497559044	TMEM158.3	-0.840021345785901	UBE2T.2	0.416483857051176	MYH9.4	-0.757198840173476	JAG1.4	0.2530433756899	COX7C	-0.331652726745187
ACAT2.1	0.498133947360365	GGCT	0.504652796146829	LSM3.1	0.418877003725752	RAB6B	0.469250330670002	DDX3X	0.350511905730938	NDUFS6	-0.353093138139861
UBE2D3	0.382703798072541	FKBP3.1	0.36983288978345	KDELR1.1	-0.451588212811991	VAT1.2	0.574680359405947	TNRC6B.1	0.375318980828678	BTF3	-0.364478096491609
FLNA.1	0.547893287778786	MXD4.2	-0.543342601744415	ACTR3.2	-0.527868743511232	LINC01578.1	0.49792605240582	CHD9.2	0.462525715943853	RPS23	-0.369777620603512
RAP2B	0.450414033576332	MAGOHB.2	0.424010687149806	PFN2	-0.420680349036643	TRAM1.1	-0.428973275831407	CD63.4	-0.490300062608347	SALL4	-0.375727831241021
RGS3.1	0.551361440717635	EPB41.1	0.321069582225409	MIR99AHG.1	-0.520685705399441	SFT2D1	-0.438292776239788	LARP1	0.338214312029066	HMGN1	-0.377637200404696
POU5F1.1	-1.1970549816125	TMEM59.3	-0.517588947030865	TIMP2.2	-0.784432337380732	CXADR.2	0.593963978284229	PPP1R11	-0.263665333426346	COX7A2	-0.38150770406234
FAM114A1.1	0.322987275626676	SUV39H2	0.286952254095314	PSAT1	0.471313462565017	TMEM123.2	-0.47023602542398	PSMB2.1	-0.262230692391853	NDUFS5	-0.384212182850458
TMCO3	0.31785622367059	CCT6A.2	0.45250034509459	USP9Y	0.292559694795821	MYL12A.4	-0.570187249227418	SNRPF.4	-0.378149579076297	ATP5PO	-0.386927549136288
PABPC1	0.377459444280068	AIF1L	0.3027481118648	A2M.2	-0.928365183743358	RPS17.3	-0.348689012746336	FLNA.4	0.414369529479577	RPSA	-0.387456669939158
SLC25A13.1	0.359283119268754	HDGF.1	0.579100218343689	FST.2	0.594905202331567	CCDC112	0.596114726218527	ATP5F1C.2	-0.274486352293071	THOC3	-0.38809435753584
PAM.1	0.309448251688167	SYNE2.1	0.471686340836978	ACOT13.1	0.420243226842435	EPS8.2	-0.48836498597149	NDUFAB1.2	-0.250244514120998	NAP1L1	-0.38914330652005
BZW2.1	0.347098873475485	FAM136A.2	0.480113061736224	LAPTM4B.1	0.420451361136173	COL18A1.2	-0.479558663905636	TRIM28.3	0.369656265812224	NUSAP1	-0.400321982794618
DAPK3	0.331416734341957	SEPTIN10.1	0.401756227979929	MRPL15.1	0.417811675321339	RPL8.1	-0.335612366661612	FGFR1.1	0.300254560457786	ATP5MC2	-0.400757336610238
CREM	0.351304520459899	UNG.2	0.395108027867009	HLA-B.2	-1.52996762967825	ALYREF.4	-0.488262506320096	C11orf74	-0.256573821676233	ACVR2B	-0.405333413517318
LIX1	0.293614296318933	BRCA2	0.336974538043371	DAD1.1	-0.448211952019024	CALM1.1	0.461712480037696	PHIP	0.353113389095056	CMTM7	-0.411583174046852
SERTAD2	0.268929866111688	TET1.2	0.419295444071711	MSN.1	-0.66878959879779	PODXL.1	-0.487508837218258	EIF4A2.3	0.356583454602591	XRCC5	-0.41264354843354
YAP1	0.284756101499284	RPL35.1	0.304830120947775	KTN1.2	-0.463776012853871	YWHAG.1	0.632290352132398	ACTN4.2	0.253077407303091	MND1	-0.413815106216693
FZD2	0.309735944173746	PTPRZ1	0.421989018289558	RAB2A.1	-0.47105514128934	ANP32B.3	-0.548374810538806	P4HA1.2	-0.323795461476358	SFRP2	-0.418957968447109
NUTF2	0.34562330732771	HIST1H1E	0.60810909197953	MCM5	0.363300587464323	CLASP2	0.552163656374227	ACTR6	-0.261001264216924	LSM7	-0.424478247011925
DLL3.1	-1.65027706504056	OST4.2	-0.467273713688176	ATP2B1.2	-0.667467323721173	KIAA0408.1	0.566904637728859	MDP1	-0.287874477166785	TOMM6	-0.424931681719344
SLC2A3.1	0.529209848552695	TNC.2	-0.646138982474174	BRIX1.1	0.41695844489599	ZFP36L2	-0.440316085284757	SLIRP.2	-0.311387520227109	LSM3	-0.425161334291095
TUBA1B.1	0.452927770837296	SAT1.3	-0.794223295551054	PARP1.1	0.450990424390629	LIX1.3	-0.623242433903043	CTNNB1.1	0.319615327861604	RPL14	-0.42594435885108
PLAT	0.267028853337018	HNRNPK.1	0.399222414856074	BAD.1	-0.494592014187922	KLHL24	0.399072309688084	KDELR1.3	-0.29813599000017	TOMM20	-0.430755151922875
MT-ND5.1	-0.774803926128563	PARP1.2	0.620018683782618	H2AFY.1	-0.458849683148486	CACNA2D1	0.531254397857661	POLR2I.2	-0.256577193102157	DHX36	-0.432777361398285
NOTCH2	0.2695528135434	UBE2S.3	0.529026598020676	ACAA2.1	0.403851113037913	BLVRB.2	-0.502015884525147	SFRP2.4	-0.321596116506817	XRN2	-0.433573874091674
CKS1B.1	0.523153162409306	ISG15.3	-1.50130571275327	EXOSC5.1	0.362828381210277	MAML2.2	-0.461339808258785	NEFL.5	-0.342102759495629	ERH	-0.434257318721341
PLS3.1	0.406904062239264	CCT4.2	0.422764756900858	GCSH.1	0.394832640587734	JAG1.3	-0.545025139567141	HSPE1.4	-0.347878808949833	IGF2BP3	-0.437023462340646
EPHA2	0.296681538960459	NONO.1	0.462732222547322	APLP1	-0.651875917163023	PON2.3	-0.599320997698946	SNRNP70.2	0.368543524970114	ATP5MC1	-0.443606654838437
DPYSL5	0.31910368793609	CSE1L.2	0.461027020397574	RRM2.1	0.385329901868678	IKBIP.1	-0.5021030554172	NTAN1.1	-0.278288035620053	MIS18A	-0.44948659575383
VDAC2.1	0.322861965720496	BUB1	0.336862283679594	ESD	-0.461166194543372	IQGAP1.2	-0.47216460163729	PDIA6.3	-0.322946600537536	SNRPC	-0.449802661980244
REXO2	0.395416411990241	KIF4A	0.368246238406859	PAK1IP1.1	0.417259003587361	ATXN7L3B.2	0.557735346753972	CHD2.1	0.355643536865783	NUDT1	-0.451402011023327
FKBP5	0.289287951369029	PSMG1.1	0.450352606032688	PLK1.1	0.423498307679137	PDIA4.1	-0.518638184828789	ALKBH7.1	-0.309647716224001	BIRC5	-0.451792545793287
CFLAR	0.369252046111299	IFITM2.3	-2.11901615196727	CENPW.2	0.401612289034044	BAZ2B.1	0.632144943495161	TLE4.2	0.498241091584303	PPIA	-0.453268207223145
DPH3	0.352438457750386	KIF20B.2	0.458091508097242	SEPTIN7.1	-0.417905896921965	FST.4	-0.774580046628645	SS18L2.1	-0.303846622836072	CCT4	-0.453403341653427
GNPDA1	0.307422071631083	ECT2.2	0.400221275871691	WDHD1	0.328784218157976	DYNC1H1.1	0.674655698656087	SOX11.3	0.312161294004196	CALM1	-0.457045993216283
DDT	-0.39993186110026	COX5A.2	0.315015599217242	MAML2.1	-0.490956112772976	MARCKS.2	0.465424328585198	TUBA1B.4	-0.43152564356937	SF3A3	-0.457476435071106
SH3BP4	0.315188192113904	TFAM.2	0.428414400298894	GNAI2.1	-0.501184214757117	LINC01315.1	0.419163304420146	CFL1.2	-0.318005932935242	TSPAN18	-0.458251730942616
GOLT1B	0.28310531445429	RHOC.3	-0.648743728726979	CD3EAP.1	0.365328502060328	CNN2.1	-0.592918297488023	RANBP1.5	-0.499482145747326	TCP1	-0.46027261913685
IER5L	0.38035228016691	FDFT1.2	0.44324076566192	PDLIM7.1	-0.551266746542657	MAP4K4.2	0.570415079342879	PDLIM4.5	-0.495849335979812	G3BP2	-0.460628072084811
RRBP1.1	0.499862405507559	FAM111B	0.297368223515558	HNRNPA1.1	0.382669060630813	NDUFA5.1	0.539088400231462	IGF2BP2.1	0.324251327646994	SSBP1	-0.461451506441322
CPE.1	0.655310167085016	BTF3.2	0.286742978445805	HIST1H4C.1	0.622046676634165	HSPA5.1	-0.534274120250021	SNRPD1.4	-0.362357905849416	USP44	-0.466315318448924
PHACTR2	0.423712571951528	PLK1.2	0.502638722117347	UBA52	0.274982375569937	PRDX5.4	0.468374947956336	HMGN3.2	-0.30892617792569	NDUFAB1	-0.467033562096832
MYOF.1	0.277716926374049	DDX46.2	0.419437240675915	ANLN	0.401830679508317	NR2F2.3	-0.337628456264096	GADD45A.3	0.312567579846315	LSM4	-0.467237429852798
GLI3	0.290493181452898	BAALC.3	-0.716169029977896	DSG2	0.37944249608596	RAB31.4	-0.4908777691846	TAF1D	0.404594655788073	PRKDC	-0.46785916597058
UACA	0.523786385095817	ESCO2	0.31592665503727	NUSAP1.1	0.334092233288845	ETFB	0.515605416704031	TEAD1.2	0.283735540075546	CCNA2	-0.472312224907591
HDAC7	0.257127296754607	IFI27L2.2	-0.558688873486617	TRAPPC1	-0.438398768831804	SERPINB6.3	-0.459319762872061	SYPL1.2	-0.275577534537382	IDH1	-0.473698235876786
PLEKHA8	0.276104522461386	PLP1	0.254954554183328	CCT6A.1	0.364385216104117	GPC4.1	-0.484426057891103	UBE2D1.1	-0.257937513873202	VDAC2	-0.47377663014925
COL18A1.1	0.487523504349293	GLIPR2.1	-0.503278640120145	RPS27	0.276386662035568	TFPI2.1	-0.787290609913362	KDM5B.2	0.334193071119864	TFAM	-0.477575557580169
FAM107B	0.340464056686368	GPATCH4.2	0.587441268669954	IDS.1	-0.560201194349121	PAWR.2	-0.491616879375694	GCC2	0.316715360736931	CNBP	-0.478943527584322
PURPL.1	0.307106996157199	DCLK1.3	-0.587897302752389	TERF2IP.1	-0.618268337165425	FMC1	0.533042769919989	ATP5PD.1	-0.254592550444689	EEF1B2	-0.479193896262767
TFPT	0.259021222789295	DPYSL5.2	0.34681979305448	NUCKS1.1	0.334884849742417	STMN3	0.509179903559118	TOP2B.1	0.280819494633941	SNRPB2	-0.479234122126116
JAM3	0.3077862019234	TOMM7.2	-0.404404827036216	NR2F2.2	-0.615658574479309	TMBIM6.2	-0.421857797517348	UBA1	0.2865726371952	RBM8A	-0.480023325037765
TMEM123	0.40615223141297	KNL1	0.319847354713243	TUBB3.2	-0.861696825359467	CLIC1.3	-0.511013682800666	RBFOX2.3	0.286463602797907	SIVA1	-0.48026564199443
ARPC2.1	0.406323341700707	BLVRB.1	-0.502665661690827	CD44.1	-0.709642667787364	BICD1	0.489310020910601	MAP4K4.3	0.330098218436768	DBF4	-0.480411765108771
FDPS.1	0.432770339246463	SRSF9	0.340826668368507	PHPT1.1	-0.414504725839446	DNPH1	-0.450500278746292	MYH10.3	0.278692958973812	MIS18BP1	-0.482437694955904
HLA-B.1	-1.34440374497396	DDX18.1	0.379506278680112	SARAF.1	-0.527096819735552	SHROOM3.1	-0.433549626168376	TTC3.4	0.324672666711099	FDFT1	-0.484137885321374
CLSPN.1	0.484805749092414	HSPB1.3	-0.601355116246454	NDUFAF4.1	0.408891211604837	TBCB.1	0.452080864107952	OSTC.2	-0.255600168566929	CCT7	-0.484867792884217
RTN4	0.391024182710904	TTK	0.313756656741036	CDC6.1	0.341425323117267	CKS1B.4	-0.636832997415788	CYBA.4	-0.503213929638748	PPP1CC	-0.487000603307876
SSR3.1	0.391671282115328	SGO2	0.461773275127595	ARMCX3	-0.54494156376959	MTHFD1L.2	-0.423601223428565	SPP1.3	-0.584535995933924	KLHL23	-0.489762298243625
H2AFZ.1	0.427171965624401	CDKN3.1	0.406191502371383	COL5A2.2	-0.512407714656007	CDKN2A.3	-0.737950153727613	NUFIP2	0.333188711450656	CNTFR	-0.492017133586621
N4BP2	0.323241640214371	USP44.2	0.268968586426913	GALNT7	0.390140193657749	PSME1.4	-0.670190995944093	MALAT1.3	1.27725401411305	HNRNPDL	-0.494821608677865
FAM126A	0.314873965259048	IL32.2	-1.22730122817462	EXOSC9.1	0.374257812963661	AASDHPPT	0.54759881383205	COL11A1.5	0.277332781693534	RRM2	-0.496217403108847
RCN1.1	0.387209145628646	CTPS1.1	0.372756183445145	CCT8.1	0.382071715297627	DAAM1.3	0.619961819908289	SCAF11	0.292330034574693	EIF4A1	-0.497961352962608
NIN	0.325498066850718	DYNLT1.2	-0.468901349914112	GCHFR	0.27745152261109	COX5A.3	0.362374073695757	ATP5MC1.2	-0.300880223954013	HMGB1	-0.49894691422911
ROCK1	0.412576105434732	C1QBP.2	0.431185087251196	DDX46.1	0.395140383190388	CYP51A1	0.547431738853078	TNPO1	0.265600999667774	ILF2	-0.502472735918291
IFNGR1	0.256556419443497	PPP1CC.2	0.417958336234385	RPS13	0.285945984429642	TUBA1C.2	-0.81966785280921	BPTF	0.37113786203461	SNRPD2	-0.50355026832786
SEPTIN9.1	0.361618172353777	NFIA.2	-0.532288449037981	EGFL7	0.297498788980342	ISG15.4	-1.51844627299786	STMN1.4	-0.632650498036689	LSM6	-0.503803895490775
ID2	-0.580031795755989	NUP107	0.36597111523932	OPTN.1	-0.641965600843353	BIRC5.2	-0.518392554465444	COMMD3.2	-0.268193227874203	DDX39B	-0.50536937275983
BZW1	0.335337042964663	ILF3.1	0.458958716431087	PLD3.1	-0.64351131239897	SEPTIN2.3	-0.443719653888189	JMJD1C	0.343157537907643	SUMO2	-0.506298232634106
TXNDC5	0.319820208660191	FZD2.1	0.346116204582111	IMPA2	0.28999615398977	HLA-B.4	-1.43302104220736	XRCC5.3	-0.286908867376015	PRMT1	-0.506674108005954
RRAS2.1	0.342812752378355	ID4.3	0.435415194176934	H3F3A.1	0.254069053456038	PRRT2	0.345187175530327	PKIA.3	-0.317323706066352	ATP5MC3	-0.508942960986067
HSPB11	0.412439196463993	TAPBP.2	-0.547277672969995	RPS20	0.297897018814814	DCTN1	0.600906364594463	FUS.3	0.374156415588857	KNOP1	-0.510001265497771
CEP135	0.270696682777216	SRSF10.1	0.395143936792577	MDH1.1	0.399752945573068	DYNC1I2.2	0.499042650747489	ZNF462.2	0.266908031764994	CCT6A	-0.511802641090376
SDSL	0.323581175902051	MCM2	0.330350654458813	GNL3.1	0.400332291780249	SSBP3.1	0.528988039036204	NUCKS1.4	-0.267508478609012	UBE2I	-0.512454152342928
ZNF561	0.265120743187575	RPL12.1	0.256162735781832	SMDT1.2	-0.558077551906799	IFITM2.4	-2.03014360142832	FTX.1	0.637624765432628	GINS2	-0.512984258208381
CYB5B	0.346072601348678	A2M.3	-0.857647844672828	TET1.1	0.391990611902821	PLTP.4	-0.635506040506295	CCNI.2	-0.31479116967549	RBPMS	-0.517057552697118
DDA1	0.391588748806078	RRAS.3	-0.538116503043037	GAPDH	0.333460485591035	FGD4	0.39297423836294	RBM25.2	0.352484007466919	LAPTM4B	-0.518166642728046
PSME1.1	-0.628526818947693	MSN.2	-0.589144348815978	MRPS12	0.437505304969912	TMEM179B.2	-0.4373201057765	TUBB.3	-0.277763502262836	GABRB3	-0.519357133354124
NRAS	0.311304367521061	TMEM258.1	0.319580804172648	LMNA.1	-0.782521045651561	COX7C.3	0.299844484049391	MSI2.1	0.382214061155564	DDX46	-0.520496341997313
LMCD1	0.346095527724019	ARHGAP29.2	-0.707405449364958	EMG1	0.408326796465681	PLAGL1.3	-0.481756730346211	CHD7.3	0.276355492167946	NDUFA4	-0.521127014811107
A1BG.1	-0.460148325822435	LIN28B.1	0.316301784640534	S100A11.2	-1.46878904772534	TYMS.3	-0.572145811192522	TMEM223	-0.252252799757519	SUPT16H	-0.521191416362749
PPFIBP1	0.304473165999269	DSCC1	0.287814593511444	PAK2	-0.466821501364515	TRIP6.1	-0.424828369488261	DHPS.1	-0.265605907479274	AKR1B1	-0.521283814015056
RANBP1.1	0.359362985822567	KIFAP3.2	-0.515604812241786	NEAT1.1	-1.40348026899199	FKBP10.1	-0.457284823114125	Jun 05	0.321301984304874	MRPL11	-0.522265560700756
CTNNB1	0.466551609339702	SAE1.2	0.434066459945751	PTGES3.1	0.332319536430057	CSRNP3	0.421882995301654	KIDINS220.3	0.290574278122631	HNRNPH1	-0.522523430243006
SAE1.1	0.355245199676145	CDCA4	0.317795998206632	ZNF814	0.287147912069652	LPP.4	-0.55272780758243	LAPTM4A.2	-0.355361743121492	KCNQ2	-0.52338514644866
USB1	0.306905436955039	COL4A1.3	-0.813211621518411	ZIC3	0.261067344287985	GPATCH4.3	-0.532486748405052	UCHL3	-0.290479902182976	PIMREG	-0.523766041173709
OSTC	0.311598157858995	CEP55.1	0.33358358188585	PALLD.2	-0.657834519262361	MRPL11.3	-0.505610662633807	ARHGAP5.1	0.314427381150645	KHDRBS1	-0.524184726595037
YPEL5	0.362340099673497	RCC1	0.295238340631933	CDKN3	0.321326464915853	MSN.3	-0.598663276830062	ACTA2.5	-0.687872014742768	CCT3	-0.528219453144243
SNRPB.1	0.349078587503903	CBX1.1	0.384299799204892	NDUFS5.1	0.290510159868326	VASH2.2	0.48357505802721	CDC42.4	-0.255924213493886	TET1	-0.528360934318939
IGF2BP2	0.395962101419341	CDCA2	0.34711954904925	TMA16.1	0.377481060742951	RPS24.1	-0.286738730253552	H2AFV.2	-0.28743615293849	NUCKS1	-0.529241935263203
BCL7C.1	0.352257254783519	CDC6.2	0.370300863530451	NOP16	0.371429761195368	PAFAH1B3.2	0.80241895636929	TIMM9	-0.259096769524865	TUBA1B	-0.530059350523108
HIGD1A	0.372609657955422	TCERG1.1	0.451575032621678	TFAM.1	0.387708808492526	PTPRG	0.457048507897965	DERA.2	-0.258665589133973	SNRPA1	-0.530810597776678
GNB4	0.320871915012181	CCT8.2	0.36826328647494	MTDH.1	-0.437453585017879	RPS27A.1	-0.291619106720881	TUBA1A.3	-0.631934359860435	NOP58	-0.531718403942153
YBX3	0.448261750469802	ARHGAP11A	0.311019011542602	GPN3.1	0.365730002803172	S100A16.3	-0.581941478387678	TMEM9B.2	-0.267870322294455	EXOSC8	-0.532267005000638
PSMC3IP	0.265979695696746	SNRNP40.2	0.38947432283685	CMSS1.1	0.380002070527454	UACA.1	-0.598984712862694	NDUFA6.2	-0.254057570720424	POLR3K	-0.532310153235767
LYPD1.1	0.346859740360637	RSL1D1.2	0.491287308821171	DCP2	0.386300250868765	BCL2L12.4	-0.440350879803698	PRPF4B	0.319535335751883	EIF5A	-0.533808764442177
TOMM5.1	0.287885618268963	SPATS2L.2	-0.480174969717627	SOCS2	0.318618351984261	DCAF13.1	-0.411799898495806	PXMP2	-0.273585844425699	COA1	-0.536147771963538
CTTNBP2NL	0.335483789347099	GLRX.1	-0.513620609475592	SRSF3.1	0.369825563356494	RPL7	-0.334643883508822	ARID4B	0.274905801879034	FAM136A	-0.538079531457671
SLC7A5	0.383089240647202	CFDP1	0.397997271717162	AURKAIP1.1	0.376630352119869	MALAT1.2	0.424901317378094	SNHG8.1	0.340707135419408	EZH2	-0.539446475551286
COX7C.1	-0.298190927752139	LSM2.1	0.36094322388307	FBL.1	0.444214808662836	LINC00461.2	-0.49229758008993	MAP1B.4	0.347913287629847	PTGES3	-0.539560920979538
SOX9.1	0.312124538959625	POU5F1.3	-1.25185816143455	CAMK2N1.2	-0.885938212334503	CHD4.1	0.522096176358239	SPAG9.2	0.26888257777627	CDC20	-0.540342455804261
CAPZB	0.310982277707678	SSRP1.1	0.614187338586371	PIMREG.1	0.391949260449106	MRPL17	-0.424446051348723	FDX1	-0.268722364851509	MCM3	-0.544582956668037
TWISTNB.1	0.372077281899622	TUBB.1	0.308545397839086	CECR2.1	0.357382174858207	FGFBP3.4	-0.592440773393309	LSM4.2	-0.250331502263491	HNRNPH3	-0.545369197017342
SERPING1	0.260640697903747	AMOT	0.267216071208309	DKC1.1	0.396161822465256	HELLS.4	-0.528338811344116	MRPL58	-0.257500243660315	DDX21	-0.545918747613227
CMTM6	0.327050385279864	HIST2H2AC	0.374195864041072	HNRNPA2B1.1	0.382944777066756	CD200	0.375817192906426	AFF4.1	0.338355521153502	SNRPG	-0.547527986611818
ADD3	0.363706695881448	NME4	0.484729569910751	MYO10	0.346104762043236	BTG3.1	-0.427152378595621	PCCA-DT.1	-0.252556106052208	VRK1	-0.548155512055416
OSBPL8	0.374193207887739	PNN.1	0.478232463167614	DCUN1D5.1	0.386377852485136	LAPTM4A.1	0.435876234608406	ANXA5.3	-0.408406392128418	HMMR	-0.548496075202158
ELK3.1	0.258120302159824	DCXR.1	0.385501427014275	HLA-C.2	-1.19793822292794	LMNA.3	-0.791176758593041	RCAN1.4	-0.477248728669168	RBMX	-0.549401234442571
TJP1	0.384938375697291	SVIL.2	-0.61877929927448	RPLP0.1	0.28638032499461	APC.1	0.657895574468264	TBCB.2	-0.263918226735489	TMPO	-0.549966720756366
KIF5B	0.366040651611963	TCEAL3.3	-0.494577350997913	CCNB2.1	0.284209031063282	FNDC3B.3	-0.435973417136584	ADAR	0.27647033945395	PNN	-0.553668083744269
CELF2	0.372091672842736	PSME1.3	-0.652477880323733	TOMM7.1	0.362573631832064	AKAP6	0.439253038390415	FAM133B.2	0.281815352158874	CLSPN	-0.556153892696635
AHNAK.1	0.438757616436594	FKBP5.1	0.331164652407802	EID1	-0.387071381080561	ECI2.1	-0.46112372154388	RAD51C.3	-0.28756384301433	TOP2A	-0.557014333239218
NTRK2.1	-0.706276841621242	MT2A.3	-1.74867553549645	TUBB2A.1	0.596364751103419	RHOA.1	-0.356191739594826	ODC1.3	-0.286335378649565	QPRT	-0.557039844193769
HMGN1.1	0.259970547094698	FADS1.2	0.631255233685073	TOMM22.1	0.382274479246446	NR6A1.2	0.440314434822104	DHX9.2	0.277356137779182	PFDN4	-0.557204878089838
KLF7	0.313448075193799	CEP78.1	0.365009743797087	TSPAN3.1	-0.447214583335231	SNRPN.2	0.395784968601848	ZFAND5	0.287421965258935	JPT1	-0.557245980844427
PEA15.1	0.34628000133353	SASS6	0.272023341085644	LYPLA1.1	0.385021689334506	SPP1.2	-1.32858566803896	ATF4	0.290835614520869	RND2	-0.560876477593826
PTP4A2	0.336709433677171	IDS.2	-0.49005482693725	PTX3.2	-0.873444754570631	ID1.3	-0.730301046032209	EIF3B	0.303078393723015	LSM5	-0.561873731481634
ACTR3.1	0.356935617680661	COA1.2	0.379821179217656	AURKB.1	0.364390800740321	NUSAP1.3	-0.479243919073134	MIAT.5	0.628949804142136	CCT2	-0.565319789830065
ZEB2	0.315904015809254	ENY2.1	0.324845377336765	CPVL	0.33653871310713	MYL9.3	-1.1117088752796	HNRNPH1.2	0.311791415402346	SPINT2	-0.56739433684875
C7orf50	0.340420119213706	MMS22L	0.280016402389154	TNC.1	-0.650018949010309	SERINC1.2	0.63395528203752	HUWE1	0.266102977515471	CCNB2	-0.571399821002929
TGIF1	0.365597832031177	PPIH	0.370745338660534	HNRNPA3.2	0.374677449949063	PROX1	0.383289099413716	GTF2I.2	0.301939346116004	NOP56	-0.571458591076422
CFL1	0.303166253000567	NOP58.2	0.469163668036833	EIF5A.2	0.359027865232207	GMNN.4	-0.493516800582409	DDIT4	-0.263060796625083	FBL	-0.572308136108734
GJC1	0.274540947608982	EIF1.2	-0.403523501005535	ARL2BP.1	-0.489129278046082	WASF1.1	0.470776371062439	DDT.3	-0.251408822502974	TUBB	-0.573025012884061
RBMS1	0.405552419991655	SKA3	0.260312492895423	CENPU.1	0.34605848543209	YAP1.1	-0.344500990268895	YME1L1	0.267884271631668	PARP1	-0.581019710022708
MT-CO1	-0.593927952496222	SERINC1.1	-0.519802935529095	SLC29A1	0.269151379413838	GINS2.4	-0.488745843937401	USP22.1	0.278369291856801	CCNB1IP1	-0.5822988929314
PCYOX1	0.280414950119888	NHP2.2	0.3618841699878	POU3F2.2	-0.604842942548109	FGFR1	-0.441684205614433	RNASEK.4	-0.304774339302451	TMEM97	-0.583228695999579
GADD45A	0.427271861815131	CDT1	0.318742909348016	PHGDH.1	0.397416698815434	LAMC1.3	-0.556053350659394	CKLF.2	-0.302181086686816	HDAC2	-0.585693112797097
UFD1	0.336241219951559	PDLIM4.3	-0.649188773252248	COMMD3.1	-0.421808250698514	BUD23.1	-0.420375371007014	IER5L.2	0.288675337434651	LYPLA1	-0.587599857042671
CDK2AP2.1	0.370684167440543	NUDT15.2	0.344057858398854	ZNF706.1	-0.378105595537454	CANX.2	-0.473602441360375	MLF1.3	-0.254093836494294	CCNB1	-0.588216463575028
ALYREF.1	0.326721478629553	XRCC6.2	0.567231154917444	GYPC.1	-0.605296049792675	CDK2AP2.2	-0.441955010080077	PSIP1.3	-0.321267658099202	LMNB1	-0.589083608191874
ERGIC1	0.285270402137573	GNG4.1	0.335431423131544	C12orf57.1	-0.457471538555737	NDRG4	0.403657828114093	TMEM158.5	-0.268881710058625	HNRNPC	-0.591966216182714
SIVA1.1	0.32090874714837	NIFK.2	0.404131435883175	TCEAL4.2	-0.522315544828499	NOC3L	-0.398608542184103	IL32.4	-0.557700144889235	NAE1	-0.593120274324375
HES4	0.37799955460958	CDCA5	0.336230145831096	KPNA2.2	0.471179576128595	SMC4.1	-0.464929402060997	TRIM44.2	0.251185925108578	ATP5F1B	-0.593728295515871
BSG	0.367197129367571	ZNF724	0.271791900900232	CALB1.1	0.313890388961721	FBN2.1	-0.376941551856459	ITM2B.2	-0.36209581554692	CHD7	-0.594118445413523
APMAP	0.302307210948033	PARPBP	0.252912717476779	ISOC2	0.413296104653479	JAM2.2	-0.465029457241712	AAK1	0.300009387107764	SMC3	-0.59765592749597
IRF2BP2	0.304095398600093	CCNB1IP1.1	0.362685319817523	HSPA9.1	0.398866856320339	CNTFR.2	0.432854264557315	POLR2J3	0.50718085717812	CXADR	-0.602864907228855
RECQL	0.310757420630367	STMN2.3	-2.20056665390392	PSMB1	0.315941906704722	CCNB1.3	-0.594961585395953	DKK3.4	-0.277468930042207	LIN28B	-0.604279833957595
PFKP.1	0.416686304432191	CHD7.1	0.431278869785239	CD151.1	-0.571716075658418	PEA15.4	-0.55385838394835	SMARCA4.2	0.29649005841134	CCND2	-0.604779688085433
VDAC1	0.275279337244014	EEF1D	0.361586991285323	LDHB.1	0.342835644440924	CIRBP.1	0.450845758356706	SRSF1.2	0.295861871238335	NR6A1	-0.605309404147297
DNAJC21	0.34105008285271	TCEAL4.3	-0.501773479462558	ATRX	-0.543839434483315	RTN2	0.327234208917186	PLAAT3.3	-0.282351466186574	EPB41	-0.607538748799131
STARD3NL	0.320888394905712	RAB13.3	-0.479473840674411	SRSF7.1	0.408071281202968	CTNND2	0.470446228938086	PABPC4.4	0.250756610827362	PPA1	-0.607714297784261
CAST.1	0.434023181096051	LSM3.2	0.328066614830467	PRXL2A.1	-0.510167903633293	ACAP3	0.326471861914702	CYB5A	-0.280461953946076	CEBPZ	-0.61205149929789
IFITM2.1	-1.94426080899943	CENPA	0.300298800798117	RNF19A.2	-0.655980900889943	IFT22.2	-0.387619737592264	RB1CC1	0.257743750249837	NME1	-0.615794508558003
ARF5	0.323709518624817	A1BG.3	-0.474197022230257	LAMTOR5	-0.408265451491232	CD59.3	-0.645121064135929	EVL.2	0.271854920971352	PA2G4	-0.616488783885439
CENPW.1	0.370478933762141	EIF1AX.2	0.458942418605689	RPF2.1	0.384313730215647	RCN2	0.387092569635396	RPL39L.1	-0.276540398039531	DEK	-0.619155241803977
REEP3	0.301472344070111	PHLDA2.3	-0.700287367675691	ACTG1.1	-0.452836803247262	SRGAP3.2	0.575938865291035	TUT4	0.328231351464238	TRIM71	-0.621023516185554

JUNB.1	0.302113193285398	U2SURP.1	0.412168357436324	ETV4	0.327403806969335	PA2G4.3	-0.467522474085353	PLCG2.3	2.15594374399565	CENPH	-0.623578653082691
TXNDC12	0.320045985056235	EIF4EBP1	0.447874649193704	MTPN	-0.409131344081978	HEBP1.1	-0.355118142249717	FRMD4A.3	0.267611325940527	POLR3G	-0.624059357205087
PMP22.1	-1.09185781065543	UBA2.1	0.398616092782585	CIRBP	-0.441183914107224	KRT18.4	-1.57848403660866	MCL1.1	0.251734531638339	HNRNPU	-0.625366041219249
SMG6	0.250026090103245	ATP6V0B.1	-0.456564726333446	SRP14	-0.302424510501796	FTH1.4	-1.10525921613753	CSRP2.4	-0.398986196241824	SRPK1	-0.627172846281961
SS18	0.284308860639151	PLD3.2	-0.588714500700576	RCAN1.1	-0.687832721052273	GCSH.3	-0.408864671368059	RIF1.3	0.270872529502829	SERBP1	-0.630304451712981
FAM210B	0.385582527794736	MSH2.1	0.400541096057514	ARHGAP29.1	-0.727007226958634	CENPF.3	-0.698285846815246	NOP56.4	0.261280623560928	ORC6	-0.63078180998918
ATP6V1F.1	-0.475947785441	MRTO4.2	0.463610002079546	PEA15.2	-0.52819942208914	RPL27	-0.303894418951818	CCL2.5	-1.25744687766075	FUS	-0.63394892983842
SWAP70	0.255190491708991	CTSB.2	-0.499092637210217	ATP6V1F.2	-0.469329358094604	TMED9.3	-0.459306821882777	CLIC1.4	-0.306554632655327	FKBP4	-0.634995495079479
SEPTIN11.1	0.357960245122549	CCDC80.3	-0.610677457525922	LINC01315	0.270102948268229	ITGAV.4	-0.564006946383814	MYL12B.4	-0.250439686884032	YWHAH	-0.642044073130192
RBM3	0.271516600911557	KIF14	0.30469689101623	COL11A1.2	-0.659215961895495	NMB.2	-0.603626447311044	CDKN3.3	-0.376816469683214	CCT5	-0.644198211359077
SHC1.1	0.421456833068455	BCL2L12.3	0.361377647969495	TMEM64	0.25515350201292	TSPAN13.1	0.503656855625354	TRA2B.2	0.310673944093644	CKS2	-0.644430135301591
ASCC3	0.429797741941619	TPRKB.1	0.36742616352448	RCC2	0.370475117761626	PHLDA2.4	-0.769974424880113	STAT3	0.268184639246782	GMNN	-0.645739527934887
PAWR	0.367612494170158	SLF1	0.286401771818062	UBXN4.1	-0.429803574913227	ENO2	0.571440361447225	AUTS2.1	0.312974590020048	LDHB	-0.650685032414684
TOPBP1	0.25750736394172	TRIM59	0.37467343826247	PRTG.2	-0.649904748687775	JUND.1	0.549528568348595	SNHG25	0.581401184015832	UQCRH	-0.653261228898559
JADE1	0.302487714751283	CLU.3	-0.744235442445975	LYPD1.2	-0.62655419383277	IFI6.4	-0.892249081391074	THUMPD3-AS1.1	0.332804250408333	MARCKSL1	-0.655471296229931
XIST.1	-0.580837004475886	CIP2A	0.294075471006392	HES6.2	0.634021627155272	ARL4A.3	-0.494162865880475	BAZ2B.2	0.36987043339927	INPP5F	-0.655719707624179
PRKDC.1	0.299311183432721	REEP5.1	-0.398030506599529	ATP5F1B.1	0.385712072762514	CCDC34.2	-0.429109177546522	AHNAK.5	0.250751123568427	C1QBP	-0.660174398474192
FTH1.1	-1.05530674444656	SEC61G	-0.368762749575508	GSTO1.2	0.419423847000766	RER1.1	-0.405825047843155	PRPF38B	0.269641308084399	CYCS	-0.667272368340433
RSU1.1	0.271450577626335	TUBB4B.2	0.569481166057763	SUMO1.1	0.322249594372698	BCL7C.3	-0.432588977368541	RPS27L.1	-0.257098439524642	SMARCC1	-0.669378791949389
SEPTIN2.1	0.29047674763255	NRP2.2	-0.798483590023386	H2AFJ	0.38149573552365	GNAI1	0.429838165187419	ZKSCAN1.1	0.256015188105226	HNRNPM	-0.672001659311133
EML4	0.2793752979349	PDIA6.1	0.367172693464849	BEX1.1	-0.753536545227927	L1CAM.1	0.350770698582324	CALM2.3	-0.322395347862231	NCL	-0.673362897646722
ANXA6.1	0.314779860547908	HIST1H3G	0.326621292124163	TMCO1.1	-0.429536425427517	TAPBP.3	-0.515506728865025	NTRK2.5	-0.416547393293763	SNRPN	-0.677733899730074
LIMA1.1	0.342776966938077	RNF130	0.378566693943503	ORC6.2	0.341658870895829	BUD31	-0.372573198084263	RPA3.4	-0.387299797715934	HSP90AA1	-0.682088242472425
CDK1.1	0.426564680782567	UHRF1	0.326168001122425	ZNRD2	0.408042556010986	RPS16.1	-0.296677371199755	ILF3.2	0.258913131925576	HELLS	-0.682106732608499
PHLDA3	0.383918028877846	EIF3M.2	0.397705139602024	DCBLD2.1	-0.729622370931083	SNRPB.4	-0.479224203677989	EPC1	0.285261473571491	UBE2T	-0.685579405822781
H2AFY	0.302484479658287	KIF18A	0.263395990365473	TCEAL7.1	-0.509276996467975	TPT1.1	-0.314296451741851	RTL8A	-0.256092791060163	HNRNPA3	-0.685759821970123
WDR41	0.273450163638744	RBM8A.2	0.331324886952352	SIVA1.2	0.381552630328206	CLSPN.3	-0.473941812651387	RBBP6	0.304074862536202	CCT8	-0.690203454813325
OSTM1	0.257205179327517	PSAP.2	-0.565843192549545	IL6ST.1	-0.422835871130204	TEAD1.1	-0.38978929383062	APOE.5	-0.433326001482022	PSIP1	-0.692448804864161
MRPS34	-0.355812015168216	POLR2D.1	0.3360577860066	ZEB1.1	-0.448534830444806	C7orf50.2	-0.428896173504956	HTRA1.5	-0.351870141373829	SMS	-0.694559764677227
MT-ND2.1	-0.621057616173743	NSD2	0.353777925390337	TPX2.1	0.359505951792863	PCLAF.2	-0.655486589746907	SFPQ.2	0.253904383260087	PAICS	-0.696753705403921
YIPF5	0.28522152514394	BST2.1	-0.718389973145072	ATP5PO.1	0.305714118754831	TMEM158.4	-0.729127560187353	PLK2.5	0.27685464863013	SFRP1	-0.704850466997834
THOC3.1	0.264538929436943	SNHG17.2	0.355715494390571	NME2	0.361470569451143	USP9Y.1	0.335801489527557	DEK.4	-0.392164730195824	DPPA4	-0.708776885618712
QPRT.1	0.411131473186348	PPM1G.1	0.383541189651633	Jun 02	-0.611319650541011	ATF3.1	-0.499667565709304	CENPN.4	-0.283600630434966	MDK	-0.709880995060856
RSPRY1	0.30292854384369	NEAT1.2	-1.32339616413958	SNU13.1	0.349402178628166	CDK2AP1.1	-0.421608899834247	SACS	0.35650016502624	SFPQ	-0.711141012708385
DUSP14	0.286574701939409	LRR1	0.299444979513594	PSMB2	0.330844670022956	RAB3B	0.410967723237861	CAMK2N1.5	-0.655287170258225	H3F3A	-0.713439756057427
ITM2C	0.470435515478311	USP3	0.30935001480166	IMP3.1	0.354470634953258	CAPN2.3	-0.514704678234925	CNN3.4	-0.275596596697254	SRSF3	-0.71698846866705
ZFHX3	0.270657598974053	NTHL1.2	0.381181962661271	MND1.1	0.315871089094377	SORBS2.4	-0.671665164461546	MYL12A.5	-0.330079817806766	EIF1AY	-0.71735160705793
UBE2Q2	0.261485226382201	EDNRB.1	0.31170102632966	SERPINB6.1	-0.445669286970248	MEST.2	-0.480348670601508	GNG4.3	-0.319739290942555	SNRPB	-0.721391871007586
IFRD1	0.285934979296999	RFC5	0.251565844625214	DAAM1.2	-0.560093758904199	EBNA1BP2.3	-0.424175561305097	SETD5	0.279720283091286	IGFBPL1	-0.72431492506189
C1GALT1	0.277892279558387	FOXM1	0.252655043693575	MLEC.2	0.394099820202588	HSPB11.1	-0.406625727887219	LINC00472.3	0.453650347665749	SOX11	-0.724467517734809
HACD3	0.294446687941348	ATP6V1F.3	-0.511491299410706	MRPS34.1	0.367335825843344	RUVBL1.3	-0.417904094552483	TUBB2A.3	-0.390058027412294	MCM7	-0.728089193540778
GULP1	0.255067817425039	SNHG16.1	0.386225360833735	DLL3.2	0.668843086799504	TACC2	0.384939251072176	PRRC2C.2	0.289220800729128	GNG4	-0.73003068480339
TMED9.1	0.350744636010872	CDC25A	0.274116911550417	CUTA	-0.379873964534549	STARD3NL.2	0.458327120134389	TUG1	0.284144791462921	HMGB3	-0.731163646252088
MIAT.1	-0.657689620480395	CMSS1.2	0.401669895655363	DCLK1.2	-0.580817583131538	RPS5.2	-0.27472437609321	CAV1.5	-0.540548989321941	SNRPE	-0.732106606814776
PTBP3	0.301082040130193	EXOSC3.2	0.361663239636845	KNOP1.1	0.34955021479862	PRDX2.3	0.451997714071699	ZBTB20.1	0.283592432105984	SNHG14	-0.73573870587214
BCL2L12.1	0.29653602783754	NME3.2	-0.411059011236166	ZBTB8OS.1	0.351332503736884	NEXN.2	-0.459160711265237	PIK3R3.2	0.30009448842094	CACYBP	-0.739972144074775
TAX1BP3	0.322028077371317	NSMCE4A	0.343379441352352	DYNC1LI2	-0.47327310192017	TCAF1.2	0.615545506513239	CARHSP1.2	-0.259004946558603	COX5A	-0.741412027158316
MCL1	0.315776176448383	TRA2B.1	0.399399350321431	ZNF649	0.254120965614548	NOVA2	0.41445701534073	KRT18.5	-0.783557210020704	NREP	-0.749407237261002
DNAJC10	0.296542745823666	CENPS	0.336609869747944	ADAM9.1	-0.462620416683188	UQCC3.1	-0.409938002217999	GSTO1.5	-0.403840739548097	ELAVL3	-0.749566968135059
PCGF5.1	0.259009612034729	PRDX6.3	0.395690425583713	THYN1.1	0.366485509278592	CENPK.3	-0.40143729273637	ISG15.5	-0.742015932325136	SRSF2	-0.752378163749294
CENPK	0.307867938043674	C19orf48.1	0.389411318871897	MRPS16	0.377676220217387	TNFRSF1A.2	-0.362081870567852	ANKRD17	0.250542923517126	PTTG1	-0.752478291121639
H3F3B	-0.345285354188467	GLO1.1	0.396152901604216	DBF4.1	0.329606382389262	IFT57.2	-0.397175517017819	INTS6	0.407842364297293	SRSF7	-0.764202718481614
MCUR1	0.300065245631195	SF3A3.2	0.41985907934497	CCT3.1	0.392919381808326	HSPE1.3	-0.490252885619629	CDC37	0.263488562733389	TYMS	-0.767961972475993
SNRPB2.1	0.267392051042573	Jun 03	-0.552280618923551	LINC00461.1	-0.530593455846446	TMEM106C.3	-0.424771042912996	MGST1.2	-0.366983119310954	MAD2L1	-0.772086514728811
EVA1B.1	0.279334344042281	DCTPP1	0.509104082521747	UBE2L6.2	-0.508370503664898	HTRA1.4	-0.561997625487382	GLRX.3	-0.312175289517838	RAN	-0.778778258169769
KTN1.1	0.305195521251216	AKR1B1.2	0.389481927092801	VRK1.2	0.342350200330636	HSPD1.3	-0.599606819765437	DDB1	0.279372951786261	BZW2	-0.779840331335595
HNRNPM.1	0.296230738900834	RNF19A.3	-0.603020328479397	EXOSC3.1	0.342441696666272	COX6B1	0.301601167349607	SOX4.3	0.277632500765018	SLIRP	-0.782349688210518
GPCPD1	0.287621460814354	MEIS2	0.263073953892966	HIST1H2BH	0.275896051686571	VMP1.4	-0.462366221394193	DUT.2	-0.317210171952873	RGS10	-0.78500424491515
SPCS3	0.309778610854073	TACC3	0.341795549540284	RUFY3	-0.564776459684972	PDLIM4.4	-0.60292576468563	NCL.4	-0.287798168648831	TMEFF1	-0.788956287436274
ATP1A1	0.334731823212982	SAAL1	0.300835807333865	NRCAM.1	-0.421555233755785	IFI16.2	-0.401841368399609	CENPF.4	-0.302875338079857	ABRACL	-0.79066572489884
ARL4C.1	-0.65268072649358	BUB3.1	0.427742067117316	PRIM1.1	0.284363960089569	JAKMIP2	0.434517611452027	HSPD1.4	-0.293277214765915	RPA3	-0.795297722533834
SEPTIN10	0.28759703992766	RAD21.1	0.415415078850633	SVIL.1	-0.631959941425639	XBP1.1	-0.407912844420923	NSD1.1	0.254703038262169	HNRNPA2B1	-0.806206681845279
RAD51C.1	0.290835386660143	HJURP	0.261047019206381	IQGAP2	0.30442973601557	ATP5PF	0.300061416851109	UBE2C.4	-0.574856356431277	DANCR	-0.807752879027777
BOLA2.1	0.275851043661594	PPIG.1	0.352200762934773	C5orf24	-0.432908302159564	SLC39A1	-0.418568273202631	ANXA1.5	-0.259763559142791	RBP1	-0.809299818093231
PPP1R12A	0.335813300914552	TAX1BP1.2	-0.448170208416052	SOX11.1	-0.702690953004973	RDX.2	-0.567361734763153	CD99.5	-0.664843027179259	HNRNPD	-0.822627426359062
TP53I3	0.539299313339827	ENO1.1	0.452392511502152	DNAJC19.1	0.345980684117841	NCL.3	-0.563069780649011	AK1.3	-0.261462102246058	SNRPD1	-0.823412778823639
VRK1.1	0.283519784649246	PRR11	0.307004007059871	MRPL20	0.350728458555938	MRTO4.3	-0.432742505666227	NQO1.5	-0.320160574801236	SET	-0.823714670820096
ARHGAP10	0.261544370304007	IQCB1	0.269068051617949	CYTOR.2	-0.585325838855216	PIP4P2	0.445598584729618	ARHGAP29.4	-0.373973440825664	SOX4	-0.830179767306049
MAP4K4.1	0.366379089119065	DEPDC1	0.312741336217546	TPI1	0.326046507254753	HSDL2.1	-0.352175366887574	REV3L	0.318912607979188	ACAT2	-0.831272972619586
UBE2L6.1	-0.433807450014577	BRD7.1	0.362423317614621	SNRPA.1	0.352948905152645	CAMK2D.3	-0.384019207485699	HIST1H4C.4	-1.26930589758943	CRABP2	-0.831856868255919
CAMK2N1.1	-0.778964515657657	CXXC5.2	-0.482716153345525	COPS9.1	-0.379133315331615	MAGT1.1	-0.330052707481286	BAALC.5	-0.432764527311723	HSP90AB1	-0.844331405015747
TRIM44	0.306780723514023	OLA1.1	0.350348040590788	FGF2	0.297439325707803	CCDC28B.1	0.515619346897592	RGS10.3	-0.350545248682268	PRDX1	-0.84622593038257
NR2F2.1	-0.570956637018339	RFC1	0.415206033837473	SDC2.2	-0.502175823999735	DLGAP5.3	-0.473754073803268	HMGB3.3	-0.274776315533431	HIST1H4C	-0.855954860181287
MAD2L2.1	0.288134566333907	XRN2.1	0.331104464239458	UQCRQ.2	-0.379828566601856	MAP7D3	-0.336093069558954	CALR.3	-0.254348678817023	CENPF	-0.87058270822756
COL1A2.1	0.665741437985562	THYN1.2	0.371782940588193	RRAS.2	-0.510285240290932	CASD1	0.378221561135331	KIF1A.2	0.274109084781822	HSPE1	-0.871389623382715
BTBD10	0.273854544135456	ALCAM.2	-0.492553631899887	NRP2.1	-0.875778709223567	UQCRQ.3	-0.397281190898342	PTN.5	-1.39009774944371	UBE2C	-0.872410950833034
KCTD12	0.261656662246485	AC011447.3	0.25630611456366	SELENOH.1	0.332591873231357	SNX5	-0.376564089425337	PIMREG.3	-0.273386018966667	MT-ND2	-0.881945858585837
MED13L	0.266345986730545	CYSTM1.1	-0.437389924940903	SERINC1	-0.532212349583417	ADD2	0.349283539246181	CALB1.3	-0.42904870530935	HSPD1	-0.882383915384132
SYT11.1	-0.431149767170249	STK26.2	0.319973679156806	DYNLRB1	-0.410737768247135	CDK4.1	-0.400366123562967	TAGLN3.5	-0.49612133907431	KPNA2	-0.883485324560892
CCT2.1	0.266548964573183	HACD3.1	0.331596841765074	CCDC167	-0.412666210324513	FN1.4	-1.02893309653232	SON	0.269493931048275	TSTD1	-0.888915698662024
TOMM7	-0.311606690040299	IMPA2.1	0.256783006276532	MDN1.1	0.319270490847418	GAR1.1	-0.366332184189942	NMU.3	-0.352152843362255	ODC1	-0.889427397413148
MEG3.1	-0.764575545078675	SELENOH.2	0.33678169616042	JAK1.1	-0.494321590271352	EMG1.2	-0.384838042611183	SPRY1.3	-0.251014350746042	HNRNPA1	-0.897979413540993
UBE2I.1	0.257322635469604	SLC25A13.2	0.339706064153069	JAM2.1	-0.481898233423964	GSTO1.4	-0.526512402393951	SNRPN.3	-0.27659080622945	H2AFZ	-0.90714606182753
JPT2	0.272012633446064	HNRNPA0.1	0.364074289288329	JAG1.2	-0.519076054439952	H2AFX.2	-0.463010984363089	ACAT2.3	-0.365402846220563	HNRNPAB	-0.915980054964551
DNAJC9.1	0.304734002304245	BASP1.2	-0.62677242285576	DCTN2	-0.426400031701403	ARMCX3.2	0.530661488098172	PCSK1N.5	-0.555956769487778	IGF2BP1	-0.924846450989817
CLIC4	0.305617769305381	FAM177A1.2	-0.397777773931966	NIFK.1	0.379969396107482	NRP2.3	-0.853493622098737	SOD2.5	-0.48525300073301	NMU	-0.926253442635809
GLO1	0.295464640052499	SNRPC.2	0.326313913685576	PDLIM4.2	-0.628215751779822	RPS6.2	-0.314580072543739	TRIO	0.299148998694403	SNRPF	-0.930472268925409
SAR1A	0.280634208309524	H3F3B.1	-0.376909751896926	LRPPRC.1	0.342632144524779	NIFK.3	-0.419229853783317	XIST.4	0.664685763081922	CYBA	-0.932152442299625
ISG15.1	-1.33247310245963	SDCBP.2	-0.445650151356638	MAP6.1	-0.498931600768316	TGIF1.2	-0.385928231209971	CKS1B.5	-0.457847009095899	SCG3	-0.964107637813373
APOE.1	1.01588765378167	WDHD1.1	0.274772494117472	PEG10	-0.653041399285594	KDELR2.3	-0.407157271990198	PCSK7	0.270723026817413	NPM1	-0.964161929047816
CLIP1	0.306388908058999	HMGA2.2	0.308732589604652	DHRS7.1	-0.461546205023503	TCEAL9.3	-0.490337414147173	CRABP1.5	-0.671876895056265	CKS1B	-0.974295357908502
RAB11FIP1	0.26864807168713	GLIPR1.3	-0.497666580838236	SSR4.1	-0.360202494995488	TM7SF2.1	0.535399787830026	SMG1	0.304464663892018	STMN1	-0.995477046742259
SEC14L1	0.290070766472989	WDR34	0.414631834351988	BCL7C.2	-0.363736665723429	IQGAP2.1	-0.319234685170942	CCNB1.4	-0.273870982411355	LIN28A	-1.00255792304475
CLDND1	0.256015765929603	NCAPD2	0.321347232613237	BSCL2	-0.510820119317646	CDK6.2	-0.500203215995867	TAGLN.3	-1.25433557970327	FST	-1.00479008234897
SH3BGRL3.1	0.341301115720227	EEF1E1.2	0.347677576757157	DKK3.1	-0.460681758460138	COMMD4.1	-0.376316742899109	LINC00632.1	0.361553143699242	INA	-1.01962380774399
TCOF1.1	0.254299873479708	HAT1	0.353459132934208	LSM5.1	0.331652855662012	SLC2A4RG	-0.3806686915891	CCNA2.4	-0.253186801545377	SINHCAF	-1.04051907038874
TCEAL7	0.317110035396852	GLRX5.1	0.338053106277906	MXD4.1	-0.50969433220907	DTYMK.1	-0.416102919738395	TIA1	0.278466090092551	PTMA	-1.05092539103517
P4HB.1	0.309460272187618	APLP2.3	-0.509667888453962	ACTA2.2	-1.20455551783551	HAT1.1	-0.354691158268719	ID3.2	-0.843313517905895	CENPV	-1.07133773733441
CEP55	0.272403189140314	CAVIN1.3	-0.445975551848072	KIF1B	-0.488486842976857	NTRK2.4	-0.673123950072276	ARL6IP1.2	-0.393624917446555	UBE2S	-1.07974839117589
HNRNPAB.1	0.278284137927859	KRT18.3	-1.37685102860615	RPL10A.1	0.278414350009072	CENPW.4	-0.400767366424137	RSRP1	0.363771627424721	RANBP1	-1.09178440064777
CD276	0.255613615604657	NOP56.2	0.509094916302263	MAGOH.1	0.337299013877597	CDC42.3	0.357421543721635	PTX3.5	-0.307836019502568	MYL9	-1.09947921522693
PPME1	0.287222752991912	TMSB4X.3	-0.599806831687053	DYNC1H1	-0.562245206758868	KCNQ1OT1.3	0.761845185379947	PVT1	0.270289503926915	HMGA1	-1.12793473233543
TMEM97.1	0.305476875978571	SERPINB6.2	-0.410466897570141	TAPBP.1	-0.523649317388752	SHC1.3	-0.429449063526607	A2M.5	-0.464895732700673	TMSB15A	-1.13450676385472
KHDRBS3	0.277659404103072	MRE11	0.267526986957004	RRP15.1	0.329198819248721	KIF21A.2	0.62942927383049	IFITM2.5	-1.47976674696072	SPP1	-1.14519617780271
MCFD2.1	0.293982336416023	MYL12A.3	-0.451602078778659	ZWINT.1	0.312912370862108	TRMT112.1	-0.370070405714993	MYL9.4	-0.570222624227659	NASP	-1.16609913488139
DLGAP4	0.27895809825701	SRSF1.1	0.395555607164604	TIMM10.1	0.338087470840153	MYDGF.2	-0.416779834450782	HCG18	0.301564846217871	APOE	-1.16846268737717
TIGAR	0.284699179057658	ACP1	0.333506423027776	SOCS1	0.293128748540677	LAP3.1	-0.472126763445646	PHLDA2.5	-0.251918429251184	IGDCC3	-1.1699420613801
MLEC.1	0.289289076229422	EIF5.2	0.304647535511136	POLR2H.1	0.365662913467378	RANBP1.4	-0.515471582436101	RBM5	0.350859241971452	MT-ND1	-1.19299935281757
NDUFB10.1	-0.341636334007084	LYAR.2	0.449926413724308	PAGR1	0.329062511345339	UFD1.1	-0.351573403758772	SPARC.5	-0.273839938757228	HES6	-1.20046215523296
ORC6.1	0.350370676096419	HAUS6	0.323147147249657	EVA1B.2	-0.426014344768798	ARID3A.2	0.373599650618664	S100A10.5	-0.633126911112611	HMGB2	-1.23685837079091
ATAD2	0.293464890866194	DLL3.3	-1.60899125266265	SEC62.1	-0.391787907045277	TMED10.3	-0.376322749709776	PCLO.1	0.318882490365158	TAGLN3	-1.23931350374786
YES1	0.273918793665622	HIRIP3	0.28062741963678	CCDC80.2	-0.622255463989112	TNC.3	-0.500287240022614	CCNL2	0.415616345520658	L1TD1	-1.27645024915443
MRPL41	-0.294967012795815	PSMA4	0.27401710108799	DPYSL2	-0.500134271238615	EXOSC8.3	-0.415879399126181	ASH1L	0.277918206057647	KRT8	-1.3804663186849
NFIB.1	-0.405152905450849	SH3GLB1.2	-0.389264226655367	SDCBP.1	-0.483431289869314	CENPN.3	-0.369001221289919	TNRC6A	0.328001157264404	CD24	-1.41421706574636
LRPAP1	0.335871499237119	IMMP1L	0.339013805117823	THOC3.2	0.284354111099524	SUMO2.2	0.280939996570674	PLXNB2.1	0.286129601271209	CKB	-1.46103420966186
SMDT1.1	-0.407839177383739	APOE.3	-1.02411548844262	GPX4.1	-0.332530281023416	VDAC2.2	0.31928623590606	LINC00662	0.257155005930257	POU5F1	-1.46338043721967
SEMA6A.1	-0.47453757627342	THOC6.1	0.273157649621894	COA1.1	0.320106663985782	YWHAH.2	0.490478786776827	S100A11.5	-0.907143575080832	DLL3	-1.89765609787218
CMPK1	0.258769108752955	RTN4.2	-0.440400403449644	GNB2.1	-0.406808542955614	EIF2AK2.3	-0.405692478162861	PMP22.5	-0.740919263227277	NNAT	-2.38123429452981
MESD	0.265532850077524	LAPTM4B.2	0.323490409350198	SNHG5	0.392233733008665	MDM2.1	-0.734000759975115	RBP1.2	-0.557193539389722	NEFM	-2.72313284420067
PUM3.1	0.281920428761574	DKK3.2	-0.447817771338391	ARPC2.2	-0.343270146628569	COPRS	-0.359103248710651	IFITM3.5	-1.55207201152514	STMN2	-2.73720726485687
DDOST	0.280899092388068	HMGCS1.1	0.4311218544381	MRPL34	0.345010419752066	CDK1.4	-0.462955463046777	B2M.5	-0.845934548137284	PDLIM2	0.424140535144912
DAAM1.1	0.269472764181252	KIF23.1	0.306225691758315	WDR43.1	0.338326905331894	NOTCH3	-0.296839455942338	HMGB2.4	-0.519920179010529	ELK3	0.308428638570768
NXN	0.251445368303499	LARS	0.362029032808279	C12orf45.1	0.394286938982608	TXNDC12.1	-0.335541545275209	BTG2	0.273336563182434	ADH5	-0.40784560791495
TMEM98	0.282597996954552	RPS2.1	0.320655570953963	KIF23	0.298455164243512	TKT	-0.444148449398478	GPATCH2L	0.274793019562328	YBX1	-0.460870316176329
RPN2	0.27790677073413	TOX3	0.315401386360713	RUVBL1.1	0.361042527536086	ANKRD46	0.320145693790269	NUMA1	0.265161954155503	CENPW	-0.415580119291492
HNRNPR.1	0.258982097461731	TAF15.2	0.481243568620581	NUDT5.1	0.348332496442663	VGLL4.3	-0.40818107559414	EIF4EBP1.2	-0.32847265974572	CALR	0.423738714220181
APRT	-0.377858453119894	ITGB1.3	-0.532727523995242	EIF4A1.1	0.353798335816045	NOL7	-0.344100492689264	GAP43.4	-0.547660770326876	ARFGAP3	0.359668448229612
RHOQ	0.273858534338599	NDUFA6.1	0.304289814185255	C17orf49.1	-0.416699670948416	ACTA2.4	-1.32879344557906	HLA-B.5	-0.806011682277466	NEFL	-1.18530673380176
CD81	0.369907195541961	TLE4.1	0.322450256125414	CALM2	-0.528464511959623	KHDRBS1.2	0.357417590494378	METRN.2	-0.256390987998277	NFE2L3	0.374417192425284
MLF2	0.299633701239361	CAPNS1.2	-0.399285010723454	EPS8.1	-0.424845731726586	MMP2.4	-0.458941683831425	MDM4	0.252507400029336	TNIP1	0.471207420477101
COX7B.1	-0.297675098903521	IFI6.3	-0.89475369310804	TMEM59.2	-0.474625347432944	LIN28B.2	0.393857486313554	ID4.5	-0.314867311763261	PRSS23	0.587403500449411
TEAD1	0.297255047631611	ID1.2	0.413571563146651	IFI27L2.1	-0.512350473752202	GSE1	0.339344149474667	GLS	0.252470478351606	VPS28	0.383495804122452
AKR1B1.1	0.273131867870831	RNPS1.1	0.392921274519579	PRDX3.1	0.328678534645839	RRBP1.3	-0.408082371317975	POU5F1.4	-0.633957043254945	MMP2	0.476983847274789
MIDN.1	0.301722758906044	RRBP1.2	-0.416004231430797	SNX3.1	-0.376381590761779	SSB.3	-0.354652402792665	SLC25A37.1	0.264535338274767	DCBLD2	0.758156976928833
MSMO1	0.329548890608167	KIF22.1	0.30894835876373	CCNC	0.3174268735447	PRSS23.4	-0.694181917432196	RDX.3	-0.259032774765192	ACTN1	0.507195420535025
KDELR2.1	0.28794386552443	ABCE1.1	0.4092413559325	AP1S1.1	-0.423406552854664	PAICS.3	-0.466608053177957	CDK1.5	-0.309774014465018	CKLF	0.369474351948931
SYNCRIP.1	0.255989693566934	SNHG1.1	0.307543518820697	UQCR11	-0.322537466806945	RAC1.2	-0.298027318727306	FABP5.2	-0.329787891090245	RFC4	-0.429667242752057
MVD	0.263976726601084	SESN3.2	-0.48830986043596	TGS1.1	0.353005779840969	MAD2L1.4	-0.371533553198877	SFRP1.5	-0.457664634975804	AGPAT3	0.253983738437879
TPD52L2	0.250954993528644	C9orf116	0.306310143543915	XRCC5.1	0.286497198456516	WASF2.2	-0.358168123681567	HES6.4	-0.328289146448506	TIMM50	-0.507148429709801
PRDX5.1	-0.327128488027969	RBBP8	0.335714326494272	CENPK.1	0.281956859176141	TULP4	0.433944796968057	TMSB15A.3	-0.42662988735952	MPC1	0.410549831139525
TAGLN3.1	-0.9891197720157	ARL2	0.329550237833181	TAX1BP1.1	-0.447727960277171	SQSTM1.4	-0.885848801137171	CHCHD2.5	-0.602989706064314	S1PR3	0.3398971376121
SBDS	0.32342068998484	CBX3.1	0.330509412491125	TBCA	0.314007923826686	BZW2.3	0.385073486821535	KMT2C	0.282775030184058	ATP2B4	0.308370785166636
C12orf57	-0.357005852221553	PPID	0.396333109998592	CD2AP	0.288497727257479	PTTG1.3	-0.412921761499545	FABP7.5	-0.71146307024282	MYBL2	-0.422851246463276
PABPC4.1	0.256349413958503	ELP5.1	0.32912026134914	IGFBP5.1	-1.27418983432461	CDC20.3	-0.479231379933462	BX890604.2.2	0.311473326677858	TPX2	-0.475573000427846
NTAN1	0.259943863775452	NDFIP1.3	-0.4895768737199	GINS2.2	0.288739476174568	TBPL1.1	0.442941805810997	CD24.4	-0.454167307516292	EIF1AX	-0.465423618510921
F2R.1	0.28526695527271	TK1	0.280852329924392	HDAC1	0.297830934989738	CPEB4	0.333690635383732	KANSL1	0.300421020083931	NDUFB11	-0.359355947920714
NDUFB1	-0.287609396803983	CCDC160	0.266691588408888	PERP.1	0.313911570254606	CKAP4.3	-0.433767147073565	NEAT1.4	0.288175372807791	RGS3	0.479265173030826
PERP	0.348314222621381	BRIX1.2	0.337494180011246	MRC2.1	-0.38155256195139	GNG2	0.490693192795968	TPM2.4	-0.822267971689204	TMEM35B	0.309042595140983
FAT1	0.323567147622932	CCDC18	0.262169566303256	TCEAL9.1	-0.497157074361714	C12orf57.2	0.401241882635406	TUBB3.5	-0.505942736662146	DYNC1LI1	-0.422771442480023
KPNA2.1	0.339374989764385	NFIB.3	-0.439285937485364	PAM16.1	0.337334162602593	PURPL.2	0.369316202551171	LGALS1.4	-1.20581713040141	CMSS1	-0.433439569011957
ACTR2	0.307083499559689	SPARC.3	-0.919113739546439	MYDGF.1	-0.400779503048438	HMGN3.1	-0.350326608627389	SLF2	0.254613043624229	LINC00461	0.447929591385062
CTSL	0.254133412939185	FAM216A.1	0.29955504589098	SCOC	-0.430998519882769	S100A11.4	-1.42572677873874	SNHG9	0.407749002734893	DRAP1	0.376091033068826
NCS1	0.26762261386678	DKC1.2	0.40654861242889	DANCR.1	0.381532072889175	WDR1.1	-0.438145253361227	SPG7	0.260879314628813	SNU13	-0.390090329926128
ACOT7.1	0.278626700158014	RRP1B.1	0.421036096882011	UBC	-0.50480994703118	MYCBP2.1	0.593158266035937	IGDCC3.5	-0.3185276751331	GABARAP	0.282177004653
RAB13.1	0.307973326965027	JAK1.2	-0.43605691494971	MCM4.1	0.308072352317282	H2AFZ.4	-0.622005031589561	DLL3.5	-1.14951442474576	ZNF738	-0.380318533431445
FDFT1.1	0.297417025489654	RTRAF.2	0.271023570618667	RBM17.1	0.323670798366354	TMA7	-0.300532604620099	CCNL1	0.307027180450243	MRPL15	-0.415598649972659
BARD1	0.258490676703556	RUVBL1.2	0.430115321596432	YWHAH.1	0.293218662495255	MCM3.3	-0.43554216933852	PCNX4.1	0.268937856137689	DNAAF4	0.313151425056217
ATP6V1D	0.307350211266333	CPSF3	0.295859155445349	CDK1.2	0.346136976723897	RPL17.2	-0.277592625842942	TTYH1.4	-0.379193446759984	ITGB1	0.429665639337269
GDF15	0.450773387421959	ING5	0.255729049249494	RFC4.1	0.301744672317579	CDH11.2	-0.422249649772724	LIX1.4	-0.259522979580936	FKBP3	-0.38799923307593
ID1	-0.496601429022115	NUP37	0.290226818615518	BNIP3	0.369710096194113	CCL2.4	-2.1799703164183	TFPI2.2	-0.354820357913513	TRA2B	-0.450327953374146
EIF3A.1	0.288197070319906	S100A13	-0.483641326113297	RAB14	-0.410917240443979	MND1.3	-0.348625793321036	UBE2S.4	-0.324181320698187	MORN2	0.384479439052897
BAALC.1	-0.564863909592656	NELL2	0.281513517379551	MYL6.1	-0.35311878321951	CMBL.1	-0.365512732086002	SELENOM.4	-0.28277489421453	PHB	-0.439033152331874
TOMM40.1	0.255059188507969	MRPL3	0.388339332630479	TMEM50A.1	-0.415085405073419	ROBO1.1	0.430702708585686	SQSTM1.5	2.21778636457016	CENPN	-0.406395340889912
RAD23B.1	0.277682652964672	HIST1H1C	0.551918652302799	TNIK.1	-0.421873208042045	SNHG5.1	-0.42535396069928	FTL.5	1.94871020110131	AURKB	-0.452762421694451
AZIN1	0.256545282394895	GLUL.1	0.351595684584198	EIF4G2	-0.421516786034461	SNHG14.2	0.455796841351563	PRKDC.4	-0.962303746383619	ALYREF	-0.422899646320502
ANKRD11	0.337872959240693	ATP6AP2.1	-0.366209684340637	PRR13	0.358560908058654	TOMM20.1	0.327610657805759	EEF1B2.5	-0.811826864089515	MRPL12	-0.475801411464061
HNRNPH3.1	0.251326973970835	IGF2BP3.1	0.364286262475358	TOMM40.2	0.385084357652122	TMEM132A.1	0.479990129028874	MDK.5	-0.916213839897646	RTL8C	0.407927938083302
IGF2R	0.252555715205451	MGME1	0.2987076478984	VPS35	-0.384540474427832	PDIA3.1	-0.373217841623062	STMN1.5	-1.30559670930309	MBNL2	0.268010038512732
PLGRKT	0.263033063844701	NPC2.2	-0.505076871440156	MICOS13.1	-0.420360491768853	ARL4C.4	-0.645374428254812	PPP1R15A.2	1.25812059849637	HMGN2	-0.480835059422089
TDG.1	0.270463309224413	DNAJA1.1	0.303893325138536	LYRM2	-0.390459321805194	DYNC1LI1.1	0.444381760673497	RPL12.4	-0.655212774084132	ACP2	0.303803561298481
RFC2.1	0.255329698381926	ATP6V0C.1	-0.407877181826037	SQSTM1.2	-0.861328580704616	DCTN2.2	0.407822848787915	KRT10.3	0.950720988978308	PPP1R14B-AS1	0.257713114117868
TTYH1	0.291138329839746	TPR.1	0.41509211251716	HTRA1.2	-0.578800545870575	RFC1.1	-0.355050625804972	TUBA1B.5	-1.09843339288867	RPL5	-0.263604767051734
NOVA1.1	-0.577427141267957	PAX6	0.435687239708543	IFI6.2	-0.908634754009064	FLNB.1	-0.388226931689967	ACTB.2	-0.837277253418786	UBE2H	0.464629073171599
DRAXIN.1	0.364774942805554	EIF4A1.2	0.312814208722181	TOP2A.1	0.327147157962103	TMTC4	0.347866026615435	TUBB.4	-0.861023315481491	ELAVL2	-0.59334887044911
UFM1	0.287812738173817	SRRM2	0.332675083656802	TSC22D1.2	-0.592137279029751	ARID5B.4	-0.432099055156699	RNASEK.5	0.732743085301563	ITM2B	0.430410894362586
AKAP13	0.293092612477283	TMOD3.2	-0.379269540896591	KIDINS220	-0.57426829556642	WARS.1	-0.421024813987276	NAP1L1.3	-0.696870204345799	SARAF	0.399834459765517
ELAVL1.1	0.263914271864095	LTV1	0.30986466231495	PPAT.1	0.315511067951444	SDC2.3	-0.454183839564197	RPL10A.4	-0.631088379595061	P3H4	0.323823678827792
Jun 01	0.278480142846753	ZNF511	0.328081704014292	ISG15.2	-1.42340767282439	CHCHD5.1	-0.349805274389372	COPS9.3	0.696147709949383	ANP32E	-0.520959610658766

COX5B.1	-0.273906744592868	UCHL1.1	-0.499219151582878	LGALS3BP.1	-0.405685767786211	ATP6V0E2	0.443267390189649	PPIA.3	-0.603940344230353	TERF1	-0.641091167851931
CAV1.1	0.330814113669982	LYPD1.3	-0.499479269314906	KMT2E	-0.464989027856836	GGCT.1	-0.395049986764132	SLC3A2.3	1.07367007864596	CORO2B	0.278660393885314
RAB5C	0.263793584705438	MLLT11.2	-0.772988383689182	TMSB4X.2	-0.538729744356366	TPX2.3	-0.462562586471643	CALM1.3	-0.634470184001316	UBA2	-0.409616767650427
PHLDA2.1	0.41534146648003	HNRNPF.1	0.4096688118206	ID1.1	0.564399501264261	TMEM263.2	-0.313440805553182	SNHG7.1	1.09386371162118	PRR7	0.296410563453547
MCM7.1	0.287939558778835	SYNCRIP.2	0.409647322683826	RTRAF.1	0.282643761075568	LCOR	0.478056939643265	CD63.5	0.785854906115638	SEPTIN11	0.423686325161206
CENPF.1	0.360210479668922	TIPIN	0.278848784070179	NUDT15.1	0.314418451596922	MTFP1	-0.301631865924312	RPL10.1	-0.547972187453211	AK1	0.434841963226101
CST3.1	0.346168863372711	NAA50.2	0.349909084850346	INAFM1.1	-0.445137390297681	FBL.3	-0.423479795646928	MIR22HG	0.610827674090209	PAM16	-0.403319977753076
NCAM1.1	-0.537717649054125	EXOSC2	0.265831540621262	NDUFV2.1	0.290422502412971	BEND5	0.295810894729429	H3F3A.5	-0.598596954158627	SSB	-0.396471456635318
NREP.1	-0.468945833936286	RBMS1.1	-0.361161960398285	CXADR.1	0.259803620222769	MFAP2.1	-0.350146753902573	A1BG.6	0.770865311406304	VEGFB	0.301636216497703
ACBD3	0.268777089570137	HNRNPL.1	0.339026758650774	PEBP1	-0.264835577625451	MACO1	0.38158264730621	HNRNPA1.3	-0.839793289245641	ZMAT3	0.408935961625714
ARCN1	0.252945692132588	MCM5.1	0.285513354009146	CTSB.1	-0.493005843595427	CENPH.3	-0.375096483781975	RPS9.1	-0.536291978424928	GPC1	0.364736953379541
KPNB1.1	0.264687836909989	UGP2.2	-0.481035003297543	TCF25	-0.380874843664972	GRB2	0.479471300852242	YBX1.2	-0.657546576617216	CECR2	-0.405792272498055
RPL27A.1	-0.269740075306476	ATP6V0E1.1	-0.374107501709521	IER5L.1	-0.436457040101438	BCL7A.1	0.376339948064516	NEAT1.5	1.76644423807549	SRD5A1	0.317481217272057
MT1X.1	-0.60062585330216	EXOSC7.1	0.329686936234162	TLN1.2	-0.500501773919608	RAD51AP1.3	-0.331158495886433	RPS8.1	-0.422291220907419	CKAP4	0.459019231371696
KIF21A.1	-0.390913589924419	LMNB2	0.29252082501048	MRPL16	0.334911383109922	STX10	-0.296435735863127	EIF1.4	0.74875631433706	SNHG17	-0.416122961859619
KRT18.1	1.79509937654634	RCAN1.2	-0.630851010805014	PTPN2	0.296862639404893	NUDT14	0.379044995069724	NDUFC1.1	0.723280179189718	FAS	0.261223727053403
GINS2.1	0.274024149548236	TRAP1.1	0.384226444229805	RABAC1.1	-0.39458358848623	VAMP3.1	-0.294327160865635	RPS19BP1	0.758273336910384	KDM1A	-0.450334627591353
BDP1	0.255136654614953	SDHAF3	0.280181735441053	PPM1B	0.299740822145933	PABPC1.2	-0.398858871642492	RPS6.4	-0.506788216007085	BRIX1	-0.427611874682954
PGRMC2	0.260170935381378	TRIP13	0.250874054536351	NOP56.1	0.393606691153883	PARVA.2	-0.304687712627985	PTMA.4	-0.786278389627428	RPL18A	-0.2836829636394
MAD2L1.1	0.281953659446134	PDCD7	0.331225636361247	RGS16.1	0.539880324196908	FAM241B	0.374640839734814	LAMTOR5.1	0.667035204850823	SYNM	0.31748829280231
MAP2	-0.583439258602247	ITM2C.1	-0.429394868597714	SURF2.1	0.333421582005012	MLEC.4	-0.39846868912985	RPL8.3	-0.479069118133083	SEPTIN2	0.366714035490439
MTHFD2	0.318789773737679	ZFHX3.2	0.293708418722769	MCM6.1	0.26912168464639	MCFD2.3	-0.35944160756239	TCEAL4.4	0.767991233121573	SYNE2	-0.454892284736208
ATP5ME	-0.270207906566427	CTXN1.1	0.313178754508471	GDI1	-0.465799444146927	SORBS3.1	-0.280860253094274	DAD1.4	0.619912835031624	SYNJ2	0.334975774936675
DNAJC15.1	0.264307751055077	SMDT1.3	-0.42771554980398	ECT2.1	0.285713286096454	RPLP0.3	-0.290669716562937	CHCHD2.6	0.852627159084933	NEK6	0.341284674279706
PPP1CA	0.25331872829551	SEC61B	-0.291263201807343	GADD45G	0.5756986099955	NUCKS1.3	-0.336782591707763	RPL3.1	-0.571041080726883	TAGLN2	0.305274627622167
BEX2.1	-0.427999868145263	SARAF.2	-0.421447280781968	ZFP36L1.1	-0.511945831373867	NFKBIA.3	-0.571272203607885	TNFRSF12A.5	1.12124047236083	LRRN3	0.30648357925934
GMNN.1	0.270871660232707	SPTBN1.2	-0.448602668669911	UNG.1	0.297314689049219	GGH.2	-0.337282235153836	RANBP1.6	-0.956867345892099	TPP1	0.284007092954572
S100A10.1	0.614466321488776	CHRAC1	0.251004266413664	ARL3	-0.364468892422546	TSPYL4.1	0.468008835255194	SNRPN.4	-0.770632785267586	IRS2	0.359368988658246
IFI6.1	-0.765521136333831	RTL8C.2	-0.4110586363423	PAWR.1	0.306856813724938	CENPU.3	-0.358019214315995	SNHG19.1	1.35393806622605	STUB1	0.369658342224693
CRABP2.1	0.422824607071174	EXOSC9.2	0.324642492264539	ANXA3.1	0.359341491394238	NKAIN3	0.395759594260158	RPSA.4	-0.526428737520004	ZMYND8	-0.426216021896283
DCLK1.1	-0.354332838915587	SCARB2.2	-0.416394165446829	MAP1LC3B	-0.455905617624733	CRELD2	-0.378811665053145	RBP1.3	-1.10036437132351	MYL12B	0.343545759848336
HELLS.1	0.263246989917266	MMAB	0.319349850024894	TPM4.1	-0.490537751681145	PRDX1.2	0.500188914323911	DDIT3	1.10330016772983	KRAS	-0.388695095959413
HES6.1	-0.770933547344476	TMEM50A.2	-0.372582361954958	PPP1CC.1	0.325235207922819	POLE4	-0.334610109535542	RPL14.3	-0.481760603063605	PLS3	0.546587544604954
UQCRB.1	-0.268850517157163	OARD1	0.307779674719376	ADH5.1	0.309068477166391	FEZ2.3	-0.397819577134095	CDKN1A.5	1.05022651855289	GPC3	-0.351821009779437
ATP5IF1	-0.260204272921672	ANK2.2	-0.538150997112497	TCEAL3.2	-0.444799877413871	SBK1	0.367188144511228	RPL7A.1	-0.441598420334201	RER1	0.369535183111106
UBE2S.1	0.299146739903766	GRN.1	-0.401323037468688	PFDN4.1	0.306262649185413	STK26.3	-0.317327570831528	BRD2.1	0.854095983678454	MTURN	0.30144031744939
TIMM13.1	-0.275317665695837	CRABP1.3	0.553414988860555	POP7.1	0.329988717144259	SARS.1	0.413279735675878	ATP6V0C.3	0.633039832641467	RAB2A	0.333961745808649
APLP2.1	0.380982862517929	IGFBP7.2	-1.21260954027688	GNG12.1	-0.374992356600828	DHFR.2	-0.430956823187872	UBL5	0.53443081089451	MAP9	0.352624739200453
PRDX2.1	-0.31183203058748	SRRT	0.331849223408276	CAMK2D.2	-0.398836139117497	PCMTD1	0.362930263956881	BRI3.3	0.632733662636851	LUC7L3	-0.439322758117036
ANK2	-0.464042628671359	HPF1	0.325031504451352	TMEM106C.1	-0.394093518861926	PCCA-DT	-0.322803803019194	LMNA.4	0.801883180401945	ACBD5	0.303448254515836
HMGA1.1	0.271219595884979	SNRNP25.1	0.387543874279997	YWHAQ	-0.307791263167798	GLI3.2	-0.322256682078799	NMU.4	-0.784047519086322	LIPA	0.272062492612165
CLU.1	-0.617659923520485	HNRNPH1.1	0.386466461441495	SRM.1	0.364810521648114	POLD4.3	-0.422996571091361	MED10	0.76397313021114	MYH10	-0.456059577407605
MDM2	0.574948608178629	DERA.1	0.285207318281832	TCOF1.2	0.317858975924617	SDHD.1	-0.317427527186983	LGALS3.5	0.903756974769375	PPAT	-0.419051934792152
PNRC1.1	-0.295062391177439	ZNRD1.1	0.292798325583378	SEPTIN2.2	-0.371097978715611	PAK2.1	0.388231918097989	BIRC5.3	-0.881180132408942	MAGOH	-0.416362844529225
UQCRQ.1	-0.300915530395427	TAGLN2.2	-0.330051859140229	ZMYND8.1	0.287912110016626	PET100	0.343015445463445	RPL15.3	-0.416349057986069	PGAM1	-0.403529735509964
TUBB4B.1	0.314771960964993	AURKA.2	0.355582502415346	RSL1D1.1	0.332516142689783	SLC16A1.3	-0.377870834314692	UBE2T.4	-0.795273448654519	RNPS1	-0.447247995654805
SOD2.1	-1.18334814974984	CDC42EP3.3	-0.375081858894023	CALD1.2	-0.942912387710555	SNRPG.3	-0.329670678781439	XRCC5.4	-0.590493828991385	POP7	-0.425901247341631
CCDC85B.1	-0.380552237907184	LUC7L2	0.364773771036558	EMP3.1	-0.567845938719355	TOP2A.3	-0.433482640656303	RPL32.1	-0.38300988435439	SMARCA2	0.268888596683758
TAF15.1	0.257331285258187	ENC1.2	-0.639502315991386	MCRIP1	-0.404034227775004	CENPM.1	-0.316322753793705	KRTCAP2.1	0.556737939070775	MEG3	0.35874703404208
MT-CYB.1	-0.461108778870467	HTRA1.3	-0.543537550173549	STARD3NL.1	-0.382860760210508	MORN2.2	-0.328299577579691	LDHB.4	-0.592919075017736	CDCA7L	-0.356729761403201
UBE2T.1	0.255007137322707	ELOVL6	0.31628500847638	IFITM2.2	-1.98810928684819	CTSB.3	-0.418890414223745	CENPH.4	-0.680591345378602	TPM1	0.38766168907725
PSMD2	0.311257760080933	STOML2	0.325168068053795	RIDA	0.291866758360755	CRABP2.3	0.626007063784938	CKS1B.6	-1.02728859093726	CBX3	-0.413665529480854
PSMB10.1	-0.32302703075241	NETO2	0.321648816741597	N4BP2L2	-0.415706354584597	TMEM47.1	-0.307231342925798	MAD2L1.5	-0.786032133320116	BCL2L12	-0.417268415141927
GSTO1.1	0.301218192642988	SMARCB1.1	0.315032474272601	COA4	0.371643083194794	ICK	0.393659597441258	SELENOS.3	0.645128930256257	GPATCH4	-0.514808602596182
VGF.1	0.324301810496255	DYNLL2.1	0.309623066686515	CANX.1	-0.416911307461943	PLD3.3	0.420938636883404	AL118516.1	0.706238195988715	TCTN1	0.273977678700085
C9orf16	-0.337165153566915	CDKN2A.2	-0.503520288412106	CADM1.1	-0.50519414896741	PLK2.4	-0.487504386995329	IRS2.3	0.703844398109936	ZEB1	0.365194122568267
MT-ND6.1	-0.515075500532682	PABPC4.2	0.390455530294392	GLIPR1.2	-0.474077367164054	MCM4.3	-0.406870588899815	PSIP1.4	-0.693565557825813	ITGB8	0.348288535577435
NPDC1.1	-0.315587098729624	PRPF19	0.333170997780277	PFDN6.1	0.305217820362999	TOMM40.4	-0.405331096227271	MARCKSL1.5	-0.646116906848155	RPAIN	-0.409000697563164
DEK.1	0.285536774982533	BRI3BP	0.288724504594387	DNAJC2.1	0.327538791578449	NDUFB4	0.314888958698633	H2AFZ.6	-0.91605385651909	CAMK1D	0.261792180872201
CENPE.1	0.264498203060185	LIMCH1.1	0.272964385089416	EEF1E1.1	0.305307658733142	KIF20B.3	-0.41733650042149	NUCKS1.5	-0.663963664212027	TM9SF1	0.305421996458129
VKORC1.1	-0.261228364832077	TSR1.1	0.372747880366213	NEFM.2	-2.44204897318461	TPM2.3	-1.08084441696081	HELLS.5	-0.772676990730115	ENY2	-0.375004064387082
MACF1.1	0.330898828984386	COX7A2.1	-0.306374199877434	DESI2	0.295357270126784	RCAN1.3	-0.577275274580086	EEF1A1.1	-0.562659553728495	ADSL	-0.403170832614164
MT-ND4.1	-0.461344171767839	CENPX.1	0.376240042121579	FAM89B	-0.39069498936258	PFKP.2	-0.418663906594595	ATP6V1F.4	0.654675072543558	MIDN	-0.414471246527873
MT-ATP6.1	-0.481611445972352	KPNB1.2	0.412837506022618	FYN	-0.405707699468374	P3H4.1	-0.288203740717001	ATP6V1G1.2	0.563272382303362	ARID3A	-0.372160985312837
UBE2H.1	0.303123508329151	PCM1.1	0.362053910611447	LMNB1.1	0.254345868783142	RPS3.3	-0.275459099749597	ROMO1	0.568212192036522	CENPU	-0.372767515046393
TCEAL3.1	-0.338201119446881	FAM161A	0.288409139885431	EXOSC7	0.305576015479485	CDV3.1	-0.408403979702396	ATP6V0E1.2	0.65984778705977	IFT22	0.379746590301499
DDX24	-0.27965547867037	DIMT1	0.286729830240425	RPL4.1	0.314397777165277	G3BP1.3	-0.365605253337752	NR2F2.4	0.794474462022301	GNL3	-0.435023676920743
SMC3.1	0.253557710781679	UQCC2	0.290026059618791	PLAGL1.1	-0.399764148750088	MKI67.1	-0.442066775840682	RPL5.3	-0.399459136465944	ATP6V1F	0.438518174237938
COL4A2.1	0.314618567344534	BOD1.1	0.336242875337839	SNRNP40.1	0.288592972394387	POLE3.1	-0.339390217607522	NASP.4	-0.99587276887694	SSR3	0.335669576963471
SCAND1	-0.262298235039837	ARPC2.3	-0.341810068357899	ERH.1	0.255809105281403	RFC2.3	-0.343057023474304	ATP6V0B.3	0.595335143223581	MRPL42	-0.380666730977369
PBX1.1	-0.452211777885895	TOP1.1	0.350231386575135	YAF2	-0.300897249436031	ETV1	0.437265182185817	FBLN1.2	-0.653453173490459	SNRPA	-0.418737251133478
RNASEK.1	-0.312743925918761	APEX1.1	0.296889240249173	PRDX6.2	0.391082969621322	RRM2.3	-0.376243136411996	CCNB2.4	-0.810022991983356	MT-ND4	-0.722357245571969
GAS5	-0.332863107612611	LAS1L	0.271068059552162	ZBTB20	-0.377390623353914	TCTEX1D2.2	-0.321059269261097	CKB.3	-1.09137326577804	LRP1	0.460794572487233
RTN3	-0.274238990430127	DHRS7.2	-0.36857008665865	EIF1AX.1	0.318536743468691	LINC00472.2	-0.43282454590965	WDR83OS.1	0.541470203872426	TRIM28	-0.502554951920777
BLOC1S2	0.273093538766658	PGLS.2	-0.392692701245259	CHMP3	-0.362841225556079	AURKB.3	-0.352550906146724	TCEAL3.5	0.66725204700368	RTRAF	-0.343479402281384
GABARAP.1	-0.280414240441355	WDR43.2	0.353979453086246	NAE1.1	0.288360666422278	MYBL2.3	-0.345593798262964	RPS3.5	-0.514630278205492	ECE1	0.363169648263054
TCEAL4.1	-0.305883669131151	GART.1	0.367066248132103	DPY30.1	0.2940626960798	SELENOW	0.332567602015625	TPX2.4	-0.93863503761057	MXD4	0.459281712229563
RPS27L	0.270550067284884	NUP62	0.320651294312736	ZFHX3.1	-0.389061738806704	CST3.4	-0.505493005734837	RPL29.1	-0.41061270828342	DDR1	0.443972191681705
HTRA1.1	-0.405911512322225	FH	0.30436151613383	THOC6	0.25163082929461	PKM.2	-0.504154871164979	RPS3A.3	-0.388512672485819	MLF1	0.348188184003418
TERF2IP	-0.373578384125696	MRPL12.2	0.383864207913395	HPRT1	0.273798447140464	SNX3.3	-0.36247023002927	MYDGF.3	0.569866710277534	SCP2	0.380206033506968
CSTB.1	-0.287782314356861	HDAC2.1	0.315528905413963	ARID5B.2	-0.434044131373222	RSL1D1.3	-0.403899517351079	TMSB15A.4	-0.910133000553732	SNRNP40	-0.387543547148441
SAT1.1	-0.447747971378859	THAP12	0.274084102682163	CCT4.1	0.294437415764901	IL32.3	-1.18293456946448	GAPDH.3	-0.344002833003111	FHL1	0.422296736714265
C18orf32.1	-0.289346611800479	GNL3.2	0.365203731279861	CUEDC2	-0.324869757519532	PCSK1N.4	0.89001275929541	SCAND1.1	0.619488918442085	POU3F2	-0.535709600780224
KCNQ1OT1	-0.433433008995941	C11orf58	0.288119029988953	DDX39B.1	0.291531924715012	CMTM6.1	-0.301528537605188	B2M.6	0.419652820601495	NUCB2	0.390014951784425
NQO1.1	-0.533323094460695	UQCR11.1	-0.314128074553174	BEX4	-0.417151989314158	MIB1	0.439688887806951	ACAT2.4	-0.772644743273289	TENM3-AS1	0.28188612070818
ELAVL3.1	-0.457190050180613	RAB2A.2	-0.350254275019197	MACF1.2	-0.536659601108105	FZD2.2	-0.294483927607955	RNF7.1	0.527901478997892	HNRNPA0	-0.408770037828733
ARL6IP1	0.296495116745498	MRPL57	0.289613907298035	VGLL4.1	-0.437083447531955	Jun 04	-0.45049580654777	EMP3.4	0.75142686643915	U2SURP	-0.403716123249314
SRGAP3	-0.324157590956838	NDUFAF2	0.302014239501782	PGK1.1	0.298888014717779	RNF24	0.317934682861914	RACK1.1	-0.434259253059692	SGPL1	0.275035517485833
TMEM158.1	-0.404417698332835	TSPAN6	0.304512636343143	TCAF1.1	-0.461626515564273	HDAC2.2	0.359694063870962	MINCR	0.629700068582774	SLC7A11	0.341945385687485
CDKN1A.1	0.414773006752922	GUK1.2	-0.375740681113311	FRMD4A.1	-0.403125979463231	ATP6V0C.2	0.417197240810268	EEF1D.2	-0.59521474076031	ANP32A	-0.391061207394239
ATP2B1.1	-0.353940858660813	GABARAP.3	-0.392187537560955	CSE1L.1	0.288149298033006	NELFE	-0.298728346227282	LMNB1.3	-0.6610265332282	PURPL	-0.366230778053884
A2M.1	-0.540390898414866	ARL4C.3	-0.714454920847969	PCGF5.2	-0.353421663848192	USP1.2	-0.342446233769032	NUSAP1.4	-1.01635816992217	AGGF1	0.299908991233056
RND2.1	-0.296049086413141	AKAP12.3	-0.725498634531484	IFITM3.2	-2.23132140352763	ZNF428.1	0.585635108974919	TCEAL9.4	0.727551025674474	SNHG16	-0.402256514000607
TMEM59.1	-0.258130621901416	FSTL1.2	-0.425209656277361	SELENOS.1	-0.385658129176268	FBXO5.2	-0.324741580481553	RRAGC.1	0.649024794286748	SEZ6L2	-0.474057998256102
COL4A1.1	0.303756296979007	CHD1	0.347159471755693	PHLDA1.1	-0.546066149642108	CKLF.1	-0.387290166785615	UQCRQ.4	0.487400790493276	TOMM40	-0.460971689451437
CALB1	0.30615948970471	FUBP1	0.366990272820468	HNRNPM.2	0.320011094241127	ALDH9A1.1	-0.328441477244677	SYNE2.2	-0.662599754747768	PRDX5	0.282117130582322
CD47.1	-0.325164163749261	EPB41L2.2	0.28929446504532	CNIH2	-0.410717722271352	ZNF511.1	-0.304642464921742	NREP.4	-0.769944687483475	RAD51AP1	-0.360610671044279
VCAN.1	0.280554649604143	PSAT1.1	0.323018017083569	CCT2.2	0.277142760777616	KMT2E.1	0.442309132152982	PHGDH.4	-0.724748882966203	RAB22A	0.269263860659172
PPP1CB.1	-0.348947280796997	SRM.2	0.351434936994441	SNHG14.1	0.318961817215608	KIF3A.1	0.497501121670361	RPL13.2	-0.373034682664777	WARS	0.544539260307796
PSME2.1	-0.397058999408523	SH3BP5.2	-0.370889329123461	FYTTD1	-0.353106797452702	TACC1.2	-0.318520897380049	SAP18.1	0.570972709852235	KDELR2	0.365231637732425
TM7SF2	-0.255926699011816	TFDP1	0.301379176417946	PRPS1	0.264015175017083	MCUB.1	-0.282030528790676	SAT2	0.71412343854814	PREX1	0.325223343155983
CRABP1.1	-0.728362280138309	PLS3.2	-0.431189033369584	PFDN5	-0.254211475880418	MAGOH.3	-0.327840518595305	CENPU.4	-0.617433788844282	NDUFAF4	-0.397105845234128
PDLIM4.1	-0.406034135242838	TMEM47	0.269931024098931	PTPRA.1	-0.385060145731875	ARL1	-0.292419301151838	RPL11.2	-0.362022835921101	TWF1	0.302611323018572
FABP7.1	-0.532439756855033	SELENOS.2	-0.352735589302716	JADE1.1	0.274196809473602	MID1.1	-0.318557836028537	CENPN.5	-0.564678666128897	HEBP1	0.306307110401054
MAP6	-0.294654416066255	SNRNP70.1	0.353066286643195	TGIF1.1	0.280019658873278	CCDC47	-0.297206030809292	RPS11.1	-0.387370487913808	GNS	0.280603947483378
EIF4A2	-0.309285387681812	MRPS26.1	0.339456692454222	SNRPC.1	0.307760645053403	CDKN3.2	-0.319348906841418	CENPK.4	-0.576685938018816	TPD52	-0.360023956327827
DPYSL3	-0.254883614633663	PPIB.2	-0.371978016506216	ENAH.2	-0.498993731196122	ITGA6	-0.270324073618935	GINS2.5	-0.640271981402507	SEMA6A	-0.503892186136955
KIF5C.1	-0.542097478383573	GABARAPL2.1	-0.398623995693244	PNO1	0.274117268139398	KIF1A.1	0.518932706622742	HSP90B1.2	-0.710395708886045	MAD2L2	-0.387385357503504
SNHG8	-0.298350014982168	TOMM40.3	0.392267082857822	CSNK1A1	-0.345536151919514	ISCA2	-0.290923831418793	CDK1.6	-0.763971838194587	GCLM	0.348514894122759
NKAIN4.1	0.327698468793357	NES.2	0.507016071205795	PIK3R3	-0.49161021207173	MAGOHB.3	-0.31554813057281	TAF7	0.843226946210167	PFDN2	-0.449768547236251
TSC22D1.1	-0.331108718592815	PPIF	0.36596509508559	MRPL21.1	0.336925837443208	ATP1B1.2	0.701630068831202	CLTA	0.497590781253907	TMEM134	0.371387196400343
MT-ND4L.1	-0.381134180806688	HADH.1	0.262634322022554	ANKRD12	-0.45010558909858	MICOS13.3	-0.367598934079847	PCLAF.3	-0.916771185284507	SGCB	0.339090487872527
CCDC80.1	-0.348388342526984	SORBS2.3	-0.484249424173131	TRAPPC5	-0.32569042132254	DNAJC3.1	-0.328423599068246	HMGB1.5	-0.724975541439232	CITED2	-0.418705395083131
TIMP2.1	-0.401385935893199	ATP6V1E1	-0.353363253343925	MMP24OS.1	-0.427648613133974	DNPEP	-0.271680374071942	RRM2.4	-0.680317425882162	PNRC1	0.461489887133959
B2M.1	-1.10044158580345	RNF181.1	-0.347020467880753	PAM.2	-0.31287773874742	SESTD1	0.413377816704326	SNHG9.1	0.869094175416128	COMMD3	0.316643599698696
TERF1.1	-0.345001507731238	MED28.1	0.296659755135289	RND2.2	0.289805980583306	SMS.3	-0.615153499360788	BZW2.4	-0.640608288999324	TMEM59	0.405339426126365
SQSTM1.1	-0.500821434930727	CCDC14	0.264100807101062	TUSC3.1	-0.388405006842028	ACVR2B.1	0.357364276247842	CENPW.5	-0.600359474451364	RNF13	0.324789502964955
STAT1.1	-0.270303879585008	ZFP36L1.2	0.278398585109432	PHC2	-0.325869437403035	EIF2AK4	-0.31119551310062	IGDCC3.6	-0.867202012329625	H1FX	-0.490555209508368
MTRNR2L12.1	-0.904968089907022	DDX50	0.272244266409884	CAPN2.2	-0.348186979800981	CRISPLD1.1	-0.300613055513421	PDIA6.4	-0.533258969366029	PTTG1IP	0.365867261712595
EZR.1	0.250513794201245	PUS7	0.26103015969831	COMMD6.1	-0.291187961753095	UCHL1.2	0.612412583925944	PARP1.3	-0.74777110380181	LBR	-0.460076712827123
STMN2.1	-1.6567097535949	SLC16A9	0.294839954884352	CDH11.1	-0.426539268553575	TPM4.2	-0.487096680323375	CYBA.5	-0.710842333813402	PRIM1	-0.331523424942943
NEFL.1	-0.461901664363312	EPHX1.1	-0.335842729584833	SAP18	0.260428660814417	COX17	0.345503004326355	LIN28A.2	-0.756326925309879	MSRB3	0.266588032194945
RNF19A.1	-0.348553485372688	THOC3.3	0.252432947164191	CCDC50.2	-0.431859957049698	NUP37.1	-0.27693168628417	EIF1AY.5	-0.557060310409876	CDC42EP3	0.341773459358746
NDFIP1.1	-0.25173994663007	POP7.2	0.351521287643404	KRAS.1	0.281633406605662	HNRNPC.3	-0.33419520622805	TSTD1.6	-0.712363494782069	DNAJC15	-0.422588066316075
LDHA	-0.274570404677126	RIDA.1	0.276331656631817	EBPL.1	0.280932233579561	CCNB2.3	-0.295473694214464	DLGAP5.4	-0.716421274639224	ABCA1	0.384528982691323
PTX3.1	-0.504265532586914	CD151.2	-0.444982837799047	IFT81	-0.318523188568011	TXNDC5.1	-0.293863839246327	ALDH7A1.2	-0.54328239055126	GCSH	-0.381497950441855
C4orf48	-0.258812455234621	BNIP3.1	-0.384558002850675	CDC42EP3.2	-0.382647642053936	PLCB1	0.371405483282812	CENPF.5	-1.61644238389408	RNF181	0.363479739240065
GPM6B.1	-0.613300871488523	E2F3	0.255748395511654	CXXC5.1	-0.476463188109064	HMGB2.3	-0.406177866481705	PYURF.1	0.619292477446431	C11orf49	0.276423533612975
MT-ND1.1	-0.440008804813635	CLNS1A	0.377141824220294	CCDC58	0.273543552694918	STOML2.1	-0.330077710332489	NUDT1.4	-0.586041041179072	CD82	0.341365985178489
MT-CO3.1	-0.386822932466965	RBM28	0.317950847558195	SYT1.2	-0.859327041416386	PAFAH1B1.1	0.450266446933588	HNRNPA2B1.3	-0.647053613070667	XRCC6	-0.496030187139435
SCG3.1	-0.403452836418782	SYT1.3	-0.769739850597489	RNH1.1	-0.326866426183373	NAV1.1	0.449295830542399	C4orf3.2	0.542984473415046	HADHA	0.360218788634641
INA.1	-0.565374730008708	GLOD4	0.307250522511022	ODF2L.1	-0.337317320345039	EEF1B2.3	-0.330356068720508	MRFAP1	0.559162358734842	GLRX3	-0.390258570957758
TUBB3.1	-0.278796182321973	CDK5RAP2	0.256936435795704	ATP5IF1.1	0.254996047080575	SNRPD2.3	-0.29763045422328	HMMR.4	-0.743972155981365	PPM1G	-0.387054712141303
MT-ND3.1	-0.295188035862935	S100A16.2	-0.446822925280135	TRIM28.1	0.381740215198505	ATAD2.2	-0.316743922857366	NCAPG.3	-0.597058855011887	THYN1	-0.378870722244607
PTN.1	-0.303824779576079	CAMK2N1.3	-0.767277625415636	ALYREF.2	0.266567115363186	HMMR.3	-0.449974275297364	SRPK1.3	-0.588041996629025	RPL10A	-0.292378754241707
TSTD1.1	-0.269623348341629	TMEM106C.2	0.28684033261231	NOLC1.1	0.303967930384665	ZNRD2.1	-0.353301182043145	TYMS.4	-0.862607216496637	SLC25A5	-0.372607352311211
STMN1.1	-0.265970107154586	PGM2L1.1	-0.450030353893438	AKAP12.2	-0.860366642653733	GTF3C6	-0.314702822723685	SMC2.1	-0.56633514449925	SOD2	1.8505322727402
NNAT.1	-1.0289672100595	NHSL1	0.274673138837825	PAPPA.1	-0.430662775870451	DBN1	0.426922092055798	MIS18BP1.4	-0.598343623658863	SRSF10	-0.380136848150582
SYT1.1	-0.504375458500386	DDX39A	0.29523865722484	NIP7	0.270105618461623	HECTD4	0.299883301805582	DPM3.1	0.632964916281503	MTF2	-0.388056853125755
CD24.1	-0.34463221593959	RRP15.2	0.299001983305921	FUS.1	0.291328289879251	ATP5PO.2	0.284713621412508	RPS15A.2	-0.326155212170116	NR2F6	-0.397808674260343
MT-CO2.1	-0.25001684056697	ZNF462.1	0.308520675472024	UPP1.2	-0.567759452818549	TP53.1	-0.296636529623984	CCNA2.5	-0.603856707915307	CHMP4B	0.313467103201656
MYC.1	-0.885574024515731	HMGXB4	0.274112143019104	RAB34.1	0.286385976858113	WDR34.1	-0.34022649477325	MKI67.2	-0.808042099208779	RPS21	-0.263194230439711
NEFM.1	-1.40611865567589	MIR4435-2HG.3	-0.386489141725825	ERP29	0.282252643558496	CECR2.2	0.340823974812768	PIMREG.4	-0.592722372389583	ATP6V0E1	0.346999368808445
PLTP.1	0.315552925880955	PAXX	0.298004725863002	PPP1R15A	-0.428271070321833	FAM174C	-0.305280721543698	AP1S1.3	0.572875397765308	PKIA	-0.479861228392535
PLCG2.1	-0.394357371228623	UTP18	0.28712999789692	PIK3R1.1	-0.386029955901021	YES1.1	-0.291760147218449	SEMA6A.3	-0.684017231197757	MT-CO3	-0.707152920296314
MTRNR2L8.1	-0.250126818232792	IQGAP1.1	-0.339337204026684	LAMP2.1	-0.326082644181526	DNAJC2.3	-0.325014627777561	RPS7.2	-0.34210374220142	PCNA	-0.456765846464793
		VDAC3.1	0.298611598435164	AKIRIN1.1	0.314378675794102	KIF11.1	-0.306534783311929	PSAT1.3	-0.654637696449315	NFKB1	0.290870514833658
		DHX9.1	0.395998909923959	ATP6V0B	-0.378961813894009	UGP2.3	-0.443714610897382	CLSPN.4	-0.661559768517909	JUNB	0.313141902796915
		SMARCA5.1	0.322395022249092	DRAP1.1	-0.307480966538741	CUTA.1	0.346018536520501	TOP2A.4	-1.10842662048113	ELOVL1	0.266292705241621
		MPST	0.298784992474839	AK1.1	-0.382355369868273	TMOD3.3	-0.347281319762487	CDC20.4	-0.687990228226389	PLSCR1	0.316284703108407
		AHCY.2	0.411521902032658	ANK2.1	-0.543658682262161	NAA50.3	-0.315496139772546	VRK1.4	-0.526134763765575	C12orf45	-0.396561176494001
		GNL2	0.285275484842418	NTHL1.1	0.283034647784354	CTSL.1	-0.291595926702953	CCNB1IP1.2	-0.515862882020609	ETV5	0.290014525901144
		COPS3.1	0.302366298070203	SLC39A6	-0.329615939176335	C1QL1	0.363060200820637	SNHG8.2	0.937748539304806	SMARCA5	-0.395181055306852
		TGS1.2	0.297964317521903	LIX1.1	-0.429924344798432	SLC7A5.1	-0.358797873416599	SLC25A5.3	-0.47096168462719	MGAT4B	0.351062423970136
		MGST3.2	-0.341986548111569	DUSP6.2	-0.450823468515478	KDM6B	0.326661797611764	Jun 06	0.782877208992019	FRMD4A	0.366538933234498
		KNSTRN	0.262527269015251	PSME1.2	-0.581236585987621	GNPNAT1.1	-0.280160109712058	C19orf53	0.441707478464905	AP1S1	0.405719762760983
		CIAO2A	0.262157028774171	SNRPD3.1	0.284493760465014	ATP6V0A1	0.397527873115664	MYBL2.4	-0.521255135575171	RAB31	0.418025402931124
		NUDC	0.347496490762406	MCM7.2	0.262279859021039	BEX4.2	0.435615676689129	RPL17.4	-0.354566919491486	MYL12A	0.333988139910662
		BZW1.1	0.272383084761896	ZKSCAN1	-0.381371905054978	LARP6.1	-0.309341118007185	GNG4.4	-0.64444635512444	C6orf89	0.253888803027229
		SYT11.3	-0.398742782929441	TMEM158.2	-0.725051948336577	TSPAN6.1	-0.304684338868365	RPS2.4	-0.473759924863985	ERLEC1	0.389554131485782
		ADSL.2	0.29798402535832	WSB1	-0.500318420832386	MCUR1.1	-0.278501936082878	MND1.4	-0.499002480377469	SLC43A2	0.45304371184759
		PDPN.1	-0.444903496063818	CDC123.1	0.289418966166128	CETN2.2	-0.286624141575146	IGFBP2.5	-0.728308959722269	RRP15	-0.376106501770082
		PHF14.1	0.306928928395437	UBE2H.2	-0.432661685092224	RGS3.4	-0.455328554906174	RGS10.4	-0.647431136171427	CDKN2B	0.349247401097481
		BAZ1B.1	0.374073728459496	TRIM44.1	-0.360716425013589	MIS18BP1.3	-0.343247006798842	TMA7.1	0.436241654563428	CDC6	-0.340999516128222
		NOP14	0.302119701649077	MRPS18C	0.290488228407861	NDUFAF2.1	-0.295596426674318	GTF2H5	0.538525253516657	RSL24D1	-0.349610007343288
		ZNHIT1.2	-0.333905748373443	ALCAM.1	-0.443116636175091	GINM1.2	-0.307656253070897	COA1.3	-0.501339440137653	DNALI1	0.26499713619814
		LUC7L3.1	0.280475238949972	MLF1.1	-0.329961242876045	SDF4.1	-0.312844009787852	KCNQ2.2	-0.561324559321903	PHF14	-0.404002055416735
		RABL6.1	0.406001245916095	ITGAV.2	-0.455263763694922	FAM120A.1	-0.299188986992416	RAD51AP1.4	-0.480940198683512	COX5B	-0.339849940921358
		GLRX3.2	0.273305185902462	GABARAPL2	-0.386037793081085	ROCK2.2	-0.341913910349889	SMIM29.1	0.491510396886394	GALNT2	0.397464447265283
		DHX33	0.274160172400125	EIF1.1	-0.322159757623468	CCNA2.3	-0.290385881097599	DBI.1	-0.413693837356813	MAGED1	0.448307232838023
		ELAVL1.2	0.297504798200393	CCDC82	-0.350383019949051	LSAMP	0.383730879061075	RPS17.5	-0.350550118866253	PBX1	-0.608087437765771
		PPP1R15A.1	-0.395395891884712	SF3A3.1	0.280363959678178	LRPAP1.1	-0.333822543473535	POMP.1	0.449906436496236	RBM17	-0.362408367395765
		IPO5	0.367076152742649	MAGOHB.1	0.276776031203284	BARD1.2	-0.284058787738361	ZIC1.1	0.515673415750302	LITAF	-0.452824598385525
		CASP8AP2	0.268389095535968	SGCB.1	-0.358443965894802	MRPL57.1	-0.314288495279079	DBF4.4	-0.527044483372453	NPM3	-0.40677126913017
		TMEM219.2	-0.332276110177489	TCP1.1	0.288169282504547	RHOBTB3.2	-0.405101545266664	CSTB.5	0.637117032565365	ACOT7	-0.390072155386501
		TRIM24.1	0.328265373826481	ENSA	0.295084600869017	SNX7.1	-0.259183524701687	HMGB2.5	-0.969021893764845	VGF	-0.38447133516998
		SMIM14	-0.304549503576785	OAZ2	0.269447964477874	GTSE1.1	-0.306956355644509	CDKN3.4	-0.588438747754485	ARID5B	0.365036018021882
		DCAF13	0.294484699837746	AHCY.1	0.363483882992245	SDF2L1.1	-0.392417810772638	SCG3.5	-0.75797618937178	NRCAM	0.336561131516865

HSPH1.1	0.457237056780414	MYH9.2	-0.545420623287763	DERL2	-0.271814904986107	ADM	0.590491943841894	NME3	0.405104642462754
ATIC	0.33103128877528	MIR4435-2HG.2	-0.365384757677921	TCF7L1	-0.257249283462902	FABP5.3	-0.659816735830611	STIP1	-0.459809584440472
PLAAT3.1	-0.345024055470792	C9orf16.1	-0.383358847340062	NOP58.3	-0.355519738692702	TMEFF1.2	-0.616736727510317	DDX18	-0.364068484066235
PTBP1	0.326743143085467	XIST.2	-0.568487931480236	LGMN.1	-0.312033897171839	RAB32.1	0.481460152009365	PRMT2	0.3527599990745
RBM25.1	0.312302854937706	ECI1	0.283400534879995	LIMA1.3	-0.34701382767486	SNHG30	0.596115366193078	HNRNPK	-0.364422400909104
BMP7	0.290345563484114	C12orf76	-0.303857709814429	PMEPA1.3	-0.38478757087435	SEPTIN11.3	-0.516910716530796	NHP2	-0.366602955713638
RPF2.2	0.282615183469268	FAM207A	0.282414984582294	EMD	-0.28758510703437	TOMM6.2	0.509717538933737	CSE1L	-0.39610771948071
GNPNAT1	0.262598753016066	BFAR	0.256939928988549	CYFIP1.1	-0.309633560591206	INPP5F.4	-0.547254551393306	WASF2	0.354119729761958
SFXN1	0.280983380326887	LEPROT.1	-0.351131389731394	MESD.1	-0.298852973092896	TRMT112.2	0.508106599766784	UPP1	0.405355268005958
PGP.1	0.339421395816266	VASP.1	0.268009727768073	AK6	-0.281962145965265	MYH10.4	-0.699531753563852	SRM	-0.413848736980829
MAP1A.1	-0.354402494997316	RBIS	0.262419434934001	ZWINT.3	-0.310766284151737	SERTAD1	0.836462498006749	LAP3	0.618800056910167
NOL11	0.279916979985077	RPL39L	0.276529460141276	RPF2.3	-0.308220560745419	GMNN.5	-0.592723900821881	DLGAP5	-0.341156854318783
CSTB.3	-0.366677413144824	MRTO4.1	0.291235949111024	ISYNA1.2	-0.37851592018754	MYL12B.5	0.438777985272676	NDUFB9	-0.330736485738314
POLD2	0.328153276180666	REXO2.1	0.290770270662425	CELSR2	0.265398026285723	GAS5.1	0.859533689056932	NDUFV2	-0.332504268869922
WWTR1.2	-0.346530527854081	EBNA1BP2.1	0.308771476094099	CENPS.1	-0.278301127715849	LIN28B.3	-0.459342934832446	EEF1E1	-0.363077374487288
THRAP3	0.305853611587696	MORN2.1	-0.341857344948597	WDR43.3	-0.323169281881785	ACOT7.3	-0.488080060637671	DRAXIN	-0.387079922443448
TMA16.2	0.276007628156651	LINC00472.1	-0.367571691855369	NASP.3	-0.513849974766357	SEC61G.2	0.430394500714915	TSPAN13	-0.36950328244643
MYH10.2	0.365274254022598	CRMP1.1	-0.528267780646595	LYAR.3	-0.381383428908556	AURKB.4	-0.531067713838035	SEPHS1	-0.442512101665368
NEFL.3	-1.26425856725358	FAM162A	0.295110229060474	MRPS12.1	-0.331377137844883	RPL18A.3	-0.356038624681214	ILF3	-0.400580896504436
AATF	0.285681565683555	C4orf3.1	-0.332453753763784	RCOR2	0.277189349474238	EZH2.2	-0.50522013531784	GRN	0.434865647909998
CYB5B.1	0.303462719002564	SH3GLB1.1	-0.359176579525781	PTBP1.1	-0.319172146983065	SFRP2.5	-1.24538638416593	PPP1R1A	-0.496851232893387
SGCB.2	-0.322674369290001	COL6A1.1	-0.393678115796166	MCM10.2	-0.287042797545972	RPL26.2	-0.29795434000314	H2AFV	-0.344829321953586
CCT7.2	0.314271357070991	KLC1.1	-0.50948243419294	AMD1	-0.309902444908906	UBE2C.5	-1.08009516110627	CASC4	0.314943579693658
URI1	0.331549854649718	TMEM263.1	-0.28613162564533	EIF3M.3	-0.328320996790306	EIF4A2.4	0.659878578581894	CARHSP1	-0.37196795708404
TCOF1.3	0.326500083091654	NEK6.2	-0.286338381911499	PIN4.1	0.3277387898429	MT-ND1.3	-0.821903507706308	NTPCR	-0.372218248186449
IMPDH2.2	0.376500692271443	ILF2.1	0.268036484890393	TIMP2.4	-0.532693560970621	GNAS.2	-0.646118506714403	PFDN6	-0.350871692065174
VKORC1.2	-0.300981211432075	NFE2L2.1	-0.378416140206887	ASPM.2	-0.321370443974158	EPB41L2.4	-0.453403162603093	PAM	0.270393683709335
TERF2IP.2	-0.452422500458566	TFDP2.1	0.263384290483611	DNAJC10.1	-0.295098793742061	SCAMP1-AS1	0.373376652864582	GTPBP4	-0.396657114753203
CTCF	0.304690212620932	PDHA1	0.269824608819152	EIF4G2.1	0.388756001701809	ORC6.5	-0.518303758528306	SELENOH	-0.359028547358809
ECI1.1	0.292724886933661	GRINA.1	-0.324248337399761	MAP1A.2	0.396231277975573	CLEC11A.1	0.555559587026383	FBLN1	-0.404086807727968
KDELR1.2	-0.303033056135827	METTL5	0.294036406057187	EPB41L2.3	-0.296707073781429	DNMT1.3	-0.517575977841454	PSMB10	0.392660829854439
IPO7.1	0.44612177435085	SSR2	-0.275862455499878	PFDN4.2	0.346458428740739	VCAN.3	-0.64388545250073	SNRNP70	-0.417994487744824
MTHFD1L.1	0.271809837614367	YWHAE	-0.257904914074526	MSRB2.1	0.394347478838436	ZWINT.4	-0.463505296870521	CAPNS1	0.372269965296697
NPDC1.3	-0.342937543391363	COA6	0.274156215718158	MGME1.1	-0.268463925638401	GTSE1.2	-0.532920435406587	SSR4	0.303439507378307
NT5C3A	0.251091366690208	ELP5	0.263442280903062	LUZP1.1	-0.312383103217766	TRIM71.2	-0.480032894317546	MRPL47	-0.350092617101949
KHSRP	0.294428279203269	TCEA1	0.274997386175607	MCM7.4	-0.403203620212686	SF3B5	0.456681929061269	NSG1	-0.541623502112736
STIP1.1	0.458535472702177	TALDO1	0.260887176315114	PODXL2	0.445273070191212	GABRB3.2	-0.482827786468347	MAN1B1	0.250679400850642
RPA1	0.267792632400429	CDKN2A.1	-0.418293160994883	CD3EAP.2	-0.287404923961478	CARHSP1.3	-0.491266652856622	ETFRF1	0.287851422244065
HES4.2	0.320666046252099	KIF5C.2	-0.719823039336353	FANCI.2	-0.265474899519656	ABRACL.3	-0.484104376446096	FAM120A	0.290947975977949
APP.1	-0.38868411284351	SOX4.1	-0.60629019269762	TP53I13.1	-0.269830823015718	SYF2	0.627283069724003	SIPA1L2	-0.362648519558068
GDI2.1	0.273930113932271	BORCS7	-0.320471826022943	LAMP2.2	-0.290454857060669	GADD45GIP1	0.457880973451362	PSMA2	-0.325245190713429
BAZ1A	0.390546569316539	CASC4.1	-0.319331041914707	GLO1.2	-0.336901345491046	MCM3.4	-0.544569890372771	NDUFB10	-0.385937540722975
KDM1A.1	0.32140845165267	F2R.2	-0.358240279249421	FEZ1.2	-0.282363977356717	EPB41.3	-0.54709317117385	GDI2	-0.360097402107901
TCEAL9.2	-0.395953847148776	TAGLN3.2	-0.984396044110755	CSNK1E.1	0.44023955248256	RBM3.1	-0.407672284111005	POLR2H	-0.380171128286812
MICOS13.2	-0.347089871823417	ATP5F1C.1	0.257028276727888	RFC4.3	-0.29834825858882	H2AFV.3	-0.462047066450273	CHAF1A	-0.328970615200504
FAM133B.1	0.289127368272114	PSMD7	0.261771323251716	MRPL3.1	-0.311446475525241	SNHG6	0.555086456911597	IFT57	0.354142971216766
EBPL.2	0.295485196411777	FXYD6	-0.398371434707549	AHCY.3	-0.394229583096865	EIF6.1	0.485122529529024	PCBP1	-0.34713350193368
TRMT6	0.261487005057461	RIF1.1	0.258802121386472	CCDC59	-0.299609114746861	SFRP1.6	-0.91947007281201	KIF20B	-0.366887591994067
UMPS	0.259930242027377	TCTEX1D2.1	-0.316532725315906	IPO5.1	-0.334144018675257	HIST3H2A	0.589576969639586	TUSC3	0.352264475507421
YPEL5.2	-0.317868213224731	PELP1	0.252306439357225	DCP2.1	-0.290805055989956	MRPL33	0.498280127875881	C11orf1	-0.35357070464735
MAT2A	0.299946591637122	RO60	-0.326269847976363	ANXA6.2	-0.297840478290834	FDPS.3	-0.583212348344782	MZT2B	0.263000938373332
JPT2.1	0.274690944112944	NGRN	-0.347837299638695	NSD3.1	0.43855956823841	REEP5.2	0.483591776289964	PLXNB2	0.278505834444982
ADAM9.2	-0.361069632445862	TSPYL4	-0.330573922619136	DCTN3.1	0.385030940419772	ASPM.3	-0.648793024782175	RFX4	0.284522181086536
RGS3.3	-0.388922358584379	IDH2	-0.275325548540827	POLD2.1	-0.30498141447993	SPCS1.1	0.452935493869189	SRSF11	-0.339973999734104
MRPS15	0.299489087182584	PSMG3.1	0.260368587925839	APRT.2	-0.387368574753078	SNRPA.3	-0.454351170583612	BRI3	0.299603473238902
ACTN3.3	-0.509528129284965	PCNA.1	0.275606699500477	IARS	-0.304188321163328	TSPAN18.3	-0.427555585696667	TMED9	0.382824890433991
VDAC1.1	0.262304469166678	TXNDC17	0.250700035255819	KIF9	-0.265071584323733	DPYSL2.2	-0.572242803578946	HMGN5	-0.347740473547377
BEX4.1	-0.351352905566126	PRAF2	-0.296070883517875	M6PR	-0.277168546677537	IDH1.2	-0.466172388792055	STK26	-0.31978849435652
ALDH7A1.1	0.295814575591481	KIAA0408	-0.331047046178072	CCNG2	0.288205334149037	LSM4.3	-0.46716150912763	COMMD6	0.277495802986723
TP53	0.253403412571674	NORAD	-0.411463669515829	CD320.1	-0.324167150763872	KIF20B.4	-0.515325972812803	BLVRA	0.284754031852748
PDP1.2	-0.313709182290237	PRELID1	0.287803183089741	FAM136A.3	-0.37000096887109	EPC1.1	0.700170817289561	LIMA1	0.373704398677665
UPF3B.1	0.285399917565093	RPS2	0.275517779752656	BRIX1.3	-0.303962479516487	DNAJC8	0.518759761908633	GRINA	0.330961987609762
ARGLU1.1	0.26395718093899	YIPF3.1	-0.365257439616395	TMEM59.4	0.324261173121979	UBA52.2	-0.315221765674777	HAUS1	-0.360980854517469
COX20	-0.31913949972981	PHB2	0.281489887092051	BRK1.1	0.324317637288996	SMARCC1.2	-0.566150612807102	MRPS9	-0.36558839304251
ARMCX3.1	-0.358225979403455	STUB1.1	-0.308240127559939	POLD3.1	-0.261485025513285	NUF2.1	-0.49118221812172	ANXA6	0.318074801651849
SSR4.2	-0.300456272164937	NBDY	-0.30769045091174	PBRM1.1	0.419330808660186	SMC1A.2	-0.492838626289155	POLR2I	-0.337991121177507
PSMD11	0.272089610350618	ACTR2.1	-0.334065304301628	EIF2S3	-0.322146517408207	SEC62.2	0.444976307129604	RRAGD	0.316503621638818
TARDBP.1	0.293884484961479	CDC42.1	-0.299806444647006	PCMT1	0.358916682398923	ECT2.4	-0.454762127337125	RPL27A	0.259508382471047
PRELID1.1	0.255531457215738	BMPR2	-0.317862546043468	AKIRIN2	0.421227480010186	COX5A.5	-0.418003363805368	TP53TG1	0.279578256243763
NPM3.2	0.300015883827028	PMF1	0.26430104098502	C9orf116.1	-0.261405351458582	TRIM56.1	0.419535251600649	GLUL	-0.410790298519805
COMMD4	0.308728529307211	GLRX	-0.383407668731388	SMIM30	-0.28544291035379	CAPZB.1	-0.42225447042592	ZNF462	-0.358921402056817
VAT1.1	-0.347685870846406	SPAG9	-0.36845184146901	RNF5	0.438415624409729	PRC1.1	-0.53524168593264	CD164	0.32392761551729
EBNA1BP2.2	0.344459120401127	ABI2	-0.33256830066463	ZYX.1	-0.323994434428853	SMIM26	0.5190802247555	RPL4	-0.361515567905977
MT-CO1.2	-0.447726746752132	DDR1.1	-0.375748794572541	GSTA4.1	0.411128021244232	SEPTIN10.2	-0.416493109873515	ADAM17	0.286535949283622
CPSF6.1	0.272156065431967	AC010642.2	-0.328597153075866	DPM3	-0.2843084572631	UQCR10.1	0.354353606862323	DHRS7	0.336034002983819
EMC7	-0.287167594498766	CHD9	-0.376145820811371	CDCA7L.3	-0.26153623357614	QPRT.3	-0.475072017844053	CFAP298	-1.27553750275847
GDI1.1	-0.375435472742106	MAP9.1	-0.320613203203445	PAPPA.2	-0.347131296678107	TCEAL7.4	0.598237427120699	MEST	0.446437839167608
G3BP1.2	0.32272589416192	REST	0.278427035543531	MRPL19	-0.272953677191956	JPT1.3	-0.443457765995405	MZT1	-0.35350589302898
CFAP298.3	-1.13707234895645	PNMA1	-0.306383531971766	NME1.3	-0.338102445076311	NDUFC2.1	0.408521917512239	CBX5	-0.374119821876342
DUSP6.3	0.257441966928493	AKAP9	-0.468888681881153	DPCD	-0.280138794061617	MCM7.5	-0.601683494102902	C4orf3	0.337380830829791
FAM92A	0.254882707147001	USO1	0.250259045718317	CMTM3.1	-0.273039909280847	RPS24.3	-0.295648250746031	NONO	-0.369467850135147
IDH2.1	0.31042172260568	TMBIM4.1	-0.362950021984689	CHAF1A.2	-0.276368832467312	RPL18.1	-0.272384304271949	SNRPD3	-0.346246508352092
FZD3.1	0.273033009411918	TCEA2.1	-0.330742033375665	SPRY1.2	-0.40005969177811	RAB14.1	0.473630672138058	SUMO1	-0.313476961390692
PSMD14	0.263405911607233	CNBP.1	0.273924130912182	HNRNPF.2	-0.321060191667649	HSP90AB1.4	-0.438449317001374	SDF4	0.351835845280222
PABPC1.1	0.272525637092874	MRPL18	0.275449356308987	SAE1.3	-0.297489814145353	CCNB1.5	-0.7883607227969	MAGOHB	-0.351970184469286
SHC1.2	-0.319725047602213	MDH2.1	0.30135136384155	TP53BP1	0.388038588428096	GCSH.5	-0.429198928534889	PPCS	0.26169242284202
ZRANB2	0.281384106929716	ASAP1.1	-0.339072807271048	COA6.1	-0.284494248713177	NDUFA1.1	0.439443304876746	SNHG1	-0.396670170510717
ATP5F1E.2	-0.317932859465323	PLK2.2	-0.431423793431396	RBCK1.1	-0.281244123188618	RPL23.2	-0.303793801281388	MRPS6	-0.355332183379544
RIF1.2	0.338350785653811	TMEM9B.1	-0.305462797070612	AAMDC.1	-0.280443370637908	RPN2.1	-0.426005639538007	CDK2AP1	0.366059233955164
LSM14A	0.282374286655689	CRNDE	-0.356082647803249	ANP32E.2	-0.333921692165662	FANCI.3	-0.389117716309361	EVI5	0.268097506205895
MZT1.1	0.266753654325578	EIF4E2	0.251116884871157	NAMPT	-0.302944575230071	CENPE.4	-0.646411553391481	HSDL2	0.292056428625496
DICER1.1	0.258601736554328	GAL	0.533061689315078	NECTIN3.1	-0.264896363537103	ZNF667-AS1.1	0.552122371204492	SAT1	0.511039569851626
CHPF.1	-0.278783783447772	TSR1	0.250350485036028	SLC25A37	-0.314947666575542	RRM1.3	-0.608003289347836	BUB3	-0.366466789672121
RUVBL2	0.274250299728751	CFL2	-0.28570189060511	CHMP4A	-0.25886122588987	RPA3.5	-0.587495227353213	AGTRAP	0.332587895943
EVA1B.3	-0.346209153107041	C11orf49.1	-0.252916796810606	NIP7.1	-0.272186477410944	CENPV.2	-0.74845401080005	EXOSC3	-0.356178204688599
TMEM123.1	0.289593935840779	UQCC3	0.285499760729695	PDCD5.1	-0.311885495767436	DDX39B.3	-0.41670210012157	RRAGC	0.271641302993055
EMG1.1	0.287299182123959	POLD4.1	-0.395400019006958	SZRD1	-0.255308746024824	SMC4.2	-0.620722476797171	RPL36A	-0.261114811759747
CCT3.2	0.420781195631241	CCDC107.1	-0.319266918490548	MAPK6	0.39341957352289	DHFR.3	-0.581436003000376	ARPC5	0.32936948023985
CCPG1.2	-0.350271540992837	NAA50.1	0.251704106882758	DNAJC21.2	-0.303929894471551	EFNB2.5	0.690944946415601	FAM229B	0.326481448923085
PUM3.2	0.287378716299345	INPP5F.2	0.255815407805989	DTD1	0.357711358999505	ATP5ME.2	0.410838527699466	ZNHIT3	-0.339072045132151
CD47.3	-0.43726024120946	NHP2.1	0.253647952910242	RPL36AL	-0.254742689474337	FTH1.6	0.88501756026283	ATP5F1A	-0.363307928195565
ME2	0.266869199064535	NFIB.2	-0.421171092792506	NDC80.1	-0.260225458773223	BEX1.4	0.369793278950694	TCERG1	-0.367831681698088
MLEC.3	0.309305218444063	MRPL32	0.256853230664014	CLNS1A.1	-0.304477982197534	NNAT.4	-1.72573281214541	PARVA	0.269138472422183
CST3.3	-0.44704794700324	PMM1.1	-0.279424168027658	NUCB2.2	-0.282299402653918	TMCO1.2	0.487252494759899	PLK1	-0.406239064675447
CEP57	0.274056762976154	GOLGB1	-0.4426884916717	CASP3.3	0.747996368791592	MAGI2-AS3.1	0.367540056188766	FAM216A	-0.341183028395666
KLC1.2	-0.445432604898155	KLHL9	-0.302054433695446	PGRMC1.1	0.449667304570768	NCAM1.5	-0.664011987128914	HEXA	0.288120872456216
SNRPD3.2	0.264939905085479	MAP1A	-0.384502938098086	TRIM2	0.414202969930355	RPS5.4	-0.327905016888438	AKAP1	-0.322825200177047
TPM2.2	-0.621238133294802	MYL12B.2	-0.317024119307002	RPA3.3	-0.408817051734984	MCM4.4	-0.494601430199807	RSL1D1	-0.410211652801371
UCHL5	0.352020769549497	DPYSL5.1	-0.280003925261651	FERMT2.1	-0.280020837977186	NDUFAF8.1	0.52732016560643	MCM10	-0.308070215703941
ITGAE	0.262646733058991	MRPL47.1	0.258828832624846	BCAT1.4	-0.380515267064411	NKAIN4.4	-0.581076999581135	DCUN1D5	-0.356051830990418
CHMP5	-0.285874921663383	CD81.1	-0.2733241204539	SKP1	0.275870160402589	IGF2BP1.3	-0.586494521707329	ZNF326	-0.343605763966791
ABCA1.2	-0.326771567528869	IFT22.1	-0.311186635268192	ABCE1.2	-0.30615163684988	SRP14.2	0.313143484539959	RGS16	-0.661537544024991
REXO2.2	-0.287048147952546	SEC14L1.1	-0.31917687172176	PBX3.1	0.39661259603786	CCDC85B.3	0.503672382824785	KRT18	-1.05461799617446
MRPL1.1	0.263549793803803	PDP1.1	-0.329193238366438	PGRMC2.1	-0.262082545174692	AC027031.2	0.355632378716582	C18orf32	0.305592381507795
MMP2.3	-0.381472315861921	STMP1	-0.281040715343761	SDSL.1	-0.280362747502216	UBB.1	-0.335021798861956	PLEC	0.259379992336533
COL5A2.3	-0.357990848705413	IFNAR1.1	-0.267295388352069	ALKBH7	0.312876684928776	CMSS1.3	-0.412062342960752	SYPL1	0.29906326056187
PRRC2C.1	0.372917398399163	BNIP3L	-0.32699570043165	PHF19.1	-0.262686919161006	RPS18.3	-0.275925420011784	SSBP3	-0.370856595364548
KRT8.3	-0.946113268502255	SH3BP5.1	-0.349581912138876	CKAP2.1	-0.305890153576376	RPL36A.3	-0.309395085140205	DYNLL2	-0.3687658687075
BEX2.2	-0.449454816787855	H2AFY2	-0.296270567702929	CCDC58.1	-0.260532686928873	ATAD2.3	-0.470934669469215	VAMP3	0.259241861091177
TRIM28.2	0.333497604940089	PTP4A2.1	-0.289230524724147	MRPL37	-0.286679172106519	MT-CO3.3	-0.638547023758375	PRDX3	-0.335700214019684
HIST1H1D.1	0.347888811219851	OSBPL8.1	-0.34717425356497	DYNLRB1.2	0.347934072393259	HIST1H4C.5	-1.56170603272942	DGCR6L	0.275777875170893
FASN.1	0.281612822353326	CCSER2	-0.273565169747741	IP6K2	0.364543571356173	RBPMS.3	-0.441313929074262	TOMM22	-0.340458867246594
SRP72	0.281531078418217	HERPUD1.1	-0.381276193194209	SMARCE1	0.321189446588926	PRMT1.2	-0.462837125391517	TOMM5	-0.420656103438017
SQLE	0.28981412413145	COA8	-0.288729765921728	DDX5.1	0.363330133352103	MTHFD2.1	-0.46388727486063	GJA1	-0.448611897673845
HMGCR.1	0.273757942744999	ZNF667-AS1	-0.289034598083797	NUDCD2.1	-0.271520519678656	PRDX6.5	-0.607840092664071	RAC1	0.251130946768103
PNRC2	0.278468346617686	MPC1.1	-0.308292363793223	UBE2L6.3	0.388585120531321	CSE1L.3	-0.423205200701284	TDG	-0.338473019718608
COL6A1.2	-0.359753570164955	G3BP1.1	0.254011589768938	UBE2C.3	-0.372179893170484	HSPD1.5	-0.681418736291202	TUBB3	-0.936116790902623
RRP7A	0.269673380448638	NME1.1	0.291426620706132	ADSL.3	-0.297764544113723	SDCBP.3	0.548406316598175	HADHB	0.327272507958356
NUDT21.1	0.262302784675438	RAB31.2	-0.399966204132075	CBR1.2	-0.274275676725439	MYL9.5	-1.09138334543912	GMPS	-0.379730100112776
CDKN1A.3	-0.483277733489683	LRRN1.1	0.298201076811493	PPIH.1	-0.271699201656771	SUMO2.4	-0.309277441188286	ATP6AP2	0.350521788576416
GAR1	0.268058464430721	MRPS17	0.27870142053702	FAM89B.1	0.413768224763787	KIF11.2	-0.412158964490432	NUDT15	-0.335214922992144
POLR2H.2	0.299901607578506	DECR1.1	-0.328768233678659	NCAPG.2	-0.268596882325473	LYRM2.1	0.4893357122397	AIG1	0.313527115215589
ATP5MD	-0.255222678698075	KLF7.1	-0.310843816139993	LLPH	-0.272588045362705	CYSTM1.2	0.662096915244437	CDK1	-0.385783704614797
GNG11.3	-0.416448674941385	TRIP6	0.262963594377908	CD164.1	-0.272652928924471	ZSCAN16-AS1	0.492647909762183	CAMK2N1	0.835239196720385
UBE2N.1	0.251609896974192	SFRP1.2	-0.637175082309196	RBBP8.1	-0.25549440766379	C18orf32.5	0.417085786646069	CDC123	-0.343957599723736
CLTC.1	-0.283103618299481	LIMS1.1	-0.420068726959825	CENPE.3	-0.367712227295682	ISYNA1.3	-0.490415679824964	UPF3B	-0.348577947976021
PANTR1	0.253281340551739	MAP2K2.1	-0.291020410698035	EVL.1	0.496982860519155	PRDX2.4	-0.492761129511589	VKORC1	0.32060862026057
SNX3.2	-0.300893408478106	NPDC1.2	-0.347076566692207	DESI2.1	-0.253977701660979	RPL7.2	-0.329729354672847	LYAR	-0.444754165276672
VPS28.2	-0.307001978036272	GNAS	-0.412498583483585	MSH6.2	-0.325236949762646	KPNA2.4	-0.706444250287476	RAB9A	0.304812724117219
DYNLRB1.1	-0.311317261281665	LPP.2	-0.422027824258195	NINJ1.1	-0.281500588381547	ATP5MPL	0.377503291896842	EPHX1	0.359265772553285
FRMD4A.2	-0.30470301747523	GOLM1	-0.310875417697122	DAG1	-0.250537828374496	HMGN3.3	-0.389045314030882	NCAPG	-0.340281490531646
RPL4.2	0.273610857095531	GRHPR	-0.259674913835089	PRKCI.1	0.35355927769886	TMPO.2	-0.565823122750295	PRTG	-0.598530991863938
CRNDE.1	0.267950358402406	SS18L2	0.250823142385973	ACTN4.1	-0.341695865979308	GMPS.2	-0.440363964261364	GNB2	0.323610408268862
KIDINS220.1	-0.424206020444712	AHNAK.2	-0.429681933647897	DDX21.3	-0.383842311933175	DEK.5	-0.615186569188213	CDH2	0.517945196890515
SCP2.1	-0.288724196129404	HNRNPR.2	-0.311767483737944	SERBP1.3	-0.34153129968319	FDFT1.3	-0.463415356282297	MARCKS	0.271361178874355
C20orf27	0.260383308613787	RIT1.1	-0.284966229166997	PPP1CB.4	-0.466366284237863	ACAA2.2	-0.39515266337495	CRMP1	-0.531292849461982
NFE2L2.2	-0.295637276499002	ARHGDIA	-0.328872146657723	DNAJB6	0.333297187173449	CALM3.2	-0.407556977451442	DCXR	-0.356774729453194
DDX21.2	0.319521772690614	HOOK3	-0.318552213656764	PSMA5	-0.290237573880324	TERF2IP.4	0.489548536540154	ATP5F1C	-0.31179876980643
PFDN2.1	0.266873774728544	MAP7D1	-0.267003036175512	HERC1	0.291393619516546	HNRNPA3.5	-0.4952989031359	DNAJC3	0.322629162869543
VGLL4.2	-0.329587926759104	TACC1.1	-0.287696130170874	ANAPC15.1	-0.263788276502735	FDX2	0.513469884268439	CNIH4	-0.360201972402882
ANKRD12.1	-0.327815461331122	DDX5	-0.327550063177425	MARCH6.1	0.383364828954141	EIF4EBP1.3	-0.557382852267647	METRN	0.442732564833446
YIF1A.1	-0.253654509850572	SH3BGRL	-0.323431030253728	ELP5.2	-0.270842967012195	STMN2.6	-2.37901723028785	IFT43	0.302161229370351
PSMC3	0.282846011489837	MORF4L2	-0.341029431205369	PDXK	-0.271057529434987	TMEM208.1	0.461440853438441	REEP5	0.327460908920484
CKAP5	0.338153776968534	NRIP1.1	-0.336275127593921	FDXR	-0.387209567084047	H3F3B.3	0.586522151847658	DPY30	-0.320822696586378
CTSD.1	-0.312523746230519	IFT57.1	-0.296606419791216	SPINT2.2	0.262337449925758	EIF5.3	0.599123139075293	ZWINT	-0.303731295351265
EIF2AK2.2	-0.318585136699384	SLC25A39	0.27366244016802	TCF12.1	0.43891476190086	PTPRZ1.2	-0.49474515251215	PDGFB	0.276485589779744
MGAT4B.2	-0.283344607099915	C1orf54.1	-0.275883261232726	HDGF.2	-0.34485731387421	CNTFR.4	-0.371327136454235	BCL7A	-0.318773711317788
ARPC5.3	-0.294349772259983	VMP1.2	-0.379861597606596	TMBIM4.2	-0.299572975690759	HNRNPD.4	-0.530764016172897	DHFR	-0.446432969996138
SLBP	0.275279693046891	NDN	-0.318645379651201	GAMT.1	-0.310146270728139	TMEM97.3	-0.506540795496542	SAE1	-0.353904535013704
RBBP4	0.251796895596075	GSTA4	-0.298514029578125	RERE.1	0.393656090856111	NR6A1.4	-0.451953059653558	ZBTB8OS	-0.319083551268296
CASP3.2	-0.440319733867634	IER3.2	-0.698916706938723	DPM2	-0.250577534145592	PDRG1	0.577316858568077	RBCK1	0.296584597712295
NUDCD2	0.276257907012153	TMEM14A	-0.267371012548061	ACTR3.3	-0.353886535703787	MAFG	0.498693564604572	NCAM1	-0.567338917182848
EIF4A3.1	0.265219901381329	DNAJA1	-0.256837335449453	SNRPF.3	-0.337877697936717	KHDRBS1.3	-0.419861291109274	CTBP2	-0.330281961842378
DYNC1I2.1	-0.302187813410677	C7orf50.1	-0.256821853755315	SNRPA1.3	-0.348567143034759	MIR100HG.1	0.330333517169561	LINC00665	-0.337056359550453
MYH9.3	-0.429433611792466	AKT3	-0.260070585831877	CHCHD3.2	-0.264072022365143	CAV1.6	0.438773080790622	VCAN	-0.385046290760442
NAA15	0.256681053907318	TMED9.2	-0.352918039003481	RRAGD.1	-0.264061351907589	DPPA4.3	-0.555588611308699	PUM3	-0.344891419486442
TCEAL7.2	-0.361997269862951	PRDX5.2	-0.306354141621483	RRP7A.1	-0.269854771758884	PPIH.2	-0.370440022040689	CSDE1	-0.35016122058406
HK1	-0.253023023225143	MEIS3	-0.30298167768558	CLIC4.1	-0.265436469532861	EIF3K.3	-0.371451808397903	GPN3	-0.324060181874542
PHF19	0.260770873507204	TBCB	-0.271578457552644	IGFBP7.3	-1.19656009435572	SEPTIN6.1	-0.370065469233038	AGO1	-0.345296923715136
RNASEH2C	0.298500278985548	MT-CYB.2	-0.508822595253322	DCBLD2.3	-0.458694692692719	AHCY.4	-0.490892468481935	GLDC	-0.263356921903922
NDUFA1	-0.259323137192353	MAP4	-0.3213852161998	MLF1.2	-0.26909338702291	CRABP1.6	-1.10671481799199	ACOT9	0.255188687814055
PHB.2	0.275195492464265	HES4.1	-0.327126857638979	TARS	-0.267499052290959	PTTG1.4	-0.871407649036837	EIF3K	-0.328994073559226
MAP4.1	-0.276541322125105	UBE2E3.1	-0.287171210322303	C19orf48.2	-0.292562658746948	ATP5F1E.4	0.334841327470393	AURKAIP1	-0.321656346294728
TLN1.3	-0.348467745158135	ATP1B1	-0.457288436623148	INTU	-0.252418075763149	LARP6.2	0.461565237632174	MRTO4	-0.375152093699156
BX890604.2.1	0.302973177558041	MSI2	-0.355714339036818	NUTF2.1	-0.275504081097346	FST.5	-0.768001887657068	TAF15	-0.397231617124838
RAB7A	-0.257053774972076	KRTCAP2	-0.253060418640454	ECT2.3	-0.2666252855481	HEXIM1	0.426083642616112	TFDP2	-0.361562349917359
TRAPPC1.1	-0.289158835320621	MMP2.2	-0.390507233894472	YWHAZ.2	0.276160197755088	GDI2.2	-0.405569709686266	AP1S2	0.531934674452491
GAMT	0.272389488302341	STAT1.2	-0.377910204988137	CYBA.3	0.452146551897626	MT-CO2.4	-0.571120803057085	BANF1	-0.349360211035895

EIF4A2.1	-0.393992729388819	CEP290	-0.305981084006351	CDC6.3	-0.265606148607172	RPLP0.5	-0.309978975933461	F2R	0.350425435615468
NIN.1	0.289235704959321	SSBP4	-0.279474885292509	PRKDC.3	-0.327850372264652	CWC15	0.453040514822363	PMAIP1	-0.461296586899579
BCCIP	0.260038221687352	EIF3M.1	0.27194269068114	SLC25A39.1	-0.287508244881063	TRAPPC5.1	0.357095332210498	CCDC85B	0.252743901679243
RRP1	0.250526933271646	HADHA.1	-0.303526039477224	PLK1.3	-0.325124049767786	PFN1.4	-0.312534187427326	SEPTIN9	-0.324580337279973
DPYSL3.2	-0.388048573155746	NEFL.2	-1.27892568405082	CSDE1.1	0.330949346332613	LYPLA1.3	-0.431149812335618	BOLA3	-0.336586621339594
DCTN2.1	-0.274187705406274	UBL3	-0.288983386435855	STAT1.3	-0.354290353102123	ANKRD12.3	0.505913546274129	EBPL	-0.319565842562222
RNF7	-0.255174645340202	XRCC6.1	0.263236988235492	GAPDH.1	-0.269577622147344	GIHCG.2	0.507910998861964	CFAP36	0.30003784231531
ATP6AP1.2	-0.289073772027946	PHF6	-0.304765675947507	IL6ST.2	-0.283363180143076	CRMP1.4	-0.585364529916264	VDAC3	-0.333610670527584
YIPF3.2	-0.291426338826211	TMEM219.1	-0.310114135020779	PHACTR2.1	-0.341469814684972	GSTP1.4	-0.558532116560859	TMED3	0.313638340766332
SCOC.1	-0.301125614628857	TAGLN.1	-1.25835635135346	ADD3.1	-0.255442737293724	CENPM.2	-0.359353742121992	MT-ND3	-0.551948408138324
ATP6V0D1.1	-0.273292116492583	MRPS7	0.267934732112318	TLE5	0.403369856302114	NOP10	0.434604295120427	RAD23B	-0.317330786853217
CDC5L	0.260670333940542	P4HB.2	-0.362434810704161	EIF5A.4	-0.296261351357423	CHEK1.2	-0.376453227511219	MGST3	0.266000958715285
KLF6.1	-0.283293442522077	ATP6AP1.1	-0.317237168369516	MAPK10	0.363695085899507	SOD1	0.360484911922034	LRPPRC	-0.363961012601259
SUMO3	0.266154280907809	HMGCS1	-0.457713193311932	OCIAD2.1	0.339001025194741	SFPQ.3	-0.513629697658561	SRSF1	-0.356815183583831
SPTAN1.1	-0.347934576969096	HSPA8.1	0.330172413787188	EIF3D	-0.292153194723975	COX20.1	0.406005872069863	TBPL1	-0.348421134245466
BCLAF1	0.321993112628435	GIHCG	-0.260819130828254	ATRAID	-0.253664880482455	BAD.3	0.445809366811433	MALAT1	0.390032167774408
GYPC.2	-0.411453067515172	FAM177A1.1	-0.316158978156619	FAM162A.1	-0.278102758256477	UPP1.5	0.779138361463051	NOVA1	-0.636708964894158
PEA15.3	-0.35936120789641	SBDS.1	-0.287886838541156	ZMAT3.2	-0.315154150361563	PAICS.4	-0.506999199807388	FLNA	0.430248658554035
SAC3D1	0.342108597390182	CSTB.2	-0.321947385936388	SELENOM.3	-0.404112647389359	RPL26L1	0.451252109348691	TP53I13	0.270205389464346
LRP1.2	-0.329812417144148	NUCB2.1	-0.326822910283334	CDKN1A.4	-0.677466258181927	FBXO5.3	-0.394318968459998	HDGFL3	-0.33492647798326
UPP1.3	-0.476372032956098	ZNF580	-0.300554662840233	KNOP1.3	-0.282786603546602	ARL6IP5.3	0.393903257853614	MTCH2	-0.369151651832833
COMMD6.2	-0.250070745493657	TSPAN14	-0.264850698966002	IRF2BPL.1	-0.260493168787384	FXYD6.2	-0.439293111229757	PNP	-0.308851530856627
CALB1.2	-0.45649023221776	PKM.1	-0.255037936021565	SPDL1	-0.276496754605088	EFNA5.3	-0.373397312086701	UBE2N	-0.321062019530454
CTNNA1	-0.280819722941233	RHOB.2	-0.353244549099959	GRN.2	-0.321385218215429	RABL6.2	-0.446139028977407	CYSTM1	0.360054131845515
BRI3.2	-0.316616241260905	NENF.1	-0.257740524412941	FLVCR1.1	0.288138529036465	GTF2I.3	-0.459432780101486	DNAJC19	-0.310197116431114
KIF5C.3	-0.591629595771514	WWTR1.1	-0.342518222826651	SMARCA5.2	0.286082556056092	BARD1.3	-0.38316928052474	PLIN3	0.314008945586917
DDT.2	-0.250449504151522	FNDC3B.1	-0.32187076455623	FYN.1	0.356370503602329	TXNRD1.1	1.09816007125469	JAG1	0.438011779319602
CCNG1	0.250263093040917	CFAP36.1	-0.289579989250648	SYNCRIP.3	-0.295915108390751	HSPB1.5	0.405565294672656	SH3GLB1	0.296762811951508
ZFAND6	-0.250762324338205	NTPCR.1	0.250808597573887	RALGDS	0.273596168359901	RAN.5	-0.40195905407147	FAM177A1	0.312149976888882
SAFB	0.264564969764736	YBX3.1	0.312872719540252	DNMT1.2	-0.297352035895478	ARL4C.5	0.47076209496953	EFNA5	-0.326437221602764
TCEAL8	-0.260273982985009	CBR1.1	-0.250482194203891	SVIL.3	-0.445010059102332	COX16	0.434740195128261	RFC3	-0.279266823034466
LAMP1.2	-0.289743923143504	FAM229B.1	-0.295763366428953	EPHX1.2	-0.2835378426671	CIAO2B	0.415994993917864	MAGT1	0.272228320964145
YDJC	0.26538063713111	PTOV1	-0.300821784765689	CD47.4	-0.380082771736473	RPL6.1	-0.296407960662461	SLC38A1	-0.340653137917105
LEPROT.2	-0.267013291603269	PYURF	-0.266456883218435	BNIP3L.1	0.333237266480384	TRIM24.2	-0.465194661539424	PGPEP1	0.272704800965853
AP2M1.1	-0.325120109776258	JUND	-0.366347372364668	NUDT1.3	-0.299046653541127	MORN2.3	0.491061141116909	SHC1	0.29860311439215
NAT10	0.253437252685841	MAP3K13	-0.300649140050306	AKR7A2	-0.275985938862663	RPL7L1	0.421729266727151	CRISPLD1	0.308734204152128
TCP1.2	0.275821431816357	EIF2AK2.1	-0.346148428883019	DAD1.3	-0.294900188111069	FAM174C.1	0.499107141781094	MGMT	0.279800177598541
ANAPC15	0.251408273967629	TMX4	-0.278628171435913	SRSF2.3	-0.364235681444067	IER5	0.434539760906814	PHPT1	0.27984309964133
CHMP2A	-0.259878360280443	METTL9	-0.268596634420564	SAR1A.1	-0.257528113798822	CECR2.3	-0.349009387794425	CAPN2	0.270074880556657
CHORDC1	0.254483906071904	IMPDH2.1	0.260339166320631	TMEM165.1	-0.255535380183536	JPX.1	0.512473980119001	MCUB	0.274302818959458
MACF1.3	-0.3600650618872	ZFHX4	-0.298324064866897	NDUFS7	0.311205868565493	MAP1LC3B.3	0.488814097643668	LUC7L	-0.32805496606399
COPS9.2	-0.266943346746833	SSBP2	-0.26874157944937	MEA1	-0.251822836439908	NAV1.2	-0.397083059150104	PHGDH	-0.335582512490649
AHNAK.3	-0.304159201197996	EVL	-0.320507527527689	YPEL3	0.264019354251191	PABPC1.3	-0.435695946522459	NUDT21	-0.325442518887696
PSENEN.1	-0.254916452098773	GRPEL1	0.270421978390219	NPDC1.4	0.373401236007305	DHCR24.1	-0.432208293369393	WDR43	-0.347243902100981
ANAPC5	0.251799551440019	CETN2.1	-0.298470235574649	SMAD5	-0.260546545586727	IGFBPL1.3	-0.653809317720994	STRAP	-0.328512981934685
FEZ2.2	-0.303312512936755	ARHGAP21	-0.31911757062532	NAA20	-0.264506460059084	RPL19.2	-0.255072050037001	NES	-0.523160086185984
FIS1	-0.25168887445231	ABCA1.1	-0.318350866245977	NFE2L2.3	-0.29148835378946	IPO5.2	-0.420696245248317	PNISR	-0.349972025923329
HCFC1R1.1	-0.273057471110061	SLC39A7.1	-0.306952169145554	VPS28.3	0.303486803238353	CHAF1A.3	-0.359672221544732	TIMM17B	-0.298563292175079
ACTR10	-0.258330226411989	MAN1A2	-0.278210914664177	FXYD6.1	0.544757801089129	AC012146.1	0.495107855214582	NPDC1	0.278076698743742
DNAJC21.1	0.254179116244585	FAM200B.1	-0.280114215932881	MALT1.2	-0.307973231998258	CITED4	0.359258687704237	ITGAV	0.328292165311247
SLC16A1.2	0.255533962003082	PDLIM2.2	-0.321460153919118	DRAP1.2	-0.290279285403128	OST4.4	0.305995167381342	PKM	0.3283500996835
TMED10.2	-0.267754080112589	APC	-0.372181220654001	EIF1AY.3	0.289345507408016	BRCA1.2	-0.364679790324278	C12orf75	0.276925624448853
FBXO22	0.251112956978977	BMERB1	-0.262635194851023	C1orf35	-0.300879856503542	MIF.2	-0.344056148185423	ANP32B	-0.447257164765567
GTPBP4.1	0.304614569470162	PAFAH1B1	-0.312899038815457	ITGB1BP1.1	-0.274339829755633	ATF4.1	0.590793113809135	LAMTOR1	0.285542698560238
GPM6B.3	-0.832886505395981	FAM204A	-0.261729029199159	KRT10.2	-0.308764386825092	JARID2.2	-0.415649265648146	SEC61A1	0.325939515891111
DNAJC2.2	0.286616632181493	CAVIN1.2	-0.343287148811453	AGAP1	0.315181079536507	LITAF.2	-0.449190195842109	UBE2L6	0.351148285455031
CDC42.2	-0.258521388639783	PGM2L1	-0.401545899397264	ISOC2.1	-0.276904380953205	FNIP1	0.372306050033831	TCOF1	-0.367211242258884
FKBP1B	-0.268243781483648	MEX3A.1	-0.322777609004635	ATP5F1A.1	0.3182910217835	MDM2.3	0.64938716722622	ATP2B1	0.405338091902485
CLTB	-0.261523137046025	TP53I3.1	-0.312835411694172	ENHO	0.300800849392111	LINC00461.3	0.668765122327075	EPHB2	0.271791464823115
PALLD.3	-0.367670442417853	S100A16.1	-0.325657836452424	RPS10	-0.254349750205846	MIS18A.4	-0.36946429390381	FXR1	-0.298334959731564
CD320	0.306622132231088	MIEN1	-0.278937794102561	KDM1A.2	0.354699225147338	SRSF3.3	-0.410905186715555	OLA1	-0.316363737068697
XPO1	0.250760347274364	TNFRSF1A.1	-0.263553866969597	TCEAL3.4	-0.304309115431921	COX4I1.2	-0.296056259471666	TIMM8A	-0.305141140039761
NMB.1	-0.47657078028216	SELENOM.1	-0.452428236142744	MYEF2	0.363388847841856	TUBB2B.3	-0.64047428545229	FANCI	-0.275635265760351
TMEM179B.1	-0.258701877479123	ADD1	-0.276189531083582	NPM3.3	-0.274745634876171	RPS4X.1	-0.312725053047797	CHD4	-0.385368451710126
CD99.3	-0.600827822456345	LAMC1.1	-0.377829515047499	PRR13.1	-0.280862347288825	RFK	0.497106799351583	CHEK1	-0.319036070486031
VMP1.3	-0.305498079834619	BIRC2.1	-0.253327916053647	CHD7.2	0.35389213265293	LIX1.5	-0.497819071196116	CTPS1	-0.291039918498522
THY1	-0.27125501410386	SRGAP1	-0.291269986855195	HADHB.1	-0.257865952666349	ZMYND8.2	-0.377349237994876	COL18A1	0.297890597487967
IFI16.1	-0.279838640902076	YPEL5.1	-0.274329302237288	C1QBP.3	-0.350529542784114	CDCA7L.4	-0.329650194220751	BTF3L4	-0.302838594048642
PMEPA1.2	-0.339529884348159	NOVA1.2	-0.524637369324417	SEC61A1.1	-0.2644918981469	MFAP2.3	-0.3535276620522	SMC1A	-0.348092521350601
TRIAP1.1	0.253405140940502	IL32.1	-0.977441235984879	NDUFC1	-0.258204673646627	RAI14.1	-0.45874659400034	STRBP	-0.309965174526702
BEX1.2	-0.48598329347016	ARPP19	-0.269600070642029	DKC1.3	-0.289242051376502	POLR2K	0.501281568460282	SMARCA4	-0.384624664098944
NFKBIA.2	-0.43012540015389	CMPK1.1	-0.255923651103782	NFIA.3	-0.373856931904706	IGF2BP3.2	-0.403585059451832	UQCRB	-0.294559874282243
DAD1.2	-0.259683047901552	RTF2	-0.269730082086262	G3BP2.1	0.326397453529751	DHRS7.3	0.474217163226468	DAZAP1	-0.315954332447327
IDI1	0.262042780802469	LRP1.1	-0.351953884409921	TLE1	0.415698750294194	YWHAH.3	-0.419178523518845	MCM6	-0.316922506583385
API5	0.275256592531464	KDELR2.2	-0.255798850105454	SEPHS1.2	-0.301166992188729	NT5DC2.1	-0.520864869299528	LAMP1	0.368756351866716
PRPF40A.1	0.258206229719352	GGH.1	-0.288242063356697	TRIAP1.2	-0.290125434292301	POU5F1.5	-1.17395070344784	SEC62	0.260647613876418
TNIK.2	-0.285479602485566	FHL1.1	-0.300984274989854	DHX36.1	0.322516429426377	HLA-E.1	0.342490149455011	CHCHD5	0.2923807937447
CALD1.3	-0.819147932506891	CLIP1.1	-0.282745575660053	SV2A	0.425900807704797	TIMP1.5	0.282494532952337	PGK1	-0.29956382498259
HERPUD1.2	-0.286320320018924	LINC01578	-0.290463897997444	HSPA9.2	-0.312578935445071	MRPS6.2	-0.437642165310505	MIAT	-0.602718537745196
BAD.2	-0.268080540902765	GINM1.1	-0.298865121118485	TUBB4B.3	-0.429697605732854	ELAVL2.4	-0.500157200446708	RBM25	-0.328074616065177
ARL6IP1.1	0.38772240353232	NSD3	-0.315406019726594	MYO5A	0.366300425538104	RFC3.2	-0.31210194440694	ESF1	-0.319307783180024
BUD23	0.257684211694154	TPGS2	-0.27301146262745	XIST.3	-0.317714720638184	CALR.4	-0.465780857154914	GPX4	0.256509066482535
BMS1	0.263100835956371	FADS1.1	-0.259355572989468	MRC2.2	-0.264469316542189	BUD31.1	0.384230302489154	LEPROT	0.296785673224951
CRIP2.2	-0.310422224076467	SAT1.2	-0.623829129161064	NOP56.3	-0.369844205687304	DUT.3	-0.501213581884419	SDC2	0.405068637126157
PRMT2.2	-0.26906147692822	ZMAT3.1	-0.285796594025471	SNRPD1.3	-0.308214805763424	CHID1	-0.364751095935214	PCBP4	-0.411239579211328
FNDC3B.2	-0.270869411474746	UCHL1	-0.367061310547211	OLFM2	0.345593112395815	SEZ6L2.4	-0.431641226186113	DNMT1	-0.321041473734043
NFE2L1.1	-0.256539053604643	FARP1	-0.312130732267463	XRCC6.3	-0.331841419911656	RNF130.1	-0.333965235197905	BRD7	-0.298942130876695
EIF3A.2	0.340243787756577	HOMER3	-0.286945268488654	ATP5F1B.2	0.260163425174832	FSTL1.3	-0.455982033730339	GTF2I	-0.337013774281588
CAP1.1	-0.261087714127667	SLC43A2.1	-0.306396448630794	KRAS.2	0.298085737437125	CD59.4	0.359031014212102	TMEM258	-0.271380453776136
ITGAV.3	-0.32587099753335	CLIC1.2	-0.257599404739407	SEPTIN6	0.372895347608351	SLC39A10.2	-0.327990207153296	PFKP	0.258539698651321
CSTF3.1	0.256594927339825	MEST.1	-0.350989742453137	TXNIP.1	0.33746374644191	EVL.3	-0.423811161182185	ATAD5	-0.280160707177732
FN1.3	-0.792009321408245	MARCKSL1.1	-0.321306658525677	TGS1.3	-0.266771248280229	GPCPD1.1	-0.312612140923164	FEN1	-0.364266903954488
CCDC28B	-0.269534112854307	SPTBN1.1	-0.415263238578537	SYAP1.1	-0.275991260593779	SEPHS1.3	-0.401899307055735	BIRC2	0.263061439295766
NOLC1.2	0.382641701947674	TCF12	-0.299778066903655	BOLA3.2	-0.270532311745888	MRPS18C.1	0.420984103296459	EIF3M	-0.342848560743453
CAST.3	-0.31923982613637	ZNHIT1.1	-0.262218928889912	ADI1	-0.273835919310654	ISG15.6	0.594534154857941	SLC25A13	-0.314640427619481
DCBLD2.2	-0.503572968923258	BCAP29	-0.264470693722515	TSR1.2	-0.279022254547696	BLMH	-0.317601576121715	MAP4K4	-0.385843967648609
CCDC50.3	-0.304638249165561	SNCA	-0.328512708211669	IMPDH2.3	-0.295450242685183	HAUS1.2	-0.349149817479287	RSU1	0.287707010967166
AK1.2	-0.302021541859499	ANKRD11.1	-0.310324114558318	RRM1.2	-0.359675419291603	DNAJC15.5	-0.403419890742075	CMBL	0.262811041223376
SF1	0.253837931659245	MCFD2.2	-0.286827783747848	MIS18A.3	-0.25390172139392	NUDT5.2	-0.356961610147012	RPLP0	-0.255502287277936
SARS	-0.253524739928083	CDKN1A.2	-0.458754023646997	KLF7.2	0.298944714627886	LAS1L.1	-0.292525723183596	VCL	0.381911671251836
ATP1B1.1	-0.355147325246175	FJX1.1	-0.311993767713263	S100A10.4	-0.629625362431378	NENF.3	0.376038638404495	PTPRA	0.326531244346571
AHCTF1	0.255918113136151	AFF4	-0.294256834339136	INPP5F.3	0.325718987989054	MRPL36	0.422887774774413	RIF1	-0.355825963810296
COA4.1	0.30349195699528	TMEM132A	-0.306017656656108	ECHS1	-0.28262681311438	MLEC.5	-0.373433090385404	TCEA2	0.278047483487047
MAZ	0.256057209258157	CIB1.1	-0.269241237858726	SLC39A7.2	-0.261145966271232	ROBO1.2	-0.377398785495711	CENPX	-0.315898380331573
COL4A2.3	-0.406683667642533	PHF20L1	-0.271564834667581	NUDC.1	-0.291277147510326	LBR.3	-0.420915692306725	IGFBP7	1.60491343737492
MIAT.3	-0.46153965268016	UBE2B	-0.250667400004774	AUTS2	0.366572339940771	CCDC144NL-AS1.2	-0.343519003961735	DKC1	-0.348664388577744
MTCH2.1	0.254737253622442	MGAT4B.1	-0.270681219899995	APLP2.4	-0.385548884631265	SEPTIN9.2	-0.350688069359727	HMGCR	-0.365225218329853
LPP.3	-0.29437134923731	CCDC88A	-0.348287749780557	HDLBP	-0.322273755472415	NQO1.6	1.48377626896415	NFE2L1	0.344639536248715
SNCA.1	-0.284482638007546	ATP6V0D1	-0.27189524314922	RSBN1	0.277635651398958	KLHL23.3	-0.364772858156098	CHCHD10	-0.331051590788502
HSPA8.2	0.379857285873118	APP	-0.354780612230835	HOMER3.1	0.356739952446312	MYO10.1	-0.393478686040424	ITGB1BP1	0.281830428912491
DHCR24	0.255522521131577	RND3.1	-0.356505263321343	MCM6.3	-0.257794985736728	ILF3-DT	0.584789526746691	ASPM	-0.316527378061867
TUBB2B.1	-0.423521529079327	MEG3.2	-0.69337717472651	FEN1.2	-0.304289089084481	HMGB3.4	-0.431974608301992	FLVCR1	-0.283854328934957
TTC3.2	-0.396582599913187	ARID4A	-0.254576347458712	MGST3.3	0.311973882507113	MRPL51	0.377092406131299	NDUFA6	-0.289293390066567
MXRA7.2	-0.305824204119725	IRS2.1	-0.290869076329907	AK2	-0.26894197557688	NSMCE3	0.499001109913963	HILPDA	-0.285064590132997
LAMC1.2	-0.2650327244582	ZC2HC1A	-0.293691554655748	EIF6	-0.268962529713875	MCM6.4	-0.356528688255971	PIN4	-0.304097453052856
ARID5B.3	-0.278675269722438	CAPNS1.1	-0.291683615719484	RIOK3	0.333052218716972	TPGS1	0.460978644799193	BCL7C	0.272075939556235
GADD45A.2	-0.308028023968817	USP47	-0.271773555534074	LYPLA1.2	0.268563302181935	ZFAS1	0.616071574889394	SMARCB1	-0.297654987279874
TAGLN3.3	-0.892977736031519	GNAI3	-0.251808163384539	GNG10	-0.257049894139047	CCND2.4	-0.655041962698841	FGFBP3	-0.443868871109237
RPL22L1.1	0.470157062305569	RGS3.2	-0.332441529433642	SMC5.1	-0.270394012636754	ZNF830	0.426293743280083	CIAPIN1	-0.298104187606088
EPRS	0.335227408330391	SMARCC2	-0.260093473447583	EIF2S1	-0.265480640473106	CBX5.2	-0.409001066170566	AP2M1	0.3300981955792
TUBB3.3	-0.696591889868047	SPATS2	-0.257577293142075	IDH1.1	0.352079388927425	SET.4	-0.386430412378886	MYH9	0.357637112280879
AP1S1.2	-0.265798954481847	UBE2R2	-0.253786328680538	DKK3.3	-0.311622655565697	FASN.2	-0.38660323754494	NUDT5	-0.304894566815459
SF3B2	0.301244638911089	TTYH1.1	-0.58271595615073	A1BG.4	-0.307011166487889	SINHCAF.4	-0.440417630158958	ATP5PB	-0.287939459971742
PRSS23.3	-0.297466116944124	ATP6V0C	-0.287387693768221	BMERB1.1	0.358342090510624	TPM2.5	-1.09922432279935	CCDC144NL-AS1	-0.282916016032852
KCNQ1OT1.2	-0.364262137647547	PCNX4	-0.278957606677274	SSRP1.2	-0.295304479918509	CKS2.4	-0.508936222770162	ASAP1	0.287659166703165
PLTP.3	-0.316627407141399	CEP170.1	-0.367608487543376	SNRNP25.2	-0.294628978895681	RPS26.4	0.306724082169177	MALT1	0.328784676213206
SEZ6L2.1	-0.304586477219094	ZNF608	-0.25624464568546	TP53I3.2	-0.368864490354484	SBDS.2	0.427025561675555	RRBP1	0.32484669653155
RND3.2	-0.281863728696611	PPP2CB	-0.265155302040956	DDR1.2	-0.287696665802246	SSBP3.2	-0.36396891993529	PSENEN	0.291287652235197
PDLIM2.3	-0.250885657936985	TMED10.1	-0.279304408008612	SLC25A6	0.276517669173704	IDH2.2	-0.414760272162524	VASH2	-0.497229268647759
UBC.1	-0.302075080157481	MT-CO1.1	-0.506441224024898	MEIS3.1	0.390844704325347	BCAT1.5	-0.462956693621179	CETN2	0.295137482339105
COL11A1.3	-0.332138803591055	STMN1.2	-0.620182070422071	NCOA1	0.25341325706743	APOE.6	-0.640244292447115	UNG	-0.305552959269822
PLK2.3	-0.336282881850151	PGRMC1	-0.272844553214452	RAB5IF	-0.262976418726475	RFC2.4	-0.348175290088508	CCDC107	0.262608428754034
PON2.2	-0.307500294465519	RFX3	-0.255055567599404	SH3BP4.2	-0.25664382926778	ARHGDIA.1	0.42836355683575	MDH2	-0.335546686336303
SLC3A2.1	-0.331193746959524	ARHGAP5	-0.288279316105257	EI24	-0.289252693367403	RND2.4	-0.418931788321437	COX7B	-0.280195599303112
CRABP2.2	0.253892330145413	SPTAN1	-0.353122711254118	LAMTOR1.1	-0.268853530304994	ATAD5.2	-0.304622337950893	RFC2	-0.322241587920214
NOVA1.3	-0.460280569249462	NFKBIA.1	-0.451054130430029	TENM3.2	-0.280763823412857	RFC4.4	-0.347094485226105	PSMD4	-0.315199477925165
NCAM1.3	-0.450925354749707	SH3BP4.1	-0.254705451645327	RRS1	-0.260156589439528	CNN2.2	-0.504965449451511	CPD	0.297465288887207
TPM1.3	-0.503670376633088	DNAJC1.1	-0.261887795316059	GSK3B	0.347081292015318	ACTG1.3	-0.36045903804755	RBFOX2	-0.382464932659527
NNAT.2	-0.50297940359006	PRKAR2A	-0.271611375992483	RAP1B.1	-0.25458458268578	PDIA4.2	-0.402367846110189	RAP1B	0.26853444167604
CCAR1.1	0.256707553915674	ZEB2.1	-0.270042091571158	CD151.3	-0.408869269344779	DNAJB2	0.305136491997796	ACAA2	-0.3013204466534
VAMP2.1	-0.331662824186311	NPC2.1	-0.402815453717501	ZMIZ1	0.301126798409333	PCBP4.2	-0.417912569936957	ATP6AP1	0.286757726275762
WSB1.1	-0.28955299457044	A1BG.2	-0.318049712359166	GNL3.3	-0.25953179137595	TCEAL1	0.300084488355018	CASP3	-0.475570800321443
MT-CYB.3	-0.382383167839268	RAB13.2	-0.294945908535292	RNASEH2C.1	-0.263729663896802	PKIA.4	-0.406616931480817	HNRNPL	-0.300522420231348
RAB31.3	-0.264676746187832	EIF1B	-0.284928381134481	HIGD2A	0.253750684517023	PODXL.2	-0.356324499842675	RPA2	-0.322882463525325
PMAIP1.2	-0.295457981743232	WASF2.1	-0.250367498475792	CXXC5.3	0.351118441328402	SMARCA4.3	-0.464174015368254	CRABP1	-0.640747157786879
MAGED1.2	-0.251309782489523	ARL4A.1	-0.349527993960729	GART.2	-0.254624731412575	BEX4.3	0.381909362173396	TENM3	0.378274989110813
EFNB2.3	-0.2937855702173	XPR1	-0.312823138627293	CKS2.3	-0.378638532624221	CAVIN1.5	0.363561708228224	TPRKB	-0.28780684710082
C4orf48.2	-0.310529010528398	PAFAH1B3.1	-0.338098758192751	PRTG.4	-0.371378215602236	GABARAPL2.3	0.313980459661074	EXOSC5	-0.273676469903028
ELAVL3.3	-0.394691901617614	LIMD2	-0.250542555003437	HSPA4L	-0.272539625098525	NFE2L2.4	0.454577514143359	JARID2	-0.365843671104567
RUFY3.1	-0.279045136336366	TENM3.1	-0.302553501033987	PHF14.2	0.349990977707059	XRN2.2	-0.335591465545765	RAD21	-0.287860322651011
RBFOX2.1	-0.258760614764434	TLE4	-0.300681771765354	MRPS17.1	-0.26231469377241	LARS.1	-0.356293984309758	PRDX2	-0.377651357720802
CALM2.1	-0.338592223003081	TXNIP	-0.322352300647596	DNAJC15.3	0.262007612925497	SGO2.1	-0.403272995421767	FKBP10	0.320052162081698
MT-CO2.2	-0.261787515297269	CCPG1.1	-0.314469242014795	GNAI2.2	-0.277770729515831	POLR2L	0.336129015849224	SLC16A1	-0.341789593490552
CALU.3	-0.253645347481414	ACTN1.2	-0.490964223800634	COA4.2	-0.308121802973069	MTDH.2	0.351251940446173	SPC25	-0.253236376242023
RGS5.3	-0.33623291612317	MARCH6	-0.26682929375207	FAAP20.1	-0.250580969502695	PPP2CB.1	0.397427076909842	EIF4E	-0.282553627276052
APLP1.1	-0.297360090197945	MALT1.1	-0.25845499804899	SMARCA4.1	0.383365769466994	CHCHD6	-0.319779125029522	CLIC1	0.251701580827221
INA.3	-0.585337435793982	MSRB2	-0.251552434276582	DST.3	0.292897197105598	RBMX.2	-0.3944057577212	PPIL1	-0.298232282812782
NT5DC2	0.288833684453534	APLP2.2	-0.390666298072023	FHL1.2	-0.321157741600136	UBE2S.5	-0.575886159805256	CYFIP1	0.260059363816564
PPP1CB.3	-0.301049916415092	DSTN.1	-0.278059262189017	TRAPPC1.2	0.268832851430379	HMGCR.3	-0.370759519527531	BCAT1	-0.378185317730699
TSC22D1.3	-0.299711845099361	KMT2A	-0.289156863646042	SHOC2	0.302640972141117	HNRNPAB.4	-0.442140182293639	CENPE	-0.283789917432874
CPE.3	-0.318145751989589	DOK5	-0.251562431385978	PMAIP1.3	-0.253651735587917	TUBB2A.4	-0.463388634459769	AURKA	-0.303819591243363
NEFM.3	-1.80847855194106	ZFR	-0.253911446087119	MXI1	0.289342038536504	UBXN1	0.346973657938504	NTHL1	-0.31325370278881
MT-ND5.3	-0.260955796927251	ROCK2.1	-0.258644285595115	KLHL4	0.259948679903522	MLF1.4	0.423955273686967	TMEM9	0.257374766654028
PSME2.3	-0.350425341540386	LINC00632	-0.273659278048651	ETNK1	0.325284304501565	FARP1.1	-0.356754345869317	CDH6	0.606533579359525
MYL9.2	-0.261534575294347	NQO1.2	-0.629258055701425	PLAAT3.2	-0.305958322786968	DRAXIN.3	-0.359901195954406	PAK1IP1	-0.303046441827135
ACTA2.3	-0.953972091647986	CCN2.2	-0.929197925281674	SVBP.1	0.358757649591532	BOLA2.2	-0.274195572555234	MCM4	-0.349333301945355
TSTD1.3	-0.25777314963192	PKIA.1	-0.322783628799639	SNN	0.288325977938147	SNHG25.1	0.800501978237736	EXOSC9	-0.29799235249858
LDHA.1	0.327579245046326	TMOD3.1	-0.280711703992719	TNRC6B	0.351212782568764	ANAPC5.1	-0.310262831897738	PCLAF	-0.317603383930156
MT1X.3	-0.428902156045428	SPRY1.1	-0.391149872596377	BTF3L4.1	0.29691448251153	IFI27L1	-0.289698935835602	EDIL3	-0.372178273052015
CRMP1.2	-0.250087366607378	SVBP	-0.254526878029721	GALNT2.1	-0.256904120122204	SERBP1.4	-0.394350973828458	H2AFX	-0.362619433181723
PLCG2.2	-0.258017083850197	BCAT1.2	-0.31406387928441	NUDT3	0.416045079262047	ADD3.2	-0.317250788252854	TMEM106C	0.29412055080267
ELAVL2.2	-0.299592643183665	RAB6A	-0.269502842906606	PABPC4.3	-0.272536212672719	CXADR.3	-0.376522405701314	MXRA7	0.319452093057616
SOD2.3	-1.03250773164673	NAV1	-0.261441399559149	HDGFL3.1	0.344957369568113	ACLY	-0.337609697585672	SYAP1	0.300724239455299
MAP1B.2	-0.405418897168735	DDX17	-0.277375291348145	MRPL12.3	-0.292097931719195	CKAP5.1	-0.415790557364976	COPS3	-0.292104861034053
PTN.3	-0.567167385021919	ROBO1	-0.269623623910461	PALM	0.275224801193376	PGP.2	-0.340077440565223	CDKN2AIPNL	-0.288062382599314
DPPA4.2	-0.261751040637689	GNB1	-0.25166125764964	HSPH1.2	-0.303315630518539	PITPNA-AS1	0.489410495086302	BOLA2	-0.28123833049002
RGS10.1	-0.272070807029973	HMGA2.1	-0.348938794392665	DPYSL3.3	0.492117253460146	RPL4.3	-0.401461037704018	EIF5	-0.308755692029388
CNTNAP2.2	-0.272398045231295	HAGH	-0.25623620054498	SOD2.4	-1.17200438086384	EXOSC8.4	-0.396632521181432	TMEM165	0.299652733507237

SCG3.2	-0.346455896083473
NREP.2	-0.258174519905716
PTX3.3	-0.404077281246965
SDF2L1	0.254650742131848
TUBA1A.1	-0.458052973169331

BAZ2B	-0.317896520416998
LIMA1.2	-0.268075534663521
CSNK1E	-0.264846623988055
FN1.2	-0.865745895000487
RSF1	-0.252812711079702
FSTL1.1	-0.343098051303413
KIF1A	-0.275521302592571
KIF3A	-0.250776843272638
AP1S2.1	-0.360789784385067
TAOK1	-0.26379402254974
FGFBP3.2	-0.354267819893006
MYCBP2	-0.307267894794122
SFRP2.1	-0.270554151211555
CHD2	-0.267283994633961
GADD45A.1	-0.312941918621495
EIF4G3	-0.250437014182839
RPL22L1	0.332862798681578
ITSN1.1	-0.284447430462309
ATXN7L3B.1	-0.254561475449314
ZNF292	-0.279487366644927
STMN2.2	-2.04730655254228
TNFRSF12A.2	-0.492796600764924
VAT1	-0.251392247836982
CLU.2	-0.547033885287481
NKTR	-0.268888287234729
SOD2.2	-1.14207314774697
LAMP1.1	-0.251204238246307
CNTNAP2.1	-0.567653880169429
CDK6.1	-0.253990579098354
PSME2.2	-0.417909656210558
IGFBP7.1	-1.02803649551465
IGF1R.1	-0.284302800771802
MT-ND5.2	-0.476281641593538
PMEPA1.1	-0.303968368332572
S100A10.2	-0.516811792301728
FLNA.2	-0.328021538653284
DPYSL3.1	-0.338950462131795
KCNQ1OT1.1	-0.466529059720752
MYL12A.2	-0.255236204596819
HSPB1.2	-0.294003488475884
MTRNR2L12.2	-0.445127567597832
NCAM1.2	-0.409374950247047
ELAVL3.2	-0.420458871542605
NKAIN4.2	-0.340607069122641
PLPPR3.1	-0.296868129606083
SUCO.1	-0.282925641024767
CCND1.1	-0.481315414206103
IGDCC3.2	-0.437041621650695
SESN3.1	-0.302636991292125
FTL.1	-0.551194902803745
INA.2	-0.646793490872005
IGFBPL1.1	-0.327788625625881
PLTP.2	-0.294921297930974
PHLDA2.2	-0.343947806227114
MYC.2	0.669846260257433
RGS5.2	-0.348314336429563
ELAVL2.1	-0.350299107652256
CRABP1.2	-0.692847320123773
MIAT.2	-0.404581404873408
MT2A.2	-0.754422861825135
MT-ND3.2	-0.292335248973975
MT-ND4L.2	-0.285212477292659
TPM1.2	-0.468504333207118
IFITM1.2	-0.924535739388807
MT-ND6.2	-0.287100253784323

MANF	-0.296467436787516
SSBP2.1	0.319747554869808
DHPS	0.310624156480604
ORC6.4	-0.260012744553895
TCOF1.4	-0.274441854907389
ARHGEF9	0.264176942055622
SNHG3.1	-0.267073143075968
DBF4.3	-0.260172630782343
FTX	0.315941258844791
IRS2.2	-0.251022946554767
POLR2I.1	0.255220652384918
DUT.1	-0.298197187875393
GET1	0.328026415559172
MMD	0.312028888574473
HNRNPA3.4	-0.286065040538241
HIST1H1C.1	-0.286159203193977
DDAH2.1	0.324588909760868
RAN.3	-0.266107355066857
SARAF.3	0.327098481307546
FLOT1	0.292313012213619
DUSP4	0.289776093746039
PGAP1	0.283843336178018
GNB1.1	0.32321005158837
GNAQ	0.388750730831543
PPIP5K2	0.296680568407637
CCDC144NL-AS1.1	0.275368357127682
INAFM1.2	0.358178983036746
CAMLG	0.338052630366055
CBX3.2	0.266354765335232
MTX2.1	0.296001654140297
SLC39A10.1	0.301927000437385
CNBP.3	-0.257783158562835
CNTNAP2.3	-0.529239962557763
LNPK	0.26826863343143
ARL8A	0.289768823190781
SCOC.2	0.316538203337972
DCTPP1.1	-0.262684853968208
SPAST	0.266151034139645
HNRNPM.4	-0.312979476550575
FKBP1B.1	0.315106675084653
AGTPBP1	0.262252832903946
SAC3D1.1	-0.287090951620749
ATAT1	0.253730064411547
ARPC2.4	-0.256757020601523
DOK5.1	0.367711423613017
TUBA1B.3	-0.320947868326843
AGO1.1	0.261196314958665
KLHL23.2	0.292369405067948
TPM3.1	-0.260682106947122
CCDC88A.1	0.374135069041832
RNF19A.4	-0.408483450946913
IPO7.2	-0.25859994948927
KAT6B	0.279582763469246
PHLDA3.1	-0.273315089343618
FTL.3	-0.622820486064124
BNIP3.2	0.268273413923625
TOP2B	0.355732809991112
ZNF292.1	0.343360277887327
APP.2	0.359966149065686
MTMR9	0.250812001755968
MEAF6	0.354436369772112
BAG1	0.301211234615241
RGMB	0.301838949576104
STRBP.1	0.291448070903407
ANKRD12.2	0.34806854455238
RAB6A.1	0.346570163811742
UBL3.1	0.320369906222965
CCPG1.3	-0.269274074883332
KIF1B.1	0.341773148907599
ARID1B	0.316116759521925
LAMP1.3	-0.251989042105273
SOGA3	0.263374685030058
TSG101	0.303960408111624
CHD9.1	0.281354909640974
PIAS1	0.259666200785634
ATP6V1A	0.279561797888567
WSB1.2	0.333654197283
UBE2D1	0.264567117933491
HNRNPD.3	-0.280452235330888
NEAT1.3	-0.966443969067192
WTAP.1	-0.261672027602161
CEBPZ.2	-0.279591914750213
NSF	0.269300833275647
SPAG9.1	0.29103789966515
HMGA1.4	-0.454113971712245
CCDC85B.2	-0.290234239952334
ATF7IP	0.270072408359162
PCLO	0.269625658971414
OSBPL8.2	0.313035339762558
NGRN.1	0.313342946951677
SMARCAD1	0.271064021334107
CDH2.3	-0.34830427999684
NT5C3B	0.300313350159007
TTC3.3	0.592489841334188
XPR1.1	0.345439467928084
USP22	0.321641876930808
MTF2.1	0.272057945408353
DRAXIN.2	0.286579482254197
BAG6	0.291700171756424
ITGB1.4	-0.388373649160973
MT1X.4	-0.315357441141618
MEG3.3	0.304053302342469
STX2	0.254807688183277
ATAD1	0.254482899190236
CSTB.4	-0.281789449891457
TERF1.3	-0.337372626973281
CLTB.1	0.278783199829163
VGF.2	0.270363952683382
TXNRD1	-0.300489402338534
AFDN	0.267031679590858
ISCA1	0.253440216068248
KRT8.4	-0.977644417905699
ACOT7.2	0.289211551930564
AKAP9.1	0.35108688218543
AP2B1	0.290039140439316
HMGCR.2	0.356978443570577
SRSF7.3	-0.259324724204683
NIPSNAP1.1	0.261934684624709
CASK	0.254403027609189
BSCL2.1	0.383061898938707
SLC38A1.1	0.290371694924257
ARID1A.1	0.26411788976358
AKAP12.4	-0.642871349641951
OGA	0.285517603771508
PPP2R2B	0.332239211400651
KIFAP3.3	0.328140469495275
HES4.3	-0.341164913178024
LDHA.2	-0.400796753207225
SNHG19	-0.260821867944199
MEX3A.2	0.310626045906192
PLEKHA1	0.286590554904165
H1FX.2	0.264633829658402
SPTAN1.2	0.398582029662581
COTL1.2	-0.339224771984433
PRRC2B	0.276390573747153
NES.3	-0.437281933948769
SOCS4	0.273339549741187
OPTN.3	-0.251651825230644
TMX4.1	0.291981192076442
TFDP2.2	0.266941173710665
ATP2B1.3	0.36997615225811
EZR.3	-0.254601323632871
CAMSAP2	0.251216468475861
BOD1L1	0.264290593393843
SFRP1.4	-0.272647784511603
RMST	0.256266842304676
MSMO1.1	0.250958658036246
BAALC.4	-0.317062545065118
SAT1.4	0.485540721422712
ENAH.3	-0.337583007154174
CD63.3	-0.268630942864114
CCND2.3	-0.286637405904698
HIST1H4C.3	-0.692393007119826
SPOCK1	0.328279447072334
ID4.4	0.417559338372524
CRABP1.4	1.30384326535035
FABP5.1	-0.301767144605505
MTRNR2L12.3	-0.409070170465257
TCEAL7.3	0.254755459854485
CNIH2.1	0.291130121304363
SPTBN1.3	0.289146897117421
APOE.4	-0.370152039107082
SNCA.2	0.416782012361973
BEX1.3	0.251310489203624

SLFN5.1	0.348795119216721
UBXN4.2	0.37485574387839
UBE2I.3	-0.336689687356236
NTPCR.2	-0.334471624425786
NSMCE4A.1	-0.291497905593539
DTD1.1	-0.324194268286003
GAMT.2	-0.344339191312281
ITGB1.5	-0.444022367501082
CDKN2AIP	0.404634555535303
TERF1.4	-0.525434099865376
ATP5PO.4	-0.262199348018723
EMC7.1	0.441692441901704
TECR	-0.324488292629595
R3HDM1	-0.282425979402898
BEX2.4	0.484578992059225
AASS	-0.286104396563992
PLK2.6	0.666034678214234
SF3B6	0.38833103103404
NFIA.5	0.459429911442
AKR1B1.3	-0.358823163968504
RIT1.2	0.412108024651087
TRIR	0.352207059301804
SRGAP3.3	-0.411571452985822
MCRIP1.1	0.395743978294328
IMMT	-0.310405401560249
JAGN1	0.446264878600048
RPL39L.2	-0.328119081012152
SCOC.3	0.388088323416217
PDXK.1	-0.303658862828864
NOL7.1	0.375824575495704
PPDPF.3	-0.27625553531031
TET1.3	-0.318309915247529
RCN2.1	-0.311239567143003
SH3BP4.3	0.436139691895448
MCFD2.4	0.406849574358603
NUP107.1	-0.28131452039613
LRPPRC.2	-0.31813569226289
AC010642.2.1	0.420823170558554
FAM177A1.3	0.366324977837459
EPRS.1	-0.390276663955717
VMP1.6	0.45535299148542
TAGLN3.6	-0.934456381907233
ANXA5.4	0.392341360646926
NXN.1	-0.287902320381252
SLC7A11.1	0.560567272839532
TMBIM6.3	0.300315579453597
PLAGL1.4	-0.385207573949803
DTYMK.2	-0.394822912615003
HES6.5	-0.604811011149288
GJA1.4	-0.425338446290997
PDLIM7.2	-0.465286557626779
CCNI.3	-0.379992360491533
LAMTOR1.2	0.326553201834
GLUD1	-0.274763775864745
SNHG3.2	0.788139038995162
RHOC.5	0.326841596385907
PFDN1.1	0.408020732876139
SELENOM.5	0.439269330296181
C4orf48.4	0.283759072141091
PHLDA1.3	0.490549673346708
MRPS24	0.383030097617256
LAMTOR4	0.318773552983369
CNIH2.2	-0.337974846850742
CDV3.2	0.33875790884301
POLD4.4	0.424346889798238
PSENEN.2	0.405465701776497
CHMP1B	0.525635377784336
KRT8.5	-1.04413262041406
PRTG.5	-0.494979718211297
UBC.2	0.554536648128089
RBFA	-0.270593002531348
GPM6B.5	0.412689387101301
CTNNBIP1	-0.308607600367146
GTF2A2	0.40780336108076
FKBP4.2	-0.371885713039205
HMGN1.3	-0.275097412699802
SIPA1L2.2	-0.346765992156885
ZFPM1	0.341813372702955
THOC3.4	-0.257776193688726
SNRNP70.3	-0.388968160971484
PGK1.2	-0.307080780294415
LRP1.3	-0.352939856085658
MICOS13.4	0.346461827096278
TPM4.3	-0.386141537947154
NDUFV1	-0.346703717823299
DPYSL5.3	-0.330141959960647
TXN.1	0.717526448854907
MIEN1.1	0.404541887449137
KIF5B.2	0.320379237607527
ELOC	0.337301961668516
CALM2.4	0.335464545723002
PFDN2.2	0.357421568759311
ATP5F1C.3	-0.272592413931683
C1orf122.3	0.280880810189219
SLC25A13.3	-0.280948627104467
NSD2.1	-0.302486560325069
SNHG14.4	-0.522757683048814
LGMN.2	-0.267491786998429
PHLDA3.2	0.541825616758793
SRSF2.4	-0.356548048840432
AGO1.2	-0.307965199977412
SLC25A6.1	-0.367485074167949
ECI1.2	-0.288256747811073
TUBB3.6	-0.838789189940478
CKAP2.2	-0.381588619969217
BCL2L12.5	-0.288950390067869
MGME1.2	-0.252959279711805
EMC6	0.448919585027294
AFF4.2	0.43926000077529
CLIC4.2	-0.283105696428222
CSRP2.5	-0.353341529050754
TIMM13.3	0.321592376472216
MRPL23	-0.296330237703141
AFDN.1	-0.302516409512605
MRPL54	0.363674701397785
EPB41L4A-AS1	0.449450045584723
ZNF428.2	0.261660644197783
SOX4.4	-0.405291235529379
POU3F3.1	0.286639788351266
PODXL2.1	-0.312281075655689
TMEM132A.2	-0.303103940348776
CPSF6.2	-0.263006970661127
TPR.2	-0.352932526711149
CNPY3	-0.261286886829092
LUC7L.1	-0.272818984623388
MZT1.2	-0.286146388904663
SKA2.1	-0.327441552927707
SPTY2D1	0.355213127211432
GABPB1-AS1.1	0.498457695227624
NAT14	0.394492993953915
PDIA3.2	-0.323556067241397
GNG11.5	0.349802228610349
TGFBR1.1	-0.271068184581371
ATG101	0.400121536086611
MAP9.2	0.39280901748767
DNAJC9.3	-0.310224327612282
ZNF226	0.291707463595155
BLOC1S2.1	0.352598807465528
CRABP2.4	-0.418157043762355
CCDC34.3	-0.386659151164234
THY1.1	-0.311172962033291
ACADM	-0.266736720283774
PBRM1.2	-0.3106971479282
PHAX	0.395098275183691
POLR3K.3	-0.299473691190917
LINC00662.1	0.408664454742432
CD24.5	-0.701287504635877
CADM1.2	-0.399589467141129
NRDC	-0.266548311288793
C9orf16.3	-0.360308783837003
UBE2E3.3	-0.325490757443299
FADS2	-0.315861798743887
COTL1.3	-0.463363064026519
MTHFD1L.3	-0.255919574669306
TPM1.5	-0.746576734238988
RAB5IF.1	0.386140673543714
HACD3.2	-0.292969164050255
NORAD.1	0.423540951527575
ACTA2.6	-1.07470784631206
LAPTM4B.3	-0.349432653937064
SLC2A1.2	-0.355706786851965
HUWE1.1	-0.301343351430055
RNF181.2	0.326553602923579
HMGN2.4	-0.439366316010762
HIKESHI	-0.277068881970741
ANXA1.6	0.321405042120777
SMC6	-0.271278409655601
AP2B1.1	-0.294335424151272
NAA38	0.33411939204491
EVA1B.6	0.339461029957441
COPRS.1	0.347058418042479
JPT2.2	-0.264331733380069
ARF3	-0.253474570512249
SCD	-0.299836171390385
BORCS7.1	0.364080283712867
ACVR2B.2	-0.253227297764804
CCDC14.1	-0.258882408331826
ADSL.4	-0.287352665860157

PAFAH1B3	-0.422101447590775
SSRP1	-0.417714000363049
AHCY	-0.373422728184557
BRCA1	-0.262270291195464
FASN	-0.344513073983052
PDCD6	0.253031919243019
POU2F1	-0.268436666649512
NAA50	-0.308531984513218
TRAM1	0.276784892964753
TPR	-0.323148076300716
TSPAN3	0.279145794312307
CHCHD3	-0.289733401379486
UBE2V2	-0.269982703418114
NIPSNAP1	-0.282160314555222
SPTBN1	0.280627800849089
CBX1	-0.2924567495429
MDN1	-0.268118404095175
RRM1	-0.404846273187269
EWSR1	-0.314852838688618
VMP1	0.250087376406498
SLC39A7	0.29724673496138
PSMB3	-0.263073341593317
HES1	0.28054151473899
MTRNR2L8	-0.482364925951352
CDKN1C	-0.50725339631139
ELAVL1	-0.277303540655739
SMC5	0.308685490743213
RRAS2	-0.277799900847576
NOLC1	-0.380016976560073
SEC11C	-0.349403044401641
NIFK	-0.306222339061166
SLC25A4	-0.293123593029895
RAD51C	-0.307310270050326
ECT2	-0.255226886306625
RUVBL1	-0.334672660878304
SPAG16	0.25360357045496
EPB41L2	-0.285027628883036
RNH1	0.259765032947079
COMT	0.30089812532309
PABPC4	-0.337420683053398
NSD1	-0.285748950065971
ACOT13	-0.274348002216267
CTSD	0.351525061457435
ECI2	0.331296252257215
MT-ATP8	-0.419384877717999
TMA16	-0.289660869476019
PIK3R1	0.28577065872345
SNRNP25	-0.300959064004094
C3orf14	-0.322499812846529
MSH6	-0.335608168553202
TIMM10	-0.261397269111104
FAM133B	-0.279420553652016
DGUOK	-0.267504818533388
ITSN1	0.360104346900962
PTK7	0.293565706470285
TRIAP1	-0.295554280956254
C19orf48	-0.295738185233943
CYB5R3	0.287694766083514
SRRM1	-0.287646091858637
TGOLN2	0.256326114493799
MPHOSPH10	-0.273040579609583
EZR	-0.359093047135074
ALDH7A1	-0.280696045653831
TUBB4B	-0.382173690704677
ZNHIT1	0.255065035232002
TARDBP	-0.294578581079078
LPP	0.262811075567615
TMCO1	0.255230553349533
POLR2F	-0.282286416395714
RPF2	-0.293284305064864
PRRC2C	-0.331491050370517
ZC3H13	-0.279433440462203
KIF22	-0.257571179844401
PGP	-0.307431225520612
IMP3	-0.292874382043984
MDH1	-0.25888403888242
HNRNPR	-0.303629488476068
POLR2D	-0.261202642903252
RRP1B	-0.322944487458822
GLIPR2	0.256938679136861
FDPS	-0.346429426450773
CD3EAP	-0.255521891414144
BEX1	0.433507739972528
INAFM1	0.258430133810495
CCDC34	-0.302089996229449
THOC7	-0.260711696283565
RERE	-0.287981513564839
TRMT10C	-0.278204640062416
AAMDC	0.256828659130709
SYNCRIP	-0.309703865439167
TCTEX1D2	0.252902396485473
MLEC	-0.305907528367487
APEX1	-0.259725743109321
SLC39A10	-0.252800620297718
CDKN1A	0.490251165632385
TMEM160	-0.250248451711939
TMEM208	0.259465757348263
HSPA4	-0.283503476649907
G6PD	0.257080026879809
ARGLU1	-0.285282615633179
LSM2	-0.254825908974658
EIF5B	-0.275102335736172
EBNA1BP2	-0.303327268103781
ARID1A	-0.274172700799633
YTHDF2	-0.252984415965465
MRPL21	-0.292330030167407
HSPH1	-0.357308774803055
BEX2	-0.384301149079402
ATXN10	-0.260921176669685
SLC3A2	0.349552229485089
USP1	-0.264665050190079
FBXO5	-0.262488442187603
NINJ1	0.26936004605643
BOD1	-0.265059492275868
XBP1	0.295034658827837
CISD1	-0.257147728129132
HNRNPF	-0.296817230276417
HCFC1R1	0.284097887915221
WDR18	-0.259317233062328
DNAJC9	-0.256785214845763
CDK2AP2	0.262456417549491
PPIG	-0.259839797778668
NDUFA5	-0.288951973418922
NKAIN4	-0.269469294811759
PBRM1	-0.287637600384031
HSPA9	-0.295917264302842
WASF1	-0.273189642474236
SERPINH1	0.298822014768505
MED28	-0.253463298875946
TGS1	-0.275424498371207
LRRN1	-0.296995248984131
USP14	-0.250672458201484
ZNRD1	-0.266214585253654
MAP2K2	0.260078171954239
NCBP2AS2	-0.268070845959736
PSMG1	-0.294351961161512
AKIRIN1	-0.267059889196294
PLK2	0.27973773974269
SPRY1	0.353656024941645
PLD3	0.336527190818817
MSH2	-0.275305260498958
TIMM13	-0.274169773294916
BX890604.2	-0.284630080914289
CEP78	-0.253837236479968
CPSF6	-0.261881676579886
DICER1	-0.299630562514417
ATXN7L3B	-0.300223465876624
MTX2	-0.256084358287022
EIF4A3	-0.267364202986713
CLTC	0.271720883837437
PSMG3	-0.251862692800339
ACTL6A	-0.253894073811407
ZC3H15	-0.256058288233137
RPS3	-0.256512844552293
PRPF40A	-0.266050041186365
ISYNA1	-0.255333269706073
MCFD2	0.256560683524133
PRKAR2B	-0.280417495916985
NFYB	-0.264718107674055
SURF2	-0.251997141742222
YIPF3	0.259777581114632
UBE2E3	-0.279188829361462
GART	-0.273987869132803
MRPL1	-0.254025645337237
MPP6	-0.251780500078459
HEG1	0.316662487996319
CSNK2A1	-0.254644817075903
HM13	0.259591557795547
KDM5B	-0.297328498672206
PALM2-AKAP2	-0.278801860918267
CSRP2	-0.295533899269188
TRAP1	-0.302651759091518
SNHG7	-0.261588589919968
TAF9	-0.254224893432724
MTRNR2L12	-1.0741137878353
CXXC5	0.284474977051603
G3BP1	-0.254365354785206
MT-ATP6	-0.604722008224145

PXMP2.1	-0.282239286966892	PBX3	-0.259565409101943
GPI	-0.281932461625211	DDX1	-0.256042286623506
SRP19	0.36112574205902	GLRX5	-0.263181351046369
STRBP.2	-0.265773616168161	NDUFB8	-0.25247055258048
SMARCA5.3	-0.308295681285502	TXNL4A	-0.251925242954657
MSH2.2	-0.285796403474925	TWISTNB	-0.258577898970207
KIF5C.6	-0.65919768862383	IMPDH2	-0.288645672098821
ISOC2.2	-0.277941694672174	YIF1A	0.251004120019691
MMAB.1	-0.260557720027312	YWHAG	-0.310113533293589
CD44.4	0.354273047484603	ALDH9A1	0.262372143736653
PBX3.2	-0.264649560884914	SESN3	0.378520512671016
PPAT.3	-0.278255631699142	DHX9	-0.29265110713281
MPP6.2	-0.257675340668102	KIF21A	-0.326955265850728
BEX3.1	0.27376544459075	ABCE1	-0.261578463569618
TRAP1.2	-0.318940743003106	PRKCI	-0.252934446565855
NPC2.4	0.28818475990928	RPL35	-0.252391163395174
SNRPB.5	-0.380893438855102	MT-ND4L	-0.480289967521953
FN1.5	-0.831572289957772	RHOB	-0.339313547512197
SH3BGRL.1	-0.265862030143423	DNAJC2	-0.261970443993504
KDM1A.3	-0.328621579092244	FNBP1L	-0.279890777310764
DCP2.2	-0.265782868785049	CALM3	-0.27062357178908
DAZAP1.2	-0.258341181340464	CCAR1	-0.254505600043248
BTG1	0.557642544066145	SNHG3	-0.28798394985761
HADH.2	-0.250876967527999	KIF5C	-0.625684919928838
MAGEF1	0.436172782011141	ACTA2	1.09934085723691
ID3.3	-0.535732175054249	THUMPD3-AS1	-0.267231125999151
NAE1.3	-0.311506113836695	KPNB1	-0.266555316373308
NIPSNAP1.2	-0.27720287564591	TMSB4X	0.368319018103794
COX19	0.380186027747038	TUBB2B	-0.421907781728523
H2AFY2.2	-0.286238651878641	EIF3A	-0.288232930279052
CPE.5	-0.70588048668551	DUSP6	-0.25512747379289
GANAB	-0.269535605057373	PCM1	-0.259156942886912
KCNMA1.1	0.394924598052998	MRPS26	-0.254049696147034
DDX24.1	0.304338749231115	BAZ1B	-0.258191063399907
TMED9.4	0.29120465422291	CPE	-0.69925948452864
MEST.3	-0.322334191879962	CSTF3	-0.254007567971756
AHNAK2.1	0.38919639665272	MACF1	0.302566641832159
KRR1	0.416493225132498	CADM1	0.271150532935478
HNRNPH1.3	-0.315656951924622	SLC2A3	-0.304280903700983
CYFIP1.2	-0.268774331430823	HSPA8	-0.337835831816109
SUPT16H.2	-0.366597197833095	TOP1	-0.250862728849392
ARL8B	0.347900240468038	FN1	0.593508752980588
UQCRH.3	-0.2774856221222	CCND1	0.654012848767304
TCOF1.5	-0.297186639292398	RGS5	0.521328585923329
EEF2	-0.415184465503209	RABL6	-0.289223990535366
TAF15.3	-0.374522186711165	GPC2	-0.266574733844408
SSNA1	0.333560854810533	CEP170	-0.313642147098799
PDP1.3	0.40541717698402	GSTO1	0.284538281884626
MED31.1	0.389453144261652	NEXN	-0.280700520678689
CFL1.3	-0.336314579440805	SUCO	0.406990721275944
IARS.1	-0.259338112682337	DAAM1	-0.270288607695185
TRMT10C.1	0.469558514557233	MEX3A	-0.262800753044858
PTPRF.1	-0.264129868657219	TSC22D1	0.474523309939992
ZNF791	0.327605658719932	HDGF	-0.298297299272407
EID1.1	0.26103879256023	CNTNAP2	0.750847514755685
MIDN.2	0.438232879509544	CCN2	-0.288265233629207
TIMM8B	0.271326197467852	FADS1	-0.330867320678569
STMN3.1	-0.257558843267848	IPO7	-0.268382544747006
TMEM147	0.295719146573916	SPRY2	0.254287694124351
C1orf21	-0.26271763951021	CCDC50	-0.281802203990398
ETFRF1.1	0.3409990822159	TUBA1C	-0.293026563694944
PHKB	-0.259074135403411	BID	0.261403527510602
SRSF7.4	-0.361090052329537	TCAF1	-0.258406108547703
RPP21	0.355619872279003	ID4	-0.252053058960064
PGAM1.2	-0.279249984785717	PRDX6	-0.266718293055676
LRRC58	0.286315136465476	PLPPR3	-0.264902090202189
DCTN1.1	-0.294939817654745	AKAP12	0.31138046881622
LRRN1.2	-0.265603754018086	KLC1	-0.339324244007425
SNRNP25.3	-0.31592735467616	TPM2	-0.59972197256988
CRELD2.1	-0.268512145624214	UGP2	-0.309564515880783
NUFIP2.1	0.433997948940695	SYT1	-0.6385002697218
NR2F1.4	0.350645831330453	MT-CO2	-0.420087039276154
GGCT.2	-0.328379050329987	MLLT11	-0.557354610834107
PURA	0.312761228092232	MT1X	-0.483106105792467
CTNNAL1.2	-0.275618052851848	ENAH	0.359141470324582
ANK2.4	-0.405210286623828	MT-ND6	-0.402283218622384
EXOSC9.3	-0.258088124202208	PLCG2	-0.504119127235699
EIF3E	-0.253800027810338	WTAP	0.294096505225264
TUBA1A.4	-0.468217029223704	MYC	-1.38895912993354
FEN1.3	-0.300352083373119	MT-ND5	-0.513071242330899
PABPC4.5	-0.270168406433268	MAP1B	-0.386348861900384
AL627171.2	0.31588802379964	BASP1	-0.309652227466832
LYPD1.5	0.387077429099341	MT-CYB	-0.296862423625095
GART.3	-0.257157498617271		
APLP1.3	-0.397053426347353		
LY6E	-0.297563235448146		
MAPKAPK5-AS1	0.397435540551965		
SLC16A1.4	-0.269154337719154		
TAF1D.1	0.356219781148127		
C8orf33	0.398726014905098		
HMGCL	0.254724149864872		
UQCRFS1	0.338364894483714		
RETREG2	0.257222604993303		
RSRP1.1	0.380584895938924		
MPLKIP	0.391709662808764		
ZFX	0.263257341384954		
MTCH2.2	-0.288037579701788		
PCM1.2	-0.298427391577946		
JOSD2.1	0.308028350156497		
ELAVL1.3	-0.251988908316142		
MRPS33	0.332613323944936		
RRBP1.4	-0.265675788670436		
TP53I13.2	0.343723208691944		
NDUFB3	0.296651687688234		
PDLIM2.5	0.354933327020993		
NKAP	0.349750279267778		
NAP1L4	-0.271265435964221		
RUVBL1.4	-0.297562282293144		
IPO9	-0.255016896824517		
RWDD1	0.31048468502817		
HMGCS1.2	-0.364116260554718		
PSMD12	0.309624918931124		
TRIM52-AS1	0.289926065577453		
TRAPPC4	0.367997194700657		
CALD1.5	-0.816459725467158		
CCDC88A.3	-0.301157087730817		
STK19	0.268963031124539		
RAD23A	0.364459130474857		
MRPL52	0.295844449218008		
TRIAP1.3	0.381527239906507		
CBR1.3	0.281223931615404		
SEPTIN2.4	-0.257769854640348		
COX7B.4	0.253270253648322		
APP.3	-0.341188424170496		
SYAP1.2	0.342215976273793		
BX890604.2.3	-0.279950432954767		
FBL.4	-0.33981752146728		
MORF4L2.1	0.321632381880631		
NDUFB2.1	0.254786209697426		
CHD2.2	0.398570495600967		
RAB4A	-0.252521611020036		
ANP32E.3	-0.394231154062244		
MAGEH1	0.291158837507695		
ATP6V1E1.1	0.346748083925151		
NQO2.2	-0.286042590020288		
C12orf57.3	0.250355502515875		
TUBA1C.3	-0.354651010993601		
ATP5F1B.3	-0.277091278452162		
NGRN.2	0.265295554180786		
PHF14.3	-0.301616336618262		
SORBS2.6	0.317718355638454		
MRPL57.2	0.274111202527432		
ARRDC3	0.500896138260611		
ID2.2	-0.343133132712009		
TSC22D1.4	0.283883299237384		
JUND.2	0.361190408160051		
SEM1.1	0.276200952633625		
ASCC3.1	-0.253974365333733		
NECAP2	0.324057953554725		
PBX1.3	-0.447573027182336		
SERPINE1.2	0.370790433740482		
PSMB6.1	0.283270626446515		
TMEM101	0.25318826688517		
LDHA.3	-0.490741988122161		
QKI.3	0.287253155357309		
TAF13	0.302651853265674		
TOP2B.2	-0.284300953329949		
CSDE1.2	-0.285507013418843		
TFDP2.3	-0.258603285081376		
RTN3.2	-0.322228552111094		
UQCC3.2	0.353441640153302		
NPM1.5	-0.405348107219483		
C5orf24.1	0.336188455442233		
CCDC12	0.339951768397754		
SLAIN2	0.297096577235543		
ATP5F1A.2	-0.26696658129302		
SURF6	0.312458674096107		
CACYBP.3	-0.382587664335578		
CTSL.2	0.343307456600528		
ATF3.2	0.550810347714654		
RNASEH2C.2	-0.25096785587513		
DAAM1.4	-0.301644629300363		
PURB	0.28980352215831		
SRM.3	-0.280948230386783		
PDCD6.1	0.263880969245318		
EEF1G	-0.410479042934101		
NEXN.3	-0.293492659791037		
THUMPD1	0.407882970456736		

FADS1.3	-0.384315137911595
LINC00632.2	0.33239832284977
COL18A1.3	-0.265975161404985
H2AFY.3	-0.365008492113427
MRPL41.1	0.275143548164163
FNBP1L.2	-0.306173264897015
PLPPR3.3	-0.313824664343544
CHMP5.1	0.263782800012776
TNFRSF1A.3	0.293189953101244
CEBPD.1	0.269828545094475
FEZ2.4	0.282481766206859
KMT2E.2	0.303273195555427
COX14	0.350286355430117
S100A16.4	0.388584242904286
BAX	0.252579622674143
ENO1.3	-0.274428066186614
NCBP2AS2.1	0.395413201306038
C16orf91	0.354332403233967
EPM2AIP1	0.326241635133221
FABP7.6	-0.789819362296865
WDR34.2	-0.263768231865257
YY1	0.285291866789525
PDGFB.2	0.347242203730133
COL4A2.5	-0.411421034983552
LSM6.3	-0.259820341094641
MT1X.6	-0.448159721977173
SEPHS2	0.384290816166277
NONO.2	-0.269706939677231
INA.5	-0.61900506144067
AC027644.3	0.338638595384088
LINC02802.1	0.349937066065316
CHMP2A.1	0.262221479437822
CBX6	0.269828648175041
ZCRB1	0.288163693359429
TAGLN.4	-1.36302355796223
FUS.4	-0.305455974129882
MGST1.3	0.523456016295528
RHOB.3	-0.254394800473069
PRKRIP1	0.310767344306994
ZNF580.1	0.345323705706862
ZNF292.3	0.34413485078504
LAMTOR3	0.289246553134768
GADD45B.1	0.410340867529611
RP9	0.358534764883617
UACA.2	-0.343798877187732
BLVRB.3	0.402235769828195
AKIRIN2.1	0.355449066641836
AIMP1	0.341761335325902
ADPRHL2	0.294284363911442
DHX9.3	-0.267250399927745
RNF187	0.361988496052424
CSNK1A1.1	0.255679904219877
NDUFA8	0.308095742970599
COIL	0.372429253783591
BUD23.2	0.343222712343447
IER3.5	0.347205344472235
NCL.5	-0.359384143013244
CLNS1A.2	-0.27308456590326
TMEM87A	0.30720437085652
ARPC5L	0.316317730889118
SVIL.4	-0.300640405199133
C12orf73	0.361032839918676
DNAJA1.2	0.255247530439468
C1orf35.1	0.298761464923625
NEU1	0.332657840885781
TRAPPC3	0.31126144546559
MRPL27	0.287374752752501
CRK	0.311464059092262
UGP2.4	-0.336833277910382
CREB3	0.288636823489135
PPA1.2	-0.376988760954043
MTHFD2L	0.334044566631403
IFI6.5	0.253104250714349
HDAC2.3	-0.254851675046581
CHD7.4	-0.254039007702888
POU3F2.4	-0.317306514005663
RAB9A.2	0.333342909284628
C12orf75.3	-0.30875998368087
DSTN.4	-0.252696838183347
TCEAL8.1	0.346190787065256
TSSC4	0.327718724034747
EIF1B.2	0.364238019330011
ATXN7L3B.3	0.315119789647306
UHMK1	0.303545266909161
BBC3	0.382764111065134
SMS.4	-0.404051914418028
RPL36AL.1	0.253150080274817
RAB22A.1	0.269628659160216
PAFAH1B3.3	-0.335523428165866
TAX1BP1.3	0.259359240003128
IK	0.264455970298185
PRRC2C.3	-0.262963605797959
CDKN2A.4	0.341977894724421
SDHAF2	0.303438971241233
CYTOR.5	0.305932995473546
ID1.4	-0.295407059083949
MRPS26.2	0.255285372980289
ZMAT3.3	0.272996976734931
KRT18.6	-1.04063210107577
LLPH.1	0.354537355929569
EPS8.3	0.338563252661491
ZNF433-AS1	0.354898406566875
NAA20.1	0.288554807666069
OTUD6B-AS1	0.333431280765837
DCBLD2.4	0.438207625487483
ZMAT2	0.26099800129015
IGFBP5.2	0.633408923385356
SNRPB2.2	0.285704414452929
MRPL49	0.273145002443541
GADD45A.4	0.69880498724609
RAB30-DT	0.290872975982157
NKIRAS2	0.315962297700137
CAMK2N1.6	0.370963645245288
ID4.6	-0.434343266712333
MRPL28	0.277343105735834
RND3.3	0.392230480332979
BLOC1S6	0.306939390447495
GCLM.2	0.376095984190433
SYT1.5	-0.61431424701197
SESN3.3	-0.258119169601722
RBIS.1	0.250219233278922
MT-CO1.4	-0.283433833376951
PSMD8	0.271568286676859
NSRP1	0.36313263471852
PGRMC2.2	0.319085528269439
PRDM2	0.270073940147401
HMGA1.5	-0.397428729026565
AKAP12.5	0.288940439880625
PCF11	0.319789566529842
AKAP8L	0.304479163652029
MRFAP1L1	0.318221795279113
POLR1D	0.329664864574415
SERINC1.3	0.259865005245168
HNRNPM.5	-0.32212961021364
NRBP1	0.309503539099644
PA2G4.4	-0.284521814106705
THUMPD3-AS1.2	0.512534432495551
ARL14EP	0.311205982055494
C9orf78	0.328026332434206
ADGRG1.1	0.269138375635549
FAM200B.2	0.269639265371787
NME4.2	-0.288971192577897
LINC00667	0.250663542014826
DPF2	0.288477454736315
TMEM158.6	0.392746763695184
ZNF322	0.311298031485957
ARF4.1	0.399801882138458
SLU7	0.330931130640649
NES.4	-0.506090848259639
MAP4K4.4	-0.282868953368286
MRPL21.2	0.256516786431073
TMEM251	0.29528262089697
CCN1.2	-0.314384768618222
NFIB.5	0.270318585639019
C2orf49	0.278342274806216
KIF21A.3	-0.26841931323616
CIB1.3	0.270187852176057
VPS25	0.269520349361341
CAMLG.1	0.31513878551975
KPNB1.3	-0.267531255416472
SELENOK	0.263970537251602
YIPF5.1	0.302365498774935
CFL2.1	0.293671841674665
SNHG15	0.501959510919749
RGS5.5	-0.285007726118164
SSRP1.3	-0.307117881981229
FGFBP3.5	-0.282733517028308
EAPP	0.326192592509321
PSMC1	0.250639295878485
KDM6B.1	0.265783679783309
PBDC1	0.301139656716759
TMEM179B.3	0.262918778338049
SOX11.4	-0.358016676565145
RRAGA	0.286131062067587
PNMA1.1	0.258831280753989
CWC25	0.256019585929573
C19orf25	0.302624857918738
SELENOT	0.29067203067086

SRSF8	0.374536254672026
GET3	0.321393998694218
RSRC2	0.322056641563409
CDKN2B.2	0.277345250239896
MIR99AHG.3	0.280441270727617
C1QBP.4	-0.264810860068294
MRPL11.4	-0.281849155638883
PDCD2	0.263593853593029
FSCN1.1	-0.258880474082365
MPHOSPH10.1	0.316736231922598
DAZAP2	0.25598660624752
MAP2.3	-0.420101348135389
UBE2B.1	0.268897932440493
PNKD	0.253625420188237
LINC00472.4	0.296208676045272
PLEKHJ1	0.259639771177437
SMIM30.1	0.295278509894263
CCDC50.5	-0.272452548024175
YIF1A.2	0.254094375990878
SREK1IP1	0.31160509653025
MSANTD3	0.280562304712559
M6PR.1	0.272428103848434
ZCCHC10	0.29995069941557
SNW1	0.295190314271405
MRPS18B	0.320278354481431
ATP2B1.4	-0.270834665655847
KIF9.1	0.272070938494194
CRIPT	0.311628898465722
MED29	0.255032963818757
CFAP97	0.277660595877467
SESN2	0.302820669292123
MTLN.1	0.315590429142611
CEBPB.1	0.326037674994679
TCF7L2	-0.256471719600144
TRIP11	0.284254013481862
KLF6.2	0.250353225513365
CEP170.3	-0.258913088892052
DNTTIP2	0.290802097694372
CCN2.5	-0.578556993510531
RRAGD.2	0.266079840713223
SLC39A7.3	0.251754967121268
SFT2D1.1	0.250697867944147
MAF.1	0.287648936521072
USP16	0.26762423258549
SCNM1	0.251235588536329
NGDN	0.262569087045859
UTP3	0.298838473839652
MED19	0.271380403209463
TMEM205	0.272951442405888
KANSL1.1	0.28481730640927
HIST1H1E.2	-0.281993728212413
ZNF622	0.265079473009062
CD164.2	0.268807078139531
IER5L.3	0.349490419570692
WDR45B	0.299833752762144
SMIM15	0.28771485354958
ARHGAP5.2	0.273274960397801
PKIB	-0.257209290940398
ARMCX6	0.256368880003704
EMC3	0.253692172655299
PITHD1	0.317235705983795
FUNDC1.1	0.26514774772456
PDLIM4.6	0.254692885213923
ITGAV.5	-0.270569103893987
NEFM.6	-1.91619598732579
TMEM126A	0.314423377619176
COL11A1.6	-0.288708965207445
PPP1R2	0.302710643433344
GAR1.2	0.28194322011265
GOLGA4.1	0.253728694876182
CCDC59.1	0.346021637830148
FAM32A	0.250803947627637
TMF1	0.255649877392593
GSPT2	0.302525213066803
TTYH1.5	-0.355295006735034
DLL3.6	-1.34457703806432
MRPS10	0.275196146247058
YPEL5.3	0.261810442854346
MRPL38	0.284082261143557
BAG1.1	0.253571881050357
TSR2	0.284003854189849
SMIM10L1	0.294073355925123
KLHL9.1	0.257145721886859
C14orf119	0.257583682815471
ZNF644	0.258718720274234
TOPORS	0.271160335933095
PON2.4	-0.260123611041301
MGAT4B.3	0.254091026273903
PLTP.5	-0.252345347003049
URM1	0.2561039195163
MIAT.6	-0.268417554323653
AUP1	0.276329626825727
TWISTNB.2	0.275747936087423
BLOC1S4	0.278005449480373
MAP1A.3	0.316105874839747
TUSC2	0.270583207818679
BCAS2	0.25791747469018
JUNB.2	0.300137996722679
HSP90AA1.2	-0.279813286795727
SNHG32	0.280053278129232
RBBP6.1	0.333817046671544
LRIF1	0.253724873870934
MFAP1	0.273773639221057
MYH9.5	-0.282589936016925
SAC3D1.2	0.301158773083948
ZFAND2A	0.263880708942666
RCAN1.5	0.369034695948152
ZNF593	0.263941990031885
CCNL1.1	0.2975767887555
ANP32B.4	-0.256094383518013
MT-ND4.3	-0.296511201012685
TTC3.5	-0.368764955648444
COA6.2	0.261595778323822
C1D	0.274590518605493
IER2	0.4097233386242
ARL6IP1.3	-0.281133874313254
KLC1.4	-0.259808741409491
S100A10.6	0.357490524338384
VGF.3	0.772583110080412
SOD2.6	0.34527154469857
HNRNPU.2	-0.274057583644089
MT-ATP6.3	-0.356593135856494
ZFP36L2.1	0.251245549351671
NFKBIA.4	0.321491383537977
FLNA.5	-0.2996536739711
ACTN1.5	-0.314976116132998
COL4A1.5	-0.353250101816285
KHDRBS3.1	0.274724275679486
CCND1.3	0.300795398470892
SPP1.4	-0.322196816383002
NRP2.4	0.281801164871456
IFRD1.1	0.267927824702757
A2M.6	-0.307563202054664
PHLDA2.6	0.441130136315978
MALAT1.4	-2.46776431352434
FTH1.7	1.04377784960308
RPS15.2	0.631366173370259
FTL.6	1.11493578525415
RPS12.3	0.725404893000324
RPL36.2	0.708718008160536
RPL28.2	0.624455410102177
RPL41.2	0.632542176780037
RPS24.4	0.693113994818679
RPL39.1	0.663007652349993
RPS18.4	0.67977042244754
RPS28.2	0.608610988636235
RPLP2.2	0.692245953516051
SERF2.3	0.678338819911545
RPL27A.4	0.798639586734629
RPL32.2	0.634533410733064
RPS27.2	0.708484518313627
ATP5F1E.5	0.800801717130919
RPL12.5	0.66840167607512
RPL37A.1	0.751897234239983
RPLP1.3	0.679990760952943
RPL13.3	0.594635181462168
RPL37.1	0.625054601380411
RPL30.1	0.532250916111636
RPL21.1	0.634252799517208
RPS4X.2	0.640004719453422
TPT1.3	0.651994239924411
RPS29.1	0.649350948462631
RPS21.2	0.567334790610363
RPS19.1	0.691779839665724
RPL29.2	0.548635265207324
UBA52.3	0.598300347691873
NME2.3	0.603199983644843
RPL11.3	0.512901246746226
RPS15A.3	0.529862969590766
RPL13A.1	0.689768645612106
RPL18A.4	0.660662134850357
RPL34.1	0.555941515364607
RPL23A.2	0.640051151177667
RPL6.2	0.596673959305214
SNHG29	0.721507324828225
IFITM3.6	0.6227500581545
FAU.1	0.447902395475586
RPS14.2	0.477643680758502

RPS5.5	0.540604077677417
H3F3B.4	-0.516806155558609
RPS26.5	0.589555797374168
RPLP0.6	0.604065989845051
RPL10.2	0.513560314448191
RPL35.3	0.643590397437111
RPS6.5	0.545745917304195
RPL9.1	0.547838325757652
OST4.5	0.621678271816908
PPDPF.4	0.607624006549205
RPL8.4	0.463134290995519
RPS8.2	0.434853417683773
RPS3A.4	0.500115536135614
RPS2.5	0.799011170429345
PFDN5.2	0.532646022976882
TMSB10.4	0.539762769055868
RPL26.3	0.458567479840257
RPL3.2	0.57372344000018
RPS9.2	0.539324416129624
RPL22.3	0.519075943264908
RPL27.2	0.554148898524685
S100A10.7	0.792921826123323
RPS25.2	0.466043539157286
MIF.3	0.524916733628985
RPS27A.3	0.394262245900041
SH3BGRL3.5	0.605555132694262
RPS13.2	0.485538462049399
RPL19.3	0.427065527005123
RPL35A.2	0.422910515381653
GAPDH.4	0.536086366127203
RPL36A.4	0.518217927077262
PTMA.5	-0.594181167316884
MT-CO3.4	-0.664592329538841
RPS11.2	0.448721921759542
RPS20.2	0.476775048248927
CHCHD2.7	0.459422517244351
RPSA.5	0.544597020626227
EFNA5.4	-0.443060087433971
RPS16.3	0.471893257970216
GUK1.3	0.575831112350344
UQCRQ.5	0.5477339791936
CNTFR.5	-0.435983622645655
LIN28B.4	-0.462123216776634
NACA.1	0.400496785988926
SSBP2.3	-0.39082951797856
BRD8	-0.376117065666711
C17orf75.1	-0.370118592116478
RPS3.6	0.408555960253861
TMA7.2	0.468068209227492
SMAD1.1	-0.386937171601332
RPL10A.5	0.521603898658363
UQCR11.3	0.483643107447953
RPL31.1	0.500202241648803
ELOB	0.472511473925588
ZC2HC1A.2	-0.424303091987632
METRN.3	0.576591058694542
TRIM71.3	-0.453748572957997
RPL24.3	0.356251060982783
TMSB4X.5	0.736455109336564
SET.5	-0.449652495636519
RAB8B.1	-0.359229693900619
ZNF322.1	-0.39559326369022
ARL6IP6.1	-0.341430850384171
GULP1.1	-0.327758423683584
MARCKS.3	-0.447058396855316
TEAD2.1	-0.344362911977023
EIF1AY.6	-0.45399774770987
C1orf52	-0.378579365839941
TLK1	-0.392139689693243
ODF2L.2	-0.347887762999563
PAK1.2	-0.381174779609938
PPIL3.1	-0.338248035119474
PCSK1N.6	0.38192416177322
JKAMP.1	-0.360599652217266
SEZ6L2.5	-0.49479706421289
UNC50.1	-0.339743349643274
FUT8.1	-0.351167126375701
CECR2.4	-0.381622188267744
SHOC2.1	-0.351787524575798
SPPL2A.2	-0.332788910100352
CEBPG	-0.352020670427347
GLT8D1.1	-0.338783971778449
SYNRG	-0.373043115702108
EBLN3P	-0.319306384082147
CAAP1.1	-0.319376431482163
STX2.2	-0.369401220520834
EEF1G.1	0.618529565863883
EBAG9.1	-0.313959552814051
ZNF518A	-0.368680289304838
C5orf15	-0.329345207584786
RPL38.1	0.432770447350774
ATOX1.3	0.499588718867916
CALM2.5	-0.493311330887129
PURA.1	-0.311786936566096
EEF1AKMT2.1	-0.278801149851711
RPL17.5	0.413610982911243
TMEM60.1	-0.310897511411515
RPL7.3	0.417302811648036
RBM7.1	-0.308821750997236
MMD.2	-0.350110766347998
NGDN.1	-0.332120822513452
UBE2Q2.2	-0.303492958650022
XRCC5.5	-0.384211967178404
C12orf65.1	-0.349925717665563
NUCKS1.6	-0.452052818783996
WNK3	-0.368339238606924
UFSP2.1	-0.279154791488009
NR6A1.5	-0.449362367013335
MTA3.1	-0.324958021627399
PCMTD1.1	-0.325186494989329
VPS4B	-0.350965409242431
GNAI1.2	-0.313319744995854
TMEM170A.1	-0.344542275293977
SRP14.3	0.375707395383748
VIM.5	0.431446539949178
OSTM1.2	-0.316696589251009
BET1.1	-0.293679009109002
TUBB.5	-0.411330662964197
FUNDC1.2	-0.283978264890426
IGFBP7.5	0.369668855943287
BICD1.1	-0.396370118469083
RPL18.2	0.365365883101243
PDCL.1	-0.295408750442788
JPT1.4	-0.365465449165479
UBA5	-0.316549694695976
IFT74.1	-0.275022307523554
PIP4P2.2	-0.316339179952203
C17orf58.1	-0.288490262464666
MFAP2.4	-0.338192909849094
SLC2A3.3	-0.423268100289807
BMERB1.3	-0.329586901118565
ICK.1	-0.339065706499571
IFT27	-0.317926377153619
CCDC174	-0.309332173416421
MTRNR2L12.5	0.656081557804733
MTLN.2	-0.292859988302053
GPX4.2	0.426790331228143
ING2.1	-0.315337990329646
PTPN2.1	-0.326409347400456
PPIP5K2.1	-0.329464825423945
TXNDC15.1	-0.30934402268552
CHMP2B	-0.304717271609188
UBXN2A	-0.322650231606035
ITGB3BP.2	-0.309719732969345
MTRES1.1	-0.292321751635308
MIER1	-0.344219871724981
ILF3-DT.1	-0.359112912551814
ARFGAP3.2	-0.297284001966712
SRPRA.1	-0.28532087426414
NQO2.3	-0.365944712046082
GOLT1B.2	-0.291929002762835
FBXW11	-0.310991492556381
SON.1	-0.363360324157901
CITED2.2	-0.401575704603387
IST1	-0.325632332151535
RPS23.4	0.347115963440752
BBS7.1	-0.279140802004093
HINT1.1	0.350118496634237
CLDND1.1	-0.313317094495742
FOXN3	-0.34871398513729
NENF.4	0.38231872401247
KRIT1	-0.272016722353652
STAM.1	-0.285372751043406
ACTR6.1	-0.292007442534805
CUL2	-0.329327767589543
C9orf16.4	0.473888230639579
GTF2A1	-0.305056207882081
NUP54	-0.324467474450999
G2E3	-0.33163319750492
GSPT2.1	-0.348352202457611
SMAD4	-0.317922824748318
FAM104A	-0.291555294883483
PITPNA-AS1.1	-0.313986236068448
YAF2.1	-0.299329382135989
SCG3.6	-0.647053539870988
UBE2D1.2	-0.329594841314127
RUFY2	-0.283607970919642
PKIA.5	-0.428089494496012

NRBF2	-0.347246932598538
ZCCHC7.1	-0.283072714952316
FABP7.7	0.261494907918035
HSD17B4.1	-0.280349892770037
HIST1H1E.3	-0.495859240523497
RAB9A.3	-0.320803104678415
RACK1.2	0.402167686873091
MICU1.1	-0.262453825204904
BAG5	-0.35373719683587
TM9SF1.2	-0.263018061384111
YBX1.3	0.427888004659256
MRC2.3	-0.308971000057603
TM9SF2	-0.285351061298437
GOLGA2	-0.31524847299864
CCSER2.1	-0.329396845844681
CNOT4	-0.307128530884328
WASL	-0.330163134072539
OBI1	-0.316138756052595
UBL3.2	-0.348675387336347
PSPH.1	-0.271161640817013
SNN.1	-0.313293289391749
RAB13.5	0.554446818071387
NSMCE3.1	-0.318145393525558
GTF2B	-0.305000676388175
CSNK1G3	-0.336320695104546
MNAT1	-0.302515705807366
KIF1BP	-0.30411098948487
EPN2	-0.329945957081981
SMG6.1	-0.316368108978711
KLHDC2	-0.311475710552633
KIN	-0.286547270904894
YBEY.1	-0.25500322961226
CWC22	-0.271306236396508
TMED7	-0.287370598101942
TNKS2	-0.308556374398156
PHF5A.1	-0.277299531851945
FAM126A.1	-0.303630337167109
WDR33	-0.338862570266089
RNF214	-0.269158068369225
SPIN1	-0.330892502492936
MZT2B.2	0.45780143856324
SUDS3	-0.326411091599586
CHD6	-0.309079175389403
RIOK2	-0.289966593821882
RPL15.4	0.360257468916897
NAPG.1	-0.292578893117897
BTBD7	-0.316970477310136
HIST1H1C.3	-0.422466725871124
ACBD5.2	-0.276617898475969
DOK5.3	-0.335737629119821
MAPKAPK5-AS1.1	-0.282397978088411
DOCK7	-0.3230242024568
STX8.1	-0.256648190719805
ACAP2	-0.324605011445754
CTTNBP2NL.1	-0.33705164596315
ZNF720	-0.289388903277911
KDSR	-0.308842369085687
BCL10.1	-0.253846969221806
DNAJC15.6	-0.375184324524543
GSTZ1.1	-0.264073524616041
PTBP3.1	-0.357629227804151
ADAM10	-0.290380968201046
YEATS4	-0.285326457539866
RPL7A.2	0.352022299509341
SCAMP2.1	-0.2586409513534
SMNDC1	-0.323670534091643
IFT52.1	-0.251762417984157
DERA.3	-0.314060701245862
MITD1	-0.290545244267977
MAML2.3	-0.375569377016253
RPL14.4	0.424940633284474
PRKDC.5	-0.409082332470592
TMEM30A	-0.297225681257386
SLC25A24.1	-0.316818890985568
USP3.1	-0.303727727924215
COMMD6.4	0.410086244837162
DNAJB14	-0.326125133828679
TIMP1.6	0.355884139313594
MRFAP1L1.1	-0.275705215155261
PITPNB	-0.332102133138036
FNBP1	-0.299232416951672
USP33	-0.315459350579193
KLF3.1	-0.291402229401214
TSPAN13.2	-0.334356725342506
IFNAR1.2	-0.294863008881431
MARCKSL1.6	-0.397082316297158
MYL6.4	0.3503265882095
RPL5.4	0.318268774349252
C1GALT1.1	-0.288605948146334
VPS36	-0.313004365920714
SPTLC1	-0.263409985708745
ZCCHC9	-0.273014943477814
PTGES3.4	-0.329947114405061
MED31.2	-0.268004448600498
MYO1B	-0.347599064871419
ANXA2.5	0.366530467745934
TBC1D7.1	-0.2623296337474
SRSF3.4	-0.452492181568045
WASF1.2	-0.342649501166188
B4GALT5	-0.323840035121478
WDR41.1	-0.275821992697635
KIAA1143	-0.274713438790294
SEPHS2.1	-0.294339857512296
ARMT1.1	-0.28068444972386
UBE2E2	-0.270339862759966
ATMIN	-0.283371442710753
VIRMA	-0.296887331093178
CDYL	-0.296987894859096
ATG5	-0.259406493368703
SP3	-0.2815922335338
SECISBP2	-0.299222327649869
TMEM106B	-0.271878431473242
HMGN4	-0.304150759223666
LRIF1.1	-0.281425076521975
TOR1A	-0.302770934225741
SS18L2.2	-0.300446226050315
RBM22	-0.33030491770057
METTL2A	-0.278976918264664
CDC40	-0.303652930979659
MARCH7	-0.321714397382631
NIPA2	-0.283112343285169
LAMTOR3.1	-0.265479420944501
FRG1	-0.311738618415587
TP53RK	-0.270194393093219
GOPC	-0.346276208828568
ANKIB1	-0.287794616561903
BLMH.1	-0.297896525909744
CREB1	-0.353427730556374
DCUN1D1	-0.256244210594602
SMIM12	-0.299309028356932
ZMYND11	-0.271999667749166
DSEL	-0.30473407331284
STMN2.7	-1.71498488630341
EID2	-0.280055792252123
RSBN1L	-0.355919785144015
KATNBL1	-0.31250630134477
DNAAF2	-0.262596272829032
ACVR2B.3	-0.31602362930325
BCL7A.2	-0.334815090131421
YAP1.2	-0.263394759816477
C16orf87	-0.309384371097935
PRPSAP1	-0.28203025449381
ZFHX3.3	-0.35999417736021
C12orf75.4	0.407337783737463
TLK2	-0.302145092916322
NUB1	-0.279572340502914
ADGRL2	-0.288868571851221
TMEM87A.1	-0.282135080370217
LRRFIP2	-0.32158604748804
PARD3	-0.293482790012079
COIL.1	-0.283179611555158
SYPL1.3	-0.254381390354768
WAC-AS1	-0.283289488516661
SLC25A13.4	-0.325610975723318
STRN3	-0.279001008146446
DDX50.1	-0.330763967296313
CBL	-0.27571101761658
CRBN	-0.290112742045726
KIAA0232	-0.280994677196109
ZNF431	-0.302412826318934
PLAGL1.5	-0.396465796767215
SUCLA2	-0.297530095433464
SMARCAD1.1	-0.318259471251896
ARMC1	-0.325934943859542
ADAM17.1	-0.295031984287737
SNHG6.1	0.495305506250467
TULP4.1	-0.309252777360504
PWWP3A	-0.312020647734463
ARFGEF1	-0.310960499549396
DTWD1	-0.308388474206242
PPAT.4	-0.356584473982243
MT-CO2.5	-0.386813790021882
H3F3A.6	-0.281989767428643
SLAIN2.1	-0.299107483914505
SPECC1	-0.284884362420721
CASP6.1	-0.278097921784557
LINC00665.2	-0.297808448814002
PRKRA	-0.283642491129362

BNIP2	-0.288279100357565
TSPYL4.2	-0.34252513529322
PPP1R10	-0.32862534268003
TTF1	-0.311155271060537
EXOSC5.3	-0.258562589441397
SAR1B	-0.284055566319796
PDS5B	-0.312736557325991
EMC2	-0.255152453491255
ZMYM2	-0.309383491894492
ZC3H7A	-0.285147272480852
BCAP29.1	-0.27152120083443
ICMT	-0.26803904873686
RECQL.1	-0.31122254253792
CERT1	-0.306786941760528
RMDN1	-0.278818330067009
GDAP1.1	-0.308184639262049
NEPRO	-0.263865360864275
DR1	-0.304976947991571
MARS	-0.294354303649101
UNG.3	-0.319830571845622
UBA6	-0.286220142435952
CNOT2	-0.291953191806569
SOCS4.1	-0.314515901461716
EHMT1	-0.319039185368419
KLF7.3	-0.330944201564067
CDCA7L.5	-0.27645009663866
RPS7.3	0.312036543130527
ICE1	-0.329093060258999
MSH2.3	-0.37785975942025
SH3BP4.4	-0.31260342571053
SERBP1.5	-0.334503579538661
TRA2A	-0.291284355857536
RBM33	-0.272398644806507
RAD50	-0.319595264134089
MRPL16.1	-0.328392841549442
PPP1R14B	0.454311844574605
ARL8B.1	-0.269407451262936
CHTOP	-0.307846169468065
TOPORS.1	-0.309114383097225
NUFIP1	-0.264650310268644
SHPRH	-0.332247720430969
SGO2.2	-0.414982616162272
ACSL4	-0.278075189710635
MRPS31	-0.278838948454632
XIAP	-0.283152022143365
MT-ATP6.4	-0.471562295067891
FAM98B	-0.25003131953351
REEP3.2	-0.261358775459167
CLUAP1	-0.255667912137556
ZNF738.1	-0.272634511029662
NSMCE4A.2	-0.304631274474099
SYMPK	-0.261361826767634
TCF12.2	-0.357254019021828
IMPAD1	-0.258504620177777
YIPF6	-0.284452807122665
RAD51C.4	-0.329204487783785
PKN2	-0.337060622193221
PPP6C	-0.252501655808694
PDCD6IP	-0.266401743279956
SERINC3	-0.293575856060735
TGFBR1.2	-0.320050975216802
H2AFZ.7	-0.587399814514562
CDC73	-0.309664115535704
R3HDM1.1	-0.276540105071665
ELAVL2.5	-0.479722674881364
CIAO2A.1	-0.271724163406528
SRRM2.1	-0.336519007108136
BMS1.1	-0.302675668454189
FNBP4	-0.321171012635286
MBTPS1	-0.263822010739546
ORC4	-0.291906122943778
ZNF618	-0.291588865270548
PHPT1.3	0.396676984770583
EPB41L2.5	-0.320827397516233
SMC6.1	-0.305993506707596
NBN	-0.279719087772397
MAN2A1.1	-0.281905031101874
OAT	-0.263910154624766
UBXN7	-0.291734002986789
QSER1	-0.336513730766658
KLF6.3	-0.310221246112858
RPS17.6	0.324525721651482
NUTM2A-AS1	-0.269973262482077
STRBP.3	-0.324328478108699
EXOC1	-0.252170051235476
TTC19	-0.288868571851221
ZC3H14	-0.313472215016228
POLR2L.1	0.462649818448393
ATAD1.1	-0.25008719360384
C1D.1	-0.264500817904619
NFYB.2	-0.323965699422845
ALCAM.3	-0.340730733778851
NKAPD1	-0.254940001120213
ZBED5	-0.281465339286423
MED6	-0.262945818476505
RBM28.1	-0.302459016249302
ETNK1.1	-0.321720174858135
PDCD4	-0.27646729112812
SLBP.1	-0.344533099977048
FIP1L1	-0.30742172204419
NUDT15.3	-0.293637754681161
UFL1	-0.282536225613146
RABEP1	-0.313273619680276
ARF6	-0.275333679035937
UBLCP1	-0.292081379790795
MIB1.1	-0.362318330654232
BRWD1	-0.266995089799208
RFC2.5	-0.340113856100396
BMPR2.1	-0.299903488937679
KLHL7	-0.263576829340468
AP3B1	-0.290797922060852
VPS26A	-0.315358612541293
DNM1L	-0.314206855325737
COMMD3.3	-0.27180842816169
LARP4	-0.290304990605735
ZNF721	-0.281885972526658
TMEM223.1	-0.284772371283496
H2AFJ.2	-0.277823233674004
PDCD7.1	-0.338962290168363
ZNRD1.2	-0.256490922509036
TSPO.5	0.27532874885808
TBPL1.2	-0.311944156828422
PCYOX1.1	-0.283360987403805
CCNL1.2	-0.290203574143212
UTP6	-0.310331041596264
NUP58	-0.291569493253284
RNF8	-0.269358860065948
TIMM23	-0.26707823522943
KANSL1.2	-0.334239138905611
KHSRP.1	-0.301780802368523
CCDC144NL-AS1.3	-0.339627208657564
GPCPD1.2	-0.320137071364853
ILKAP	-0.250302041163842
N4BP2.1	-0.334537060950771
NCL.6	-0.4730992902054
JAGN1.1	-0.287334500136096
UBE2G1	-0.281138604200923
CDK5RAP2.1	-0.281825134931382
SNRPD1.5	-0.374682859449419
EPB41.4	-0.406558429655777
YIPF4	-0.284940407228414
COPS4	-0.263455599478781
C19orf25.1	-0.26638198398952
ABCA1.3	-0.344559185809051
RBMX2	-0.263378761249492
CCDC43	-0.288821644191933
TRIM59.1	-0.334829867905291
MKRN1	-0.255648368401179
RRP1B.2	-0.348835631236417
CDK12	-0.326293342026146
NF1	-0.264575382772321
SRFBP1	-0.261434634281995
STX16	-0.287936648968567
EXOSC3.3	-0.311448502398833
FBXO11	-0.269598325130927
MYO6	-0.274677812144812
PTAR1	-0.271176074225128
CTSL.3	-0.253227606978481
GPATCH8	-0.290441408880775
SEPTIN6.2	-0.280012913162563
CFAP20	-0.307837993281384
PAGR1.1	-0.302526685089579
IRF2BPL.2	-0.320554548105623
AFG3L2	-0.33366543887522
ZNF622.1	-0.258509318796524
MPP6.3	-0.280276570805565
EIF4EBP2	-0.2928361515458
USP15	-0.271248997556188
LEO1	-0.256557325099499
UTP18.1	-0.263481376353683
NUP50	-0.283127661046167
CMIP	-0.284692634391674
ATF7IP.1	-0.308774562088278
BLOC1S4.1	-0.265566327261037
NRIP1.2	-0.379979748601339
EXOSC9.4	-0.3202106836616
XPOT	-0.25290362498896
CHN1	-0.250813983106425

RAB21	-0.273553157750267
TMEM219.3	0.31355498982237
KAT6B.1	-0.302310034336073
ZNF131	-0.269972007687738
FAM98A	-0.266716768202555
DHPS.2	-0.250066610503733
STARD7	-0.277910055903747
COL1A1.4	-0.544045292537391
SOX4.5	-0.637087194781062
DDX27	-0.301213573680217
ZC3H18	-0.310408485593636
ELOVL6.1	-0.31986805024063
MND1.5	-0.322592381493114
ROBO1.3	-0.339671046724223
ME2.1	-0.293281649367249
ZNF91	-0.291075246555694
ZNHIT6	-0.263934881806264
CCNK	-0.277456035857895
GATAD1	-0.297579166343657
PLPBP	-0.251854876134902
VEZF1	-0.267015647637984
VAPB	-0.263939757538508
IREB2	-0.279525663775846
TXN.2	0.349432838064551
IFRD1.2	-0.293056726334066
HSP90AB1.5	-0.301375206773701
CCDC82.1	-0.274055311506806
MT1X.7	-0.517889788297036
ERP44	-0.25756709438669
AGO3	-0.261562643610808
NOL11.1	-0.292230986731401
BARD1.4	-0.307381308834006
MBD4	-0.265295642798781
SNX4	-0.257753045798611
TMEM203	-0.253471457215063
PHKB.1	-0.270499963985117
HNRNPA3.6	-0.37261522943457
TMX4.2	-0.279304248257229
TACC1.3	-0.287298712936746
HERC2	-0.293079961948907
TXLNA	-0.269807930073864
MRPS22	-0.262153775146546
MAT2A.1	-0.332584467406121
GNB4.1	-0.282261989952622
ERICH1	-0.294346764263787
NUDCD1	-0.258786055927697
RHOC.6	0.323790076243833
ZNF451	-0.274065689691526
GON4L	-0.299075584981407
NEXN.4	-0.37161070312691
HSBP1.1	0.367134006129159
HMGB1.6	-0.430631931986276
CASP8AP2.1	-0.295659098535359
USP46	-0.276382724851855
NT5C3A.1	-0.274318824824959
GSK3B.1	-0.301687900010655
BRD9	-0.256271222014118
GABRB3.3	-0.359717759263386
TSPAN18.4	-0.325460492881225
HNRNPDL.2	-0.310298173584748
PPP4R3A	-0.292488524171713
CCDC6	-0.273658264508531
MIA3	-0.333373085388522
GALNT7.1	-0.287817069431088
RBM39	-0.306501344651786
FBXO21	-0.270054839660562
IARS2	-0.291407388322942
DNAJA1.3	-0.303590797015157
CALM1.4	-0.251886635771637
ADNP	-0.305863297926864
THUMPD1.1	-0.295609812414215
UBQLN1	-0.267095303807171
NRAS.1	-0.261462315155844
CNOT1	-0.269993961633094
ZEB2.2	-0.349976282012127
PRRC2C.4	-0.375116254951762
RNF216	-0.268856691235854
CPSF3.1	-0.262986238460695
SCO1	-0.27562614487013
CASK.1	-0.254869946962624
SLU7.1	-0.253262564445226
CKAP5.2	-0.351224961660077
DEK.6	-0.497939622648818
TMEM161B-AS1	-0.286927986792553
FNTA	-0.271346118522843
OSBPL9	-0.270973207986412
PIK3C2A	-0.301721951545422
SPDL1.1	-0.307372347356684
SMDT1.4	0.374501921161364
THUMPD3	-0.282733696024451
CUL1	-0.261208608468922
SMIM15.1	-0.266935954411158
SUPT6H	-0.250444656906079
PNO1.1	-0.279798876707387
ANKHD1	-0.283928247823373
ATP6V1A.1	-0.271771866940346
SUGP2	-0.263870839312507
SAP30.1	-0.280300999876403
EEF1A1.2	0.395163118971266
SLC38A2	-0.312915389781528
HNRNPAB.5	-0.421848183600032
SLC16A9.1	-0.28747918165334
RSRC1	-0.284845108206949
SC5D.1	-0.322798512201222
GOLIM4.1	-0.296447786662111
CACUL1	-0.298633432462065
TNRC6A.1	-0.304929332174331
COPS9.4	0.360476984886122
TSR1.3	-0.260681968306807
SFXN1.1	-0.262970333400985
SETD2	-0.313699697675282
PLEKHA1.1	-0.271749418511029
RBM6	-0.312348209083901
RFC3.3	-0.257685677128838
RP9.1	-0.255746389871717
UBN1	-0.286761151580886
PODXL.3	-0.316486807202776
DDIT4.1	-0.307403345783064
NUP62.1	-0.304419618191773
NEFM.7	-1.84413222124155
KAT6A	-0.264486596056957
AGO1.3	-0.294692605071759
CST3.5	0.377965803081617
TGS1.4	-0.32453977249159
AASS.1	-0.287594731564969
ANK2.5	-0.443665032233011
UBE2J2	-0.269805631597439
STAG2	-0.306927875366284
TRIM33	-0.279772364826982
FAF2	-0.289244468292736
BRD3	-0.28124628056554
PANK3	-0.261172214520717
PRR14L	-0.282559086796251
HMGXB4.1	-0.271602626015642
SRGAP1.1	-0.328330550513766
AGO2	-0.292484760997683
ATG101.1	-0.252523511356277
MAP1B.5	-0.630166127908157
PCNX4.2	-0.317282336756263
NUDT3.1	-0.309824723010378
PHF23	-0.268253228076834
ZNF22	-0.286328080966814
RYBP	-0.260196296626536
PLCG2.4	-0.325234937076558
PRRC2A	-0.272782235676431
NDUFAF8.2	0.4243122781395
HSPB1.6	0.370832376468527
LCOR.1	-0.333497450995498
KLHL9.2	-0.285522405032257
MT-ND3.4	-0.342066408919455
RSRP1.2	-0.275663033051953
TMF1.1	-0.298333985228291
DPF2.1	-0.265446119552819
MED13L.1	-0.286632836142915
GRPEL1.1	-0.272443748759518
PRDM2.1	-0.271224267086369
BAZ2A	-0.255581211197484
SERPINE2.1	-0.320474676970478
WAPL	-0.264156663816462
SDHD.2	-0.257162616365864
SPEN	-0.295003665068304
KLHL42.1	-0.265170700830683
MTF2.2	-0.328150148491225
KIF3A.2	-0.318336228722632
PTPN1	-0.272069741015973
PBX3.3	-0.28368720926389
KIAA1586	-0.265767678252862
HCG18.1	-0.286050383316076
HMGN5.2	-0.265498365044675
PPP1R15A.3	-0.318081778536421
EML4.1	-0.258708383809597
C1orf122.4	0.356672589123112
SLC4A7.1	-0.332293583396236
CTR9	-0.284525924542723
GNL2.1	-0.255469448630396
SFSWAP	-0.258677884063892
PRKCI.2	-0.299426296630561
FTX.2	-0.352531771087609

MEIS3.2	-0.279019816313301
GNG4.5	-0.385008730007825
CCDC14.2	-0.281984497049782
LTV1.1	-0.258353148616316
RNF114	-0.252367963831815
EPM2AIP1.1	-0.260426152730798
EIF5.4	-0.279533390311519
CMAS	-0.266986718762346
SNHG8.3	0.347165404030779
PGM2L1.3	-0.414684914710819
RNF115	-0.259881500766624
CEP290.1	-0.335750300113112
NAA15.1	-0.273540260688067
RBFA.1	-0.274638100042471
OARD1.1	-0.272873000995365
AATF.1	-0.26178744310083
SRRM1.2	-0.268258072716769
ZNF146	-0.266768105739536
CEP350	-0.291228885479627
TIA1.1	-0.295805964597394
SRSF11.1	-0.254534558224452
HLTF	-0.281032959153758
UGCG.1	-0.30860117885824
SHTN1	-0.250944339865538
FBXO5.4	-0.32578267907314
SMAD2	-0.251011618527259
NSD2.2	-0.264931976865393
POLR2D.2	-0.269732851096044
WDR82	-0.308202363550451
DNAJC1.2	-0.275663525532373
SUZ12	-0.289071798778701
IFITM2.6	0.353656474891369
KHDRBS1.4	-0.300678119076256
EEF1D.3	0.398526058124844
HNRNPA2B1.4	-0.345157774100551
STK4	-0.252969425388909
CCNA2.6	-0.40040522938889
PRDX5.5	0.33556849936296
RAD51AP1.5	-0.298499468999854
PSIP1.5	-0.355356464085276
KMT5B	-0.278256355911459
RFC4.5	-0.318626972387765
MMAB.2	-0.250989853849648
TTC14	-0.26211591697923
IWS1	-0.289816173021928
IFT81.1	-0.254601903469301
ASPM.4	-0.544398084068579
MICOS13.5	0.338475963305684
ZMYM4	-0.286749684367749
RBM5.1	-0.285534701854742
HYAL2	-0.263989351783454
RPAP2	-0.292855926533679
NCBP3	-0.276122246704597
CEP78.2	-0.278205263743677
CHORDC1.1	-0.277601270174285
ANKLE2	-0.267811642396663
CHD1.1	-0.316294390903063
ARL14EP.1	-0.268026624468702
ZNF608.1	-0.275434626451017
HNRNPUL2	-0.275471596333408
FAM107B.1	-0.306562615396192
PHACTR4	-0.280051462415581
HECTD1	-0.255863630700344
DPM1	-0.265890802701203
RAP2B.1	-0.282126737991753
EPS8.4	-0.334113039631323
CHEK1.3	-0.274245466848041
HAUS6.1	-0.253781375641156
NIP7.2	-0.25731008046739
GIGYF2	-0.272546594350749
RTN4.4	-0.344282648682824
MSH6.3	-0.383981350063405
EXOSC6	-0.250177239244881
ZNF827	-0.267443595976916
SOD1.1	0.336821696393832
ARRDC3.1	-0.267357738101938
HILPDA.2	-0.262296018523942
SDAD1	-0.262880710387693
ELAVL3.5	-0.568829371579863
VEZT	-0.274990397631371
SMC3.3	-0.360074090634115
DHX15	-0.263538354751335
ANXA5.5	0.321126960956343
LYAR.4	-0.306545829825455
NOC3L.1	-0.265787275797765
ZNF286A	-0.262087419017948
COL5A2.5	-0.310090764581147
ST5	-0.254732847713396
RBM27	-0.291188703222856
SEPTIN10.3	-0.264686924708081
GOT2	-0.255097043563821
CFAP97.1	-0.263052683549961
EXT1.1	-0.280986239630329
CPSF6.3	-0.261287755144372
SMARCC2.1	-0.271811523446103
PDS5A	-0.254462185150307
ATAD5.3	-0.26153787599252
MCM3.5	-0.365793881186239
GTSE1.3	-0.359863228401128
CLIP1.2	-0.301308267822173
HNRNPD.5	-0.389326278988352
CTPS1.2	-0.262254848157996
TPR.3	-0.333106857415292
CAV1.7	-0.262912070352774
RNF145	-0.251671074912058
SLK	-0.260819578038073
CD46	-0.250669181919002
SPG7.1	-0.250500640703513
ZNF24	-0.266519742048641
AHCTF1.1	-0.289953735973383
DBF4.5	-0.386509741854379
VRK1.5	-0.343993579089875
TMX1	-0.261459782453215
JADE1.2	-0.261681831705118
PLEKHO1	-0.288906547043702
POGZ	-0.272919376244667
HNRNPU.3	-0.446583112384819
SEC14L1.2	-0.259784514573473
COL6A1.3	-0.261387495098054
AURKB.5	-0.320564772495662
PRC1.2	-0.372881044109541
SBNO1	-0.28267531187471
ADD3.3	-0.278754335253923
CDC42EP3.4	-0.273573251975485
BRK1.2	0.310964972852623
FADS2.1	-0.260625370954217
ODC1.4	-0.308265514117574
NOL8	-0.258627180096356
RGS10.5	-0.401292072600943
PAXBP1	-0.251318934321675
MCM4.5	-0.296964391035332
NPC2.5	0.332028899731802
CBX5.3	-0.31958803900366
KMT2C.1	-0.267062163547598
CHML	-0.26112205653482
TCOF1.6	-0.29213031123436
KIF22.2	-0.251086483507266
ZFP36L2.2	-0.280586581954843
YLPM1	-0.25452398231117
GPATCH2L.1	-0.277623334360561
AMOTL1	-0.262582735805454
TET1.4	-0.339461187631943
POLD3.2	-0.258063279385724
SMARCA5.4	-0.292852647208047
BX890604.2.4	-0.254268845927822
USP34	-0.258635766973286
PPIG.2	-0.263472908056519
NCAPG.4	-0.358104503923076
NASP.5	-0.621949048658002
PHF20	-0.257762965533133
RHOB.4	-0.360758591510703
PTK2	-0.283104841743742
APC.2	-0.365600773771547
LRRN1.3	-0.323339388616249
PIMREG.5	-0.336612201414019
MARCH6.2	-0.289338717380921
MYBL2.5	-0.301101357075285
NUF2.2	-0.326119150452315
TNIK.3	-0.286978524271104
KLHL23.4	-0.331252522471805
GAS5.2	0.422432046858073
CHAF1A.4	-0.277111594625043
UBL5.1	0.301324577126591
RRS1.1	-0.269604574885323
POLR2J3.1	-0.27829107675693
ZFAS1.1	0.320486454795296
SNHG17.3	-0.256112960331244
COX6A1.2	0.319497510079435
HMGN2.5	-0.350916639284333
PTPRF.2	-0.253820272883487
CYSTM1.3	0.287733438466365
ATP6V1F.5	0.32986731760301
AMD1.1	-0.29254223927551
LINC00461.4	-0.351823297267737
CENPK.5	-0.272025940272383
STAT1.4	-0.283530208988482
NAV1.3	-0.300235252724522
RRM2.5	-0.348765122013582

NME4.3	0.326619828149929
KIF20B.5	-0.407115547802102
HES1.2	-0.255939443516186
POU2F1.1	-0.253156381074275
CHD8	-0.293197751091425
YWHAH.4	-0.274940716432003
PEA15.5	0.285589726444186
CCL2.6	-0.638719970376635
MIS18A.5	-0.284357674772139
NUDT21.2	-0.275448772802803
KIF11.3	-0.286339924772708
CYBA.6	-0.456555936787859
FASN.3	-0.263932256594768
HACD3.3	-0.269273513127051
HSPA8.3	-0.310009903515534
CAND1	-0.262785364671296
ROMO1.1	0.374334496340519
PAK1IP1.2	-0.256182792600155
ATAD2.4	-0.328832100356432
MGST3.4	0.278753340438832
PA2G4.5	-0.269112227433468
CCPG1.4	-0.280088134817971
CENPE.5	-0.48123624753398
COX6B1.2	0.295537566924286
FEN1.4	-0.297927542643629
SFPQ.4	-0.389701386614514
GART.4	-0.261420844605776
MCM6.5	-0.263132631005359
EIF3A.3	-0.312772421814778
GLI3.3	-0.252599091223621
SMCHD1	-0.28044476765484
RPS10.1	0.36712571989234
USP10	-0.258628199979113
CTCF.1	-0.293420310177611
NDUFA1.2	0.368218268135726
SLC16A1.5	-0.283856377059269
ATP5MG.1	0.281262361791034
MMP24OS.3	0.396499310156308
NIN.2	-0.271119688389403
ZWINT.5	-0.258535162649698
MAPK6.1	-0.288086869143578
CD63.6	0.269414838899692
NDUFA13.1	0.254130177921171
ATXN2	-0.265302864938568
SLC39A6.1	-0.253357777943125
ARHGAP21.1	-0.282349647374398
CDKN3.5	-0.340086601190969
CCDC112.1	-0.262192064507486
HSP90B1.3	-0.349730937255937
TP53BP1.1	-0.282465473387084
BASP1.4	-0.454342426830675
ATP5ME.3	0.300924038111967
TGIF1.3	-0.250373400290018
MZT2A	0.317427144492299
DKC1.4	-0.269177014024682
MIAT.7	-0.508694895930935
ASH1L.1	-0.257482360700592
RCAN1.6	-0.336138922918718
SNRPF.5	-0.270226502132065
ATP5MD.1	0.268673564182727
DDX5.2	-0.251161183227436
SRGAP3.4	-0.400276575509039
PRTG.6	-0.454176687996418
TOMM7.4	0.265544672109834
MIS18BP1.5	-0.370203753109154
CDC20.5	-0.415178636047348
ATF3.3	-0.309956935246089
COX6C.2	0.29499014676519
ATP5F1D.1	0.274571090176821
GLRX.4	-0.26705966045187
ACLY.1	-0.258967550560377
SLC25A36	-0.258072003301636
INA.6	-0.676754423103985
SQLE.1	-0.375519191600349
SNCA.3	-0.252700329361362
H2AFY2.3	-0.251039003550216
SACS.1	-0.29769164934917
MT-ND4.4	-0.318871747437775
BAZ1A.1	-0.27404938253963
SNX3.4	0.25262341226862
SELENOW.2	0.267378704057848
CD151.4	0.31500348193738
MYEF2.1	-0.274697135378676
HNRNPR.4	-0.25742287528868
DLGAP5.5	-0.428637932877949
CRABP1.7	-0.502825083224209
KPNB1.4	-0.294480836629202
ARGLU1.3	-0.313921694987548
UQCRB.4	0.296177784584197
PMP22.6	-0.401959796204464
SUPT16H.3	-0.291274218032434
NETO2.1	-0.278308890826582
GNAQ.1	-0.26483355211618
SIPA1L2.3	-0.273081693966437
DICER1.2	-0.277288670309565
DYNLL2.2	-0.254387909799376
NSD1.2	-0.250260139282593
JARID2.3	-0.322412959043869
SINHCAF.5	-0.360430806342422
USP1.3	-0.299703158253014
U2SURP.2	-0.256271178709061
EZH2.3	-0.280581622638318
NDUFA3	0.360318803624238
FUS.5	-0.290033195547151
FOXP1	-0.255419201658936
TSTD1.7	-0.390719455082832
GLUL.2	-0.262621066661094
TAF10	0.31741287484224
DRAP1.3	0.283631074816917
ZMYND8.3	-0.296336294888114
ZIC2	-0.259531714114191
LUC7L3.3	-0.255855381267932
NDUFA11.1	0.280599961920466
SNHG14.5	-0.481304986489858
LBR.4	-0.312397618861151
RNF19A.5	-0.379248835791677
ATP5MF.1	0.277566306486
PMEPA1.4	-0.282745571584844
ACAT2.5	-0.381792504878659
SLC25A6.2	0.36671901586832
GTF2I.4	-0.283798307618649
RANBP1.7	-0.368349709249476
HMGB2.6	-0.740755410960196
SFRP2.6	-0.474680996757768
HSPD1.6	-0.35550400370409
MXRA7.3	0.350787973264621
CDK1.7	-0.414746172212554
RABAC1.4	0.253183666967037
MKI67.3	-0.530243069612594
HMGCR.4	-0.333457314881741
SSR4.3	0.271557588253153
POU5F1.6	-0.591138342776983
SRSF2.5	-0.291180148089562
SMC2.2	-0.284487968117946
FBLN1.3	-0.267418940401904
NCAM1.6	-0.451188916182947
NDUFA7	0.282502872591445
GAP43.5	-0.291636689027306
BAZ2B.3	-0.281426320436833
MYL6B	0.309501770183336
TMEFF1.3	-0.372748352265148
TUBB2A.5	-0.346561649445185
HCFC1R1.2	0.276860945059179
SEC61G.3	0.274798365902888
HMMR.5	-0.442670242262045
PRDX2.5	0.274688083953935
FAAP20.2	0.267007906213481
PALM2-AKAP2.2	-0.347634044904939
HIST1H4C.6	-1.27553310290129
RBM25.3	-0.253654816677265
S100B.1	0.320516443845644
MAP6.3	-0.273540260688067
FDFT1.4	-0.282995623734122
UACA.3	-0.257514277050091
G3BP2.2	-0.263610303821269
TUT4.1	-0.270414905865875
SRSF7.5	-0.333169206082882
TLE1.1	-0.273929031662465
LIX1.6	-0.360307875436891
IFI27L2.3	0.252699733707287
HSPA5.2	-0.367395135568476
INPP5F.5	-0.289632335992068
MYCBP2.2	-0.292516576614248
SPP1.5	-0.506670743745467
CELF2.1	-0.253169288976635
AP2M1.2	0.286544224587381
AUTS2.2	-0.260825193223504
RRM1.4	-0.292663342499336
SYNE2.3	-0.332689912820407
UBE2S.6	-0.461006336004365
SPRY1.4	-0.257633544418707
TXNIP.2	-0.318403709973451
PIK3R3.3	-0.342251605906557
CETN2.3	0.285047578418853
ARHGAP29.5	-0.405280482630547
TCERG1.2	-0.254687091232807
POU3F2.5	-0.357649969793545
ITGB1.6	-0.285422704766982

SNHG25.2	0.253518827045823
HNRNPM.6	-0.268347802833141
SCP2.2	0.256753762394791
FNBP1L.3	-0.271309922954707
STMN1.6	-0.487431675582921
EIF3F	0.291284796535691
TUBA1A.5	-0.441377992157851
HMGB3.5	-0.259051563666307
APOE.7	-0.521509180080129
PNISR.2	-0.252557701074407
ARL6IP1.4	-0.374416652398899
MT-ND1.4	-0.366495507966282
BLOC1S1	0.264875828511187
TCAF1.3	-0.270446637873356
PNN.3	-0.262935586627442
SOX2.2	-0.299165259592573
ANKRD12.4	-0.269300155677881
RIF1.4	-0.257302286689429
XIST.5	-0.39165027840286
KRT18.7	-0.717571196424365
TUBB2B.4	-0.31315788525963
DLL3.7	-0.882510651425815
RPA3.6	-0.2867136966867
UBE2C.6	-0.593479617081675
ANKRD11.3	-0.28048704612736
NTRK2.6	-0.292281994610706
IGF2BP1.4	-0.36264049739983
IGDCC3.7	-0.477289026721605
DAAM1.5	-0.282808547092821
ANP32E.4	-0.271805947311453
NMU.5	-0.332481517313497
CD24.6	-0.560752763117804
GABPB1-AS1.2	-0.255579544242216
LIN28A.3	-0.365517887836613
NRP2.5	-0.288478403308848
LMNB1.4	-0.294211719930627
NKAIN4.5	-0.311356767998745
TNC.4	-0.300818355728722
HLA-B.6	-0.50145218617868
CDH2.4	-0.269998845767254
DRAXIN.4	-0.278887007691618
NKTR.2	-0.266452753910117
ARL4C.6	-0.283683472631608
RND3.4	-0.284099511166676
CCNB2.5	-0.376291758971214
ID3.4	-0.697868687841757
CKAP2.3	-0.273709872487556
KDM5B.3	-0.253285788123337
TMPO.3	-0.330276571495132
RBFOX2.4	-0.256029793044332
CKS1B.7	-0.303374056580628
SESN3.4	-0.262464725717219
ORC6.6	-0.279719891354608
NEFL.6	-0.813757687544352
HLA-C.5	-0.258888164454595
ATP1B1.3	-0.302980704712761
RUFY3.4	-0.273523280926765
TUBB3.7	-0.502588552915256
ID2.3	-0.385211581549418
CAMK2N1.7	-0.502428742822924
CCN2.6	-0.679590536461458
KIDINS220.4	-0.277396089309585
NES.5	-0.327057410526581
FN1.6	-0.514240713749503
HMGA2.4	-0.277727556553127
DUSP6.4	-0.265564131646726
WSB1.4	-0.29517124147357
DPPA4.4	-0.298894146384965
KRT8.6	-0.828747752572309
ITGAV.6	-0.256528727683729
SOX11.5	-0.393333488256109
MYC.3	0.651268491272471
CRABP2.5	-0.298653128602881
CCNB1.6	-0.376676897097465
TOP2A.5	-0.518091302572116
SOD2.7	-0.403873958626007

Table S2 IPA upstream analysis of untreated samples vs. mean of untreated opposite group

Upstream Regulators	all_control	BIH040_ctrl_vs_all_progessive	BIH246_ctrl_vs_all_progessive	BIHi046_ctrl_vs_all_progessive	BIHi047_ctrl_vs_all_benign	BIH237_ctrl_vs_all_benign	BIHi039_ctrl_vs_all_benign
EPO	1,091	4,916	1,856	N/A	1,633	1,915	0,059
IFNG	-0,433	2,662	4,049	N/A	N/A	1,259	N/A
BDNF	0,854	3,565	2,403	N/A	-1,812	1,516	0,113
homocysteine	N/A	3,063	2,729	N/A	-1,131	0,883	N/A
romidepsin	N/A	2,605	2,967	N/A	N/A	N/A	N/A
arsenic trioxide	N/A	3,096	2,181	N/A	N/A	0,57	1,963
TCR	0,896	2,556	2,692	N/A	3,536	-0,185	-2,55
BCL6	N/A	2,683	2,542	N/A	N/A	N/A	-1,969
staurosporine	N/A	3,201	1,94	N/A	N/A	N/A	N/A
ammonium	N/A	2,863	2,137	N/A	N/A	0	N/A
MEL T1	N/A	2,958	2	N/A	N/A	N/A	N /A
PRKAR1A	N/A	2,961	1,987	N/A	N/A	N/A	N/A
CAT	N/A	3,569	1,23	N/A	N/A	N/A -1,103	
Ap1	N/A	2,81	1,983	N/A	N/A	N/A	N/A
selenomethylselenocysteine	N/A	1,996	2,627	N/A	N/A	-0,447	N/A
tert-butyl hydroperoxide	N/A	2,746	1,726	N/A	N/A	N/A	N/A
AKT3	N/A	4,654	-0,243	N/A	N/A	N/A	N/A
HOXA10	N/A	2,734	1,667	N/A	N/A	0	N/A
MAPK9	N/A	2,835	1,554	N/A	N/A	1,854	-0,338
thioacetamide	0,327	2,264	1,97	N/A	N/A	2,15	N/A
IGFBP2	N/A	2,035	2,164	N/A	N/A	0,272	N/A
LEP	0,792	2,174	1,98	N/A	N/A	-1,012	0,775
2-deoxyglucose	N/A	1,378	2,768	N/A	N/A	N/A	N/A
BCL2L1	N/A	2,183	1,932	N/A	N/A	N/A	N/A
YBX1	N/A	2,667	1,414	N/A	N/A	N/A	N/A
PLAU	-1,015	1,302	2,735	N/A	N/A	N/A	N/A
CD247	N/A	2,019	1,976	N/A	0	N/A	N/A
nitrofurantoin	0,73	1,98	1,987	N/A	1	0,671	-1
hexachlorobenzene	0,898	2,578	1,387	N/A	N/A	1,89	N/A
daidzein	N/A	3,08	0,883	N/A	N/A	N/A	N/A
PNPLA2	N/A	1,953	1,969	N/A	N/A	N/A	N/A
TMPRSS2-ERG	N/A	2,382	1,469	N/A	N/A	N/A	N/A
cyclophosphamide	-0,643	3,117	0,724	N/A	N/A	-0,2	N/A
potassium chloride	N/A	2,852	0,923	N/A	N/A	N/A	N/A
IL9	N/A	2,36	1,387	N/A	N/A	-1,982	N/A
PTBP1	N/A	2,605	1,129	N/A	N/A	0	N/A
mycophenolic acid	N/A	2,15	1,508	N/A	N/A	0	N/A
MAPK14	-0,849	2,09	1,56	N/A	N/A	N/A	N/A
cycloheximide	-0,213	1,669	1,98	N/A	0,396	-0,155	1,769
FOXO3	0,121	1,633	1,945	N/A	N/A	N/A -	0,626
2-bromoethylamine	0,718	2,399	1,166	N/A	N/A	2,121	N/A
MAP2K4	N/A	1,45	2	N/A	N/A	N/A	N/A
PITX2	N/A	2,012	1,423	N/A	N/A	N/A	-2
DGCR8	N/A	1,764	1,664	N/A	N/A	N/A	N/A
MLXIPL	2,449	6,199	2,286	-3,402	6,877	-1,604	-6,083
vinorelbine	N/A	1,207	2,121	N/A	N/A	N/A	N/A
SNAI1	-2,008	2,454	0,873	N/A	N/A	N/A	N/A
ETV5	-2,433	1,206	2,041	N/A	N/A	-2,219	N/A
imiquimod	N/A	2,97	0,187	N/A	N/A	N/A	N/A
YY1	N/A	1,458	1,671	N/A	N/A	N/A	N/A
FBXW7	1,414	1,993	1,131	N/A	-1,89	-3,302	N/A
AMPK	1,238	1,225	1,89	N/A	N/A	-2,646	-1,508
estrogen receptor	0	1,551	1,557	N/A	N/A	-1,32	-1,387
bufalin	N/A	2,255	0,823	N/A	N/A	N/A	N/A
NUPR1	0,378	1,956	1,067	N/A	N/A	N/A	N /A
SAV1	N/A	1,872	2,65	0	N/A	N/A	N /A
FHIT	N/A	1,107	1,897	N/A	N/A	N/A	N /A
3,3'-diindolylmethane	N/A	3,224	1,257	0	N/A	N/A	N /A
TUG1	N/A	2,219	0,762	N/A	N/A	N/A	N /A
ATG7	N/A	1,761	1,195	N/A	N/A	0,404	N/A
ILF3	0	0,973	1,972	N/A	N/A	-1	N/A
calcitriol	N/A	1,557	1,387	N/A	N/A	-0,126	N/A
PTP4A1	-1,584	1,845	1,091	N/A	-2,178	0,735	-0,831
wortmannin	-0,115	2,845	1,752	-0,217	N/A	-1,245	-1,52
spermidine	N/A	1,565	1,346	N/A	N/A	N/A	N/A
SLC13A1	N/A	3,71	2,988	-2,333	1,342	N/A	N/A
SP110	-0,447	1,808	1,046	N/A	N/A	-0,707	0
metribolone	N/A	0,843	2	N/A	2,335	1,675	-1,753
miR-145-5p (and other miRNAs w/seed UCCAGUU)	N/A	2,765	0,068	N/A	N/A	N/A	N/A
methylmercury	N/A	3,039	1,704	-0,577	N/A	N/A	N /A
phenacetin	0,543	1,309	1,432	N/A	N/A	1,664	N/A
L-threonine	N/A	1,537	1,195	N/A	N/A	N/A	N /A
mir-210	N/A	1,119	1,609	N/A	N/A	N/A	N /A
tyrphostin AG490	-1,741	1,703	1	N/A	N/A	N/A	N/A
CAB39L	N/A	4,667	2,807	-3,439	3,873	1,633	-2,273
(1S,2R)-NCL-1	N/A	1,455	1,226	N/A	N/A	N/A	N/A
L-triiodothyronine	2,183	0,389	2,219	N/A	N/A	-0,773	N/A
BMP7	N/A	1,997	0,606	N/A	N/A	N/A	N/A
methamphetamine	N/A	2,33	0,268	N/A	N/A	1,934	N/A
leupeptin	N/A	2,359	0,171	N/A	N/A	N/A	N/A
bortezomib	N/A	0,518	2	N/A	-1,195	N/A	0 , 2 4 2
RELB	N/A	0,832	1,664	N/A	N/A	N/A	N/A
niacinamide	N/A	1,026	1,455	N/A	N/A	N/A	0
25-hydroxycholesterol	N/A	1,379	1,095	N/A	N/A	N/A	N/A
GATA1	N/A	0,896	1,565	N/A	N/A	-2,183	-0,832
LDB1	N/A	1,457	1	N/A	N/A	N/A	N/A
mir-148	N/A	1,121	1,265	N/A	N/A	N/A	N/A
indirubin	N/A	1,254	1,719	0,563	N/A	N/A	N/A
CD44	-0,349	1,274	1,076	N/A	N/A	0,862	N/A
FN1	0,304	1,218	1,109	N/A	N/A	0,82	N/A
diazoxide	N/A	1,818	0,507	N/A	N/A	N/A	N/A
methyl-beta-cyclodextrin	N/A	1,701	0,61	N/A	N/A	N/A	N/A
PAX3-FOXO1	-0,832	0,916	1,387	N/A	0,607	-1,929	-0,707
levothyroxine	N/A	1,297	1	N/A	N/A	N/A	N/A
LDHB	N/A	1,398	0,895	N/A	N/A	N/A	N/A
doxorubicin	-0,06	1,767	0,509	N/A	N/A	2,203	1,766
mir-322	N/A	0,963	1,289	N/A	N/A	N/A	N/A
tempol	N/A	1,992	0,254	N/A	N/A	N/A	N/A
TNFSF13	N/A	1,253	0,958	N/A	N/A	N/A	N/A
ACVRL1	N/A	2,097	0,103	N/A	N/A	N/A	N/A
MXD1	N/A	2,997	1,667	-1,372	-0,447	N/A	N/A
GSKJ4	-2,53	2,004	1,265	0	2,121	2,887	N/A
DETA-NONOate	N/A	1,79	2,416	-0,975	N/A	N/A	N/A
nitroprusside	N/A	1,394	0,753	N/A	N/A	N/A	N/A
Ca2+	-0,896	1,134	1	N/A	N/A	0,799	N/A
JAK2	N/A	0,549	1,55	N/A	N/A	N/A 0,689	
Raf	N/A	1,303	0,794	N/A	N/A	N/A	N/A
Ubiquitin	N/A	2,504	0,742	-0,128	N/A	N/A	N/A
CREBZF	N/A	0,655	N/A	1,414	N/A	N/A	N/A
GnRH analog	N/A	0,57	1,414	N/A	-0,333	-1,414	N/A
PML	N/A	1,219	0,762	N/A	-1,342	-1,39	N/A
DSCAM	N/A	1,353	0,623	N/A	N/A	N/A	N/A
ZNF148	N/A	2,191	-0,631	1,387	N/A	N/A	N/A
PSEN2	N/A	0,504	1,46	N/A	N/A	N/A	N/A
melatonin	-1,121	1,48	0,447	N/A	N/A	N/A	N/A
THZ2	N/A	1,02	2,059	-0,199	N/A	N/A	N/A
mir-193	N/A	1,912	0	N/A	N/A	N/A	N/A
isobutylmethylxanthine	N/A	-0,088	2	N/A	N/A	1,342	N/A
adenosine	N/A	1,756	0,152	N/A	N/A	N/A	N/A
oleic acid	N/A	1,424	0,448	N/A	N/A	N/A	N/A
bicalutamide	N/A	1,454	2,338	-1	N/A	N/A	N/A
Esrra	N/A	2,513	-0,707	N/A	N/A	N/A	N/A
PD 153035	N/A	0,414	1,387	N/A	N/A	N/A	N/A
tamoxifen	-1,404	0,9	0,9	N/A	0,426	0,374	1,763
CDKN1B	N/A	1,817	-0,028	N/A	N/A	N/A	N/A
Pkc(s)	N/A	0,206	0,474	2	N/A	N/A	N/A
luteolin	N/A	0,883	0,661	1,134	N/A	N/A	N/A
KDM5B	N/A	1,222	0,559	N/A	N/A	-1	N/A
STK11	N/A	0,597	1,172	N/A	2,138	0,971	-1,492
allopurinol	0,898	0,768	1	N/A	N/A	2,138	N/A
PUF60	N/A	0,459	1,303	N/A	N/A	N/A	N/A
EDN3	N/A	0,417	1,345	N/A	N/A	N/A	N/A
STAT5B	-0,447	1,19	0,555	N/A	2,121	-0,653	-1,414
betulinic acid	N/A	2,322	-1,681	1,96	N/A	N/A	N/A
LMX1B	N/A	1,943	0,802	-0,184	N/A	N/A	N/A
inosine	0,324	0,788	0,908	N/A	-0,655	1,4	N/A
gambogic acid	N/A	2,1	-0,407	N/A	N/A	N/A	N/A
MSX2	N/A	0,983	0,686	N/A	N/A	N/A	N/A
CYB5R4	N/A	N/A	0,307	1,36	N/A	N/A	N/A
PDGF BB	0,416	2,196	-0,55	N/A	N/A	1,391	1,536
miR-124-3p (and other miRNAs w/seed AAGGCAC)	N/A	1,216	2,371	-1,118	N/A	-2,64	N/A
IPMK	0	1,426	0,218	N/A	N/A	2,2	N/A
SKP2	N/A	0,778	0,831	N/A	N/A	N/A 0	781
NSUN6	N/A	1,19	0,388	N/A	N/A	2	N/A
N1,N11-diethylnorspermine	-2	1,707	-0,156	N/A	N/A	2	N/A
salubrinal	N/A	1,549	0	N/A	N/A	N/A	N/A
Hbb-b1	N/A	1,95	2,599	-2,236	3	N/A -	2
RB1	N/A	1,562	0,304	0,447	2,646	0,447	-2,233
PRKCQ	N/A	0,695	0,845	N/A	N/A	N/A	N/A
STAT5A	N/A	1,086	0,447	N/A	N/A	N/A	N /A
AXIN1	N/A	2,213	-0,688	N/A	N/A	N/A	N /A
PD 169316	N/A	1,947	-0,436	N/A	N/A	N/A	N /A
piceatannol	N/A	1,492	0	N/A	N/A	N/A	N /A
miR-338-3p (miRNAs w/seed CCAGCAU)	N/A	0,478	1,643	0,101	N/A	N/A	N /A
imipramine blue	N/A	0,563	0,911	N/A	N/A	N/A	N /A
PTGS2	N/A	0,011	1,463	N/A	N/A	1,432	N/A
NR3C1	0	1,122	0,351	N/A	N/A	-0,342	N/A
mir-145	N/A	0,449	-0,366	2,125	N/A	N/A	N /A
LCAT	N/A	0,781	0,676	N/A	N/A	N/A	N /A
carfilzomib	N/A	1,32	0,127	N/A	N/A	N/A	N/A
OSM	-0,791	0,312	1,093	N/A	N/A	0,568	0,696
IGF1R	N/A	-0,816	2,2	N/A	2,137	-0,128	-1,168
miR-17-5p (and other miRNAs w/seed AAAGUGC)	N/A	2,21	-0,827	N/A	N/A	N/A	N/A
molybdenum disulfide	0	2,714	-2,646	2	N/A	N/A	N /A
let-7	N/A	3,023	0,981	-2	0,156	-1,661	N/A
PRKAA2	N/A	1,508	2,121	-1,633	N/A	N/A	N/A
TRAP1	N/A	0,24	1,067	N/A	-0,555	N/A	0,152
CDK2	N/A	1,509	-0,218	N/A	N/A	N/A	N/A

TAS4464	N/A	0,989	0,294	N/A	N/A	N/A	N /A
NRF1	N/A	0,808	0,46	N/A	N/A	N/A	N / A
CRTC1	N/A	0,367	0,887	N/A	N/A	N/A	N/A
bexarotene	1	1,214	0,025	N/A	N/A	0,632	-0,762
CDK1	N/A	0,788	0,45	N/A	N/A	N/A	N/A
calphostin C	N/A	0,832	1	0	N/A	N/A	N/A
TXNIP	N/A	1,155	0,059	N/A	N/A	N/A	N/A
miR-122-5p (miRNAs w/seed GGAGUGU)	N/A	1,213	N/A	0	N/A	-0,849	N/A
APOE	1,109	1,213	0	N/A	N/A	-0,152	N/A
miR-204-5p (and other miRNAs w/seed UCCCUUU)	N/A	-0,74	0,464	2,047	N/A	N/A	N/A
oxysterol	N/A	2,184	-2,119	1,706	N/A	N/A	N/A
docetaxel	-1,987	0,049	1,131	N/A	N/A	-1,072	N/A
cadmium	N/A	0,607	0,555	N/A	N/A	N/A	N/A
PRKAA1	N/A	0,642	1,914	-0,816	N/A	N/A	N/A
triamterene	0,898	0,108	1,046	N/A	N/A	2,157	N/A
eprenetapopt	N/A	0,5	0,651	N/A	N/A	N/A	N/A
HDAC4	N/A	0,586	0,562	N/A	N/A	-0,927	N/A
BNIP3L	N/A	1,08	0,068	N/A	N/A	N/A	N/A
INSIG1	N/A	1,596	-2,121	2,236	N/A	-0,152	N/A
sulindac sulfide	N/A	1,151	-0,011	N/A	N/A	N/A	N/A
progesterone	0,287	0,503	0,623	N/A	N/A	1,659	-0,138
fulvestrant	0,686	0,153	1,069	0,447	N/A	-0,728	N/A
HEXIM1	N/A	1,489	-0,386	N/A	N/A	N/A	N/A
FGFR1	0,822	0,649	0,447	N/A	N/A	1,982	N/A
TGFBR2	-0,174	1,342	-0,816	1	N/A	-0,156	N/A
gentamicin C	0,898	0,306	0,689	N/A	N/A	2,138	N/A
vancomycin	0,49	0,029	0,956	N/A	N/A	1,554	N/A
PSMB11	-1,667	0,73	0,594	0,13	2,449	1,667	N/A
LIN28B	N/A	-0,192	1,158	N/A	N/A	N/A	N/A
zerumbone	N/A	0,846	0,105	N/A	N/A	N/A	N/A
SB203580	1,876	2,255	1,609	-2,439	N/A	-1,362	N/A
TRPC4AP	N/A	1,552	-0,07	-0,137	N/A	N/A	N/A
BMS-690514	N/A	0,334	0,763	0,243	N/A	N/A	N/A
Y 27632	0,64	0,413	0,472	N/A	N/A	N/A	N/A
HNF4A	-0,447	-0,075	0,946	N/A	0	-0,113	N/A
tretinoin	-0,089	-0,066	0,931	N/A	0,589	-0,424	1,295
E2F3	N/A	-0,168	1,026	N/A	2,236	-0,128	-1,732
miR-16-5p (and other miRNAs w/seed AGCAGCA)	N/A	2,282	0,957	-1,982	N/A	-2,01	N/A
PTP4A3	N/A	1,281	-0,447	N/A	N/A	N/A	N /A
HDAC2	N/A	0,8	0	N/A	N/A	N/A	N /A
fenretinide	N/A	0,308	0,482	N/A	N/A	0,218	N/A
miR-2392 (miRNAs w/seed AGGAUGG)	-2,441	2,386	-0,511	-0,718	1,992	1,995	N/A
NVP-TAE684	N/A	0,585	0,184	N/A	N/A	N/A	N /A
ciprofloxacin	N/A	0,763	1,523	-1,139	1	N/A	N/A
HRAS	0,933	0,389	0,373	N/A	2,068	-0,374	-0,511
temsirolimus	N/A	1,22	-1,363	1,236	N/A	N/A	N/A
delta-aminolevulinic acid	N/A	-0,169	0,239	1	N/A	N/A	N/A
TMEM126A	N/A	1,016	0,478	-0,447	N/A	N/A	N/A
CHUK	N/A	-1,517	2,215	N/A	N/A	N/A	1,446
CCN5	1,067	0,392	0,302	N/A	N/A	N/A	N/A
RBL1	N/A	0,232	0,456	N/A	N/A	N/A	N/A
SMAD7	N/A	0,372	1,21	-0,555	N/A	1,478	-2,177
everolimus	N/A	0,483	0,201	N/A	N/A	N/A	N/A
MK2206	N/A	0,026	0,99	0	N/A	N/A	N/A
epigallocatechin-gallate	N/A	1,306	-2,333	2	N/A	N/A -0,919	
ENO1	N/A	-0,181	0,816	N/A	N/A	N/A	N/A
MDM4	N/A	0,825	-0,2	N/A	N/A	N/A	N/A
KLF2	N/A	0,759	1,139	-0,968	N/A	1,982	N/A
AZGP1	N/A	-1,127	0,374	1,665	N/A	N/A	N/A
PAX7	N/A	-0,251	0,853	N/A	N/A	N/A	N/A
caffeine	N/A	-0,468	1,053	N/A	N/A	-0,975	N/A
MYC	1,026	-0,246	0,829	N/A	5,966	0,628	-5,155
PTEN	0,572	-1,132	1,698	N/A	-2,6	-2,295	2,349
desmopressin	N/A	0,388	0,168	N/A	N/A	N/A	N/A
PHF12	N/A	N/A	0,137	0,414	N/A	N/A	N /A
CEBPA	-1,206	0,168	0,378	N/A	N/A	0,623	N/A
4-phenylbutyric acid	N/A	0,665	1,122	-0,97	N/A	N/A	N /A
C1QA	N/A	0,342	0,168	N/A	N/A	N/A	N / A
mir-183	N/A	-0,67	1,176	N/A	N/A	N/A	N /A
tacrolimus	N/A	-0,353	0,632	0,447	N/A	N/A	N /A
AGO2	-2,236	1,937	-1,167	-0,048	N/A	1,633	N/A
PIK3CA	N/A	0	0,447	N/A	N/A	N/A	N /A
HAVCR1	-0,391	0,545	-0,101	N/A	-0,577	-0,277	N/A
SUMO2	N/A	0,736	-0,326	N/A	N/A	N/A	N/A
IFNA2	0,498	0,466	-0,035	0,149	N/A	1,726	-0,017
trichloroethylene	N/A	1,792	-0,376	-0,846	N/A	1	N/A
AGN194204	N/A	-0,497	0,859	N/A	N/A	1,134	0,277
Lh	0,711	2,101	0,93	-2,495	6,183	-1,103	-5,098
E2f	N/A	-0,707	1,026	N/A	N/A	N/A	N/A
KIF3A	N/A	1,5	-1,181	N/A	N/A	N/A	N /A
PPP1R15A	N/A	1,006	-0,692	N/A	N/A	N/A	N /A
SMARCB1	0,277	0,5	-0,191	N/A	N/A	N/A	N /A
MACROH2A1	N/A	0,676	-0,372	N/A	N/A	N/A	N /A
SP2509	-0,956	1,625	-1,342	N/A	-2,366	-0,383	N/A
puromycin aminonucleoside	0,954	0,559	-0,287	N/A	N/A	1,026	N/A
LMX1A	N/A	-0,103	0,61	-0,105	N/A	N/A	N/A
U0126	0,097	-0,452	0,017	0,83	N/A	-1,762	-1,9
phorbol 12,13-didecanoate	N/A	0,82	-0,57	N/A	N/A	N/A	N/A
MAX	N/A	2,449	-2,236	N/A	N/A	N/A	N/A
mir-2392	-2,439	2,449	-2,236	N/A	1,044	2,635	N/A
budesonide	N/A	0,571	-1,929	1,664	N/A	-1,667	N/A
RPL22	N/A	0,013	-0,789	1,067	N/A	N/A	N/A
CYP2J2	N/A	0,774	-0,588	N/A	N/A	N/A	N/A
15-deoxy-delta-12,14 -PGJ 2	0	-0,747	0,926	N/A	N/A	-0,216	N/A
bardoxolone	N/A	0,243	-0,064	N/A	N/A	N/A	N/A
MAP4K4	N/A	0,528	-0,354	N/A	N/A	N/A	N/A
ROCK1	N/A	-0,56	0,711	N/A	N/A	N/A	N/A
NCD-38	N/A	-1,185	1,31	N/A	N/A	N/A	N/A
actinonin	-2,53	1,05	0	-0,896	2,828	3,162	N/A
E2F2	N/A	-2,145	2,236	N/A	N/A	N/A	N/A
IGF2BP1	N/A	N/A	-0,071	0,152	N/A	N/A	N/A
UCP1	N/A	-0,368	0,448	N/A	N/A	-1,342	N/A
RBL2	N/A	0,351	-0,277	N/A	N/A	N/A	N/A
ciglitazone	N/A	0,329	-0,269	N/A	N/A	N/A -0,579	
VHL	0,225	-0,475	0,532	N/A	N/A	N/A	N/A
RBM5	N/A	0,873	1,069	-1,89	N/A	N/A	N/A
MAFB	N/A	1,239	-1,188	-0,012	N/A	N/A	N/A
camptothecin	N/A	-1,461	1,482	N/A	N/A	2,236	N/A
indomethacin	1,155	1,98	-1,98	N/A	N/A	1,339	-0,378
PPP1R13L	N/A	0,547	-0,555	N/A	N/A	N/A	N/A
DDX3X	N/A	-0,212	-2,302	2,495	3,606	0,73	-2,333
miR-128-3p (and other miRNAs w/seed CACAGUG)	N/A	0,396	-0,447	N/A	N/A	N/A	N/A
HBA1/HBA2	N/A	1,528	0,378	-2	3	N/A	-2
SIX2	N/A	-0,067	0	N/A	N/A	N/A	N/A
UDP-D-glucose	N/A	-0,098	-0,601	0,557	N/A	N/A	N/A
RASSF1	-0,707	-0,954	0,849	N/A	N/A	-2,333	-0,447
IQGAP2	N/A	0,265	-1,503	1,027	N/A	N/A	N/A
benzene	N/A	-0,096	0,447	-0,577	N/A	N/A	N/A
betaine	N/A	-1,533	0,277	1	N/A	N/A	N/A
N-acetyl-L-cysteine	0,128	0,317	0,124	-0,707	N/A	-1,495	-0,78
Sox2ot	N/A	-0,825	0,646	N/A	N/A	N/A	N/A
BCYRN1	N/A	0,816	N/A	-1	2	N/A	N/A
RGS2	N/A	0,348	-0,791	0,152	N/A	N/A	N/A
geldanamycin	-1	-0,478	0,277	N/A	-0,429	0,272	N/A
mir-21	N/A	0,044	-0,113	-0,254	N/A	-1,238	N/A
epirubicin	N/A	2,203	-2,438	N/A	N/A	N/A	N/A
uranyl nitrate	0	0,054	-2,646	2,236	2,224	0,447	0,053
SGPP2	N/A	-0,137	1,747	-1,982	N/A	N/A	N/A
MEL S3	N/A	-0,254	0	N/A	N/A	-1	N/A
IND S7	N/A	-0,258	0	N/A	N/A	-1	N/A
BMI1	N/A	-0,06	-2,303	1,964	N/A	N/A	N/A
SEL1L	N/A	0,591	-0,862	N/A	N/A	N/A	N/A
glutamyl-Se-methylselenocysteine	N/A	-0,698	0,391	N/A	N/A	-0,378	N/A
PD98059	-0,06	0,6	-0,348	-0,781	N/A	-2,681	-1,315
PCGF2	N/A	1,633	-2	N/A	N/A	N/A	N/A
miR-155-5p (miRNAs w/seed UAAUGCU)	N/A	1,633	-2	N/A	N/A	N/A	N/A
CXCL12	N/A	0,166	-0,555	N/A	N/A	N/A 1,449	
miR-24-3p (and other miRNAs w/seed GGCUCAG)	N/A	-1,806	-1,236	2,449	N/A	N/A	N/A
TWIST2	N/A	-0,466	0,053	N/A	N/A	N/A	N/A
metformin	-0,218	1,169	-2,21	0,418	N/A	N/A	N/A
silibinin	N/A	1,531	-2	N/A	N/A	N/A	N/A
salirasib	N/A	-0,849	0,377	N/A	N/A	N/A	N/A
butyric acid	-1,878	-0,303	-0,175	N/A	0,848	-0,73	N/A
mir-8	-0,07	-2,143	-0,686	2,105	N/A	-2,268	N/A
mir-29	0,887	0	-0,508	N/A	N/A	-0,588	N/A
mir-133	N/A	1,463	-1,982	N/A	N/A	-1,73	N/A
amino acids	N/A	-0,971	0,447	N/A	N/A	N/A	N/A
SETD2	N/A	-0,644	0,105	N/A	N/A	-1,342	N/A
CDKN1A	0,152	-0,669	0,128	N/A	N/A	-1,4	0,074
TP53	-1,214	0,168	-0,711	N/A	2,06	-2,102	0,829
CDK19	N/A	-0,39	-0,179	N/A	N/A	-0,447	-1,342
HOXB13	N/A	0	-0,585	N/A	N/A	N/A	N/A
Immunoglobulin	-0,058	-0,077	-0,508	N/A	1,915	-1,139	-1,246
setanaxib	N/A	0,147	-0,736	N/A	N/A	N/A	N/A
ELOVL5	N/A	-0,453	-1,444	1	N/A	N/A	N/A
MXI1	N/A	0,224	-0,831	N/A	N/A	N/A	N/A
fluoxetine	N/A	0,18	-0,788	N/A	N/A	N/A	N/A
sterol	N/A	-0,761	0,132	N/A	N/A	N/A	N/A
SIRT1	-1,118	-0,433	-0,25	N/A	N/A	0	-0,647
mir-7	N/A	-1,266	-1,746	1,98	N/A	N/A	N/A
SAHM1	N/A	0,072	-0,766	N/A	N/A	N/A	N/A
FAS	N/A	-1,343	0,647	N/A	N/A	N/A	N/A
lenalidomide	N/A	-0,707	0	N/A	N/A	N/A	-1,982
COL5A1	N/A	-0,707	0	N/A	N/A	N/A	N/A
AICAR	N/A	-0,711	0	N/A	N/A	N/A	N/A
SPDEF	N/A	-0,118	-0,6	N/A	N/A	N/A	N/A
DNAJB6	1,387	0,577	-1,664	0	N/A	N/A	N/A
NDUFA13	N/A	0,087	-0,853	N/A	N/A	N/A	N/A
FOXO1	N/A	-1,604	0,832	N/A	N/A	N/A	0,032

androgen	N/A	-1,445	0,623	N/A	N/A	N/A	N /A
MDM2	N/A	-0,043	-0,78	N/A	N/A	N/A	N /A
CLPP	N/A	1,134	N/A	-2	N/A	-2	N/A
tetrachlorodibenzodioxin	N/A	0,24	-1,109	N/A	N/A	-0,156	-0,944
miR-483-3p (miRNAs w/seed CACUCCU)	N/A	-1,229	0,343	N/A	N/A	N/A	N/A
BRCA1	N/A	-0,629	-0,271	N/A	N/A	N/A	N/A
SUB1	N/A	-1,332	0,404	N/A	N/A	N/A	N/A
steroid	N/A	-0,316	-0,632	N/A	N/A	N/A	N/A
ochratoxin A	N/A	-1,022	-1,281	0,849	N/A	N/A	N/A
trichostatin A	-0,493	0,128	-1,103	N/A	-1,762	1,438	0,206
alvocidib	N/A	-0,788	-0,2	N/A	N/A	N/A	N/A
YWHAZ	N/A	-1,864	-1,554	1,929	N/A	N/A	N/A
RARB	N/A	-0,938	-0,069	N/A	N/A	0	N/A
mitomycin C	N/A	-1,375	0,347	N/A	N/A	1,387	N/A
let-7a-5p (and other miRNAs w/seed GAGGUAG)	1,386	-2,437	1,99	-1,116	N/A	-1,347	-1,262
SPTLC2	N/A	-0,51	-0,555	N/A	N/A	N/A	N/A
entinostat	N/A	-0,972	-0,102	N/A	N/A	N/A	N/A
SB-431542	1,091	0,302	-1,408	N/A	N/A	N/A	N/A
PTPN6	N/A	-0,258	-0,853	N/A	N/A	N/A	N/A
MYCN	1,271	-1,677	1,994	-1,992	6,234	-2,782	-5,899
UBA1	1	0,335	-1,393	-0,655	0,447	N/A	N/A
HES3	N/A	-1,02	-0,127	N/A	N/A	N/A	N/A
SH3KBP1	N/A	-0,711	-0,459	N/A	N/A	N/A	N/A
verapamil	N/A	-1,414	-1,342	1	N/A	N/A	N/A
glutamine	N/A	-2,53	2,646	-2	N/A	-0,068	N/A
phenylbutazone	0,382	-1,375	0,07	N/A	N/A	1,782	N/A
IND S1	N/A	-1,964	0,478	N/A	N/A	-1,342	N/A
MAZ	N/A	-1,567	-0,552	-0,14	N/A	N/A	N/A
BCL2	N/A	-0,711	-0,816	N/A	N/A	N/A	N/A
IFT88	N/A	-0,13	-1,4	N/A	N/A	N/A	N/A
TFRC	N/A	-2,673	-1,633	2	N/A	N/A	N/A
sorafenib	N/A	-1,626	0,085	N/A	N/A	N/A	N/A
mannose	N/A	-0,368	-1,183	N/A	N/A	N/A	N/A
Collagen type IV	N/A	-1,129	-0,438	N/A	N/A	N/A	N/A
CDKN2A	N/A	-0,769	-0,816	N/A	-0,707	N/A 1,412	
baicalein	N/A	-1,394	-0,218	N/A	N/A	N/A	N/A
captopril	0,691	-0,555	-1,067	N/A	N/A	1,091	N/A
PRKACA	N/A	-1,751	0,128	N/A	N/A	N/A	N/A
mir-122	N/A	-0,111	-2,556	0,229	N/A	-2,449	N/A
guanidinopropionic acid	N/A	0,44	-0,898	-1,982	2,635	N/A -2	
SFRP1	N/A	-1,703	0,046	N/A	N/A	N/A	N/A
KLF3	-0,378	-2,191	1,667	-2	N/A	-1,667	N/A
SIRT3	-2,211	-0,905	-0,64	-1,054	2,307	3,153	N/A
DCAF1	N/A	-1,291	-0,447	N/A	N/A	N/A	N/A
TNFRSF8	N/A	0,293	-2,032	N/A	N/A	N/A	N/A
DDX5	N/A	-2,642	2,213	-2,236	3,162	1,134	-2,551
MST1	N/A	-1,376	-0,44	N/A	N/A	N/A	N/A
MYCL	N/A	-0,816	-1	N/A	3,703	-1,172	N/A
CRBN	N/A	-0,483	-1,353	N/A	N/A	N/A	N/A
diclofenac	N/A	-1,078	-0,763	N/A	N/A	N/A	N/A
PNN	N/A	-1,414	-0,447	N/A	N/A	N/A	N/A
ZBTB7A	N/A	-3,212	-1,091	1,476	N/A	N/A	N/A
hexamethylene bisacetamide	N/A	-1,627	-0,277	N/A	N/A	N/A	N/A
PPM1D	N/A	-1,091	-0,849	N/A	N/A	N/A	N/A
NFE2L1	N/A	-1,501	-0,469	N/A	2	N/A	N/A
miR-133a-3p (and other miRNAs w/seed UUGGUCC)	N/A	-1	-1	N/A	N/A	N/A	N/A
AIFM1	N/A	-0,968	-1,067	N/A	N/A	N/A	N/A
ACOX1	N/A	-2,997	-2,37	2,261	N/A	N/A	N/A
mifepristone	-0,796	-0,849	-1,229	N/A	N/A	-0,137	N/A
trastuzumab	N/A	-1,291	-0,799	N/A	N/A	N/A	N/A
actinomycin D	-0,114	-0,378	-1,725	N/A	N/A	-1,463	N/A
INSIG2	N/A	-1,498	-0,606	N/A	N/A	N/A	N/A
ethionine	0,632	-2,132	0	N/A	N/A	1,633	N/A
PP1	N/A	0,421	-2,577	N/A	N/A	N/A	N/A
xanthohumol	N/A	-2,724	N/A	0,555	N/A	N/A	N/A
DICER1	-1,982	-0,832	-1,342	N/A	N/A	N/A	N/A
MIR124	N/A	-1,912	-0,303	N/A	N/A	N/A -0,152	
HSP90B1	N/A	-1,828	-0,478	N/A	1,897	-0,632	N/A
Smad	N/A	-1,18	-1,127	N/A	N/A	N/A	N/A
SPI1	N/A	-0,329	-1,98	N/A	N/A	N/A	N/A
SUPT16H	N/A	-3,568	1,25	N/A	-1	N/A	N/A
ACLY	N/A	-2,253	-0,083	N/A	N/A	N/A	N/A
raltitrexed	N/A	-3,053	0,707	N/A	N/A	N/A	N/A
forskolin	2,421	-1,331	-1,026	N/A	1,943	1,617	1,095
sapanisertib	N/A	-1,778	-0,64	N/A	N/A	N/A	N/A
DAB2IP	N/A	-1,911	-0,6	N/A	N/A	N/A	N/A
sodium arsenite	N/A	-0,957	-1,604	N/A	N/A	1,969	N/A
IREB2	N/A	-1,868	-0,721	N/A	N/A	N/A	N/A
garcinol	N/A	-1,338	-1,32	N/A	N/A	N/A	N/A
TSC1	0	-1,024	-1,681	N/A	N/A	N/A	N/A
tyrphostin AG 1478	N/A	-3,965	-3	2,858	N/A	N/A	N/A
E. coli B5 lipopolysaccharide	-0,152	-5	-3,413	4,243	N/A	N/A	N/A
MAPK11	N/A	-1,987	-0,801	N/A	N/A	0,882	N/A
miR-26a-5p (and other miRNAs w/seed UCAAGUA)	N/A	-1,475	-1,363	N/A	N/A	N/A	N/A
CST5	-0,302	-0,896	-1,982	N/A	0,632	-1,649	1,134
sulforafan	N/A	-2,203	-0,719	N/A	N/A	1,238	N/A
propylthiouracil	0	-1,941	-1	N/A	1,667	-2,449	N/A
HDAC5	N/A	-2,368	-0,644	N/A	N/A	-1,131	N/A
CUL4B	N/A	-1,912	-1,117	N/A	N/A	N/A	N/A
DKK1	N/A	-1,257	-1,89	N/A	N/A	N/A	N/A
GDF2	N/A	-2,335	-0,928	N/A	N/A	N/A	N/A
COL18A1	N/A	-1,542	-1,732	N/A	N/A	N/A	N/A
Rb	N/A	-2,514	-0,816	N/A	N/A	N/A	N/A
mir-30	N/A	-1,995	-1,347	N/A	N/A	N/A	N/A
HTATIP2	N/A	-2,563	-0,818	N/A	N/A	N/A	N/A
docosahexaenoic acid	N/A	-1,155	-2,236	N/A	N/A	N/A	N/A
SET	N/A	-2,458	-1,12	N/A	N/A	N/A	N/A
topotecan	N/A	-2,33	-1,478	N/A	N/A	-0,816	1
methylselenic acid	N/A	-1,45	-2,433	N/A	N/A	N/A	N/A
miR-27a-3p (and other miRNAs w/seed UCACAGU)	N/A	-1,553	-2,401	N/A	N/A	N/A	N/A
ADCYAP1	1,414	-2,138	-2	N/A	0,378	1,58	1,342
BCAP31	N/A	-2,283	-1,883	N/A	N/A	N/A	N/A
EWSR1-FLI1	1,566	-2,219	-1,98	N/A	N/A	N/A	N/A
U73122	N/A	-2,085	-2,169	N/A	N/A	N/A	N/A
aldesleukin	N/A	-2,728	-1,539	N/A	N/A	2,236	N/A
GABA	-1,134	-5,196	0,927	N/A	-2,744	-1,347	3,545
FMR1	1,131	-1,677	-2,646	N/A	1,744	-1,369	N/A
GJA1	N/A	-3,013	-1,706	N/A	N/A	N/A	N/A
PAX5	N/A	-2,643	-2,12	N/A	N/A	N/A	N/A
TSC2	-0,623	-3,606	-1,633	N/A	-0,243	-0,277	0,277
BMP10	N/A	-3,676	-1,953	N/A	N/A	N/A	N/A

Table S3 IPA upstream analysis of treated vs. untreated in all samples

Upstream Regulators	all_benign	BIHi040	BIHi246	BIHi046	all_progessive	BIHi039	BIHi047	BIH237-A
lipopolysaccharide	4,768	4,434	4,543	5,202	4,856	6,746	4,868	5,218
TNF	3,903	3,343	3,774	4,39	4,233	5,365	3,737	4,924
IFNG	2,966	3,256	3,74	4,197	4,131	6,076	3,711	4,491
tetradecanoylphorbol acetate	3,574	2,867	2,309	3,894	3,004	3,626	3,842	3,424
IL1B	2,506	3,657	2,884	3,541	3,913	4,093	2,286	3,646
poly rI:rC-RNA	2,812	1,767	2,363	3,801	3,647	4,731	3,352	3,531
IL1A	2,368	2,156	2,556	3,338	3,476	3,212	3,045	3,618
IFNA2	1,931	2,638	2,622	2,907	3,108	4,747	2,396	3,262
RELA	2,735	2,546	2,404	3,113	3,12	3,344	2,753	3,038
ETV6-RUNX1	-3,293	-1,287	-2,224	-2,724	-3,57	-3,429	-2,305	-3,206
progesterone	2,891	2,585	2,713	2,891	2,514	2,878	3,059	2,191
STAT1	1,837	2,363	2,784	2,494	2,541	4,022	2,045	2,526
Interferon alpha	1,476	1,897	1,594	2,733	3,136	4,197	2,103	3,08
filgrastim	-2,646	-1,878	-2,779	-2,867	-2,615	-2,489	-2,032	-2,722
TLR4	2,243	2,418	1,844	2,593	2,423	3,08	2,915	2,305
pirinixic acid	1,705	2,217	2,033	2,479	2,479	3,085	3,652	1,97
NFkB (complex)	2,151	2,389	2,295	2,178	2,172	3,222	2,11	3,076
SP1	2,067	2,593	2,39	2,76	1,807	3,237	2,054	2,498
valproic acid	-1,37	-0,115	-2,101	-3,427	-2,894	-3,296	-2,9	-2,979
IKZF1	-2,594	-1,718	-2,412	-2,402	-2,778	-2,956	-2,598	-1,605
BHLHE40	2,333	1,664	2,121	2,714	2,887	2,673	2,53	2,121
IL15	2,608	1,131	2,607	2,935	2,575	1,977	3,123	1,96
TLR7	1,943	1,949	2,184	2,375	2,397	3,241	1,982	2,742
F2	2,274	2,768	2,401	2,689	1,627	2,413	2,655	1,892
EIF4E	2,159	2,236	2,159	2,165	2,373	2,747	2,566	2,159
IRF7	1	1,964	2,177	2,033	2,889	3,893	1,461	2,889
N-acetyl-L-cysteine	-2,607	-2,167	-2,418	-2,424	-1,402	-2,215	-2,613	-2,418
camptothecin	1,633	2,187	2	2,655	1,897	2,828	2,832	1,806
Ige	2,236	2,449	2,236	2,236	1,912	2,236	2,828	1,633
Ifnar	1,432	N/A	1,98	2,75	2,563	3,393	2,578	2,8
TGM2	1,915	N/A	2,2	2,414	2,611	3,576	2,414	2,343
SB203580	-2,34	-1,815	-1,552	-3,234	-1,107	-3,452	-2,233	-1,512
CITED2	-1,982	N/A	-1,982	-2,784	-2,2	-3,102	-2,407	-2,784
NKX2-3	-2,449	-0,819	N/A	-2,828	-3,162	-3,317	-2,828	-1,769
TP53	2,407	3,083	2,571	1,836	1,619	2,156	1,726	1,729
IRF3	1,573	N/A	2,136	2,431	2,748	3,36	1,925	2,748
CD 437	-2,331	-0,181	-3,148	-0,824	-2,853	-2,985	-2,374	-2,197
sirolimus	-2,321	-2,56	-2,087	0,173	-1,896	-3,438	-2,404	-1,684
resiquimod	2,401	N/A	2,401	2,764	2,16	2,396	2,16	1,947
epigallocatechin-gallate	-2,38	-1,265	N/A	-2,517	-2,577	-2,198	-2,517	-2,658
actinomycin D	-2,236	0,468	-2,433	-1,912	-2,804	-2,433	-2,138	-1,636
MYC	-1,919	-0,856	-2,73	-3,576	-2,002	-1,839	-0,983	-1,951
IL6	1,899	1,845	1,573	2,018	2,27	3,343	1,198	1,661
EP300	1,97	2,175	1,744	2,18	1,356	1,97	2,18	2,183
S100A8	2,219	N/A	1,982	2,433	2,219	2,219	2,219	2,433
CASR	2,236	1	2	2,433	2,449	2	1,912	1,633
Ifn gamma	1,982	N/A	1,982	2,2	2,2	2,607	1,982	2,599
OSM	1,304	1,124	0,619	3,008	1,713	3,139	2,272	2,254
RC3H1	-1,342	N/A	-2	-2,331	-2,138	-3,606	-1,706	-2,138
IFNB1	1,675	N/A	1,603	2,053	2,02	3,126	2,16	2,507
LMO2	2,236	0	1,633	2,236	2,646	2	2,449	1,89
LDB1	2,236	0	1,633	2,236	2,646	2	2,449	1,89
IRF1	0,908	0,305	1,562	2,196	2,359	3,127	1,821	2,639
Ifn	1,484	N/A	1,931	1,992	2,368	2,918	1,992	2,171
IL27	1,981	N/A	N/A	2,789	2,42	2,947	2,26	2,42
U0126	-1,591	-2,353	-0,946	-2,12	-1,545	-2,613	-1,847	-1,674
VDR	2,228	1,941	1,992	1,863	2,317	1,3	1,863	1,087
dexamethasone	-1,718	-1,411	-1,694	-2,242	-2,054	-2,168	-1,276	-1,992
FN1	2,345	0,841	1,855	2,089	1,274	1,876	1,5	2,664
CD40LG	1,685	0,097	1,369	2,727	2,356	1,905	2,736	1,443
doxorubicin	1,406	2,464	1,673	1,941	1,839	1,698	2,224	1,028
homocysteine	1,932	1,955	0,762	2,394	1,113	2,174	1,803	1,866
FOXM1	2,374	0,886	1,927	1,749	2,202	2,154	1,498	1,127
PLAU	2,213	0,94	1,982	2,219	1,297	1,131	1,518	2,433
IFN Beta	1,293	1,067	0,907	2,28	1,354	2,634	1,914	2,242
CD28	2,213	N/A	1,067	2,345	2,345	0,896	2,63	2,157
TNFSF11	1,708	1,445	1,394	1,263	2,289	2,112	1,86	1,574
E. coli B4 lipopolysaccharide	1,633	N/A	2	1,342	2,449	2,611	1,48	2
E. coli B5 lipopolysaccharide	1,022	1,709	1,694	1,967	2,002	2,107	1,661	1,301
IL1	1,725	0,555	1,485	2,171	1,987	1,485	1,987	2,019
ST1926	-2,138	0,092	-2,433	-0,316	-2,345	-2,219	-2,35	-1,463
JUN	1,88	1,167	1,315	1,744	2,457	1,906	1,18	1,636
quercetin	-1,98	N/A	-2,213	-1,698	-1,937	-1,432	-2,621	-1,387
SP110	-1,342	-2	N/A	-1,897	-1,89	-3,317	-1,134	-1,633
SMARCA4	1,405	1,311	0,447	1,625	2,393	2,571	1,554	1,824
C5	2,177	N/A	1,44	1,948	1,315	2,177	2,387	1,675
PRL	0,623	0,942	2,177	1,552	1,375	3,104	1,522	1,701
CD3	1,725	1,109	0,156	2,111	1,937	1,508	2,525	1,866
hydrogen peroxide	1,969	3,17	1,837	0,647	1,878	1,408	1,693	0,279
IFNL1	0,896	N/A	N/A	2,011	2,393	3,93	1,234	2,203
ELAVL1	1,067	2,168	N/A	1,698	1,664	2,36	1,937	1,706
AGT	1,776	2,499	0,994	1,524	1,324	1,76	1,313	1,398
CREBBP	1,982	N/A	N/A	2,19	2,19	1,982	1,96	2,19
trovafloxacin	1	2	N/A	1,89	1,633	2,236	1,414	2,236
NFKB1	0,862	2,19	0,862	1,562	1,562	1,818	1,221	2,278
MAPK1	-0,544	-0,235	-1,567	-1,709	-1,99	-3,315	-1,408	-1,474
JAK	1,342	N/A	2	1,633	1,408	2,646	0,956	2,236
CTNNB1	1,777	2,222	1,2	2,159	1,043	1,913	0,952	0,954
PRKCA	1,673	-0,459	1,408	1,406	1,949	1,406	1,673	2,234
STAT6	-1,274	N/A	N/A	-2,025	-2,592	-2,204	-1,816	-2,22
TNFSF10	1,066	0	1,664	1,951	1,32	3,212	1,839	1,066
PIK3R1	1,96	N/A	1,082	2,19	1,342	2,2	1,96	1,342
TGFB1	2,741	4,11	0,699	0,883	-0,337	1,467	1,288	-0,55
arsenic trioxide	1,404	1,217	1,196	2,227	0,911	1,543	1,901	1,675
KAT2B	1,964	N/A	1,091	1,964	1,4	1,964	1,964	1,72
LY294002	-1,429	-3,196	-1,221	-0,945	-0,533	-1,577	-1,429	-1,697
AHR	0,447	-0,085	1,342	1,937	2,194	1,964	1,66	2,387
medroxyprogesterone acetate	1,696	1,846	0,874	1,656	1,151	1,967	1,734	1,059
PI3K (family)	1,972	N/A	1,513	2,195	1,009	1,513	1,972	1,755
wortmannin	-1,942	-1,592	-1,452	-1,342	-1,287	-1,103	-1,534	-1,648
CSF2	0,581	1,083	1,941	0,817	1,404	2,156	1,853	1,934
PDGF BB	-0,229	2,588	1,408	0,955	1,263	1,715	0,669	2,784
MUC1	1,07	N/A	1,983	1,18	1,646	2,414	1,336	1,913
etoposide	0,818	1,79	1,412	1,812	1,516	1,154	1,224	1,788
DDX58	N/A	N/A	N/A	2,383	2,397	2,372	2,178	2,178
Z-LLL-CHO	1,783	2,167	1,26	1,897	0,23	0,836	2,077	1,253
ATG5	2	N/A	N/A	0,816	2,433	2	2	2,236
deferoxamine	1,026	2,769	1,026	1,026	1,294	1,709	1,539	1,091
MED1	2,2	0,928	1,964	2,2	1,964	N/A	2,2	N/A
mifepristone	-1,491	-0,438	-1,536	-1,491	-1,457	-2,423	-1,269	-1,215
EGF	1,023	1,167	1,23	1,562	1,19	1,193	2,227	1,702
miR-124-3p (and other miRNAs w/seed AAGGCAC)	-1,622	-2,413	-1,987	-0,742	-1,073	-1,987	-1,115	-0,354
CXCL12	1,759	N/A	1,491	1,788	0,902	1,999	1,551	1,759
tazemetostat	-1,248	1	-1,227	-1,729	-0,938	-1,119	-1,566	-2,366
aldesleukin	N/A	N/A	N/A	2,138	2,138	2,828	2,138	1,89

trichostatin A	-1,373	1,375	-1,213	-1,292	-1,814	-0,328	-1,879	-1,853
JAK1	N/A	N/A	N/A	2,415	2,207	2,63	2,415	1,446
Nfat (family)	1,964	N/A	1,964	0,686	1,4	1,964	1,964	1,091
CSF1	1,963	1,521	0,642	1,431	1,307	1,61	1,107	1,368
TLR3	N/A	N/A	1,176	1,977	1,977	1,824	1,757	2,208
budesonide	1,633	0,152	N/A	1,89	2,646	N/A	1,89	2,63
NONO	1,192	N/A	N/A	1,45	1,818	3,429	0,883	2,06
EDN1	2,216	2,39	N/A	1,644	0,559	1,086	1,26	1,644
tretinoin	0,947	1,246	0,78	1,186	1,515	2,594	1,312	1,214
IL2	1,632	N/A	0,87	2,38	1,711	0,87	1,559	1,748
TICAM1	N/A	N/A	N/A	2,133	2,133	2,215	2,133	2,133
cigarette smoke	1,538	2,154	1,21	1,712	0,633	0,792	1,512	1,179
PRKCD	1,492	1,941	1,423	0,35	1,492	1,423	1,118	1,449
decitabine	1,306	3,589	0,864	0,839	-0,337	2,565	0,263	-0,918
PF4	1,225	N/A	1,225	1,543	1,816	1,543	1,543	1,78
FOS	1,706	1,773	0,832	1,339	0,678	1,941	0,785	1,609
Tgf beta	1,452	1,913	2	1,498	0,609	1,183	0,275	1,699
Insulin	1,684	2,328	1,188	0,948	0,681	1,544	1,638	0,614
EIF2AK2	0,686	1,123	1,095	1,109	1,474	2,474	0,951	1,697
EBI3/IL-27B	N/A	N/A	N/A	2,219	1,982	2,186	2,219	1,982
concanavalin a	N/A	1,96	N/A	1,974	2,2	N/A	2,2	2,19
NFE2L2	1,044	1,665	1,546	0,719	0,534	2,048	2,547	0,417
JQ1	-1,446	N/A	-1,446	-1,4	-1,706	-1,091	-1,671	-1,741
STAT4	1,561	N/A	1,253	1,489	1,489	2,207	1,144	1,317
ribavirin	N/A	N/A	N/A	2,138	1,912	2,813	1,664	1,912
fenofibrate	1,698	N/A	1,698	1,698	1,937	0,555	1,937	0,896
IRGM	-1	N/A	N/A	-1,667	-1,89	-3,148	-0,816	-1,89
8-bromo-cAMP	1,046	2,183	0,218	1,947	-0,095	2,177	1,947	0,794
cytokine	0,536	N/A	1,36	1,887	1,36	1,945	1,638	1,638
AICAR	1	1,772	1	1,633	1,195	1	1,633	1,095
NFATC2	2	N/A	N/A	1,633	1,408	2,236	1,408	1,633
tamoxifen	0,308	0,346	1,63	1,981	1,022	1,877	1,105	1,99
lactacystin	2,037	2,062	2	0,761	0,338	0,421	1,186	1,408
Irgm1	-0,555	N/A	N/A	-1,706	-1,706	-2,753	-1,276	-2,2
Salmonella enterica serotype abortus equi lipopolysaccharide	N/A	N/A	N/A	1,953	1,941	1,941	2,177	2,183
SOCS1	N/A	N/A	N/A	-2,218	-1,762	-2,592	-1,553	-2,026
CLDN7	-1,664	N/A	N/A	-2	-1,294	-2,236	-2	-0,923
SP2509	-1,248	1	-1,227	-1,729	-0,938	N/A	-1,566	-2,366
TNFSF12	N/A	N/A	1,96	N/A	1,96	1,96	2,2	1,96
ZFTA-RELA	N/A	N/A	N/A	2	2	2	2	2
IL21	N/A	N/A	N/A	2	2	2	2	2
NOS2	1,432	1,481	0,655	1,095	1,628	1,3	0,784	1,609
triamcinolone acetonide	1	N/A	2	2	1,134	2,219	0,447	1,134
NGF	1,959	1,605	N/A	1,118	1,532	N/A	2,181	1,53
tofacitinib	N/A	N/A	N/A	-1,982	-1,982	-1,982	-1,982	-1,982
RNASEH2B	N/A	N/A	N/A	-1,664	-1,982	-2,433	-1,387	-2,433
IFNA1/IFNA13	N/A	N/A	N/A	1,812	1,873	2,751	1,59	1,873
1,2-dithiol-3-thione	1,418	N/A	1,018	1,388	0,94	1,686	2,936	0,493
forskolin	0,645	1,644	-0,218	1,841	0,469	2,2	0,801	2,035
HAVCR1	1,134	0,447	1,342	1,667	1,89	0,822	1,414	1,134
5-fluorouracil	-1,348	1,277	-1,347	0,35	-1,883	-1,661	-1,429	-0,544
KLF6	N/A	1,046	N/A	1,961	1,199	1,97	1,474	2,16
SPP1	1,432	N/A	N/A	1,458	1,452	2,213	1,067	2,173
CG	0,728	0,282	1,132	1,483	1,322	1,48	2,021	1,328
CD40	N/A	N/A	N/A	2,212	1,892	1,93	1,821	1,899
AGN194204	1	2,39	N/A	1,342	1,89	N/A	1,414	1,673
MTOR	1,673	1,544	1,941	0,853	0,777	1,677	0,777	0,431
ZBTB10	0,447	N/A	N/A	1,195	2,219	2,379	1,195	2,219
FOXC1	0,816	N/A	N/A	1,941	1,265	3,051	1,265	1,265
ciprofibrate	1	1,213	0,655	1,213	1,528	0,832	1,823	1,213
2-amino-1-methyl-6-phenylimidazo-4-5-b-pyridine	N/A	N/A	0,555	2,63	1,912	N/A	3,302	1,067
diethylstilbestrol	2,144	2,091	N/A	N/A	1,987	N/A	1,363	1,852
CCN1	1,966	N/A	N/A	1,987	1,987	1,235	N/A	2,213
estrogen	0,761	1,011	-0,15	1,353	1,721	1,727	1,353	1,233
LDL	1,469	1,467	0,464	1,197	0,464	2,175	1,197	0,842
IRF4	-0,447	-1	-1	-0,971	-1,706	-1,432	-1,32	-1,387
JAK2	N/A	N/A	N/A	2,418	2,215	N/A	2,418	2,201
tyrphostin AG490	-0,747	N/A	N/A	-0,766	-1,839	-2	-1,969	-1,854
nitric oxide	1	2,382	1	1	0,737	1	0,577	1,411
REL	N/A	N/A	N/A	2,176	1,72	2,176	1,471	1,556
IKBKB	0,414	-0,626	1,336	1,416	0,594	1,87	1,775	0,973
IL22	1,165	N/A	1,206	1,165	1,761	1,452	0,714	1,524
FSH	0,692	0,615	1,154	1,969	1,44	N/A	1,351	1,706
OGA	0	-1,129	0,447	1,026	1,732	2,121	0,508	1,959
RPTOR	2	1,982	N/A	1,342	N/A	N/A	2,219	1,342
SENP3	N/A	N/A	N/A	2,219	2	2,433	2,219	N/A
PD98059	-1,405	-0,616	0,477	-0,952	-0,616	-1,126	-1,405	-2,274
CHUK	0,767	0,302	1,159	1,288	0,855	2,144	0,872	1,467
CREB1	0,956	1,664	-0,478	1,556	0,508	1,947	0,954	0,777
MYD88	N/A	N/A	1,099	1,722	1,377	1,722	2,207	0,685
TRIM24	N/A	N/A	N/A	-1,915	-1,671	-2,39	-1,4	-1,4
AR	-0,481	0,555	0,083	-1,121	-1,314	0,692	-1,905	-2,596
THPO	1,154	N/A	1,154	1	1,446	1,491	1	1,446
NRG1	1,356	1,71	N/A	1,363	1,327	N/A	1,034	1,877
JAK1/2	N/A	N/A	N/A	2,219	2,219	N/A	2,219	1,982
troglitazone	-0,952	1,854	-0,73	0,046	-1,509	-1,279	0,128	-2,132
PAF1	N/A	N/A	N/A	1,342	2	2,236	1	2
ESR2	0,894	1,945	0,092	0,834	1,955	-0,698	1,39	0,75
NFKBIA	1,675	0,501	1,094	1,412	0,75	0,888	1,014	1,212
RICTOR	-0,798	N/A	-1,109	0,369	-2,111	-1,726	-1,192	-1,238
bromodeoxyuridine	N/A	N/A	N/A	1,673	1,406	2,598	1,406	1,406
stallimycin	N/A	N/A	N/A	1,673	1,406	2,598	1,406	1,406
IFNAR2	N/A	N/A	N/A	2,236	2,236	2	N/A	2
isotretinoin	N/A	N/A	N/A	2	2	2,219	N/A	2,236
estrogen receptor	-1,36	-1,342	-1,408	-1,539	-0,563	-0,64	-0,956	-0,647
CpG ODN 2006	N/A	N/A	N/A	2,132	1,886	2,549	N/A	1,886
resveratrol	-1,475	-0,152	-0,816	-1,845	-0,484	-1,264	-1,588	-0,804
NLRC5	N/A	N/A	N/A	2,216	1,983	1,981	2,216	N/A
Tnf (family)	1,165	N/A	1,165	N/A	1,452	1,709	1,452	1,452
ERBB2	1,184	1,908	0,967	0,497	0,556	0,991	-0,528	1,691
IL3	1,98	2,183	N/A	0,577	0,129	1,715	1,732	N/A
NCOA2	1,165	N/A	1,165	1,287	1,016	1,452	0,912	1,287
SP600125	-1,945	-1,019	-0,396	-1,177	-1,067	-0,939	-1,469	-0,265
acetaminophen	1,706	N/A	1	1,066	1,063	N/A	1,912	1,463
PAX3-FOXO1	-0,707	-1,134	-0,447	-2,343	-1,718	N/A	-0,632	-1,219
IL5	N/A	2	0,896	0,647	0	2,433	1,492	-0,647
salmonella minnesota R595 lipopolysaccharides	N/A	N/A	N/A	1,965	1,965	N/A	2,183	1,965
DICER1	-1,715	-1,604	N/A	N/A	-1,715	-1,067	N/A	-1,969
P38 MAPK	0,684	2,216	0,201	1,223	0,252	1,935	N/A	1,559
sphingosine-1-phosphate	2,183	N/A	N/A	1,953	1,709	N/A	2,219	N/A
NR3C1	-1,543	-0,267	-1,177	-1,144	-0,369	-1,144	-1,029	-1,374
prostaglandin J2	N/A	N/A	N/A	1,698	1,937	1,067	2,157	1,154
TLR9	N/A	N/A	N/A	1,823	1,823	2,474	N/A	1,823
curcumin	-0,794	0,53	-0,359	-1,337	-1,278	-1,335	-1,036	-1,226
peptidoglycan	N/A	N/A	N/A	1,969	1,969	N/A	1,969	1,969
GnRH analog	N/A	0,896	N/A	-1,155	-0,707	-2,449	-1,508	-1,134

SNCA	N/A	N/A	N/A	2,213	2,213	N/A	1,982	1,432
G protein alphai	N/A	N/A	N/A	1,941	1,982	1,969	1,941	N/A
miR-34a-5p (and other miRNAs w/seed GGCAGUG)	-1,078	-1,993	N/A	-0,64	-1,109	-1,408	-0,277	-1,303
CCND1	N/A	2,176	N/A	0,816	2	N/A	-1,134	1,673
SYVN1	1,342	N/A	N/A	1,134	0,378	2,236	1,89	0,816
testosterone	0,708	2,658	2,245	-0,042	0,033	0,95	0,845	0,302
IL1RN	N/A	-0,152	0	-1,709	-2,012	-1,709	-1,172	-1,026
nitrofurantoin	0,798	2	1,342	0,087	1,121	-0,152	1,601	0,659
CST5	-1,066	-2,121	-1	-0,632	-0,577	1	-1,291	0
metformin	0,816	1,457	1,242	1,134	1,364	0,378	0,707	0,573
mir-21	-1,066	-2,132	0,283	-0,485	-1,533	-0,558	-0,718	-0,724
MYCN	-0,8	-0,465	-0,585	-2,131	-0,163	0,621	-1,594	1,067
methapyrilene	N/A	N/A	N/A	2	1,89	N/A	1,89	1,633
corticosterone	N/A	N/A	-1,941	-2	-2,368	N/A	N/A	-1,082
Jnk	1,928	N/A	1,229	1,483	0,625	N/A	1,22	0,892
BCL2	-1,452	-1,966	N/A	-0,665	-1,016	N/A	-1,474	-0,794
5-O-mycolyl-beta-araf-(1->2)-5-O-mycolyl-alpha-araf-(1->1')-glyc	N/A	N/A	N/A	1,342	2	2	N/A	2
PITX2	1,117	N/A	N/A	0,264	1,411	1,117	1,929	1,488
beta-estradiol	0,233	2,161	-0,455	-0,734	-1,141	1,33	0,768	-0,476
prednisolone	N/A	N/A	N/A	-0,816	N/A	-2,236	-2,236	-2
molybdenum disulfide	-0,707	-1,342	N/A	1,342	1,633	N/A	N/A	2,236
bucladesine	0,338	N/A	N/A	1,991	1,355	N/A	1,759	1,794
FGF2	0,204	0,833	-0,391	1,339	1,331	-0,174	1,333	1,625
PML	0,365	1,523	-0,73	1,322	0,998	1,504	-0,296	0,458
STK11	N/A	2,219	-1	-1,667	-1	N/A	0,333	-0,956
PGR	0,928	1,711	0,928	0,404	1,004	0,718	0,388	1,063
CEBPB	-0,478	0,932	0,64	-0,27	0,79	1,873	1,895	0,262
fulvestrant	0,713	0,895	0,713	1,262	1,316	0,713	0,397	1,093
SPI1	0,497	N/A	0,343	1,107	1,281	2,355	-0,099	1,411
PP2/AG1879 tyrosine kinase inhibitor	N/A	N/A	N/A	-2,2	-1,342	N/A	-1,342	-2,2
dihydrotestosterone	1,026	2,359	0,772	-0,351	-0,184	1,484	0,237	-0,654
cyclophosphamide	-2,144	N/A	N/A	-0,967	-0,808	-1,131	-0,673	-1,314
PNPT1	N/A	N/A	N/A	N/A	-2	-2,795	N/A	-2,236
GFI1	N/A	N/A	N/A	-1,951	N/A	-0,849	-2,186	-2
cyclosporin A	-1,609	0,221	-1,54	0,093	-0,951	-1,072	-0,427	-1,05
iron	0,974	N/A	1,989	0,974	0,974	N/A	0,974	1,06
daidzein	N/A	N/A	N/A	-1,664	-2,219	-1,387	-1,664	N/A
IFN alpha/beta	N/A	N/A	N/A	2,236	N/A	2,4	2,236	N/A
D-glucose	0,488	-0,101	-0,05	2,318	1,466	0,578	1,364	0,478
rosiglitazone	-0,439	N/A	-0,568	-1,253	-0,81	0,117	-1,483	-2,157
oblimersen	N/A	N/A	N/A	1,633	0,447	2,828	1	0,896
tanespimycin	-0,6	0,528	-1,091	-0,552	-0,149	-1,4	-0,877	-1,604
miR-182-5p (and other miRNAs w/seed UUGGCAA)	N/A	N/A	N/A	-1,633	-0,816	-2,236	-0,447	-1,633
IgG	0,123	-2,236	-1,342	0,38	-0,231	-2,135	0,017	-0,277
diclofenac	0,555	1,154	0,391	0,447	0,896	1,4	0,896	0,97
BTK	N/A	N/A	N/A	-1,342	-1,342	-2,449	-1	-0,557
5-azacytidine	1,964	1,941	1,4	N/A	N/A	N/A	0,818	0,555
LGALS3	1,172	N/A	N/A	1,27	1,953	N/A	0,985	1,286
BRCA1	N/A	1,749	N/A	0,816	-0,254	2,449	0	1,393
aspirin	-1,387	N/A	-1,067	-1,387	-0,478	-1,982	N/A	0,342
Lh	N/A	-1	N/A	-2,309	0,625	N/A	-0,748	1,951
cisplatin	0,772	2,451	0,374	0,854	0,536	0,435	0,75	0,369
fenamic acid	0,651	N/A	N/A	1,129	1,373	1	1,521	0,863
ESR1	0,932	0,357	-1,107	-0,148	-0,63	-1,534	-0,368	-1,428
bexarotene	-0,765	0,152	-0,44	-1,471	-1,316	-0,068	-1,054	-1,195
NRAS	0,896	0,152	-0,762	-0,198	0,478	-2	-1,026	0,933
LEP	0,699	0,747	1,505	0,668	1,196	N/A	0,592	0,991
ERK	0,328	1,671	0,668	0,803	0,301	0,865	0,328	1,429
HMGA1	-1,442	-2,236	-0,594	-0,322	-1,287	-0,133	-0,369	N/A
GLI1	-0,061	-0,493	-0,83	-1,388	-0,621	-1,715	-1,019	-0,235
metronidazole	-1,342	N/A	N/A	-1	-1,633	N/A	-1,342	-1
ESRRA	1,982	1,912	N/A	N/A	0,555	N/A	1,71	0,152
VCAN	1	0,447	0	0,898	0,607	1,897	0,302	1,127
KDM5B	1,073	-1,342	-0,221	1,073	0,293	-0,585	-0,091	1,59
AKT1	0,815	2,032	0,931	-0,593	-0,75	0,463	-0,284	-0,376
VEGFA	1,206	-1,521	0,703	0,235	0,38	1,059	0,275	0,851
SMARCB1	-0,625	N/A	N/A	-1,673	-1,23	0,937	-1,23	-0,524
IFNAR1	N/A	N/A	N/A	1,994	N/A	2,229	1,994	N/A
E. coli serotype 0127B8 lipopolysaccharide	0,651	N/A	0,651	0,97	0,57	1,432	0,97	0,97
ethanol	0,506	2,189	1,131	0,496	-0,267	0,087	0,811	0,721
RASSF1	0,64	1,633	N/A	0,64	0,508	-1,067	-0,478	1,238
tyrphostin AG 1478	-0,911	N/A	N/A	-1,27	-0,911	N/A	-0,911	-2,194
ZFP36	N/A	N/A	N/A	-1,981	-1,98	N/A	N/A	-2,211
ETS1	N/A	N/A	N/A	1,987	1,969	N/A	N/A	2,213
LARP1	N/A	N/A	N/A	3,159	N/A	N/A	3	N/A
TEAD4	2,213	N/A	N/A	1,715	1,982	N/A	N/A	-0,246
NR4A1	N/A	N/A	N/A	1,751	2,418	N/A	N/A	1,977
MLXIPL	N/A	N/A	N/A	-3,317	N/A	N/A	-2,828	N/A
rottlerin	N/A	-1,927	N/A	-1,982	N/A	N/A	N/A	-2,16
NCOA3	N/A	N/A	N/A	1,758	1,758	N/A	1,944	0,596
Pam3-Cys-Ser-Lys4	N/A	N/A	N/A	1,235	N/A	2,407	1,235	1,176
mibolerone	N/A	N/A	N/A	1,4	1,915	N/A	2,2	0,522
colistin	-1,342	N/A	N/A	-1	-1,342	N/A	-1,342	-1
kanamycin A	-1,342	N/A	N/A	-1	-1,342	N/A	-1,342	-1
15-deoxy-delta-12,14 -PGJ 2	-0,166	2,384	0,15	-0,707	-0,964	-0,904	-0,214	-0,517
geldanamycin	1,633	N/A	0,87	0,113	-1	-1	0,915	-0,469
Tlr	N/A	N/A	N/A	2	N/A	2	2	N/A
prexasertib	N/A	2	N/A	N/A	N/A	2	N/A	2
ACOX1	-0,378	-1,89	0	-0,447	-0,378	-1	-1,195	-0,707
APP	1,127	1,573	0,292	-0,115	0,303	1,451	0,213	0,904
KAT5	N/A	N/A	N/A	1,982	N/A	N/A	1,982	2
SMAD4	-0,676	0,771	-1,133	-0,748	-0,468	-0,901	-1,103	-0,15
PIK3CG	N/A	N/A	N/A	-1,98	N/A	-1,98	-1,98	N/A
gentamicin	0	1,912	0,113	1	0,418	-0,816	1,364	-0,302
TO-901317	-0,555	N/A	-1,067	-1,067	-1,067	N/A	-1,067	-1,067
Go 6976	N/A	N/A	N/A	-1,96	-1,96	N/A	N/A	-1,96
3,3'-diindolylmethane	-0,389	1,117	N/A	1,242	0,327	1,013	1,242	0,533
TAZ	N/A	N/A	N/A	1,131	1,597	2,219	0,896	N/A
methylnitronitrosoguanidine	N/A	1,959	N/A	1,938	N/A	N/A	N/A	1,931
raloxifene	N/A	N/A	N/A	1,387	2,219	N/A	N/A	2,219
SRC	N/A	N/A	N/A	1,539	1,103	N/A	1,206	1,972
carbon tetrachloride	0,179	1,981	0,849	0,762	-0,414	N/A	0,762	0,871
oxaliplatin	0,447	1,181	N/A	1,4	0	0,447	0,928	1,412
IGF1	1,068	1,942	0,718	-0,35	0,327	1,087	-0,094	-0,175
calphostin C	-0,958	N/A	N/A	-1,253	-0,054	-0,958	-0,958	-1,577
IL4	-0,26	2,12	0,264	0,12	-0,744	-0,514	0,962	-0,764
SMAD3	2,222	N/A	0,529	1,407	0,409	0,389	0,349	0,44
triamterene	0,447	N/A	N/A	1	1,265	1	1,32	0,707
allopurinol	0,447	N/A	N/A	1	1,265	1	1,265	0,707
gentamicin C	0,447	N/A	N/A	1	1,265	1	1,265	0,707
TNFRSF1A	N/A	N/A	N/A	0,625	0,946	1,103	1,951	1,044
BCL6	N/A	N/A	N/A	-1,067	-1,067	-1,067	-1,067	-1,387
FOXO1	0,526	0,376	-0,094	0,565	0,839	1,065	0,807	1,27
N-Ac-Leu-Leu-norleucinal	-0,336	0,607	0,246	-1,478	-0,642	-1,273	-0,932	-0,004
genistein	0,431	1,923	0,047	-0,075	0,51	0,758	0,535	1,226
imatinib	N/A	0,372	N/A	-0,277	-1,455	-0,882	-1,4	-1,109

STAT5B	0,046	0,128	-0,659	-1,847	-0,267	0,906	-1,412	0,215
Vegf	N/A	N/A	N/A	-1,178	-2,396	N/A	N/A	-1,891
RET	1,103	N/A	N/A	1,406	0,218	0,254	1,534	0,946
dehydroisoandrosterone	1,091	N/A	N/A	1,091	1,091	N/A	1,091	1,091
bortezomib	0,055	1,688	-0,437	0,913	0,39	0,146	1,132	0,688
PLCG2	N/A	N/A	N/A	-1,231	-0,927	-2,626	-0,663	N/A
sulforafan	-0,128	2,169	0,64	-0,478	-1,037	-0,152	0,342	0,497
GPER1	0,41	2	0,832	0,41	0,518	N/A	N/A	1,213
enalapril	-1,432	N/A	N/A	-1,432	-0,104	N/A	-1,432	-0,97
streptozocin	N/A	1,727	N/A	N/A	-0,666	N/A	-1,671	-1,298
PRMT1	1,154	N/A	1,969	1,154	0,537	N/A	0,537	N/A
BRD4	N/A	N/A	N/A	1,982	1,387	N/A	N/A	1,982
let-7	-1,956	N/A	N/A	-1,486	-0,437	N/A	N/A	-1,47
SIM1	N/A	N/A	N/A	1,342	N/A	2	2	N/A
ARNT2	N/A	N/A	N/A	1,342	N/A	2	2	N/A
HIF1A	0,107	2,329	-0,365	0,095	-0,827	0,659	0,518	-0,439
paclitaxel	0,085	0,614	1,378	0,987	0,162	1,233	0,704	-0,161
PPARGC1A	1,153	-0,64	N/A	0,616	1,059	-0,17	N/A	1,678
CLPP	N/A	N/A	N/A	-0,928	-0,6	-2,2	-1,4	-0,186
KAT2A	1,114	N/A	N/A	1,114	1,673	N/A	1,41	N/A
IGF2	-1,103	0	-1,103	-1,103	-0,625	N/A	-1,103	-0,254
WNT3A	1,91	N/A	0,201	N/A	1,256	N/A	1,91	N/A
Alpha catenin	N/A	N/A	N/A	-1,067	-1,491	N/A	-1,741	-0,97
Pdgf (complex)	0,714	0,499	N/A	1,082	N/A	N/A	0,796	2,173
H89	-0,686	N/A	-0,686	-1,067	N/A	N/A	-1,067	-1,715
let-7a-5p (and other miRNAs w/seed GAGGUAG)	-2,419	-2,231	N/A	N/A	0,556	N/A	N/A	N/A
TP63	-1,409	1,436	N/A	-0,36	0,032	N/A	0,248	1,7
BCR (complex)	1,961	N/A	N/A	N/A	1,461	N/A	N/A	1,761
ethionine	0	N/A	N/A	1,134	1,414	1	0,816	0,816
SIRT1	0,707	N/A	1,342	0,745	-0,578	-1,732	-0,043	0
PRKAA1	1,342	0,496	0	0,816	1,387	N/A	N/A	1
ATF4	0,294	N/A	-0,311	-0,728	-0,792	1,96	0,555	-0,368
mir-96	N/A	N/A	N/A	1	1	2	N/A	1
tacrolimus	-0,73	N/A	-0,853	-0,853	-1,029	0	-0,649	-0,853
pyrrolidine dithiocarbamate	N/A	N/A	N/A	-1,491	-1,154	N/A	-1,154	-1,154
CDKN1B	-0,618	N/A	-0,618	-0,312	-0,895	-0,994	-0,618	-0,848
IL10RA	N/A	N/A	N/A	-0,896	N/A	-1,982	N/A	-2
vorinostat	N/A	N/A	N/A	N/A	-2,4	N/A	-1,434	-1,01
COPS5	-0,707	-1,633	-0,816	-1,195	0	N/A	-0,478	0
RAF1	0,963	N/A	N/A	0,953	0,183	N/A	0,861	1,854
miR-29b-3p (and other miRNAs w/seed AGCACCA)	-1,502	-2,762	N/A	N/A	0,2	N/A	-0,218	0,128
thapsigargin	N/A	1,567	N/A	-0,739	-1,252	N/A	N/A	-1,252
ERBB3	0,186	N/A	-1	-1,342	-0,186	-1,387	-0,707	0
glucocorticoid	1,118	N/A	0,817	0,721	1,387	0,721	N/A	N/A
promegestone	1,098	N/A	N/A	N/A	1,098	1,098	N/A	1,468
miR-16-5p (and other miRNAs w/seed AGCAGCA)	N/A	-0,636	N/A	N/A	N/A	N/A	-2,167	-1,947
KLF4	0,455	N/A	N/A	-0,326	-0,524	-1,879	0,737	-0,823
miR-1-3p (and other miRNAs w/seed GGAAUGU)	-1,406	N/A	N/A	-0,468	-0,785	N/A	-1,98	-0,092
IGF1R	0,257	2,183	N/A	-0,902	-0,586	N/A	-0,713	-0,084
FGF7	N/A	N/A	N/A	1,195	0,44	N/A	0,882	2,2
methotrexate	-2,219	N/A	N/A	N/A	-1,134	N/A	-1,342	N/A
hexachlorobenzene	0,277	N/A	N/A	0,853	1,117	1	0,853	0,563
PTEN	0,022	0,171	-0,918	0,609	-0,805	-0,918	-0,761	-0,45
DUSP1	-0,447	N/A	-1	-0,096	-0,456	-1,938	0,254	-0,456
thioacetamide	0,175	N/A	N/A	1,238	0,632	1	1,294	0,269
Immunoglobulin	-1,571	-0,958	-0,155	-0,044	-0,19	0,819	-0,369	-0,497
CD44	-0,651	1	N/A	-1,131	-0,039	N/A	-0,651	-1,131
HRAS	0,669	0,525	0,071	0,501	0,492	-0,093	-0,303	1,877
CEBPA	0,027	1,842	0,931	-0,22	0,423	-0,154	0,342	0,562
NR1H4	2	N/A	N/A	N/A	1,499	N/A	N/A	1
IKBKG	N/A	N/A	N/A	0,351	0,588	1,804	1,16	0,588
PI3K (complex)	0,541	1,686	0,541	0,048	-0,07	N/A	-0,392	1,195
HGF	-0,765	0,605	-0,838	-0,1	-0,948	-0,186	-0,457	-0,558
GAST	N/A	N/A	N/A	0,596	N/A	1,936	1,919	N/A
SMAD7	-1,958	-0,991	N/A	-0,439	-0,339	N/A	0,722	N/A
phytohemagglutinin	N/A	N/A	N/A	N/A	1,071	1,412	1,908	N/A
FOXO4	-0,87	-0,412	-0,557	0,343	0,649	0	-0,2	1,334
FCGR2A	N/A	N/A	N/A	1,067	N/A	2,219	1,067	N/A
STAT2	N/A	N/A	N/A	0,586	0,646	2,172	0,302	0,646
YAP1	N/A	1,22	N/A	1,009	0,298	N/A	1,82	N/A
TEAD1	2,2	N/A	N/A	N/A	1,964	N/A	N/A	-0,179
IFN type 1	N/A	N/A	N/A	1,949	N/A	2,391	N/A	N/A
ciglitazone	-0,581	1,958	-0,289	-0,533	-0,263	0,034	-0,263	-0,411
CALCA	0	N/A	0,447	-0,816	-0,816	-0,447	-0,447	-1,342
methylprednisolone	0,937	1,123	-0,254	-0,244	0,566	0,263	0,566	-0,328
ascorbic acid	0,447	N/A	1	0,218	0,943	N/A	N/A	1,671
QKI	N/A	N/A	N/A	-0,713	-1,342	N/A	-0,243	-1,974
Growth hormone	N/A	N/A	N/A	1,181	1,459	N/A	0,422	1,181
puromycin aminonucleoside	-0,61	N/A	0,2	0,845	1,075	0,063	0,845	0,6
UCHL1	N/A	N/A	-0,83	-0,297	-0,83	-1,96	N/A	-0,321
RNY3	N/A	N/A	N/A	1	1	2,236	N/A	N/A
MAPK9	N/A	-0,619	0,762	0,64	1,109	0,053	0,64	0,405
epoxomicin	N/A	N/A	0,353	1,412	0,674	N/A	1,412	0,353
TFAP2A	N/A	N/A	N/A	1,937	N/A	N/A	1,067	1,192
MAPK14	0,651	N/A	N/A	1,194	0,014	1,222	0,665	0,443
MAVS	N/A	N/A	N/A	N/A	1,98	2,2	N/A	N/A
miR-338-3p (miRNAs w/seed CCAGCAU)	-2,164	-2	N/A	N/A	N/A	N/A	N/A	N/A
HTT	1,964	2,191	N/A	N/A	N/A	N/A	N/A	N/A
COL18A1	-0,762	N/A	N/A	-0,762	0,057	-1,461	-0,334	-0,762
EGR1	-0,447	1,961	1	-0,083	0,128	N/A	0,264	0,254
mitomycin C	N/A	2,172	N/A	N/A	-1,953	N/A	N/A	N/A
USP18	N/A	N/A	N/A	-1,067	N/A	-1,982	-1,067	N/A
mir-29	-1,96	-1,192	N/A	N/A	0,881	N/A	N/A	0,08
PLG	N/A	N/A	N/A	2,169	N/A	N/A	1,934	N/A
OGT	-0,577	1,214	N/A	0,447	-0,447	N/A	0,816	0,6
miR-199a-5p (and other miRNAs w/seed CCAGUGU)	-1,387	N/A	N/A	-1,172	-1,387	N/A	0,152	N/A
SOX11	N/A	N/A	N/A	-1,664	-1,067	-1,342	N/A	N/A
CYP19A1	N/A	N/A	N/A	N/A	1,029	N/A	1,981	1,029
miR-21-5p (and other miRNAs w/seed AGCUUAU)	-1,967	-1,095	N/A	N/A	N/A	N/A	N/A	-0,964
BDNF	0,351	-1	N/A	-0,156	0,66	N/A	1,316	0,522
TSC2	0,216	-1,091	N/A	0,426	0,364	0	-0,713	1,195
CRP	N/A	N/A	N/A	N/A	2	N/A	2	N/A
PS-1145	N/A	N/A	N/A	N/A	-2	N/A	N/A	-2
S100A9	N/A	N/A	N/A	2	N/A	N/A	N/A	2
NOTCH1	0,862	N/A	N/A	0,681	0,358	N/A	1	1,099
pembrolizumab	N/A	N/A	N/A	2	N/A	2	N/A	N/A
KRAS	0,418	-0,487	-0,368	0,548	-0,096	-0,329	1,04	0,702
dimethyl itaconate	N/A	N/A	N/A	-1	-1,982	N/A	-1	N/A
PDLIM2	N/A	N/A	N/A	-1,387	-1,387	-0,128	-1,067	N/A
IKZF3	N/A	N/A	N/A	-1,982	N/A	-1,982	N/A	N/A
PTGS2	1,131	N/A	N/A	0,492	N/A	1,131	0,808	0,4
RARA	0,447	-1	N/A	-0,632	-0,302	-1,265	0	-0,302
SHC1	0,271	N/A	1,009	0,421	-0,861	-0,106	0,465	0,811
TP73	N/A	1,227	N/A	0,596	-0,978	-0,808	-0,228	-0,105
NS-398	N/A	N/A	N/A	-1,969	N/A	N/A	-1,969	N/A
IRF2	0,728	N/A	N/A	0,728	1,4	0,277	0,277	0,522

GW3965	N/A	N/A	N/A	-1,964	-1,964	N/A	N/A	N/A
ADIPOQ	-0,82	N/A	-0,82	-1,184	N/A	-0,82	N/A	0,281
FLT1	1	-0,447	N/A	N/A	0,447	0	1	1
PTPN6	N/A	N/A	N/A	-1,091	-1,091	N/A	-0,6	-1,091
GH1	-0,447	N/A	N/A	-0,447	-1,633	N/A	-1,342	N/A
PTP4A1	0	0,915	N/A	1,253	-0,808	N/A	-0,492	-0,391
CDKN1A	0,135	0,13	0,085	1,197	-0,488	0,543	0,648	0,632
arsenite	N/A	2,141	N/A	N/A	0,6	N/A	1,091	N/A
mir-8	-0,973	-0,273	-0,598	-0,241	0,215	-0,598	-0,681	0,22
lomustine	0,447	N/A	N/A	0,707	1	N/A	1,265	0,378
CIITA	N/A	N/A	N/A	1,264	N/A	1,264	1,264	N/A
FGFR1	N/A	N/A	N/A	1,346	N/A	N/A	N/A	2,421
inosine	0,447	N/A	N/A	0	1,219	N/A	1,093	1
TLR2	N/A	-0,762	N/A	N/A	1,686	N/A	0,548	0,749
MKNK1	1,134	N/A	N/A	-0,447	0,707	N/A	0,447	-1
IPMK	N/A	N/A	N/A	N/A	0,625	N/A	2	1,103
ADRB	0,781	-1,172	N/A	-0,13	0,97	0,283	0,243	0,115
SMARCA5	N/A	-2	N/A	-0,6	N/A	N/A	-1,091	N/A
JAG1	N/A	1,446	N/A	N/A	2,205	N/A	N/A	N/A
Ca2+	0,555	N/A	0,686	-0,391	1,304	N/A	N/A	0,714
KLF11	-0,686	N/A	N/A	-1,715	-0,152	N/A	N/A	-1,067
IL33	0,585	N/A	0,585	0,585	0,106	0,585	0,585	0,585
RAS	N/A	N/A	1,202	1,117	N/A	0,305	N/A	0,976
IRF5	N/A	N/A	N/A	0,692	N/A	2,213	0,692	N/A
EGFR	-0,455	0,028	-1,225	0,178	-0,036	0,217	1,051	0,401
IFI16	0,277	0,661	N/A	0,44	N/A	0,882	0,882	0,444
FAS	-0,435	-0,209	-0,389	-0,908	-0,959	0,134	-0,275	-0,272
Collagen type I (complex)	N/A	N/A	N/A	1,982	-0,147	N/A	N/A	1,432
IGFBP2	1	N/A	N/A	1	0,277	N/A	0,277	1
mycophenolic acid	-0,478	1	-0,152	-0,152	-0,896	-0,152	0,239	-0,478
PRDM1	0,78	N/A	0,152	-0,373	-0,312	-0,719	0,345	-0,835
COL5A1	-1,067	N/A	N/A	-1	-0,447	N/A	N/A	-1
vancomycin	-1,066	N/A	-0,896	-0,088	0,113	-0,788	0,075	-0,47
NORAD	1,342	2	N/A	N/A	-0,152	N/A	N/A	N/A
STAT3	-0,468	-0,418	0,563	-0,171	-0,416	0,879	-0,539	-0,038
ERK1/2	0,114	0,84	0,114	-0,443	-0,756	-0,82	-0,366	-0,031
calcitriol	0,057	1,408	0,44	0,438	-0,247	0,479	-0,307	-0,105
2-bromoethylamine	0,447	N/A	N/A	0,378	1	N/A	1,265	0,378
Collagen type IV	0	N/A	N/A	1	0,447	N/A	N/A	2
cadmium	N/A	N/A	N/A	1,982	N/A	N/A	N/A	1,458
FOXO3	-0,033	-0,528	-0,38	0,015	1,226	0,443	-0,138	0,677
KLF2	0,075	-0,596	N/A	0,482	1,118	0,088	0,82	0,258
metribolone	-0,033	1,82	0,687	0,061	0,163	0,33	-0,091	-0,251
PPARA	-1,079	N/A	-0,25	-0,976	-0,379	N/A	-0,606	-0,128
tunicamycin	N/A	1,281	N/A	N/A	-1,944	-0,192	N/A	N/A
GATA6	1,446	1,969	N/A	N/A	N/A	N/A	N/A	N/A
vitamin E	N/A	N/A	N/A	N/A	-1,406	N/A	-2	N/A
E2F3	0,447	N/A	N/A	N/A	0,816	N/A	1,134	1
VIP	0,849	N/A	0,849	-0,357	0,056	0,849	-0,357	0,056
ATG7	N/A	-0,728	N/A	-0,728	1,508	N/A	0,394	N/A
SOX4	N/A	N/A	N/A	N/A	-1,406	N/A	-1,948	N/A
CDKN2A	-0,228	1,064	N/A	0,108	-0,882	N/A	-0,25	0,817
4-hydroxytamoxifen	0,027	2,183	N/A	0,332	0,027	0,332	-0,084	0,363
BMP10	N/A	N/A	N/A	-1	-1	0	-1,342	N/A
KLF5	1,922	N/A	N/A	N/A	1,412	N/A	N/A	N/A
EZH2	1,941	-0,842	N/A	N/A	N/A	0,52	N/A	N/A
lenalidomide	N/A	N/A	-0,651	1,408	N/A	1,109	N/A	0,13
6-hydroxydopamine	N/A	N/A	N/A	N/A	1,109	N/A	2,164	N/A
HOXA10	0	N/A	N/A	0,447	N/A	-1	0,816	1
MAP2K1	N/A	N/A	N/A	1,501	0,16	N/A	1,172	0,42
APC	-0,339	N/A	N/A	N/A	-0,692	N/A	-1	-1,214
mir-1	N/A	N/A	N/A	N/A	-1,067	N/A	-2,173	N/A
NFE2L1	N/A	N/A	N/A	N/A	1	N/A	2,236	N/A
FGF8	1,941	N/A	N/A	N/A	1,292	N/A	N/A	N/A
HSF1	N/A	0,415	N/A	1,192	-0,074	0,585	0,96	N/A
L-glutamic acid	N/A	N/A	N/A	1,164	-0,2	N/A	0,708	1,154
BMP4	0,895	1,985	N/A	N/A	0,34	N/A	N/A	N/A
ZBTB16	0	-1,981	0,152	N/A	0,152	-0,927	N/A	N/A
Bay 11-7082	N/A	N/A	-1,067	N/A	-1,067	N/A	N/A	-1,067
mir-155	-0,13	N/A	-0,13	-0,13	-0,798	-0,933	-0,485	-0,577
GAPDH	N/A	N/A	N/A	0	-0,816	-1,915	0	-0,447
RAC1	N/A	N/A	N/A	1,939	0,344	N/A	0,894	N/A
tetrachlorodibenzodioxin	0,484	-0,512	N/A	1,317	-0,048	N/A	0,094	0,716
miR-17-5p (and other miRNAs w/seed AAAGUGC)	-1,926	N/A	N/A	N/A	-1,231	N/A	N/A	N/A
levodopa	-0,426	0,378	N/A	0,777	0,612	N/A	-0,169	0,777
herbimycin	-1	N/A	N/A	-1,067	N/A	N/A	N/A	-1,067
GSTO1	N/A	1,131	N/A	N/A	N/A	-2	N/A	N/A
STING1	N/A	N/A	N/A	1,179	N/A	1,944	N/A	N/A
SP3	1,091	N/A	N/A	N/A	0,865	N/A	N/A	1,159
HNF4A	-0,102	N/A	0,508	-1,127	-0,708	0	-0,336	0,334
APOE	0	N/A	-1	-0,128	-0,239	-1,387	-0,342	N/A
IL10	N/A	1,997	N/A	N/A	0,072	N/A	N/A	1,002
aflatoxin B1	N/A	2,236	N/A	N/A	0	N/A	N/A	0,816
fenretinide	N/A	N/A	N/A	N/A	-1,109	-1,941	N/A	N/A
ANGPT2	N/A	N/A	N/A	-0,223	-0,156	N/A	1,951	0,714
ETV5	-1	N/A	N/A	0,272	-0,426	N/A	-0,52	0,816
PAX6	N/A	N/A	N/A	N/A	-1,206	N/A	-0,9	-0,911
KDM5A	N/A	N/A	N/A	1,387	0,555	N/A	N/A	1,067
SAFB2	N/A	N/A	N/A	-1	-1	N/A	-1	N/A
SAFB	N/A	N/A	N/A	-1	-1	N/A	-1	N/A
IFNGR1	N/A	N/A	N/A	1	N/A	N/A	1	1
bisindolylmaleimide I	-0,692	N/A	N/A	-0,218	-0,294	N/A	-1,067	-0,67
IL17A	0,798	0	0,768	-0,035	-0,174	0,398	0,378	0,372
carbon monoxide	N/A	N/A	N/A	-0,9	-1,109	N/A	-0,9	N/A
SRF	N/A	0,61	N/A	1,122	-0,094	N/A	-1,067	N/A
uranyl nitrate	-0,97	N/A	N/A	-0,784	0,218	N/A	0,114	0,762
butyric acid	-0,621	-0,561	-0,105	-0,255	-0,375	0	-0,819	-0,106
TWIST1	0,218	-0,277	N/A	0,218	-0,679	N/A	-0,446	-1
PDX1	N/A	N/A	N/A	N/A	1	N/A	1	0,832
bleomycin	0,246	N/A	N/A	1,308	0,507	N/A	N/A	0,742
HDAC2	1	-0,975	N/A	0,447	0	N/A	N/A	-0,377
Akt	-0,083	0,573	0,657	-0,352	-0,282	0,293	0,315	0,243
RGS2	N/A	N/A	N/A	-0,447	0	N/A	-1	1,342
VHL	0,359	-0,604	0,577	0,45	0,514	N/A	0,203	-0,055
PTH	N/A	N/A	N/A	-0,756	-1,206	N/A	N/A	-0,794
SMAD2	N/A	N/A	N/A	N/A	-1,106	N/A	-1,154	-0,492
TGFBR2	N/A	0,186	-0,594	N/A	0,543	N/A	0,371	1,044
MDK	1	N/A	N/A	1	0,728	N/A	N/A	N/A
nicotine	1,23	N/A	N/A	N/A	1,454	N/A	N/A	N/A
CDK19	N/A	-2,646	N/A	N/A	N/A	N/A	N/A	N/A
lysophosphatidic acid	N/A	N/A	N/A	1,898	0,748	N/A	N/A	N/A
IRF8	N/A	0,254	N/A	-0,18	-0,579	N/A	-0,898	-0,73
caffeic acid phenethyl ester	-0,927	N/A	0,132	-0,351	-0,715	N/A	-0,351	0,132
cobalt chloride	N/A	2,2	N/A	N/A	-0,132	N/A	-0,132	-0,132
RB1	N/A	-1	N/A	-0,243	1	N/A	0,277	-0,069
N-nitro-L-arginine methyl ester	N/A	-1,067	N/A	1,521	N/A	N/A	N/A	N/A

ADORA2A	0,378	N/A	N/A	0,816	0,478	N/A	0	0,896
trans-hydroxytamoxifen	1,067	0,152	N/A	0	N/A	1,342	N/A	0
carboplatin	N/A	N/A	N/A	0,64	0,956	N/A	0,956	0
hemin	0,22	N/A	1,264	N/A	-1,025	N/A	0,004	N/A
HMOX1	-1,406	N/A	N/A	N/A	-0,468	N/A	N/A	-0,625
Ap1	N/A	N/A	N/A	1,246	N/A	N/A	N/A	1,246
methamphetamine	N/A	N/A	N/A	1,091	N/A	N/A	1,4	N/A
mir-183	N/A	N/A	N/A	0,083	0,083	2,219	N/A	0,083
erlotinib	N/A	N/A	N/A	1,387	N/A	N/A	N/A	1,067
CD38	N/A	N/A	N/A	0	-0,555	N/A	0,896	-1
ACKR2	N/A	N/A	N/A	N/A	N/A	-2,449	N/A	N/A
KLF3	-1,134	N/A	N/A	0	-0,632	N/A	-0,302	-0,378
phenylbutazone	-0,651	N/A	N/A	-0,2	0,186	-0,152	0,594	-0,655
indomethacin	-0,822	N/A	-0,218	-0,441	-0,171	0,277	-0,436	-0,048
cycloheximide	0,28	-0,442	0,17	-0,198	-0,264	-0,136	-0,443	-0,418
HNF1B	0	N/A	N/A	N/A	1,342	N/A	N/A	1
temozolomide	-0,831	0,312	N/A	0,061	N/A	-0,831	0,061	0,185
NME1	N/A	N/A	N/A	0,943	N/A	N/A	1,33	N/A
sodium arsenite	N/A	N/A	N/A	1	N/A	N/A	1	0,254
POU2AF1	N/A	N/A	N/A	0,332	0,631	0,958	0	0,332
zinc	N/A	N/A	N/A	0,928	0,505	N/A	0,312	0,505
Y 27632	N/A	N/A	N/A	-1,109	-0,025	N/A	N/A	-1,109
idarubicin	N/A	2,236	N/A	N/A	N/A	N/A	N/A	N/A
CAB39L	N/A	N/A	N/A	-2,236	N/A	N/A	N/A	N/A
FAAH	N/A	N/A	N/A	2,236	N/A	N/A	N/A	N/A
emactuzumab	N/A	N/A	N/A	N/A	N/A	2,236	N/A	N/A
Mek	1,121	N/A	N/A	0,047	0,341	0,271	-0,289	0,164
alitretinoin	N/A	N/A	N/A	0,625	0,528	N/A	N/A	1,077
miR-30c-5p (and other miRNAs w/seed GUAAACA)	N/A	-2,221	N/A	N/A	N/A	N/A	N/A	N/A
HSF2	N/A	N/A	N/A	N/A	N/A	N/A	2,219	N/A
IFNE	N/A	N/A	N/A	N/A	N/A	2,219	N/A	N/A
hydroxyurea	N/A	2,209	N/A	N/A	N/A	N/A	N/A	N/A
WR 1065	N/A	2,2	N/A	N/A	N/A	N/A	N/A	N/A
1,4-bis[2-(3,5-dichloropyridyloxy)]benzene	N/A	N/A	N/A	N/A	N/A	N/A	N/A	2,2
S-nitrosoglutathione	N/A	N/A	N/A	N/A	1,131	N/A	N/A	1,067
MAP2K4	N/A	2,193	N/A	N/A	N/A	N/A	N/A	N/A
TCF7L2	N/A	N/A	N/A	0,827	1,178	N/A	N/A	0,186
nocodazole	N/A	2,19	N/A	N/A	N/A	N/A	N/A	N/A
NUPR1	N/A	2,186	N/A	N/A	N/A	N/A	N/A	N/A
methyl methanesulfonate	N/A	2,176	N/A	N/A	N/A	N/A	N/A	N/A
ADCYAP1	-0,333	0,816	N/A	0,102	-0,434	0	0,102	0,389
Pka	N/A	N/A	N/A	-0,747	0,068	-1,067	0,068	0,218
cytarabine	N/A	2,164	N/A	N/A	N/A	N/A	N/A	N/A
fluticasone propionate	N/A	N/A	N/A	-1	N/A	-1,134	N/A	N/A
GSK3B	-0,339	N/A	0,179	0,057	0,603	-0,339	0,057	0,55
glutamine	-0,6	N/A	0,555	0	-0,388	N/A	0	0,555
BACH1	N/A	N/A	N/A	0,64	0,391	N/A	-1,067	N/A
GABA	N/A	N/A	N/A	1,732	-0,333	N/A	0	0
ELK1	N/A	N/A	N/A	1,095	0,969	N/A	N/A	N/A
TGFB3	1,964	N/A	N/A	N/A	0,083	N/A	N/A	N/A
SUMO2	N/A	N/A	N/A	N/A	0,152	-1,109	-0,762	N/A
TGFA	1,021	N/A	N/A	N/A	N/A	N/A	N/A	0,991
RARB	N/A	N/A	N/A	-1	0,447	0,555	0	N/A
CAT	N/A	N/A	N/A	-2	N/A	N/A	N/A	N/A
TCF4	N/A	N/A	N/A	N/A	1	N/A	1	0
miR-335-3p (miRNAs w/seed UUUUCAU)	N/A	-2	N/A	N/A	N/A	N/A	N/A	N/A
CCR2	2	N/A	N/A	N/A	N/A	N/A	N/A	N/A
SERPINF1	N/A	N/A	N/A	-1	0	N/A	-1	N/A
HBEGF	N/A	N/A	N/A	N/A	2	N/A	N/A	N/A
SLC29A1	N/A	2	N/A	N/A	N/A	N/A	N/A	N/A
CCN2	N/A	2	N/A	N/A	N/A	N/A	N/A	N/A
5-N-ethylcarboxamido adenosine	N/A	N/A	N/A	N/A	N/A	N/A	-2	N/A
EPO	N/A	N/A	N/A	N/A	2	N/A	N/A	N/A
dimethyl sulfoxide	N/A	N/A	N/A	N/A	-1	N/A	N/A	-1
dinoprost	2	N/A	N/A	N/A	N/A	N/A	N/A	N/A
EHMT1	-1	N/A	N/A	-1	N/A	N/A	N/A	N/A
calyculin A	N/A	N/A	N/A	N/A	N/A	N/A	N/A	2
MAP2K6	N/A	N/A	N/A	N/A	N/A	1,997	N/A	N/A
CGAS	N/A	N/A	N/A	N/A	N/A	N/A	N/A	1,993
THRB	N/A	N/A	N/A	N/A	-1,992	N/A	N/A	N/A
NLRP12	N/A	N/A	N/A	1,99	N/A	N/A	N/A	N/A
LRP6	N/A	N/A	N/A	N/A	1,987	N/A	N/A	N/A
TREX1	N/A	N/A	N/A	N/A	N/A	-1,983	N/A	N/A
TREM1	N/A	N/A	N/A	N/A	-0,64	N/A	N/A	-1,342
MAP2K3	N/A	N/A	N/A	1,982	N/A	N/A	N/A	N/A
POLR2M	N/A	-1,982	N/A	N/A	N/A	N/A	N/A	N/A
NR3C2	N/A	N/A	N/A	-1,982	N/A	N/A	N/A	N/A
BO-653	N/A	N/A	N/A	N/A	N/A	N/A	-1,982	N/A
carbamazepine	N/A	N/A	N/A	N/A	N/A	N/A	1,982	N/A
CX-5461	N/A	1,982	N/A	N/A	N/A	N/A	N/A	N/A
probucol	N/A	N/A	N/A	N/A	N/A	N/A	-1,982	N/A
AS1842856	N/A	N/A	N/A	N/A	N/A	N/A	N/A	-1,982
CNOT7	N/A	N/A	N/A	N/A	N/A	-1,98	N/A	N/A
NEK10	N/A	1,98	N/A	N/A	N/A	N/A	N/A	N/A
imiquimod	N/A	N/A	N/A	N/A	N/A	1,974	N/A	N/A
mir-25	N/A	-1,97	N/A	N/A	N/A	N/A	N/A	N/A
sodium orthovanadate	N/A	N/A	N/A	N/A	N/A	N/A	N/A	1,969
cholesterol	0,351	N/A	-0,492	0,351	-0,106	N/A	0,663	N/A
benzo(a)pyrene	N/A	1,962	N/A	N/A	N/A	N/A	N/A	N/A
reactive oxygen species	N/A	1,96	N/A	N/A	N/A	N/A	N/A	N/A
advanced glycation end-products	N/A	N/A	N/A	N/A	1,96	N/A	N/A	N/A
pifithrin alpha	N/A	-1,96	N/A	N/A	N/A	N/A	N/A	N/A
okadaic acid	N/A	N/A	N/A	N/A	1,96	N/A	N/A	N/A
USF2	N/A	N/A	N/A	N/A	N/A	1,958	N/A	N/A
MET	N/A	N/A	N/A	N/A	0,865	N/A	N/A	1,091
morphine	N/A	N/A	N/A	N/A	N/A	N/A	1,954	N/A
glutathione	N/A	N/A	N/A	N/A	N/A	N/A	N/A	1,951
doxifluridine	N/A	1,951	N/A	N/A	N/A	N/A	N/A	N/A
IFIH1	N/A	N/A	N/A	N/A	N/A	1,951	N/A	N/A
hydrocortisone	0	N/A	N/A	0	-0,816	N/A	-1,134	0
mir-34	N/A	N/A	N/A	-1,948	N/A	N/A	N/A	N/A
TSH	N/A	N/A	N/A	N/A	N/A	N/A	N/A	1,948
TBK1	N/A	N/A	N/A	N/A	N/A	N/A	N/A	-1,941
NGLY1	N/A	N/A	N/A	-1,172	N/A	-0,762	N/A	N/A
1-methyl-4-phenyl-1,2,3,6-tetrahydropyridine	N/A	N/A	N/A	N/A	N/A	N/A	1,933	N/A
LEF1	N/A	N/A	N/A	N/A	0,824	1,109	N/A	N/A
lithium chloride	0,391	N/A	N/A	0,115	0	N/A	-0,728	-0,692
F2R	N/A	N/A	N/A	1,922	N/A	N/A	N/A	N/A
INS	N/A	1,117	N/A	N/A	0,298	N/A	N/A	0,504
alvocidib	N/A	N/A	N/A	N/A	N/A	N/A	-1,914	N/A
MDM2	N/A	-1,912	N/A	N/A	N/A	N/A	N/A	N/A
TFRC	N/A	N/A	N/A	N/A	0,555	N/A	-0,277	1,067
ZNF106	0	N/A	N/A	0,447	-1	N/A	-0,447	N/A
TREM2	-0,447	N/A	N/A	0	-0,447	1	0	0
PLAUR	N/A	N/A	N/A	1,701	-0,187	N/A	N/A	N/A
palmitic acid	N/A	1,188	-0,202	N/A	N/A	N/A	N/A	0,498

NFYA	N/A	N/A	N/A	N/A	0,762	N/A	N/A	1,109
PDGFB	N/A	N/A	N/A	N/A	1,858	N/A	N/A	N/A
WT1	N/A	0,685	N/A	N/A	N/A	-1,172	N/A	N/A
prostaglandin E2	N/A	-0,728	-0,054	0,367	0,63	N/A	N/A	0,075
4-phenylbutyric acid	-0,651	N/A	0,191	-0,152	0,057	0,277	0,277	0,247
LGALS1	N/A	N/A	N/A	N/A	-0,78	N/A	N/A	1,067
silibinin	-0,754	0,317	N/A	N/A	0,209	N/A	N/A	-0,543
TGFB2	0,849	N/A	N/A	N/A	-0,956	N/A	N/A	N/A
PTGER4	N/A	N/A	N/A	N/A	-0,447	N/A	N/A	-1,342
SHH	1,119	N/A	N/A	-0,197	0,17	N/A	N/A	-0,301
L-triiodothyronine	-0,202	1,009	N/A	-0,556	N/A	N/A	N/A	-0,015
androgen	0,555	N/A	1,067	N/A	0,152	N/A	N/A	N/A
TAL1	N/A	0,147	N/A	N/A	N/A	-1	N/A	-0,625
Sb202190	N/A	N/A	N/A	N/A	0,478	-1,109	N/A	0,152
MRTFA	N/A	N/A	N/A	1,051	0,677	N/A	N/A	N/A
PARP1	N/A	-1,049	N/A	N/A	N/A	N/A	0,207	0,452
SNAI1	0,132	N/A	N/A	N/A	-0,382	N/A	N/A	-1,192
ERBB4	N/A	1,067	N/A	N/A	0,243	N/A	N/A	-0,391
propofol	N/A	N/A	N/A	N/A	N/A	N/A	-1,698	N/A
CYB561A3	N/A	N/A	N/A	N/A	0,849	N/A	0,849	N/A
PPARD	N/A	N/A	N/A	0,2	0,2	-0,339	-0,73	-0,218
eflornithine	0	N/A	N/A	0	0	1	0	0,678
SUMO3	N/A	N/A	N/A	N/A	0,152	-0,762	-0,762	N/A
GNA12	1	N/A	N/A	N/A	0,655	N/A	N/A	N/A
E2F1	0,478	0,403	0,152	-0,14	0	0	0,308	-0,173
AREG	N/A	N/A	N/A	0,046	0,625	0,254	0,254	0,456
2,5-bis(5-hydroxymethyl-2-thienyl)furan	N/A	1,633	N/A	N/A	N/A	N/A	N/A	N/A
SB-431542	N/A	N/A	N/A	N/A	-0,555	N/A	-1,067	N/A
RHOA	N/A	N/A	N/A	N/A	N/A	N/A	N/A	1,586
FBXW7	N/A	N/A	N/A	N/A	-0,577	N/A	-1	N/A
phenacetin	0	N/A	N/A	0,239	0,563	N/A	0,647	-0,128
melatonin	0,218	N/A	N/A	-0,697	0,189	N/A	-0,251	0,218
LUCAT1	N/A	N/A	N/A	1	N/A	N/A	N/A	0,528
PPARG	0,157	N/A	N/A	0,157	1,211	N/A	N/A	N/A
bafilomycin A1	N/A	N/A	N/A	N/A	-0,762	N/A	N/A	-0,762
RUNX2	-0,714	N/A	N/A	0,025	0,406	N/A	0	0,369
CDK4/6	N/A	N/A	N/A	0,555	N/A	N/A	-0,956	N/A
LIF	N/A	N/A	N/A	N/A	1,506	N/A	N/A	N/A
ANXA2	N/A	-1,503	N/A	N/A	N/A	N/A	N/A	N/A
ILF3	-0,218	N/A	N/A	0,848	-0,218	N/A	-0,218	N/A
HDAC1	-0,152	-0,799	N/A	0,218	0	N/A	N/A	-0,33
simvastatin	N/A	N/A	N/A	-0,514	-0,337	N/A	-0,15	-0,498
L-methionine	N/A	N/A	N/A	N/A	-1,342	N/A	N/A	0,152
IL12 (complex)	N/A	N/A	N/A	0,182	N/A	N/A	1,122	0,182
triptolide	N/A	N/A	N/A	0,152	0,447	N/A	N/A	0,868
phosphate	N/A	N/A	N/A	N/A	-1,452	N/A	N/A	N/A
HSP90B1	N/A	N/A	N/A	0	0,447	N/A	0	1
Firre	N/A	N/A	N/A	1	0,447	N/A	N/A	0
CX3CL1	N/A	N/A	N/A	0	0,447	1	N/A	N/A
gemcitabine	-0,173	N/A	-0,173	-0,576	-0,173	-0,173	-0,173	N/A
NFAT5	N/A	N/A	N/A	0,277	0,64	0,239	N/A	0,277
WNT5A	N/A	N/A	N/A	N/A	0,254	N/A	1,165	N/A
MAPK8	N/A	N/A	-0,152	0,577	0	0,577	0	0,108
captopril	0	N/A	N/A	0,342	0,726	N/A	0,342	0
CIP2A	N/A	N/A	N/A	N/A	N/A	N/A	N/A	-1,4
thyroid hormone	N/A	N/A	N/A	N/A	N/A	N/A	-0,581	0,818
anisomycin	N/A	N/A	N/A	-0,283	-0,346	N/A	N/A	0,768
atorvastatin	0,176	N/A	N/A	N/A	-0,511	0,176	-0,511	N/A
SREBF1	0,058	N/A	N/A	N/A	-0,643	N/A	-0,643	N/A
ROCK2	N/A	N/A	N/A	N/A	1,342	N/A	N/A	N/A
HOXA4	N/A	N/A	N/A	0,97	0,347	N/A	N/A	N/A
ITGB1	N/A	0,557	N/A	N/A	0,152	N/A	N/A	0,607
CpG oligonucleotide	N/A	N/A	N/A	1,091	N/A	N/A	0,218	N/A
YY1	N/A	N/A	N/A	N/A	N/A	N/A	N/A	-1,303
PKM	N/A	N/A	N/A	1,029	-0,035	N/A	-0,035	0,192
IKBKE	N/A	N/A	N/A	0,152	N/A	0,478	0,651	N/A
NRF1	N/A	N/A	N/A	N/A	0,359	N/A	0,919	N/A
DGCR8	N/A	N/A	N/A	N/A	-1,274	N/A	N/A	N/A
platelet activating factor	N/A	N/A	N/A	N/A	-0,831	N/A	N/A	0,44
FMR1	N/A	N/A	N/A	0,192	0,192	N/A	-0,849	N/A
NOTCH3	N/A	1,124	N/A	N/A	-0,108	N/A	N/A	N/A
INSR	N/A	N/A	N/A	-0,277	0,555	N/A	N/A	0,391
palbociclib	N/A	1	N/A	N/A	N/A	N/A	N/A	0,218
EPAS1	N/A	0,054	0,252	N/A	N/A	N/A	N/A	-0,902
baicalein	N/A	N/A	N/A	N/A	N/A	N/A	-1,205	N/A
26s Proteasome	N/A	N/A	N/A	N/A	N/A	N/A	-1,196	N/A
apigenin	-0,762	N/A	N/A	0,152	N/A	N/A	0,152	-0,13
BMP2	-0,911	N/A	N/A	N/A	-0,284	N/A	N/A	N/A
topotecan	N/A	1,195	N/A	N/A	N/A	N/A	N/A	N/A
verapamil	N/A	N/A	N/A	N/A	N/A	N/A	-1,192	N/A
BTG2	0,369	N/A	0,025	0,025	0,369	N/A	0,025	0,369
DLL4	N/A	N/A	N/A	N/A	-1,154	N/A	N/A	N/A
lovastatin	N/A	0,883	N/A	N/A	0	N/A	0	0,254
docetaxel	N/A	-0,063	N/A	N/A	N/A	N/A	N/A	-1,069
CLU	N/A	N/A	N/A	N/A	N/A	N/A	N/A	-1,131
SB 216763	N/A	N/A	N/A	1,131	N/A	N/A	N/A	N/A
CPT1B	N/A	N/A	N/A	1,131	N/A	N/A	N/A	N/A
NDRG1	N/A	-1,124	N/A	N/A	N/A	N/A	N/A	N/A
TCR	N/A	N/A	N/A	-0,222	0,52	N/A	0,309	0,068
tert-butyl-hydroquinone	N/A	N/A	0,924	N/A	-0,186	N/A	N/A	N/A
mir-193	N/A	N/A	N/A	N/A	N/A	N/A	N/A	-1,103
IRF6	N/A	N/A	N/A	1,103	N/A	N/A	N/A	N/A
bezafibrate	N/A	N/A	N/A	N/A	-1,103	N/A	N/A	N/A
GNAQ	N/A	N/A	N/A	N/A	N/A	N/A	1,091	N/A
phenobarbital	N/A	1,091	N/A	N/A	N/A	N/A	N/A	N/A
N-methyl-D-aspartate	N/A	N/A	N/A	1,091	0	N/A	N/A	N/A
DDX5	N/A	N/A	N/A	-0,82	N/A	N/A	0,254	N/A
torin1	N/A	N/A	N/A	-0,294	-0,342	N/A	-0,437	N/A
HOXC8	N/A	N/A	N/A	N/A	-1,067	N/A	N/A	N/A
miR-125b-5p (and other miRNAs w/seed CCCUGAG)	N/A	N/A	N/A	N/A	N/A	N/A	-1,067	N/A
mir-223	N/A	N/A	N/A	N/A	-1,067	N/A	N/A	N/A
birabresib	N/A	N/A	N/A	N/A	N/A	N/A	N/A	-1,067
ROCK	N/A	N/A	N/A	N/A	1,067	N/A	N/A	N/A
ALB	N/A	N/A	N/A	N/A	N/A	N/A	N/A	1,067
RUNX1	N/A	0	N/A	N/A	1,067	N/A	N/A	0
oleic acid	N/A	N/A	N/A	0,372	-0,322	N/A	0,372	N/A
MEN1	N/A	N/A	N/A	N/A	N/A	N/A	N/A	1,059
mir-148	N/A	N/A	N/A	N/A	-1,057	N/A	N/A	N/A
TWIST2	N/A	N/A	N/A	N/A	-0,988	N/A	N/A	-0,068
DDX25	N/A	N/A	N/A	0,283	N/A	N/A	-0,762	N/A
LCN2	N/A	N/A	N/A	N/A	0,277	N/A	0,762	N/A
KDM3A	N/A	N/A	N/A	N/A	1,02	N/A	N/A	N/A
SASH1	N/A	N/A	N/A	N/A	N/A	N/A	N/A	1
CYP1B1	N/A	N/A	N/A	N/A	N/A	N/A	N/A	-1
CYP1A1	N/A	N/A	N/A	N/A	N/A	N/A	N/A	-1
Brd4	1	N/A	N/A	N/A	N/A	N/A	N/A	N/A

SAMSN1	N/A	N/A	N/A	N/A	N/A	N/A	N/A	1
EML4-ALK	N/A	1	N/A	N/A	N/A	N/A	N/A	N/A
mir-133	-1	N/A	N/A	N/A	0	N/A	N/A	N/A
tributyrin	0	N/A	N/A	N/A	-1	N/A	0	0
isobutylmethylxanthine	0	N/A	N/A	N/A	0	N/A	N/A	-1
EGR2	N/A	N/A	N/A	N/A	N/A	N/A	N/A	-1
MEOX2	N/A	N/A	N/A	N/A	-1	N/A	N/A	N/A
CLOCK	N/A	N/A	0	N/A	N/A	-1	N/A	N/A
UBA1	N/A	N/A	N/A	N/A	-1	N/A	N/A	N/A
CYP1A2	N/A	N/A	N/A	N/A	N/A	N/A	N/A	-1
MMP1	N/A	N/A	N/A	1	N/A	N/A	N/A	N/A
CUL4B	N/A	N/A	N/A	N/A	N/A	N/A	-1	N/A
DOCK8	N/A	N/A	N/A	N/A	N/A	N/A	N/A	1
IL9	N/A	N/A	N/A	N/A	N/A	N/A	0	1
arsenic	N/A	N/A	N/A	N/A	N/A	N/A	0,975	N/A
HDAC4	0,152	N/A	N/A	0,152	-0,492	N/A	N/A	-0,132
STAT	N/A	N/A	N/A	N/A	N/A	N/A	N/A	0,927
aldosterone	N/A	N/A	N/A	N/A	0,765	N/A	N/A	0,152
GPS2	N/A	N/A	N/A	N/A	N/A	N/A	N/A	-0,9
ADAM10	N/A	N/A	N/A	0	-0,447	N/A	-0,447	N/A
PRNP	N/A	N/A	-0,447	0	0	N/A	0	-0,447
MAPKAPK2	N/A	N/A	N/A	N/A	N/A	N/A	N/A	0,882
tetraethylammonium	N/A	N/A	N/A	-0,152	-0,152	N/A	-0,555	N/A
RASSF5	N/A	N/A	N/A	0,849	N/A	N/A	N/A	N/A
RETNLB	N/A	N/A	N/A	N/A	0,849	N/A	N/A	N/A
phorbol esters	N/A	N/A	N/A	N/A	N/A	N/A	0,849	N/A
zVAD-FMK	N/A	-0,849	N/A	N/A	N/A	N/A	N/A	N/A
GDF2	0,849	N/A	N/A	N/A	0	N/A	N/A	N/A
Creb	N/A	N/A	N/A	N/A	0,737	N/A	N/A	-0,098
XDH	N/A	N/A	N/A	N/A	-0,832	N/A	N/A	N/A
kainic acid	N/A	N/A	N/A	N/A	0,824	N/A	N/A	N/A
MAP3K12	N/A	N/A	N/A	0	0	N/A	0,816	N/A
PLX5622	N/A	N/A	N/A	0	0	0	0,816	N/A
CTNNBIP1	N/A	N/A	N/A	N/A	N/A	N/A	N/A	0,811
HOTAIR	N/A	N/A	N/A	N/A	-0,779	N/A	N/A	N/A
cinnamaldehyde	N/A	N/A	N/A	N/A	0,775	N/A	N/A	N/A
MXD1	N/A	N/A	N/A	0,762	N/A	N/A	N/A	N/A
linoleic acid	N/A	N/A	N/A	N/A	-0,762	N/A	N/A	N/A
Ngf	N/A	N/A	N/A	N/A	N/A	N/A	N/A	-0,747
SPDEF	N/A	-0,025	N/A	N/A	N/A	N/A	N/A	0,714
PRKN	N/A	-0,55	N/A	N/A	N/A	N/A	N/A	-0,147
EGLN	N/A	N/A	N/A	0,152	0,152	N/A	-0,391	N/A
BNIP3L	-0,343	N/A	N/A	N/A	-0,343	N/A	N/A	N/A
ERG	N/A	N/A	N/A	N/A	-0,128	N/A	-0,555	N/A
pioglitazone	N/A	N/A	-0,152	N/A	-0,128	N/A	0,391	N/A
CAV1	N/A	0,654	N/A	N/A	N/A	N/A	N/A	N/A
INHBA	N/A	N/A	N/A	N/A	-0,618	N/A	N/A	N/A
bardoxolone	0	N/A	0	0	-0,115	0	0	0,492
Notch	N/A	N/A	N/A	N/A	0,6	N/A	N/A	N/A
mir-15	-0,297	N/A	N/A	N/A	N/A	N/A	N/A	-0,297
salirasib	N/A	-0,594	N/A	N/A	N/A	N/A	N/A	N/A
isoproterenol	N/A	N/A	N/A	N/A	-0,59	N/A	N/A	N/A
EWSR1-FLI1	-0,277	N/A	N/A	0,152	0,152	N/A	N/A	N/A
dichlorovinylcysteine	N/A	N/A	N/A	N/A	N/A	N/A	-0,577	N/A
cyclic AMP	N/A	N/A	N/A	N/A	0	-0,555	N/A	N/A
cadmium chloride	N/A	N/A	N/A	N/A	-0,555	N/A	N/A	0
TGFBR1	N/A	N/A	N/A	N/A	0,555	N/A	N/A	N/A
CXCL8	N/A	N/A	N/A	0,277	N/A	N/A	0,277	N/A
ABL1	N/A	0,537	N/A	N/A	N/A	N/A	N/A	N/A
HMGB1	N/A	N/A	N/A	N/A	N/A	N/A	0,512	N/A
GLI2	N/A	N/A	N/A	0,254	N/A	N/A	N/A	0,254
HSPA5	N/A	N/A	N/A	0	-0,447	N/A	0	N/A
PSMB11	N/A	N/A	N/A	N/A	N/A	N/A	-0,447	N/A
ammonium	N/A	N/A	N/A	0,447	N/A	N/A	N/A	N/A
BMP7	N/A	N/A	N/A	N/A	0,437	N/A	N/A	N/A
mono-(2-ethylhexyl)phthalate	N/A	N/A	N/A	-0,218	-0,218	N/A	N/A	N/A
bisphenol A	N/A	N/A	N/A	-0,152	N/A	N/A	0,277	N/A
PRKAA2	N/A	-0,412	N/A	N/A	N/A	N/A	N/A	N/A
THRA	N/A	N/A	N/A	N/A	N/A	N/A	0,277	0,13
HDAC5	N/A	N/A	N/A	N/A	-0,391	N/A	N/A	N/A
Pkc(s)	N/A	N/A	N/A	N/A	0,391	N/A	N/A	N/A
nitroprusside	N/A	0,391	N/A	N/A	N/A	N/A	N/A	N/A
MIR17HG	N/A	N/A	N/A	N/A	0	N/A	N/A	-0,391
FHL2	N/A	N/A	N/A	N/A	-0,371	N/A	N/A	N/A
PRKCE	N/A	N/A	N/A	N/A	0,339	N/A	N/A	N/A
ID2	N/A	N/A	N/A	N/A	N/A	-0,339	N/A	N/A
SQSTM1	N/A	N/A	N/A	N/A	-0,334	N/A	N/A	N/A
VTN	N/A	N/A	N/A	N/A	N/A	N/A	N/A	-0,322
ATF3	N/A	0,294	N/A	N/A	N/A	N/A	N/A	N/A
fluoxetine	N/A	N/A	N/A	0,293	N/A	N/A	N/A	N/A
SNAI2	N/A	N/A	N/A	N/A	-0,289	N/A	N/A	N/A
A2M	N/A	N/A	N/A	N/A	-0,277	N/A	N/A	N/A
LONP1	N/A	N/A	N/A	N/A	N/A	N/A	-0,277	N/A
TERT	N/A	0,277	N/A	N/A	N/A	N/A	N/A	N/A
3-methyladenine	N/A	N/A	N/A	N/A	-0,277	N/A	N/A	N/A
staurosporine	N/A	N/A	N/A	0	0,106	N/A	N/A	-0,152
dimethylnitrosamine	N/A	N/A	N/A	N/A	0,254	N/A	N/A	N/A
2-deoxyglucose	N/A	N/A	N/A	N/A	N/A	N/A	N/A	-0,254
TFEB	N/A	N/A	N/A	N/A	N/A	N/A	0,254	N/A
fingolimod	N/A	N/A	N/A	N/A	N/A	N/A	0,218	N/A
TFAP2C	N/A	N/A	N/A	N/A	N/A	N/A	N/A	-0,218
RUNX3	N/A	N/A	N/A	N/A	N/A	N/A	N/A	-0,157
mir-145	N/A	N/A	N/A	N/A	-0,156	N/A	N/A	N/A
lipoxin A4	N/A	N/A	N/A	N/A	0,152	N/A	N/A	N/A
Ras homolog	N/A	N/A	N/A	N/A	N/A	N/A	N/A	-0,152
MMP3	N/A	N/A	N/A	N/A	N/A	N/A	0,152	N/A
GJA1	N/A	N/A	N/A	N/A	-0,152	N/A	N/A	N/A
LMNA	N/A	N/A	N/A	N/A	-0,152	N/A	N/A	N/A
losartan potassium	N/A	N/A	N/A	N/A	0,152	N/A	N/A	N/A
latrunculin B	N/A	N/A	N/A	N/A	-0,152	N/A	N/A	N/A
17-alpha-ethinylestradiol	N/A	0,132	N/A	N/A	N/A	N/A	N/A	N/A
STAT5A	N/A	0,128	N/A	N/A	N/A	N/A	N/A	N/A
SOX17	0,128	N/A	N/A	N/A	N/A	N/A	N/A	N/A
ZEB1	N/A	N/A	N/A	0,061	N/A	N/A	N/A	0,061
RORA	N/A	0,106	N/A	N/A	N/A	N/A	N/A	N/A
CCL2	N/A	N/A	N/A	N/A	N/A	0,106	N/A	N/A
DDIT3	N/A	N/A	N/A	N/A	0,105	N/A	N/A	N/A
miR-9-5p (and other miRNAs w/seed CUUUGGU)	N/A	N/A	N/A	N/A	0,085	N/A	N/A	N/A
berberine	N/A	N/A	N/A	0	N/A	N/A	N/A	-0,068
PIAS1	N/A	N/A	N/A	N/A	-0,063	N/A	N/A	N/A

Table S4 Antibody information

Antibody	Dilution	Company	Clone
anti-Nestin	1:100	Invitrogen (Human Neural Stem Cell Immunocytochemistry Kit, A24354)	N/A
anti-PAX6	1:100	Invitrogen (Human Neural Stem Cell Immunocytochemistry Kit, A24354)	N/A
anti-SOX1	1:100	Invitrogen (Human Neural Stem Cell Immunocytochemistry Kit, A24354)	N/A
anti-SOX2	1:100	Invitrogen (Human Neural Stem Cell Immunocytochemistry Kit, A24354)	N/A
anti-β-tubulin III	1:250	Biolegend (801201)	TUJ1
anti-MAP2	1:100	Santa Cruz (sc-74421)	A-4
anti-IL17R	1:50	Santa Cruz (sc-376374)	G-9
anti-TNF-R1	1:100	Santa Cruz (sc-8436)	H-5
AF 488 anti NF-H (SMI32)	1:250	Biolegend (801706)	SMI 32
AF 594 anti NF-H (SMI31)	1:200	Biolegend (801610)	SMI 31
AF 647 anti NF-L	1:200	Biolegend (845908)	NFL3
anti-AQP4	1:100	Elabscience (E-AB-64864)	Polyclonal
anti-GFAP	1:250	Agilent (GA52461-2)	Polyclonal
Goat anti-Rabbit Alexa fluor 488	1:1000	Invitrogen (A-11008)	Polyclonal
Goat anti-Mouse Alexa fluor 488	1:1000	Invitrogen (A-11001)	Polyclonal
Goat anti-Rabbit Alexa fluor 594	1:1000	Invitrogen (A-11012)	Polyclonal
Goat anti-Mouse Alexa fluor 594	1:1000	Invitrogen (A-11005)	Polyclonal
